# Synthesis and Pharmacological Evaluation of Novel 1,5-Disubstituted-3-amino-1,2,4-triazoles Designed as Multitarget Directed Ligands for Alzheimer’s Disease Targets

**DOI:** 10.1021/acsomega.5c09002

**Published:** 2026-02-17

**Authors:** Daiana Portella Franco, Lucas Caruso, Danniel Cosme Neves Grillo, Nathália Fonseca Nadur, Luciana Luiz de Azevedo, Thiago Moreira Pereira, Manuelle Cunha da Silva, Renata Barbosa Lacerda, Pedro de Sena Murteira Pinheiro, Cristiano Jorge Riger, Arthur Eugen Kümmerle

**Affiliations:** † Laboratory of Molecular Diversity and Medicinal Chemistry (LaDMol-QM), Department of Organic Chemistry, Institute of Chemistry, 67825 Federal Rural University of Rio de Janeiro, Seropédica 23897-000, Rio de Janeiro, Brazil; ‡ Laboratory of Oxidative Stress in Microorganisms, Department of Biochemistry, Institute of Chemistry, Federal Rural University of Rio de Janeiro, Seropédica 23897-000, Rio de Janeiro, Brazil; § Department of Pharmaceutical Sciences, Institute of Biological and Health Sciences, Federal Rural University of Rio de Janeiro, Seropédica 23897-000, Rio de Janeiro, Brazil; ∥ Laboratório de Avaliação e Síntese de Substâncias Bioativas (LASSBio), Instituto de Ciências Biomédicas, 28125 Universidade Federal do Rio de Janeiro, P.O. Box 68023, Rio de Janeiro 21941-902, Rio de Janeiro, Brazil

## Abstract

A series of 3-amino-1,2,4-triazole derivatives was synthesized and evaluated for their multitarget activities relevant to Alzheimer’s disease. Inhibition assays revealed potent and preferential inhibition of acetylcholinesterase (AChE) for most of compounds, with IC_50_ values of up to 0.38 μM and selectivity ratios up to 32-fold over butyrylcholinesterase (BChE). Qualitative molecular dynamics indicated that interactions at the PAS and CAS appear to occur synergistically, with positive cooperativity. Electron-withdrawing groups in R_1_ and R_2_ favorize PAS interactions that seem to guide efficient CAS interactions, mainly with residue Trp86 in AChE and Trp107 in BChE. Metal-binding studies showed intrinsic complexation of Cu^2+^ and Fe^3+^ for the 3-amino-1,2,4-triazole compounds, which could be expanded to other metals by specific structural modifications in *ortho*-position of R_1_ substituent. Selected derivatives also demonstrated low toxicity and protective antioxidant effects in *Saccharomyces cerevisiae*, significantly reducing lipid peroxidation.

## Graphical abstract

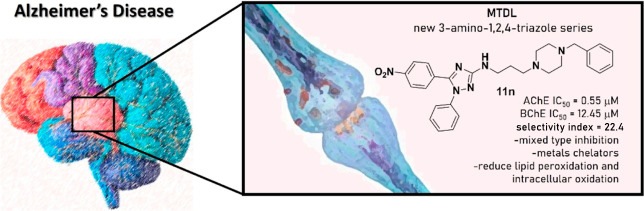

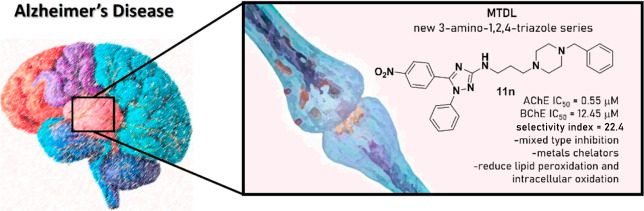

## 1 Introduction

Alzheimer’s disease (AD) is an irreversible, progressive neurodegenerative disorder that impairs cognitive functions.^1^ According to Alzheimer’s Disease International, 50 million people worldwide currently live with dementia, and this number could triple by 2050, with AD being the primary cause.^2,3^ Linked to aging and with a multifactorial etiology, AD features synaptic alterations, filamentous aggregates of amyloid-β protein (Aβ), and intracellular neurofibrillary tangles of hyperphosphorylated tau protein.^4^ While these changes can occur in healthy elderly brains, they are less frequent and less intense than in those with AD.^5^

Research into AD therapy initially centered on the cholinergic hypothesis, prompted by the profound dysfunction observed in this neurotransmitter system.^6^ As a result, cholinesterase inhibitors (ChEIs) emerged as the first pharmacologically effective class of drugs approved for AD management.^7^ Acetylcholinesterase (AChE) and butyrylcholinesterase (BChE) are serine hydrolases that catalyze the breakdown of the neurotransmitter acetylcholine (ACh). Under physiological conditions, AChE accounts for roughly 95% of total cholinesterase activity in the brain and exhibits high substrate specificity, rapidly hydrolyzing up to 6 × 10^5^ ACh molecules per minute.^7–9^ In contrast, BChE displays lower catalytic efficiency toward ACh and a broader substrate profile, contributing to the hydrolysis of other esters such as butyrylcholine.^10^ Notably, in advanced stages of AD, BChE activity increases by more than 120%, while AChE levels decline significantly (by approximately 55–67%). This shift highlights BChE as a promising target for the development of novel biomarkers and therapeutic inhibitors.^11^

The pathophysiology of Alzheimer’s disease encompasses a wide range of biochemical processes and molecular targets. This complexity reduces the efficacy of therapeutic strategies aimed at a single biological target, such as cholinesterase inhibition alone. As a result, multiple target-directed ligands (MTDLs) have gained increasing attention as a therapeutic strategy for neurodegenerative disorders. Within this framework, acetylcholinesterase remains the most extensively investigated target, followed by pathways related to β-amyloid aggregation, oxidative stress, butyrylcholinesterase activity, and metal ion dysregulation.^12–16^

The 1,2,4-triazole heterocycle is a highly versatile chemical and biological scaffold. Numerous derivatives (**1–7**) have been identified as cholinesterase inhibitors, many of which show promise for the development of MTDLs (Figure 1).^17,18^ Recently, our group reported a regioselective approach for the synthesis and functionalization of 1,5-diaryl-3-amino-1,2,4-triazoles (**5**). This valuable synthesis was compared to other 1,2,4-triazole synthesis unveiling a novel synthetic decoration process of this scaffold for cholinesterase inhibition.^19^ To our knowledge, our described 1,5-disubstituted-3-amino-1,2,4-triazole nucleus (**5**) was the first and only example of cholinesterase inhibitors belonging to this chemical class, which remained underexplored.^19^

**Figure 1:**
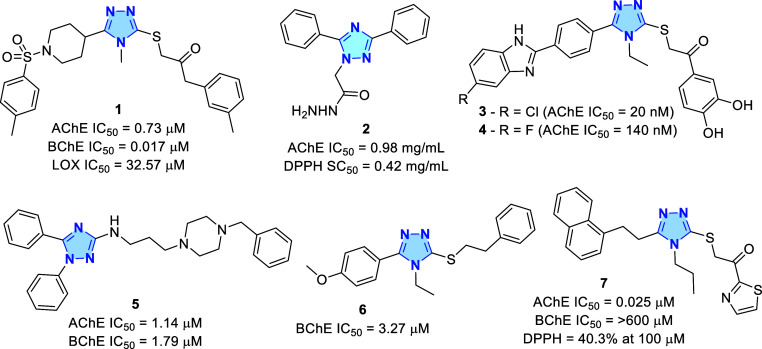
1,2,4-Triazole containing cholinesterase inhibitors and potential MTDLs.

Our original series of 1,5-diaryl-3-amino-1,2,4-triazoles (**10**) was designed through bioisosterism between triazine ring from (**8**), described as cholinesterase inhibitor and neuroprotector,^20^ and the 1,2,4-triazole.^19^ Additionally, we used molecular hybridization to insert a benzyl-piperazine group as a mimetic pharmacophore moiety of donepezil drug (**9**).^21,22^ In this study, we identified the three methylene spacer as best decoration for AChE inhibition. However, no further modifications were explored.

In this study, we aim to further develop this class of cholinesterase inhibitors by synthesizing new analogues through modifications on R_1_ and R_2_ (Figure 2), while retaining the aminoalkyl-benzylpiperazine moiety, previously identified as crucial for activity. Additionally, selected compounds will be evaluated as promising MTDL candidates, with emphasis on their in vitro metal-chelating capabilities (Zn^2+^, Cu^2+^, Fe^3+^, and Al^3+^) and eukaryotic antioxidant properties.

**Figure 2:**
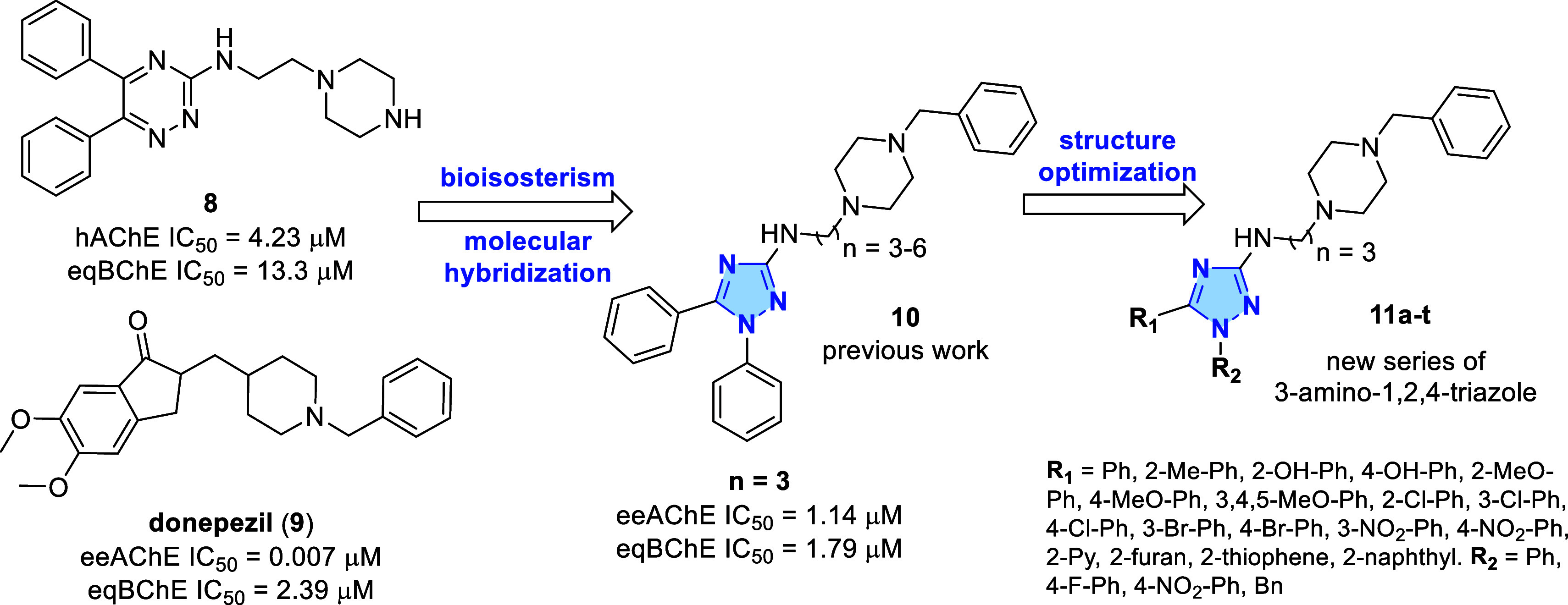
Design of a novel series of 3-amino-1,2,4-triazoles **11a–t**.

## 2 Results and Discussion

### 2.1 Synthesis of Desired Compounds

The synthesis of the designed new compounds began with the synthesis of *S*-methylisothiourea (**13**) through alkylation of thiourea (**12**) using methyl iodide, followed by product precipitation and filtration, with 90% yield. The second step was the monoprotection of **13** to produce *N*-Boc-*S*-methylisothiourea (**14**) (necessary step for the regioselective cyclization)^19^ in 85% yield, which was then reacted with various acyl chloride derivatives (commercial or synthesized) to furnish the *N*-protected-*N*-acyl-*S*-methylisothiourea intermediates **15a–r** (Scheme 1). The regioselective cyclization step, leading to *N*-protected 1,5-disubstituted-3-amino-1,2,4-triazoles (**16a–t**), involved reacting the *N*-protected *N*-acyl-*S*-methylisothiourea derivatives (**15a–r**) with different phenylhydrazines in acetonitrile at 100 °C.^19^ As previously described by our group, microwave irradiation proved to be efficacious in increasing reaction yields in this step, leading to the protected products **16a–t** in yields ranging from 45 to 89% in 40–60 min. The desired final 1,5-disubstituted-3-amino-1,2,4-triazole derivatives (**11a–t**) were obtained through three additional steps. First, an S_N_2 alkylation of **16a–t** with 1,3-dibromopropane leading to the alkylated products **17a–t**. Subsequently, an amination reaction of **17a–t** with benzylpiperazine produced **18a–t**, followed by their deprotections using trifluoroacetic acid.^19^ We also synthesized derivative **21**, which is a compound with a pharmacophore transposition from amino group to the **R**_**2**_ position (Scheme 2). Compound **21** was obtained in a similar fashion as described above in two steps. First, the *N*-Boc-*N*-acyl-*S*-methylisothiourea intermediate **15a** was reacted with 1-benzyl-4-hydrazineylpiperidine (**20**), prepared from reductive amination of benzylpiperidinone (**19**) with benzoylhydrazine, followed by hydrolysis with HCl.^23^ Subsequently, a deprotection with TFA gave the 1-benzylpiperidinyl-1,2,4-triazol derivative **21** in 90% yield (Scheme 2).

**Scheme 1:**
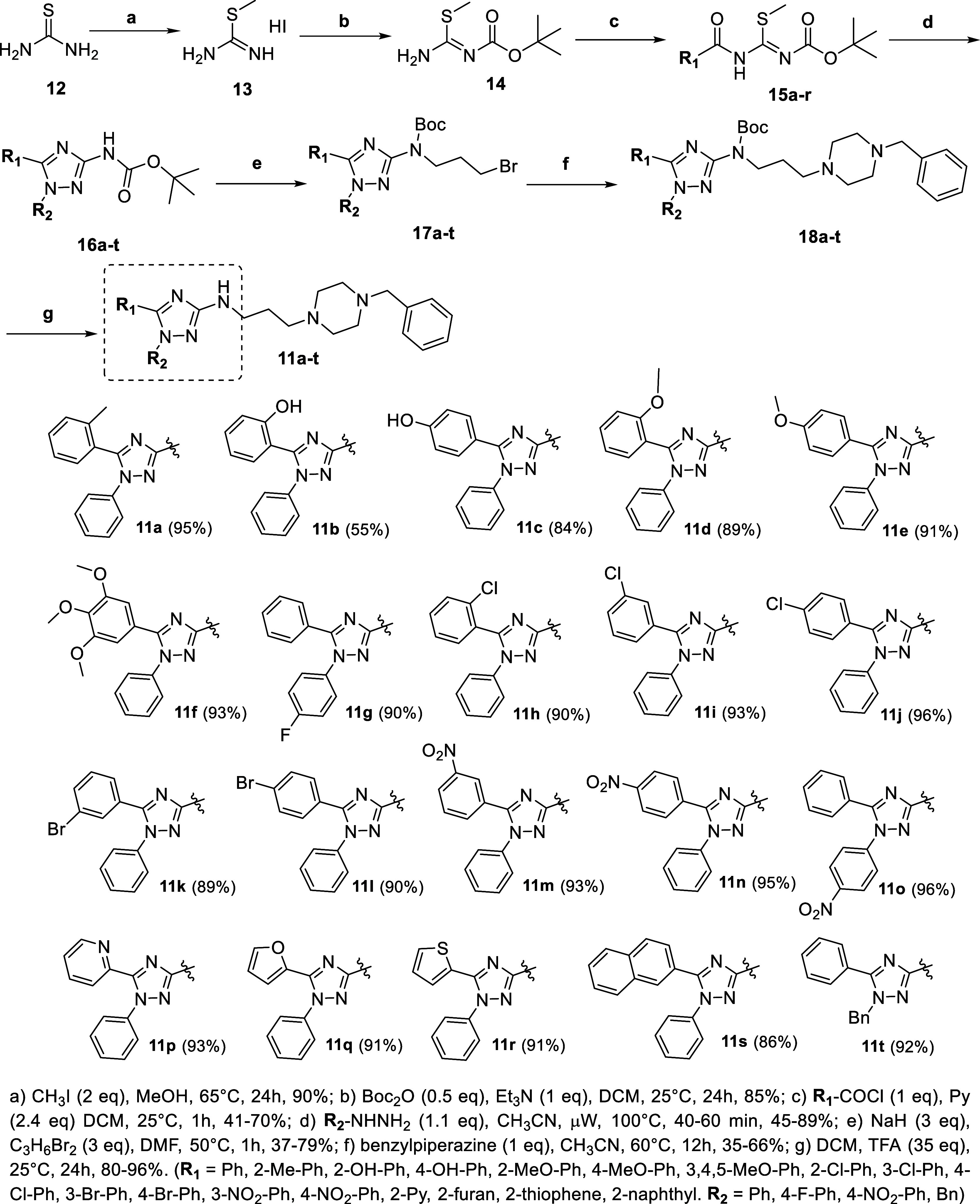
Reagents and Reaction Conditions Used for the Synthesis of the Compounds **11a–t**

**Scheme 2:**
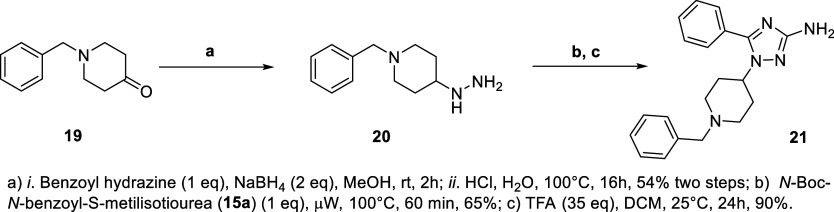
Reagents and Conditions for the Synthesis of Compound **21**

### 2.2 Cholinesterase Inhibition Evaluation

The inhibitory activities of the 1,5-diaryl-3-amino-1,2,4-triazoles compounds (**11a–t** and **21**) on AChE and BChE were determined by the Ellman’s method^24^ using donepezil (**9**) as the reference compound^22,25,26^ (Table 1).

**Table 1:**
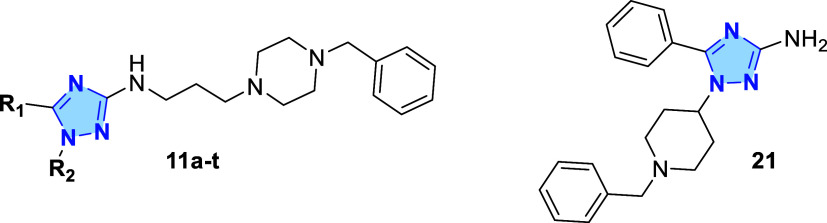
AChE and BChE Inhibitory Activities of New Series of 1,5-Disubstituted-3-amino-1,2,4-triazoles

			IC_50_ (μM) ± SD^a^		
compound	R_1_	R_2_	AChE^b^	BChE^c^	SI^d^
**5**	Ph	Ph	1.14 ± 0.03^e^	1.79 ± 0.06^e^	1.57^e^
**11a**	2-CH_3_-Ph	Ph	3.11 ± 0.21	4.16 ± 0.37	1.34
**11b**	2-OH-Ph	Ph	2.25 ± 0.21	1.29 ± 0.09	0.58
**11c**	4-OH-Ph	Ph	1.85 ± 0.02	7.80 ± 0.49	4.20
**11d**	2-MeO-Ph	Ph	2.57 ± 0.17	1.57 ± 0.06	0.61
**11e**	4-MeO-Ph	Ph	1.68 ± 0.12	11.48 ± 0.56	6.82
**11f**	3,4,5-MeO-Ph	Ph	1.36 ± 0.13	10.24 ± 0.28	7.48
**11g**	Ph	4-F-Ph	1.04 ± 0.06	3.35 ± 0.15	3.20
**11h**	2-Cl-Ph	Ph	1.75 ± 0.11	4.95 ± 0.47	2.82
**11i**	3-Cl-Ph	Ph	1.67 ± 0.16	4.03 ± 0.19	2.41
**11j**	4-Cl-Ph	Ph	2.16 ± 0.11	8.21 ± 0.57	3.80
**11k**	3-Br-Ph	Ph	2.55 ± 0.14	4.12 ± 0.15	1.62
**11l**	4-Br-Ph	Ph	2.19 ± 0.16	5.68 ± 0.08	2.59
**11m**	3-NO_2_-Ph	Ph	0.41 ± 0.01	6.48 ± 0,26	15.74
**11n**	4-NO_2_-Ph	Ph	0.55 ± 0.05	12.45 ± 0.37	22.43
**11o**	Ph	4-NO_2_-Ph	0.38 ± 0.02	2.07 ± 0.17	5.36
**11p**	2-Py	Ph	2.91 ± 0.28	9.33 ± 0.45	3.20
**11q**	2-furanyl	Ph	3.14 ± 0.28	4.17 ± 0.27	1.33
**11r**	2-thiophenyl	Ph	2.37 ± 0.17	3.25 ± 0.21	1.37
**11s**	2-naphthyl	Ph	1.29 ± 0.13	0.63 ± 0.03	0.49
**11t**	Ph	Bn	2.35 ± 0.23	0.76 ± 0.04	0.32
**21**	-	-	>500	88.23 ± 0.59	-
donepezil (**9**)	-	-	0.007 ± 0.0002^f^	2.39 ± 0.11^f^	341.43^f^

a Concentration required to inhibit 50% of cholinesterase activity, data obtained ± standard deviation (SD) in triplicates of independent assays.

b AChE from electric eel.

c BChE for horse serum.

d Selectivity index (SI) is defined as BChE IC_50_/AChE IC_50_.

e Results obtained by Santos et al.^19^

f Results obtained by De Souza et al.^22^

In general, the new derivatives substituted with electron-donating groups on the phenyl ring at R_1_ (**11a–f**) (AChE IC_50_ range = 1.36–3.11 μM) were slightly less active against AChE than the previously described derivative (**5**: R_1_ = Ph, R_2_ = Ph; AChE IC_50_ = 1.14 μM and BChE IC_50_ = 1.79 μM). The same was observed for halogenated derivatives (**11h–l**) with AChE IC_50_ ranging from 1.67 to 2.55 μM. Substitution of the phenyl group by heteroaromatics (**11p–r**) and 2-naphthyl (**11s**) at R_1_ on triazole ring was harmful for activity against AChE (IC_50_ range = 1.29–3.14 μM).

However, the addition of the strong electron-withdrawing nitro group (**11m**, *meta*-NO_2_, and **11n**, *para*-NO_2_) was favorable to the AChE inhibition and selectivity against BChE with AChE IC_50_ of 0.41 μM and SI of 15.74 for **11m**, and AChE IC_50_ of 0.55 μM and SI of 22.43 for **11n**. These nitro-substituted compounds were 2.8 (**11m**) and 2.1 (**11n**) times more potent than derivative **5**, and up to 14 times more selective compared to BChE inhibition. The nitro groups were the only ones that increased the AChE inhibition potency, indicating a possible importance electron-withdrawing substituents. Indeed, the addition of electron-withdrawing groups to the phenyl group at R_2_ also resulted in improvement in activity against AChE. Compound **11g** was slightly more potent against AChE (4-F-Ph; IC_50_ = 1.04 μM) and analog **11o** (4-NO_2_-Ph at R_2_) was the most potent compound against AChE (IC_50_ = 0.38 μM, 3 times more potent than **5**). Despite being the most potent compound, **11o** displayed potency comparable to **11m** and **11n**, but with a markedly reduced selectivity (SI = 5.36). In terms of AChE inhibition, substitution at the phenyl-R_1_ position resulted in better activity than substitution at the phenyl-R_2_ position. Clearly, the above results have shown that AChE inhibition by our 1,2,4-triazoles is governed by electronic effects instead of steric factors.

Conversely, the bulkier derivatives **11s** (R_1_ = 2-naphthyl, R_2_ = Ph) and **11t** (R_1_ = Ph, R_2_ = Bn) were unexpectedly more potent against BChE, with IC_50_ values of 0.63 and 0.76 μM, respectively. These compounds also exhibited higher selectivity for BChE over AChE, suggesting that steric effects may play a more significant role in BChE inhibition. Finally, the pharmacophore transposed derivative (**21**) was inactive against AChE (IC_50_ > 500 μM) and showed very low activity against BChE (IC_50_ = 88.23 μM) as expected.

### 2.3 Kinetic Enzymatic Evaluation

The inhibition profile was evaluated using the linearized Lineweaver–Burk form of the Michaelis–Menten equation. For these evaluations, representative compounds were selected to reflect the overall behavior of the series. We chose the most potent AChE inhibitor (**11o**) and the second most potent BChE inhibitor (**11t**), the latter being the only compound bearing a benzyl group instead of a phenyl substituent. This structural modification in **11t** may alter both the steric and electronic properties of the 1,2,4-triazole core in a manner distinct from the other derivatives, potentially influencing its mechanism of action.

Increasing concentrations of compounds **11o** and **11t** led to higher *K*_m_ values and reduced *V*_max_, indicating a mixed-type inhibition pattern for both AChE and BChE. This indicates that the inhibitors can bind to both the catalytic (CAS), competitive inhibition, and the allosteric (PAS), noncompetitive inhibition, sites in AChE and BChE (Table 2 and Figure 3). These results are in agreement with previously described by our group for compound **5**.^19^ Importantly, despite its distinct substitution pattern, compound **11t** still exhibits a mixed-type inhibition profile against cholinesterases.

**Table 2:** Kinetic Parameters of **11o** and **11t** in AChE and BChE[Table-fn t2fn3]

conc. (μM)	*V*_max_ ± SD (μM/min)	*K*_m_ ± SD (μM)	*K*_i_ (nM) ± SD^b^	*K*_i_^′^ (nM) ± SD[Table-fn t2fn3]
**11o** in AChE				
0	5.89 ± 0.38	40.2 ± 3.3		
0.3	5.67 ± 0.01	76.7 ± 2.3	0.071 ± 0.002	0.350 ± 0.006
0.5	4.36 ± 0.15	80.9 ± 6.5		
**11o** in BChE				
0	6.83 ± 0.36	85.9 ± 3.6		
1	6.02 ± 0.19	124 ± 6	1.8 ± 0.1	4.8 ± 0.3
3	5.88 ± 0.23	172 ± 1		
**11t** in AChE				
0	7.16 ± 0.14	57.8 ± 2.7		
1.4	5.00 ± 0.21	67.1 ± 0.8	0.67 ± 0.017	2.51 ± 0.05
3.4	3.50 ± 0.15	89.9 ± 5.4		
**11t** in BChE				
0	5.23 ± 0.07	101 ± 4		
0.7	3.43 ± 0.20	256 ± 6	0.16 ± 0.002	0.42 ± 0.005
0.9	2.62 ± 0.19	272 ± 12		

a *K*_i_ is the competitive inhibition constant.

b *K*_i_^′^ is the noncompetitive inhibition constant.

**Figure 3:**
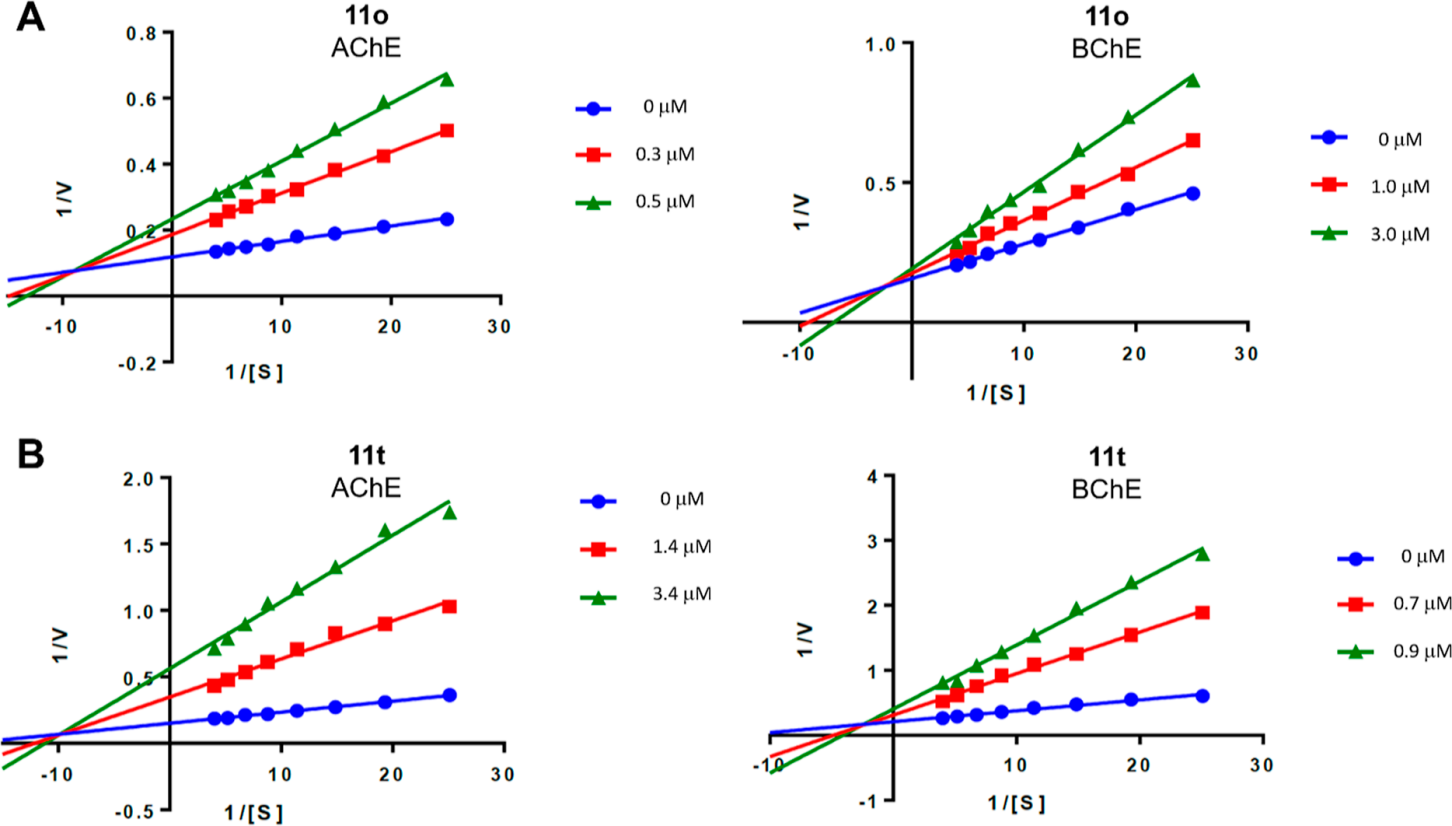
Lineweaver–Burk plots of AChE and BChE inhibition kinetics of compounds **11o** (A) and **11t** (B).

### 2.4 Docking and Molecular Dynamics Simulations

Docking for all compounds was carried out with acetylcholinesterase (AChE, PDB ID: 1C2B)^27^ and butyrylcholinesterase (BChE), for which a homology model was generated using the Swiss-Model server^28^ based on the crystal structure of PDB ID: 4EY7. The enzyme used in the biological assays was derived from horse serum, and since no crystal structures of horse BChE are currently available in the Protein Data Bank (PDB), the model was built using the horse BChE sequence. The 4EY7 structure was selected as the template because of its high sequence identity and structural similarity to the horse enzyme, ensuring the generation of a reliable model representative of the biological target used experimentally. Importantly, this template contains donepezil cocrystallized in the active site, which allowed the resulting model to incorporate the induced-fit conformation of the binding pocket. This structural feature was particularly advantageous for the subsequent docking studies, as it provided a conformationally realistic representation of the active site—preorganized to accommodate ligands through the same interactions observed experimentally with donepezil. This approach ensured consistency between the in vitro and in silico analyses and enhanced the reliability of the docking results.

The compounds were docked into acetyl- and butyrylcholinesterases with the aim of understanding their interaction modes and how simple structural modifications resulted in significant changes in potency and selectivity between the two enzymes. Based on the preferential binding poses in each enzyme, short (10 ns) molecular dynamics simulations were initially carried out, followed by trajectory-based free energy calculations (MM/GBSA) to obtain the relative binding free energy of each compound against each enzyme. This step was designed as a computationally efficient comparative approach to estimate relative affinities among the compounds, rather than to assess full conformational equilibration. However, neither the docking scores nor the calculated Δ*G* values (data not shown) directly corroborated the experimentally determined IC_50_ values.

From a qualitative perspective, the interactions at the PAS and CAS appear to occur synergistically, with positive cooperativity, where PAS interactions seem to guide efficient CAS interactions, mainly with residue Trp86 in AChE and Trp107 in BChE. This can be observed in the interaction modes of compounds **5**, **11c**, **11n**, and **11t** in AChE (Figure 4A–D) and in BChE (Figure 4E–H). At first glance, this positive cooperativity seems to be driven by efficient π and T-stacking interactions in the PAS with residues Trp286 in AChE and Phe307 in BChE, which also appear to be reinforced by additional interactions with residues Tyr72 and Tyr341 in AChE, and Tyr93 and Tyr362 in BChE (Figure 4A–H). Additionally, it is worth noting that a hydrogen bond was observed between the nitro group of **11n** and residue Tyr72; however, this same interaction was not observed in BChE.

**Figure 4:**
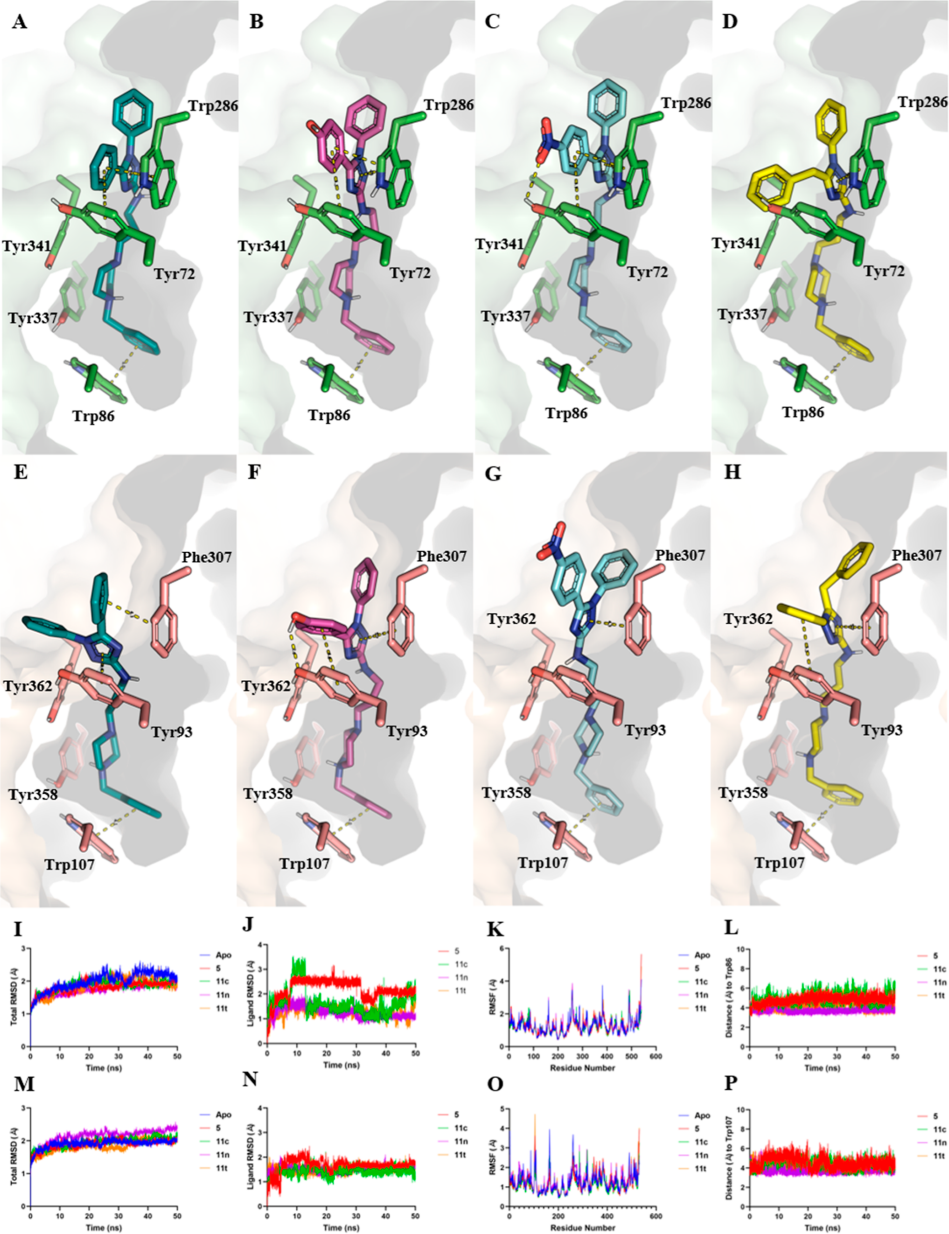
Binding modes and molecular dynamics simulations. (A–D) Interaction modes of compounds **5**, **11c**, **11n**, and **11t**, respectively, in AChE. (E–H) Interaction modes of compounds **5**, **11c**, **11n**, and **11t**, respectively, in BChE. (I) Total RMSD of AChE. (J) Ligand RMSD in AChE. (K) RMSF of AChE. (L) Distance between the terminal benzyl group and residue Trp86 in AChE. (M) Total RMSD of BChE. (N) Ligand RMSD in BChE. (O) RMSF of BChE. (P) Distance between the terminal benzyl group and residue Trp107 in BChE.

To further investigate the comparative stability of the complexes and the persistence of key interactions predicted by docking, longer (50 ns) molecular dynamics simulations were subsequently performed. Since the steric volume of the substituents does not appear to be the key parameter governing compound interactions, we selected four representative derivatives to investigate the influence of electronic effects on activity. Thus, we picked **5** as reference (R_1_ = Ph), **11c** (R_1_ = 4-OH-Ph, EDG), **11n** (R_1_ = 4-NO_2_-Ph, EWG) and **11t** (R_2_ = Bn, superior homologous) (Figure 4I–P). Overall, the backbone RMSD stabilized around 2 Å for all systems, indicating that ligand binding did not induce significant conformational rearrangements at the global level (Figure 4I,M). Similar behavior was observed for the apo forms, suggesting that the ligands mainly exploit local interactions within the active site without perturbing the overall structural stability of the enzymes.

Analysis of ligand RMSD showed values below 2 Å in most cases, confirming stable binding poses throughout the simulation time (Figure 4J,N). The only exception was compound **5** in AChE, which exhibited slightly higher fluctuations with an average RMSD between 2 and 3 Å (Figure 4N). Nevertheless, the variation remained small and is likely related to the higher conformational flexibility of this compound, without evidence of loss of global orientation within the binding site. Consistently, RMSF profiles did not reveal significant differences between liganded and apo forms or among different complexes, reinforcing that the observed selectivity does not arise from global protein dynamics but rather from localized interactions involving aromatic and polar residues of the binding pocket (Figure 4K,O).

To assess the persistence of the aromatic interactions predicted by docking, the distance between the terminal benzyl group, common to all compounds, and key aromatic residues in the CAS was monitored during 50 ns of simulation. Stable distances of approximately 4 Å were maintained in all systems, consistent with productive π–π-stacking geometries (Figure 4L,P). In AChE, these interactions were observed with Trp86 in the CAS, while in BChE analogous behavior was maintained with Trp107 in the CAS. The stability of these interactions throughout the trajectories confirms the role of PAS anchoring as a cooperative element that guides the ligands toward efficient CAS engagement.

### 2.5 Study of the Complexation of the Derivatives with Cu^2+^, Zn^2+^, Al^3+^ and Fe^3+^

Increasing evidence suggests that metal ions, including iron, copper, and zinc, are dysregulated in brain regions that are particularly vulnerable in Alzheimer’s disease. This imbalance is closely associated with the accumulation of Aβ plaques, tau hyperphosphorylation, neuronal loss, and neuroinflammation.^29^ Consequently, metal chelation therapy has emerged as a promising strategy to restore metal homeostasis and mitigate disease progression.^29^

In order to evaluate the ability of our compounds to complex with metals relevant to Alzheimer’s disease, derivative **5** (R_1_ and R_2_ = Ph) was chosen as reference compound for the whole series, while **11b** (R_1_ = 2-OH-Ph and R_2_ = Ph) and **11p** (R_1_ = 2-Py and R_2_ = Ph) were selected because of their putative complexing sites.^30,31^ The UV/vis spectrum (Figure S1, Supporting Information) of derivatives showed a maximum absorption at 287 nm (**5**) and 279 nm (**11b** and **11p**), and after the addition of the salts no significant variations were observed. Since there was no change in the absorption spectrum of the derivatives in the presence of the salts, we decided to evaluate the influence on the emission spectrum. The derivatives **5**, **11b** and **11p** showed fluorescence emission maximum at 439 nm, 424 nm and 459 nm, respectively. Fluorescence analysis (Figure 5) demonstrated that the excitation of compound **5** at 287 nm generated a change in the emission spectrum in the presence of Cu^2+^ and Fe^3+^ ions, indicating the formation of the 1,2,4-triazole Cu^2+^- and Fe^3+^-complexes with these metals. The same was not observed in the presence of Zn^2+^ and Al^3+^. The results obtained with compound **5** demonstrate that the intrinsic 3-amino-1,2,4-triazole scaffold serves as an effective metal-complexing center, as expected for the other compounds in the series.

**Figure 5:**
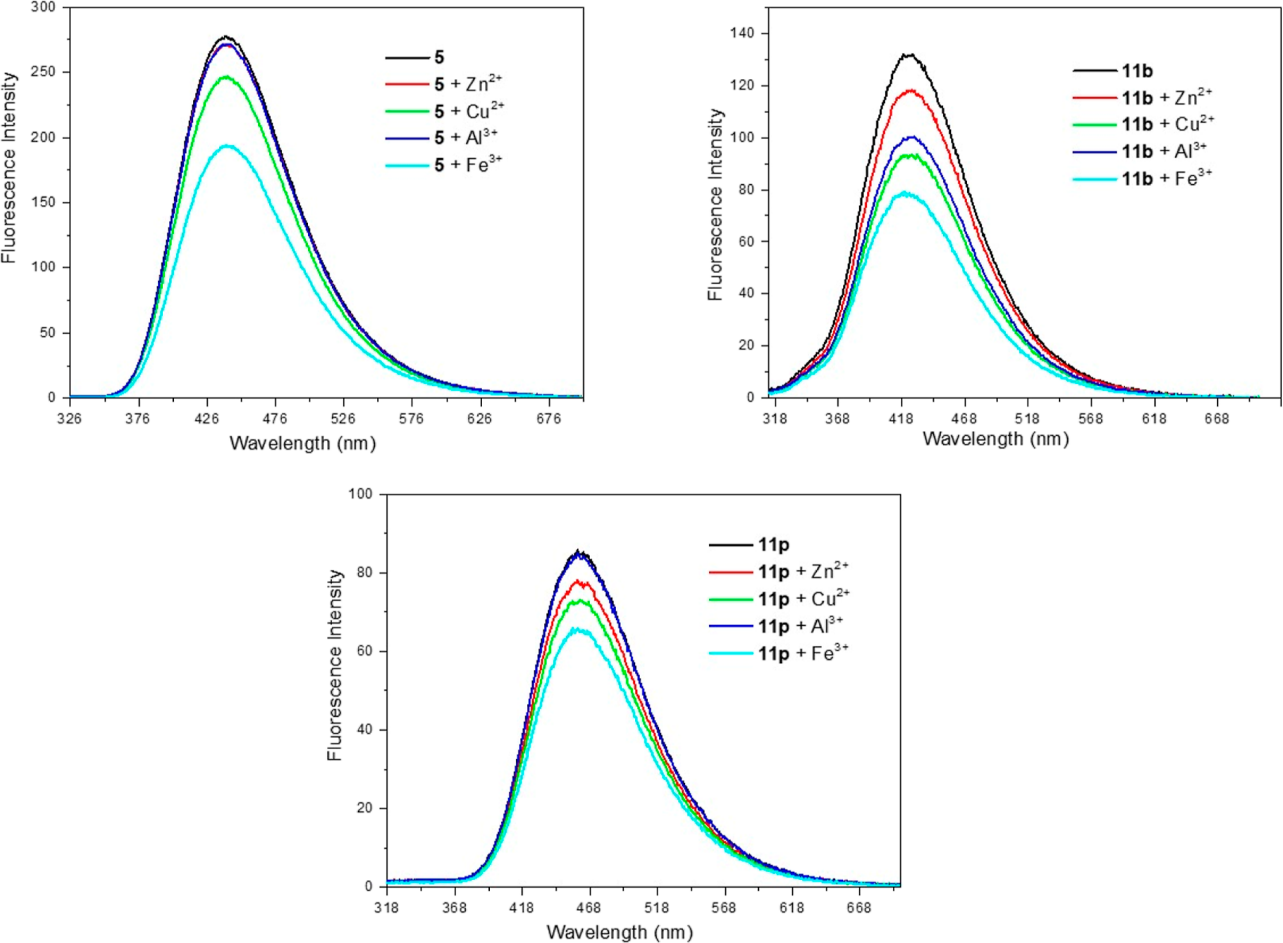
Emission spectrum of the **5**, **11b** and **11p** before and after addition of Cu^2+^, Zn^2+^, Al^3+^ and Fe^3+^.

Compounds **11b** and **11p** were excited at 279 nm in the absence and presence of metals, and thus the fluorescence emission spectra were obtained (Figure 5). The spectroscopic results for compound **11b** demonstrated that excitation caused a decrease in emission intensity in the presence of the four metal ions Zn^2+^, Cu^2+^, Al^3+^ and Fe^3+^, indicating the formation of 1,2,4-triazole complexes with all the metals tested. For compound **11p** a decrease in emission intensity was also observed in the presence of Zn^2+^, Cu^2+^, Fe^3+^ ions. However, the emission spectrum of the **11p** derivative was not altered by the presence of the Al^3+^ ion, which indicates that this 1,2,4-triazole does not complex Al^3+^. All these results indicate that 3-amino-1,2,4-triazole compounds are able to complex with Cu^2+^ and Fe^3+^ ions, while specific substitutions on the 1,2,4-triazole nucleus can broaden the complexation profile to other metals, as demonstrated for compounds **11b** and **11p**.

### 2.6 *Saccharomyces cerevisiae* Assay

Based on the results described above, we selected compounds **11b**, **11c**, **11m**, and **11p** to evaluate their toxicity and antioxidant profiles in the eukaryotic model *Saccharomyces cerevisiae* cells (strains BY4741 and Δ*gsh1*). Compounds **11b** and **11c** were selected because both are phenolic compounds with potential radical scavenging antioxidant profiles; **11m** due to the best AChE inhibition potency with substitution at position 1 of the triazole ring, which seems to be beneficial for AChE selective inhibition; and **11p** because of its ability to complex metals that can be evolved with oxidative stress.^32^

The eukaryotic preliminary toxicity of compounds was evaluated in both *S. cerevisiae* cell strains (BY4741 and Δ*gsh1*) after 24h incubation at compound concentrations range from 1000 to 0.4875 μM through resazurin viability assay (Table S1, Supporting Information). The results showed that all compounds are nontoxic at concentrations up to 250 μM and concentration of 20 μM was selected for subsequent assays. The growth curves of both strains (BY4741 and Δ*gsh1*) confirmed the results seen in the resazurin assay, i.e. any 1,2,4-triazole derivative at a concentration of 20 μM showed toxicity to yeast cells (Figure 6). These results were confirmed by the proximity in the values of cell concentrations throughout the experiments compared to the control samples that contained only *S. cerevisiae* cells (BY4741 control: 3.77 ± 0.25 mg/mL; **11b**: 3.61 ± 0.14 mg/mL; **11c**: 3.67 ± 0.22 mg/mL; **11m**: 3.75 ± 0.20 mg/mL and **11p**: 3.63 ± 0.07 mg/mL; Δ*gsh1* control: 3.84 ± 0.34 mg/mL; **11b**: 3.81 ± 0.17 mg/mL; **11c**: 3.93 ± 0.30 mg/mL; **11m**: 3.70 ± 0.19 mg/mL and **11p**: 3.77 ± 0.19 mg/mL) (Figure 6).

**Figure 6:**
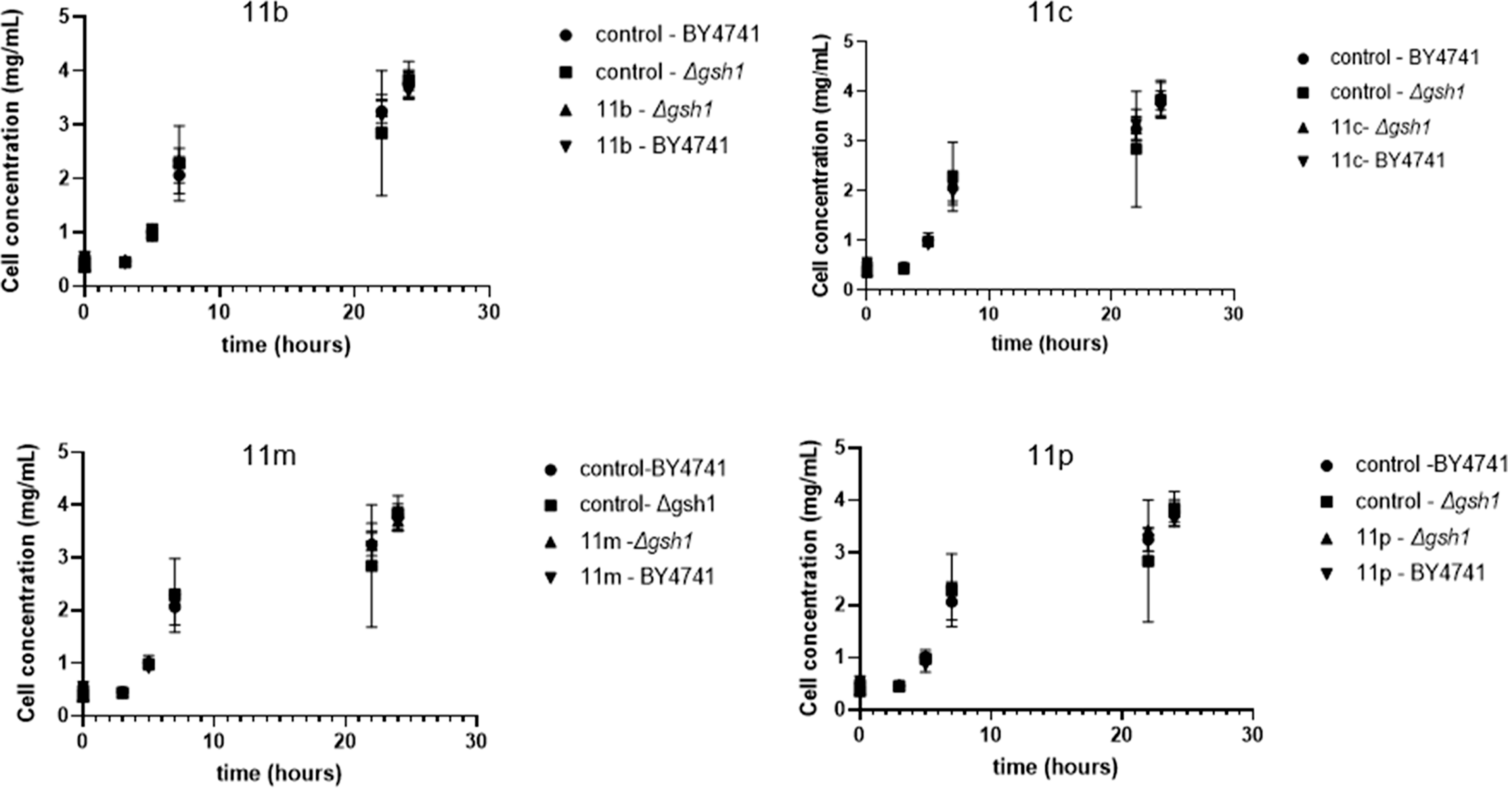
Growth curves using strains BY4741 and Δ*gsh1* after 24 h. All compounds were tested at 20 μM.

Quantification of lipid peroxidation in BY4741 strains (Figure 7) showed that treatment with all evaluated compounds significantly reduced lipid peroxidation levels, with values statistically comparable to those of the control cells. The derivatives **11m** and **11p** showed reductions of around 34% and 40% respectively, compared to stressed cells (hydrogen peroxide). The derivatives **11c** and **11b** showed similar reductions around 30%. These results also showed that all tested 1,2,4-triazole compounds were potentially antioxidant, protecting yeast cell membrane lipids against the formation of lipid peroxidation products. However, in Δ*gsh1* (a *GSH*-deficient strain) **11m** and **11p** were not efficient in reducing lipid peroxidation presented statistically similar results to cells treated only with hydrogen peroxide. Additionally, treatments performed with the phenolic compounds **11c** and **11b** were effective in reducing lipid peroxidation with reductions of around 22% and 24%, respectively (Figure 7).

**Figure 7:**
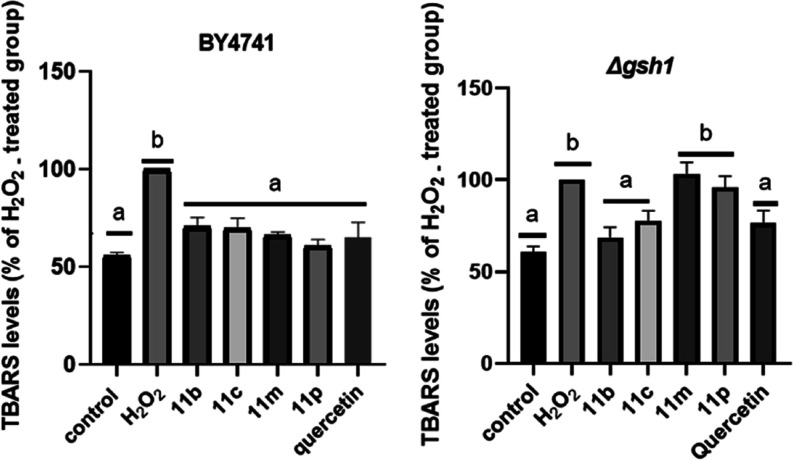
Quantification of lipid peroxidation of **11b**, **11c**, **11m** and **11p** in BY4741 and Δ*gsh1* strains in fermentative metabolism.

The results for the evaluation of intracellular oxidation levels (Figure 8) showed that there was a significant reduction in the levels of oxidant species in the intracellular environment for the BY4741 strain for **11b**, **11m** and **11p** with reductions of 14%, 18% and 21%, respectively. On the other hand, in the test performed on the Δ*gsh1* strain, all compounds were effective in reducing intracellular oxidation levels, showing statistical differences compared to the treatment with hydrogen peroxide. Among the compounds used, **11m** stands out with a reduction of approximately 27%, followed by **11c** with 20% and the compounds **11b** and **11p** with 18% each.

**Figure 8:**
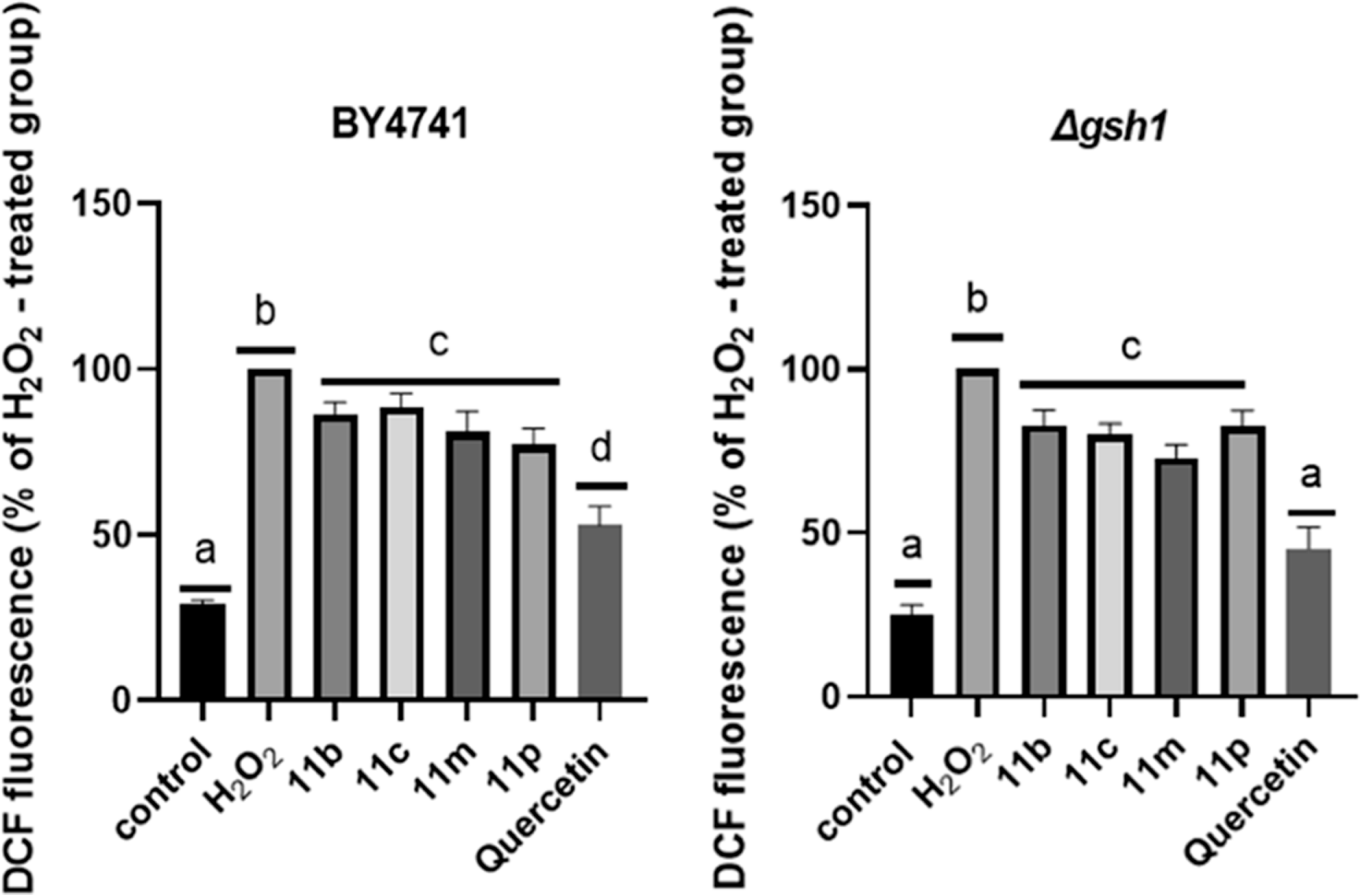
Evaluation of intracellular oxidation levels of **11b**, **11c**, **11m** and **11p** in BY4741 and Δ*gsh1* strains in fermentative metabolism.

## 3 Conclusion

In this work we could optimize and understand the influence of electron-withdrawing groups in increasing the inhibitory potency of 1,5-disubstituted-3-amino-1,2,4-triazoles against AChE, and the importance of substitution in R_1_ for the selectivity over BChE. The three best compounds were the nitro-substituted 1,2,4-triazoles **11m** (R_1_ = 3-NO_2_-Ph, R_2_ = Ph, AChE IC_50_ = 0.41 μM, BChE IC_50_ = 6.48 μM, SI = 15.74), **11n** (R_1_ = 4-NO_2_-Ph, R_2_ = Ph, AChE IC_50_ = 0.55 μM, BChE IC_50_ = 12.45 μM, SI = 22.43), and **11o** (R_1_ = Ph, R_2_ = 4-NO_2_-Ph, AChE IC_50_ = 0.38 μM, BChE IC_50_ = 2.07 μM, SI = 5.36). Qualitative molecular dynamics indicated a possible explanation for this effect. Interactions at the PAS and CAS appear to occur synergistically, with positive cooperativity, where PAS interactions (best performed by electron-withdrawing groups) seem to guide efficient CAS interactions, mainly with residue Trp86 in AChE and Trp107 in BChE. The additional metal-complexing abilities of the compounds, together with their protective antioxidant effects in *S. cerevisiae* (marked by a significant reduction in lipid peroxidation) highlight 1,5-disubstituted-3-amino-1,2,4-triazoles as promising multitarget-directed ligands with strong potential for further investigation in Alzheimer’s disease models.

## 4 Methods and Materials

### 4.1 Chemistry

All reagents and solvents were obtained from commercial sources and used as received, without additional purification. Reaction progress was monitored by thin-layer chromatography (TLC) using 0.25 mm Merck 60 F_254_ silica gel plates containing a fluorescent UV254 indicator. TLC plates were visualized under ultraviolet light at 254 and 365 nm.

^1^H and ^13^C NMR spectra were acquired on Bruker Avance spectrometers operating at 400 or 500 MHz (AC-400 or AC-500), using DMSO-*d*_6_ or CDCl_3_ as solvents and tetramethylsilane (TMS) as the internal reference. Signal multiplicities were assigned as s (singlet), d (doublet), t (triplet), q (quartet), dd (double doublet), and m (multiplet). Flash column chromatography was carried out on an automated Accelerated Chromatographic Isolation system (Biotage Isolera, model ISO-4SV). High-performance liquid chromatography (HPLC) analysis was carried out using a Prominence-Shimadzu with a loop of 10 μL and column Phenomenex C18 (100 mm × 4.6 mm × 3 μm) in MeOH (70%)/20% H_2_O (0.1% HCO_2_H). The melting points were recorded with a Fisatom model 431D fusiometer and are uncorrected. UV–vis spectra were obtained on Shimadzu UV-2450 spectrophotometer. Photoluminescence spectra were obtained on Edinburgh FS5 spectrofluorometer. High-resolution mass spectrometric analyses were performed on a Q Exactive hybrid quadrupole-Orbitrap mass spectrometer (Thermo Fisher Scientific, Bremen, Germany). Further characterization data for synthetic compounds are available in the Supporting Information.

#### 4.1.1 Synthesis of *S*-Methylthiouronium Iodide (**13**)

Methyl iodide (26.27 mmol) was added in a sealed tube (to prevent evaporation of methyl iodide) containing thiourea (**12**) (13.13 mmol) in methanol (10 mL) and, the mixture was stirred at 65 °C for 24 h. Evaporation of solvent on a rotary evaporator afforded desired product in 90% yield.

#### 4.1.2 Synthesis of *tert*-Butyl (Amino­(methylthio)­methylene)­carbamate (**14**)

A solution containing **13** (13.74 mmol), dichloromethane (30 mL), and triethylamine (13.74 mmol) was placed in a bottom flask and cooled in an ice bath. Subsequently, a solution of di-*tert*-butyl dicarbonate (Boc_2_O) (6.88 mmol) in dichloromethane (10 mL) was gradually added. The ice bath was then removed, and the reaction mixture was allowed to stir overnight at room temperature. The mixture was subsequently diluted with dichloromethane (15 mL), washed with water (2 × 10 mL), dried over anhydrous Na_2_SO_4_, and concentrated under reduced pressure using a rotary evaporator. Product was obtained as a white solid (85% yield) and used without further purification.

#### 4.1.3 General Procedure for Synthesis of *N*-Acyl-2-methyl-isothiourea-*N*-Boc Compounds (**15a–r**)

A solution of the appropriate acid chlorides (1 mmol) in dichloromethane (5 mL) was added dropwise to another solution containing **9** (1 mmol) in dichloromethane (10 mL) and pyridine (2.4 mmol). The reaction mixture was stirred overnight. Then, mixture was diluted with dichloromethane (20 mL) and washed with aqueous sodium bicarbonate 10% (3 × 10 mL). Organic phase was dried over anhydrous Na_2_SO_4_ and concentrated under reduced pressure using a rotary evaporator to give the respective products, which were purified by silica gel column chromatography (*n*-hexane/ethyl acetate gradient) (36–70% yield).

#### 4.1.4 General Procedure for Synthesis of 1,2,4-Triazole-*N*-Boc Compounds (**16a–t**)

In a sealed borosilicate tube containing **15a–t** (1 mmol) in 3 mL of acetonitrile, was added the corresponding phenylhydrazine (R_2_ = Ph, 4-F-Ph and 4-NO_2_-Ph) (1.1 mmol) or phenylhydrazine·2HCl (R_2_ = Bn) (1.1 mmol) in triethylamine (9 mmol). Reaction was stirred at 100 °C for 1 h in microwave reactor. Upon completion of the reaction, the crude mixture was partitioned between ethyl acetate (20 mL) and water (10 mL). The organic layer was dried over anhydrous Na_2_SO_4_ and concentrated under reduced pressure using a rotary evaporator. The resulting products were purified by flash chromatography using a Biotage system (*n*-hexane/ethyl acetate gradient elution) (48–87% yield).

#### 4.1.5 General Procedure for Synthesis of *N*-Alkyl-1,2,4-triazole-*N*-Boc Compounds (**17a–t**)

In a sealed borosilicate tube containing triazole **16a–t** (1 mmol) and sodium hydride (3 mmol) in anhydrous DMF (7 mL) was added 1,3-dibromopropane (3 mmol). The reaction was stirred at 50 °C for 1 h under inert atmosphere. Reaction completion was monitored by TLC using a *n*-hexane/ethyl acetate (70:30) solvent system. The reaction mixture was then diluted with ethyl acetate (30 mL) and washed with water (3 × 10 mL). The organic layer was dried over anhydrous Na_2_SO_4_, concentrated under reduced pressure, and purified by flash chromatography using a Biotage system (*n*-hexane/ethyl acetate gradient elution) (37–79% yield).

#### 4.1.6 General Procedure for Synthesis of *N*-Propyl-benzylpiperazinyl-1,2,4-triazole-*N*-Boc Compounds (**18a–t**)

Benzylpiperazine (1 mmol) was added in a sealed borosilicate tube containing **17a–t** (0.5 mmol) and acetonitrile (10 mL). Reaction was stirred at 60 °C for 12 h. Then, mixture was concentrated and purified with FLASH Chromatography System (Biotage) (DCM/methanol gradient elution) (35–66% yield).

#### 4.1.7 General Procedure for Synthesis of *N*-Propyl-benzylpiperazinyl-1,2,4-triazole-3-Amine Compounds (**11a–t**)

In a sealed borosilicate tube containing triazoles **18a–t** (0.17 mmol) in dichloromethane (18 mL), in an ice-bath, it was added trifuoracetic acid (6 mmol). The ice bath was removed, and the reaction mixture was allowed to stir overnight at room temperature. Reaction completion was monitored by TLC, after which a saturated sodium bicarbonate solution was added until neutral pH was reached. The mixture was then extracted with dichloromethane, dried over anhydrous Na_2_SO_4_, and concentrated under reduced pressure using a rotary evaporator. For those compounds presenting impurities, it was performed a purification with FLASH Chromatography System (Biotage) (DCM/methanol gradient elution).

##### 4.1.7.1 *N*-(3-(4-Benzylpiperazin-1-yl)­propyl)-1-phenyl-5-(*o*-tolyl)-1*H*-1,2,4-triazol-3-amine (**11a**)

White solid. Yield = 95%. mp = 129–132 °C. ^1^H NMR (500 MHz, CDCl_3_) (δ ppm): 7.32–7.21 (m, 14H); 3.54 (s, 2H); 3.46 (t, 2H); 2.56 (m, 10H); 1.88 (m, 2H). ^13^C NMR (125 MHz, CDCl_3_) (δ ppm): 164.06; 152.77; 138.02; 137.28; 130.55; 130.01; 129.92; 128.20; 127.07; 125.92; 125.25; 122.97; 62.91; 62.21; 56.78; 52.87; 26.30; 19.65. HPLC purity: 97.9%. HRMS (ESI) *m*/*z*: [M + H]^+^ calcd = 467.2918; found = 467.2915.

##### 4.1.7.2 2-(3-((3-(4-Benzylpiperazin-1-yl)­propyl)­amino)-1-phenyl-1*H*-1,2,4-triazol-5-yl)­phenol (**11b**)

Brown oil. Yield = 55%. ^1^H NMR (500 MHz, CDCl_3_) (δ ppm): 7.51–7.31 (m, 11H); 7.24 (t, 1H); 7.05 (d, 1H); 6.82 (d, 1H); 6.55 (t, 1H); 5.28 (sl, 1H); 3.54 (s, 2H); 3.43 (t, 2H); 2.55 (m, 10H); 1.85 (m, 2H). ^13^C NMR (125 MHz, CDCl_3_) (δ ppm): 161.52; 150.25; 138.68; 137.73; 131.49; 129.45; 129.07; 127.95; 126.35; 126.32; 118.14; 117.63; 110.63; 62.72; 56.80; 52.79; 42.50; 25.61. HPLC purity: 95.9%. HRMS (ESI) *m*/*z*: [M + H]^+^ calcd = 469.2710; found = 469.2708.

##### 4.1.7.3 4-(3-((3-(4-Benzylpiperazin-1-yl)­propyl)­amino)-1-phenyl-1*H*-1,2,4-triazol-5-yl)­phenol (**11c**)

White solid. Yield = 84%. mp = 154–156 °C. ^1^H NMR (500 MHz, CDCl_3_) (δ ppm): 9.89 (s, 1H); 7.42 (m, 3H); 7.29 (m, 7H); 7.21 (d, 2H); 6.72 (d, 2H); 3.44 (s, 2H); 3.14 (t, 2H); 2.34 (m, 10H); 1.69 (m, 2H). ^13^C NMR (125 MHz, CDCl_3_) (δ ppm): 163.81; 158.63; 152.21; 138.26; 138.59; 129.91; 128.17; 128.10; 127.77; 126.83; 125.09; 118.89; 115.22; 62.09; 55.72; 52.66; 41.33; 26.38. HPLC purity: 95.8%. HRMS (ESI) *m*/*z*: [M + H]^+^ calcd = 469.2710; found = 469.2709.

##### 4.1.7.4 *N*-(3-(4-Benzylpiperazin-1-yl)­propyl)-5-(2-methoxyphenyl)-1-phenyl-1*H*-1,2,4-triazol-3-amine (**11d**)

Brown oil. Yield = 89%. ^1^H NMR (500 MHz, CDCl_3_) (δ ppm): 7.65 (m, 2H); 7.32–7.21 (m, 11H); 7.13 (d, 1H); 7.06 (d, 1H); 3.77 (s, 3H); 3.51 (t, 2H); 3.50 (s, 2H); 2.42 (m, 10H); 2.04 (m, 2H). ^13^C NMR (125 MHz, CDCl_3_) (δ ppm): 157.54; 152.50; 135.35; 131.92; 129.11; 128.83; 128.29; 128.16; 127.22; 121.97; 111.75; 62.97; 55.94; 54.29; 44.47; 42.68; 29.71. HPLC purity: 97.2%. HRMS (ESI) *m*/*z*: [M + H]^+^ calcd = 483.2867; found = 483.2866.

##### 4.1.7.5 *N*-(3-(4-Benzylpiperazin-1-yl)­propyl)-5-(4-methoxyphenyl)-1-phenyl-1*H*-1,2,4-triazol-3-amine (**11e**)

Brown oil. Yield = 91%. ^1^H NMR (500 MHz, CDCl_3_) (δ ppm): 7.38–7.29 (m, 12H); 6.83 (d, 2H); 3.81 (s, 3H); 3.67 (sl, 2H); 3.44 (sl, 2H); 2.92–2.80 (m, 10H); 2.02 (m, 2H). ^13^C NMR (125 MHz, CDCl_3_) (δ ppm): 163.64; 160.72; 152.80; 138.43; 130.21; 129.48; 129.24; 128.56; 128.13; 125.28; 120.30; 113.89; 61.91; 55.53; 55.27; 50.64; 41.49; 24.98. HPLC purity: 99.7%. HRMS (ESI) *m*/*z*: [M + H]^+^ calcd = 483.2867; found = 483.2864.

##### 4.1.7.6 *N*-(3-(4-Benzylpiperazin-1-yl)­propyl)-1-phenyl-5-(3,4,5-trimethoxyphenyl)-1*H*-1,2,4-triazol-3-amine (**11f**)

Brown oil. Yield = 93%. ^1^H NMR (500 MHz, CDCl_3_) (δ ppm): 7.43–7.30 (m, 11H); 6.64 (s, 2H); 3.65 (s, 2H); 3.45 (t, 2H); 2.94–2.72 (m, 10H); 2.03 (m, 2H). ^13^C NMR (125 MHz, CDCl_3_) (δ ppm): 163.59; 153.01; 152.69; 139.30; 138.48; 129.33; 128.61; 128.48; 125.77; 106.06; 61.92; 60.93; 55.92; 55.48; 50.52; 41.42; 29.70. HPLC purity: 97.8%. HRMS (ESI) *m*/*z*: [M + H]^+^ calcd = 543.3078; found = 543.3078.

##### 4.1.7.7 *N*-(3-(4-Benzylpiperazin-1-yl)­propyl)-5-(4-fluorophenyl)-1-phenyl-1*H*-1,2,4-triazol-3-amine (**11g**)

Beige solid. Yield = 90%. mp = 57–59 °C. ^1^H NMR (500 MHz, CDCl_3_) (δ ppm): 7.45–7.28 (m, 12H); 7.07 (t, 2H); 3.56 (s, 2H); 3.43 (t, 2H); 2.58 (m, 9H); 1.88 (m, 2H). ^13^C NMR (125 MHz, CDCl_3_) (δ ppm): 164.10; 162.94; 160.96; 152.95; 129.92; 129.34; 128.75; 128.58; 128.28; 127.22; 127.19; 127.15; 116.33; 116.15; 62.90; 56.56; 53.07; 52.74; 42.55; 29.73. ^19^F NMR (470 MHz, CDCl_3_) (δ ppm): −112.72. HPLC purity: 97.9%. HRMS (ESI) *m*/*z*: [M + H]^+^ calcd = 471.2667; found = 471.2667.

##### 4.1.7.8 *N*-(3-(4-Benzylpiperazin-1-yl)­propyl)-5-(2-chlorophenyl)-1-phenyl-1*H*-1,2,4-triazol-3-amine (**11h**)

Beige solid. Yield = 90%. mp = 77–79 °C. ^1^H NMR (500 MHz, CDCl_3_) (δ ppm): 7.53 (d, 2H); 7.33 (m, 9H); 7.23 (m, 3H); 4.85 (s, 11H); 3.54 (s, 2H); 3.40 (t, 2H); 2.53 (m, 10H); 1.84 (m, 2H). ^13^C NMR (125 MHz, CDCl_3_) (δ ppm): 163.94; 151.07; 137.80; 134.22; 129.66; 129.64; 129.39; 129.28; 129.83; 128.89; 128.46; 128.23; 127.07; 126.80; 125.33; 62.69; 56.44; 52.70; 42.46; 26.03. HPLC purity: 99.5%. HRMS (ESI) *m*/*z*: [M + H]^+^ calcd = 487.2371; found = 483.2372.

##### 4.1.7.9 *N*-(3-(4-Benzylpiperazin-1-yl)­propyl)-5-(3-chlorophenyl)-1-phenyl-1*H*-1,2,4-triazol-3-amine (**11i**)

Beige solid. Yield = 93%. mp = 147–149 °C. ^1^H NMR (500 MHz, CDCl_3_) (δ ppm): 7.48 (s, 1H); 7.36 (m, 8H); 4.87 (sl, 1H); 3.57 (s, 2H); 3.49 (t, 2H); 2.58 (m, 10H); 1.91 (m, 2H). ^13^C NMR (125 MHz, CDCl_3_) (δ ppm): 163.93; 150.09; 137.67; 133.53; 131.44; 131.05; 129.75; 128.98; 128.69; 128.63; 127.86; 127.10; 126.78; 126.69; 122.86; 62.67; 56.54; 52.67; 42.55; 26.02. HPLC purity: 99.2%. HRMS (ESI) *m*/*z*: [M + H]^+^ calcd = 487.2371; found = 483.2371.

##### 4.1.7.10 *N*-(3-(4-Benzylpiperazin-1-yl)­propyl)-5-(4-chlorophenyl)-1-phenyl-1*H*-1,2,4-triazol-3-amine (**11j**)

Beige solid. Yield = 96%. mp = 93–95 °C. ^1^H NMR (500 MHz, CDCl_3_) (δ ppm): 7.49–7.31 (m, 10H); 7.25 (d, 2H); 6.93 (d, 2H); 3.53 (sl, 2H); 3.40 (t, 2H); 2.50 (m, 10H); 1.85 (m, 2H). ^13^C NMR (125 MHz, CDCl_3_) (δ ppm): 164.06; 148.04; 138.13; 129.70; 129.51; 129.29; 129.26; 128.38; 128.21; 127.03; 126.76; 126.76; 63.05; 56.65; 53.07; 42.65; 26.49. HPLC purity: 97.1%. HRMS (ESI) *m*/*z*: [M + H]^+^ calcd = 487.2371; found = 483.2372.

##### 4.1.7.11 *N*-(3-(4-Benzylpiperazin-1-yl)­propyl)-5-(3-bromophenyl)-1-phenyl-1*H*-1,2,4-triazol-3-amine (**11k**)

Brown oil. Yield = 89%. ^1^H NMR (500 MHz, CDCl_3_) (δ ppm): 7.71 (s, 1H); 7.52 (d, 1H); 7.40 (m, 3H); 7.34 (m, 8H); 7.16 (t, 1H); 4.80 (sl, 1H); 3.57 (s, 2H); 3.44 (t, 2H); 2.58 (m, 10H); 1.88 (m, 2H). ^13^C NMR (125 MHz, CDCl_3_) (δ ppm): 164.15; 151.20; 138.02; 132.71; 131.71; 130.08; 129.83; 129.34; 129.26; 128.45; 128.22; 127.17; 127.12; 125.28; 122.51; 62.84; 56.56; 52.71; 42.16; 26.16. HPLC purity: 98.6%. HRMS (ESI) *m*/*z*: [M + H]^+^ calcd = 531.1866; found = 531.1868.

##### 4.1.7.12 *N*-(3-(4-Benzylpiperazin-1-yl)­propyl)-5-(4-bromophenyl)-1-phenyl-1*H*-1,2,4-triazol-3-amine (**11l**)

Brown solid. Yield = 90%. mp = 102–104 °C. ^1^H NMR (500 MHz, CDCl_3_) (δ ppm): 7.44 (d, 2H); 7.33 (m, 3H); 4.81 (s, 1H); 3.59 (s, 2H); 3.44 (t, 2H); 2.70 (m, 12H). ^13^C NMR (125 MHz, CDCl_3_) (δ ppm): 164.04; 151.78; 138.12; 131.71; 130.22; 129.38; 129.35; 128.42; 128.38; 127.41; 127.01; 125.30; 124.28; 62.52; 56.18; 49.87; 42.12; 25.68. HPLC purity: 96.8%. HRMS (ESI) *m*/*z*: [M + H]^+^ calcd = 531.1866; found = 531.1867.

##### 4.1.7.13 *N*-(3-(4-Benzylpiperazin-1-yl)­propyl)-5-(3-nitrophenyl)-1-phenyl-1*H*-1,2,4-triazol-3-amine (**11m**)

Brown oil. Yield = 93%. ^1^H NMR (500 MHz, CDCl_3_) (δ ppm): 8.36 (s, 1H); 8.22 (d, 1H); 7.74 (d, 1H); 7.49 (t, 1H); 7.73–7.30 (m, 11H); 3.55 (sl, 2H); 3.44 (t, 2H); 2.57 (m, 10H); 1.87 (m, 2H). ^13^C NMR (125 MHz, CDCl_3_) (δ ppm): 164.38; 150.38; 148.17; 137.68; 134.29; 129.86; 129.67; 129.55; 129.30; 129.00; 128.26; 127.50; 125.50; 124.29; 62.90; 56.68; 52.81; 42.73; 26.14. HPLC purity: 95.4%. HRMS (ESI) *m*/*z*: [M + H]^+^ calcd = 498.2612; found = 498.2610.

##### 4.1.7.14 *N*-(3-(4-Benzylpiperazin-1-yl)­propyl)-5-(4-nitrophenyl)-1-phenyl-1*H*-1,2,4-triazol-3-amine (**11n**)

Yellow solid. Yield = 95%. mp = 124–126 °C. ^1^H NMR (500 MHz, CDCl_3_) (δ ppm): 8.17 (d, 2H); 7.64 (d, 2H); 7.42 (m, 3H); 7.32 (m, 7H); 3.56 (s, 2H); 3.44 (t, 2H); 2.51 (m, 10H); 1.88 (m, 2H). ^13^C NMR (125 MHz, CDCl_3_) (δ ppm): 164.39; 150.47; 148.13; 137.86; 134.06; 129.62; 129.57; 129.28; 128.96; 128.26; 127.20; 125.46; 123.66; 62.86; 56.53; 52.98; 42.57; 25.99. HPLC purity: 98.3%. HRMS (ESI) *m*/*z*: [M + H]^+^ calcd = 498.2612; found = 498.2612.

##### 4.1.7.15 *N*-(3-(4-Benzylpiperazin-1-yl)­propyl)-1-(4-nitrophenyl)-5-phenyl-1*H*-1,2,4-triazol-3-amine (**11o**)

Yellow solid. Yield = 96%. mp = 173–176 °C. ^1^H NMR (500 MHz, CDCl_3_) (δ ppm): 8.22 (d, 2H); 7.49–7.42 (m, 12H); 4.09 (s, 2H); 3.55–3.44 (m, 19H); 3.19 (m, 10H); 1.96 (m, 2H). ^13^C NMR (125 MHz, CDCl_3_) (δ ppm): 162.90; 153.39; 146.49; 142.51; 130.63; 130.11; 129.42; 129.17; 128.89; 126.67; 124.75; 124.63; 61.09; 55.04; 49.19; 40.34; 23.89. HPLC purity: 98.6%. HRMS (ESI) *m*/*z*: [M + H]^+^ calcd = 498.2612; found = 498.2613.

##### 4.1.7.16 *N*-(3-(4-Benzylpiperazin-1-yl)­propyl)-1-phenyl-5-(pyridin-2-yl)-1*H*-1,2,4-triazol-3-amine (**11p**)

Brown oil. Yield = 93%. ^1^H NMR (500 MHz, CDCl_3_) (δ ppm): 8.50 (d, 1H); 7.69 (m, 2H); 7.32 (m, 11H); 3.60 (s, 2H); 3.46 (t, 2H); 2.70 (m, 10H); 1.94 (m, 2H). ^13^C NMR (125 MHz, CDCl_3_) (δ ppm): 163.93; 151.33; 149.44; 147.49; 138.71; 136.56; 129.36; 128.85; 128.35; 128.05; 127.44; 125.31; 124.14; 124.10; 62.47; 56.11; 42.16; 29.67; 25.71; 42.16. HPLC purity: 96.5%. HRMS (ESI) *m*/*z*: [M + H]^+^ calcd = 454.2714; found = 454.2715.

##### 4.1.7.17 *N*-(3-(4-Benzylpiperazin-1-yl)­propyl)-5-(furan-2-yl)-1-phenyl-1*H*-1,2,4-triazol-3-amine (**11q**)

Brown oil. Yield = 91%. ^1^H NMR (500 MHz, CDCl_3_) (δ ppm): 7.49–7.27 (m, 12H); 6.38 (sl, 1H); 3.76 (sl, 2H); 3.44 (t, 2H); 3.04 (m, 10H); 2.85 (m, 10H); 2.06 (m, 2H). ^13^C NMR (125 MHz, CDCl_3_) (δ ppm): 163.65; 144.81; 144.12; 142.20; 137.89; 129.71; 129.29; 129.21; 128.76; 126.06; 112.61; 111.54; 61.49; 59.50; 55.16; 49.77; 41.06; 29.67. HPLC purity: 99.1%. HRMS (ESI) *m*/*z*: [M + H]^+^ calcd = 443.2554; found = 443.2553.

##### 4.1.7.18 *N*-(3-(4-Benzylpiperazin-1-yl)­propyl)-1-phenyl-5-(thiophen-2-yl)-1*H*-1,2,4-triazol-3-amine (**11r**)

Yellow solid. Yield = 91%. mp = 93–95 °C. ^1^H NMR (500 MHz, CDCl_3_) (δ ppm): 7.48 (m, 14H); 3.53 (s, 2H); 3.38 (t, 2H); 2.51 (m, 10H); 1.83 (m, 2H). ^13^C NMR (125 MHz, CDCl_3_) (δ ppm): 163.75; 157.69; 153.03; 136.05; 135.87; 129.58; 129.53; 128.97; 128.78; 127.93; 127.79; 127.55; 126.79; 126.25; 62.67; 56.86; 51.92; 42.42; 26.07. HPLC purity: 97.5%. HRMS (ESI) *m*/*z*: [M + H]^+^ calcd = 459.2325; found = 459.2322.

##### 4.1.7.19 *N*-(3-(4-Benzylpiperazin-2-yl)­propyl)-5-(naphthalen-1-yl)-1-phenyl-1*H*-1,2,4-triazol-3-amine (**11s**)

Brown oil. Yield = 86%. ^1^H NMR (500 MHz, CDCl_3_) (δ ppm): 8.03 (m, 17H); 3.53 (sl, 4H); 2.56 (m, 10H); 1.90 (sl, 2H). ^13^C NMR (125 MHz, CDCl_3_) (δ ppm): 164.03; 151.58; 137.80; 137.65; 133.32; 131.15; 130.07; 128.98; 128.58; 128.03; 127.92; 126.91; 126.88; 126.78; 126.18; 125.13; 124.63; 124.63; 123.21; 123.21; 62.70; 56.57; 53.00; 52.74; 42.58; 26.15. HPLC purity: 95.5%. HRMS (ESI) *m*/*z*: [M + H]^+^ calcd = 503.2918; found = 503.2917.

##### 4.1.7.20 1-Benzyl-*N*-(3-(4-benzylpiperazin-1-yl)­propyl)-5-phenyl-1*H*-1,2,4-triazol-3-amine (**11t**)

Brown oil. Yield = 92%. ^1^H NMR (500 MHz, CDCl_3_) (δ ppm): 7.46 (m, 2H); 7.40 (m, 2H); 7.32 (d, 9H); 7.18 (m, 2H); 5.23 (s, 2H); 3.53 (s, 2H); 3.38 (t, 2H); 2.51 (m, 10H); 1.83 (m, 2H). ^13^C NMR (125 MHz, CDCl_3_) (δ ppm): 163.27; 154.22; 136.09; 129.88; 128.57; 128.52; 128.24; 127.54; 126.44; 61.76; 59.56; 55.38; 52.17; 41.36; 29.71. HPLC purity: 99.1%. HRMS (ESI) *m*/*z*: [M + H]^+^ calcd = 467.2918; found = 467.2918.

##### 4.1.7.21 1-(1-Benzylpiperidin-4-yl)-5-phenyl-1*H*-1,2,4-triazol-3-amine (**21**)

Beige solid. Yield = 90%. mp = 76–78 °C. ^1^H NMR (500 MHz, CDCl_3_) (δ ppm): 7.50 (m, 5H); 7.33 (m, 5H); 4.21 (sl, 2H); 3.54 (s, 2H); 2.99 (sl, 2H); 2.34 (sl, 2H); 2.05 (m, 2H); 1.83 (sl, 2H). ^13^C NMR (125 MHz, CDCl_3_) (δ ppm): 161.70; 152.98; 129.65; 128.66; 128.60; 128.35; 128.04; 127.97; 126.83; 62.24; 52.06; 31.73. HRMS (ESI) *m*/*z*: [M + H]^+^ calcd = 334.2026; found = 334.2023.

### 4.2 Biological Assays

#### 4.2.1 Cholinesterase Inhibitory Activity^26^

The cholinesterase inhibitory activity was determined using an adapted Ellman’s method. All of the solutions were prepared in 0.02 M T–HCl buffer (pH = 7.5), stock solutions of test compounds prepared in DMSO (50 mM), and the experiment conducted in triplicate. A flat-bottom, transparent 96-well plate was loaded with 150 μL of treatment solutions containing inhibitors **11a**–**t**, **21**, and the reference donepezil (**9**), prepared at eight serially diluted concentrations. Untreated wells (negative control) and vehicle controls (DMSO; final concentrations of 0.2% v/v for AChE assays and 0.8% v/v for BChE assays) were included for comparison. Subsequently, 60 μL of 5,5′-dithiobis­(2-nitrobenzoic acid) (DTNB, Ellman’s reagent; 1.1 mM) and 30 μL of either electric eel acetylcholinesterase (eeAChE) or equine serum butyrylcholinesterase (eqBChE) solution (0.20 U/mL), prepared in the presence of bovine serum albumin (BSA, 1 mg/mL), were added to each well. Initial absorbance was measured at 415 nm using an iMark microplate reader (Bio-Rad) equipped with a λ = 415 nm light filter to establish the blank reference.

After incubation at 30 °C for 10 min, 24 μL of acetylthiocholine iodide (ACTI, 2.75 mM) or *S*-butyrylthiocholine iodide (BCTI) were added to initiate the enzymatic reaction. Absorbance at 415 nm was then recorded ten times over a 30 s period following a further 10 min incubation at 30 °C.

Enzymatic activity was expressed as a percentage relative to untreated control, after subtraction of the blank signal. Final inhibitor concentrations ranged from 100 to 0.00001 μM for AChE (10-fold serial dilution) and from 400 to 0.1 μM for BChE (4-fold serial dilution). IC_50_ values were determined using nonlinear regression analysis of dose–response curves in GraphPad Prism 7.0.

#### 4.2.2 Study of Enzymatic Kinetics^26^

Enzymatic kinetics was assessed using a modified Ellman’s assay. All solutions were prepared in 0.02 M Tris–HCl buffer (pH 7.5). Stock solutions of the tested compounds were prepared in DMSO at a concentration of 50 mM, and all experiments were performed in triplicate.

Aliquots of 150 μL of treatment solutions containing inhibitors **11o** and **11t** (Table 2) at two selected concentrations were dispensed into a transparent, flat-bottom 96-well plate, arranged in eight sets of triplicate wells. Eight additional sets of triplicates without inhibitors were included as negative controls.

Subsequently, 60 μL of 5,5′-dithiobis­(2-nitrobenzoic acid) (DTNB, Ellman’s reagent; 1.1 mM) and 30 μL of either electric eel acetylcholinesterase (eeAChE) or equine serum butyrylcholinesterase (eqBChE) solution (0.20 U/mL), prepared in the presence of bovine serum albumin (BSA, 1 mg/mL), were added to each well. Baseline absorbance was recorded at 415 nm using an iMark microplate reader (Bio-Rad) and used as the blank reference.

After a 10 min incubation at room temperature, 24 μL of acetylthiocholine iodide (ACTI) or *S*-butyrylthiocholine iodide (BCTI) were added at eight serially diluted concentrations (dilution factor = 1.3), ranging from 2.75 to 0.44 mM (final concentrations of 0.25–0.04 mM). Absorbance at 415 nm was then measured at 0, 5, 10, 15, and 20 min at room temperature.

Lineweaver–Burk double-reciprocal plots were constructed by plotting 1/velocity versus 1/[substrate] for each inhibitor concentration and the untreated control. The intersection patterns of the linear regressions were used to determine the inhibition mechanism. Kinetic parameters, including *K*_i_, *K*_i_^′^ (competitive and noncompetitive inhibition constants, respectively), *K*_m_ (Michaelis–Menten constant), and *V*_max_, were calculated using nonlinear regression models for enzyme inhibition and substrate–velocity kinetics in GraphPad Prism 7.0.

#### 4.2.3 *Saccharomyces cerevisiae* Assay

##### 4.2.3.1 Microorganisms and Culture Conditions

Two formulations of yeast–peptone–dextrose (YPD) medium were employed, YPD liquid (2%): 1% yeast extract (Difco, USA), 2% peptone (Difco, USA) and 2% glucose (Sigma) and YPD solid (2%): same composition as above, supplemented with 2% agar (Kasvi) for solidification. Two *S. cerevisiae* strains were used: BY4741 (wild type) and Δ*gsh1* (glutathione-deficient mutant). Strains were obtained from Euroscarf (Frankfurt, Germany; http://www.euroscarf.de/) and maintained on solid YPD (2%) at 4 °C. For Δ*gsh1*, 0.02% Geneticin was added to the storage medium to select for the deletion marker.

##### 4.2.3.2 Preparation of Test Compounds and Stock Solutions

Compounds **11c**, **11m** and **11p** were dissolved in DMSO/distilled water (1:1, v/v), whereas **11b** was dissolved in DMSO/distilled water (2:1, v/v). Stock solutions of each isomer (2000 μM) were prepared. Quercetin stock (5000 μM) was prepared in distilled water/DMSO (4:1, v/v). In all assays, the final DMSO concentration did not exceed 0.7%, below the reported toxic threshold for yeast.^33^

##### 4.2.3.3 Resazurin Assay

Flat-bottom 96-well plates were loaded with 50 μL YPD per well. Test compound (1000 μM) was added (50 μL) to the first well of each row, followed by 1:1 serial dilutions across the row (50 μL transfers). Then, 10 μL of yeast suspension was added to each well. After 24 h at 28 °C, 10 μL of 0.01% (w/v) resazurin solution was added. Plates were incubated for 30 min at room temperature: wells turning pink indicated metabolic activity, whereas violet or blue indicated loss of viability.

##### 4.2.3.4 Growth-Curve Analysis

Yeast cultures were adjusted to 0.1 mg/mL (wet weight) and inoculated (10 mL) into 100 mL Erlenmeyer flasks containing YPD supplemented with test compounds at 20 μM. Cultures were incubated at 28 °C, 160 rpm. Samples were withdrawn at 0, 3, 5, 7, 22, and 24 h for optical density or dry-weight measurements.

##### 4.2.3.5 Pretreatment with Test Compounds and H_2_O_2_ Exposure

After 22 h of growth, cells were harvested by centrifugation, washed twice with cold sterile distilled water, and resuspended in 0.5 M potassium phosphate buffer (pH 6.0). Test compounds were added to a final concentration of 20 μM and incubated for 2 h at 28 °C, 160 rpm. Cells were washed twice more, resuspended in fresh buffer, and exposed to H_2_O_2_ (Merck) at 1.0 mM or 2.0 mM for 1 h at 28 °C, 160 rpm. A no-H_2_O_2_ control was included.

##### 4.2.3.6 Lipid Peroxidation

Treated cells (50 mg wet weight) were pelleted (4000 rpm, 5 min) and resuspended in 0.5 mL of 10% trichloroacetic acid (TCA) in 0.5 M potassium phosphate buffer (pH 6.0). Samples were transferred to tubes containing 1.5 g glass beads and lysed by six cycles of vortexing (20 s) alternating with ice incubation (20 s). Lysates were pelleted (4000 rpm, 4 min), and 150 μL of supernatant was mixed with 150 μL H_2_O, 100 μL 0.1 M EDTA and 600 μL 1% thiobarbituric acid (TBA) in 0.05 M NaOH. A reaction blank (300 μL H_2_O only) was included. Samples were heated at 100 °C for 15 min, cooled on ice, and absorbance was read at 532 nm.^34^

##### 4.2.3.7 Intracellular Oxidation Assay

Aliquots containing 3 mg wet cells were treated with test compounds, washed twice, and resuspended in 1 mL 0.5 M potassium phosphate buffer (pH 7.8). Then, 20 μL of 20 μM H_2_DCFDA was added; after 15 min, 20 μL of 2.0 mM H_2_O_2_ was added. Following 1 h incubation at 28 °C, 200 rpm, cells were pelleted and supernatants were transferred to a 96-well plate. Fluorescence was measured (excitation/emission: 488/520 nm).^35^

##### 4.2.3.8 Statistical analysis

Results are presented as mean ± SD of at least three independent experiments. Statistical comparisons were performed in GraphPad Prism 8 by one-way ANOVA followed by Tukey’s post hoc test. Differences were considered significant when *p* < 0.05.

### 4.3 Docking and Molecular Dynamics Simulations

Molecular docking and molecular dynamics (MD) simulations were performed in *FlarePro+* using the OpenMM package (Cresset, Litlington, Cambridgeshire, UK; https://www.cresset-group.com/software/flare/). Docking was carried out with acetylcholinesterase (AChE, PDB ID: 1C2B) and butyrylcholinesterase (BChE), for which a homology model was generated using the Swiss-Model server based on the crystal structure of PDB ID: 4EY7. The docking grid was defined to encompass both the catalytic active site (CAS) and the peripheral anionic site (PAS). The docked complexes of compounds **5**, **11c**, **11n**, and **11t** with each enzyme were used as starting structures for MD simulations. Ligands were minimized using the OpenFF 2.2.0 force field, while proteins were parametrized with the AMBER force field. MD simulations were performed with default parameters, using the transferable intermolecular potential with three points (TIP3P) water model to represent explicit solvation, and AM1-BCC charges were applied to the ligands. Each system was equilibrated, followed by production runs of 50 ns, with trajectory frames saved at 1 ps intervals. Analyses included root-mean-square deviation (RMSD), root-mean-square fluctuation (RMSF), and monitoring of the time-dependent distance between the terminal benzyl group of the ligands and key aromatic residues at the PAS and CAS (Trp286/Trp86 in AChE and Phe307/Trp107 in BChE).

### 4.4 Study of the Complexation of Derivatives Against Cu^2+^, Zn^2+^, Al^3+^ and Fe^3+^

The compounds (**5**, **11b** and **11p**) were evaluated for their complexation ability with Zn^2+^, Cu^2+^, Al^3+^, and Fe^3+^ ions according to the method described by Pereira et al.^36,37^ Stock solutions of the metal ions (Zn^2+^, Cu^2+^, Al^3+^, and Fe^3+^) (1 mM) were prepared in water (Tris–HCl buffer, pH = 7.0), whereas stock solutions of the target compounds (**5**, **11b** and **11p**) (1 mM) were prepared in UV-HPLC grade ethanol. Aliquots of 60–80 μL of the compound stock solutions were diluted to 20–26 μM in 3 mL of an ethanol/water mixture (50/50, v/v, Tris–HCl, pH = 7.0). UV–vis spectra were recorded using a Shimadzu UV-2450 spectrophotometer with 1.0 cm quartz cuvettes. Photoluminescence spectra were obtained on an Edinburgh FS5 spectrofluorometer equipped with a single-photon counting detector, using quartz cuvettes with four polished faces and a 1.0 cm optical path length. All spectra were plotted using the Origin 6.0 software.

## Supplementary Material



## Notes

Additional experimental results; intermediates and final compounds ^1^H and ^13^C NMR spectra; HPLC chromatograms of the final compounds; and representative HRMS spectra of the final products (PDF)
