# Supplementary material for: Synthesis and Pharmacological Evaluation of Novel 1,5-Disubstituted-3-amino-1,2,4-triazoles Designed as Multitarget Directed Ligands for Alzheimer’s Disease Targets
Source: ACS Omega. 2026 Feb 17;11(8):13184–98. doi: 10.1021/acsomega.5c09002 (PMC12961494; doi:10.1021/acsomega.5c09002)
Supplement: Supplementary file 1 [file ao5c09002_si_001.pdf]

Supplementary Material

Synthesis and pharmacological evaluation of novel 1,5-disubstituted-3-amino-1,2,4-triazoles designed as multi-target directed ligands for Alzheimer's disease targets.

Daiana Portella Franco,[a,#] Lucas Caruso,[a,#] Danniel Cosme Neves Grillo,[b] Nathália Fonseca Nadur,[a] Luciana Luiz de Azevedo,[a] Thiago Moreira Pereira,[a] Manuelle Cunha da Silva,[a] Renata Barbosa Lacerda,[a,c] Pedro de Sena Murteira Pinheiro,[d] Cristiano Jorge Riger,[b] Arthur Eugen Kümmerle [a,]*

[a] Laboratory of Molecular Diversity and Medicinal Chemistry (LaDMol-QM), Department of Organic Chemistry, Institute of Chemistry, Federal Rural University of Rio de Janeiro, 23897-000 - Seropédica - RJ, Brazil.

[b] Laboratory of Oxidative Stress in Microorganisms, Department of Biochemistry, Institute of Chemistry, Federal Rural University of Rio de Janeiro, 23897-000 - Seropédica - RJ, Brazil

[c] Department of Pharmaceutical Sciences, Institute of Biological and Health Sciences, Federal Rural University of Rio de Janeiro, 23897-000 - Seropédica - RJ, Brazil.

[d] Laboratório de Avaliação e Síntese de Substâncias Bioativas (LASSBio), Instituto de Ciências Biomédicas, Universidade Federal do Rio de Janeiro, P.O. Box 68023, Rio de Janeiro 21941-902, RJ, Brazil.

[#] These authors contributed equally to this work.

CONTENTS

Resazurin cell viability assay……….…………………………………**2**

Absorbance spectrum of 3-amino-1,2,4-triazole compounds……… **3**

Characterization data for synthetic intermediates…………………..**4**

Copies of ^{1}H, ^{13}C NMR……………………………………………**13**

HPLC chromatogram, purity analyses……...……………………..**85**

Representative HRMS spectra ………………………………......**105**

Table S1. Resazurin cell viability assay of **11b**, **11c**, **11m** and **11p** in BY4741 / *Δgsh1* strains.

	**Toxicity[a] BY4741 / *Δgsh1***			
**Concentration (μM)**	**11b**	**11c**	**11m**	**11p**
1000	+/+	+/+	+/+	+/+
500	+/+	+/+	+/+	+/+
250	-/-	-/-	-/-	-/-
125	-/-	-/-	-/-	-/-
62,5	-/-	-/-	-/-	-/-
31,25	-/-	-/-	-/-	-/-
15,625	-/-	-/-	-/-	-/-
7,81	-/-	-/-	-/-	-/-
3,91	-/-	-/-	-/-	-/-
1,95	-/-	-/-	-/-	-/-
0,975	-/-	-/-	-/-	-/-
0,4875	-/-	-/-	-/-	-/-

[a](+) significant toxicity; (-) low/non-existent toxicity

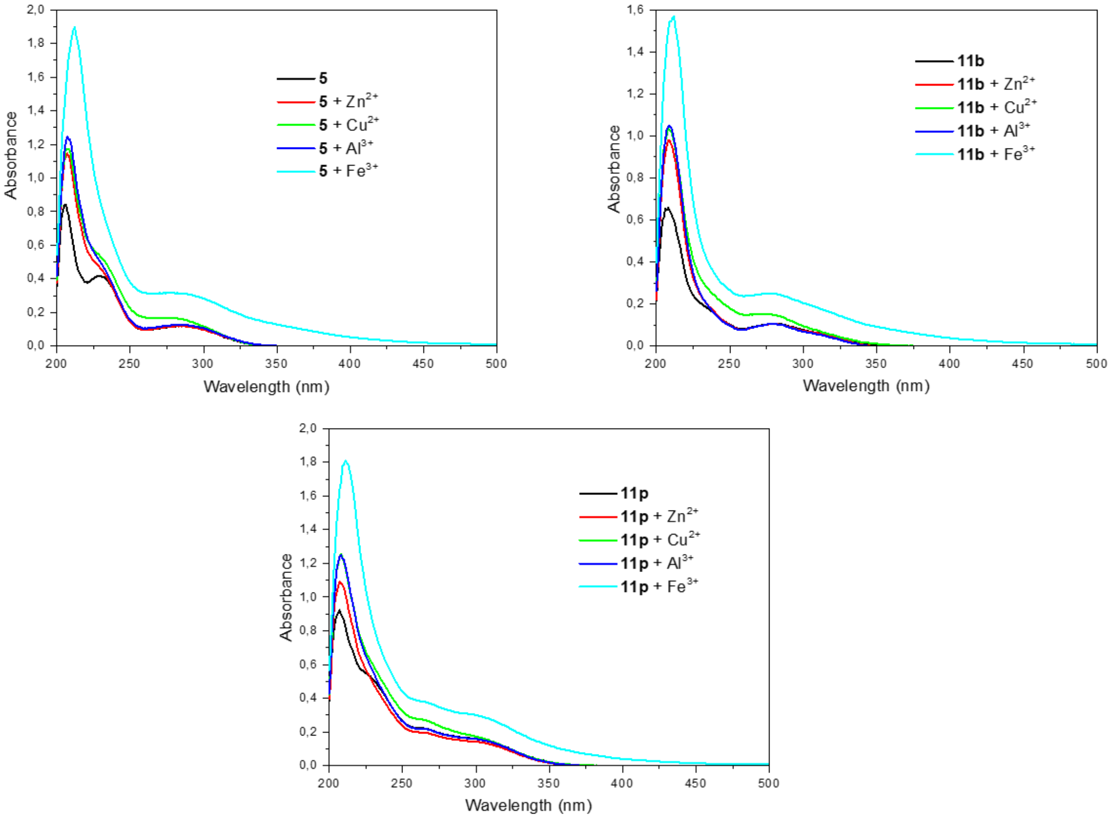

Figure S1: Absorbance spectrum of the **5**, **11b** and **11p** before and after addition of Cu^{2+}, Zn^{2+}, Al^{3+} and Fe^{3+}.

Characterization data for synthetic intermediates

S-methylthiouronium iodide (**13**)

Yellow solid. Yield = 90%. mp = 238-240°C. ^{1}H NMR (500 MHz, DMSO-d_6) (δ ppm): 8.89 (s, 2H), 2.58 (s, 3H); ^{13}C NMR (125 MHz, DMSO-d_6) (δ ppm): 171.09, 13.55. (CAS number: 4338-95-8).

tert-butyl (amino(methylthio)methylene)carbamate (**14**)

White solid. Yield = 85%. mp = 75-78°C. ^{1}H NMR (400 MHz, DMSO) (δ ppm): 8.58 (s, 2H), 2.32 (s, 3H), 1.41 (s, 9H); ^{13}C NMR (100 MHz, DMSO-d_6) (δ ppm): 171.87, 160.98, 78.11, 28.17, 13.13. (CAS number: 173998-77-1).

N-acyl-2-methyl-isothiourea-*N*-Boc (**15a-r**)

tert-butyl (benzamido(methylthio)methylene)carbamate (**15a**)

White solid. Yield = 70%. mp = 100-103°C. ^{1}H NMR (500 MHz, $CDCl_3$) (δ ppm): 8.29 (d, 2H), 7.56 (s, 1H), 7.47 (s, 2H) 2.59 (s, 3H), 1.54 (s, 9H); ^{13}C NMR (125 MHz, $CDCl_3$) (δ ppm): 172.00, 132.81, 130.12, 128.25, 27.98, 14.92. (CAS number: 204584-81-6).

tert-butyl ((4-methoxybenzamido)(methylthio)methylene)carbamate (**15e**)

White solid. Yield = 68%. mp = 97-100°C. ^{1}H NMR (400 MHz, $CDCl_3$) (δ ppm): 8.10 (d, 2H), 7.59 (d, 2H), 3.85 (s, 3H), 2.49 (s, 3H), 1.46 (s, 9H). ^{13}C NMR (100 MHz, $CDCl_3$) (δ ppm): 175.51, 171.08, 163.99, 151.12, 132.32, 129.31, 113.49, 83.26, 55.43, 27.98, 14.86. (CAS number: 2095849-99-1).

tert-butyl ((methylthio)(3,4,5-trimethoxybenzamido)methylene)carbamate (**15f**)

White solid. Yield = 56%. mp = 121-124°C. ^{1}H NMR (400 MHz, $CDCl_3$) (δ ppm): 12.54 (s, 1H), 7.60 (s, 2H), 3.93 (s, 6H), 3.92 (s, 3H), 2.58 (s, 3H), 1.53 (s, 9H). ^{13}C NMR (100 MHz, $CDCl_3$) (δ ppm): 175.05, 171.40, 152.49, 150.74, 142.67, 131.49, 107.10, 83.25, 60.08, 55.81, 27.72, 14.56.

tert-butyl ((2-chlorobenzamido)(methylthio)methylene)carbamate (**15h**)

White solid. Yield = 62%. mp = 72-75°C. ^{1}H NMR (400 MHz, $CDCl_3$) (δ ppm): 12.27 (s, 1H), 7.45-7.34 (m, 4H), 2.50 (s, 3H), 1.53 (s, 9H). ^{13}C NMR (100 MHz, $CDCl_3$) (δ ppm): 131.89, 130.85, 127.12, 126.32, 27.71, 14.66. (CAS number: 2416125-35-2).

tert-butyl ((3-chlorobenzamido)(methylthio)methylene)carbamate (**15i**)

White solid. Yield = 59%. mp = 99-103°C. ^{1}H NMR (400 MHz, $CDCl_3$) (δ ppm): 12.51 (s, 1H), 8.23 (s, 1H), 8.13 (d, 1), 7.53 (d, 1H), 7.40 (t, 1H), 2.59 (s, 3H), 1.54 (s, 9H). ^{13}C NMR (100 MHz, $CDCl_3$) (δ ppm): 133.46, 132.41, 129.31, 128.00, 27.71, 14.74. (CAS number: 2416125-32-9).

tert-butyl ((4-chlorobenzamido)(methylthio)methylene)carbamate (**15j**)

White solid. Yield = 63%. mp = 109-111°C. ^{1}H NMR (500 MHz, $CDCl_3$) (δ ppm): 12.54 (s, 1H), 8.21 (d, 2H), 7.42 (d, 2H), 2.58 (s, 3H), 1.53 (s, 9H); ^{13}C NMR (125 MHz, $CDCl_3$) (δ ppm): 174.93, 172.63, 150.95, 139.17, 135.14, 131.48, 128.55, 83.64, 27.98, 14.97. (CAS number: 2364507-65-1).

tert-butyl ((3-bromobenzamido)(methylthio)methylene)carbamate (**15k**)

White solid. Yield = 59%. mp = 85-87°C. ^{1}H NMR (500 MHz, $CDCl_3$) (δ ppm): 12.53 (s, 1H), 9.11 (s, 1H); 8.57 (d, 1H), 8.39 (d, 1H), 7.65 (t, 1H), 2.64 (s, 3H), 1.55 (s, 9H). ^{13}C NMR (125 MHz, $CDCl_3$) (δ ppm): 174.22; 173.37; 150.76; 138.46; 135.52; 129.36; 126.94; 125.05; 114.05; 84.02; 27.96; 15.13. (CAS number: 2416125-33-0).

tert-butyl ((4-bromobenzamido)(methylthio)methylene)carbamate (**15l**)

White solid. Yield = 66%. mp = 93-96°C. ^{1}H NMR (500 MHz, $CDCl_3$) (δ ppm): 12.53 (s, 1H); 8.11 (d, 2H); 7.59 (d, 2H); 2.57 (s, 3H); 1.53 (s, 9H). ^{13}C NMR (125 MHz, $CDCl_3$) (δ ppm): 172.40; 131.33; 27.72; 14.72. (CAS number: 2416125-29-4).

tert-butyl ((methylthio)(3-nitrobenzamido)methylene)carbamate (**15m**)

Beige solid. Yield = 36%. mp = 138-142°C. ^{1}H NMR (500 MHz, $CDCl_3$) (δ ppm): 12.53 (s, 1H); 9.11 (s, 1H); 8.56 (d, 1H); 8.39 (d, 1H); 7.65 (t, 1H); 2.63 (s, 3H); 1.55 (s, 9H). ^{13}C NMR (125 MHz, $CDCl_3$) (δ ppm): 135.25; 129.11; 126.70; 124.78; 27.70; 14.86. (CAS number: 2416125-30-7).

tert-butyl ((methylthio)(4-nitrobenzamido)methylene)carbamate (**15n**)

Yellow solid. Yield = 58%. mp = 151-154°C. ^{1}H NMR (500 MHz, $CDCl_3$) (δ ppm): 12.53 (s, 1H); 8.42 (d, 2H); 8.29 (d, 2H); 2.61 (s, 3H); 1.55 (s, 9H). ^{13}C NMR (125 MHz, $CDCl_3$) (δ ppm): 174.28; 173.68; 150.76; 150.21; 142.03; 130.95; 123.37; 84.06; 27.95; 15.12. (CAS number: 2095854-09-2).

tert-butyl ((methylthio)(picolinamido)methylene)carbamate (**15o**)

White solid. Yield = 45%. mp = 114-118°C. ^{1}H NMR (500 MHz, $CDCl_3$) (δ ppm): 13.62 (s, 1H); 8.77 (d, 1H); 8.28 (d, 1H); 7.94 (t, 1H); 7.58 (t, 1H); 2.50 (s, 3H); 1.62 (s, 9H). ^{13}C NMR (125 MHz, $CDCl_3$) (δ

ppm): 168.48; 162.90; 159.80; 148.86; 148.12; 137.82; 127.43; 123.49; 81.23; 28.00; 14.54. (CAS number: 2955529-35-6).

tert-butyl ((furan-2-carboxamido)(methylthio)methylene)carbamate (**15p**)

Yellow solid. Yield = 69%. mp = 92-95°C. ^{1}H NMR (500 MHz, $CDCl_3$) (δ ppm): 12.29 (s, 1H); 7.64 (sl, 1H); 7.31 (d, 1H); 6.53 (sl, 1H); 2.51 (s, 3H); 1.53 (s, 9H). ^{13}C NMR (125 MHz, $CDCl_3$) (δ ppm): 147.07; 118.93; 112.06; 27.95; 14.74. (CAS number: 2560592-36-9).

tert-butyl ((methylthio)(thiophene-2-carboxamido)methylene)carbamate (**15q**)

Yellow solid. Yield = 69%. mp = 60-61°C. ^{1}H NMR (500 MHz, $CDCl_3$) (δ ppm): 12.40 (s, 1H); 7.90 (d, 1H); 7.59 (d, 1H); 7.13 (t, 1H); 2.55 (s, 3H); 1.52 (s, 9H). ^{13}C NMR (125 MHz, $CDCl_3$) (δ ppm): 171.91; 170.78; 150.88; 142.57; 133.48; 133.08; 128.23; 83.51; 27.97; 14.86. (CAS number: 2178897-89-5).

1,2,4-triazole-*N*-Boc (**16a-t**)

tert-butyl (5-(2-methoxyphenyl)-1-phenyl-1*H*-1,2,4-triazol-3-yl)carbamate (**16d**)

White solid. Yield = 73%. mp = 98-101°C. ^{1}H NMR (400 MHz, $CDCl_3$) (δ ppm): 7.66 (s, 1H); 7.56 (d, 1H); 7.41 (t, 1H); 7.30–7.28 (m, 5H); 7.03 (t, 1H); 6.78 (d, 1H); 1.51 (s, 9H). ^{13}C NMR (100 MHz, $CDCl_3$) (δ ppm): 156.88; 156.24; 156.22; 151.39; 138.70; 131.96; 131.37; 128.57; 127.65; 123.43; 120.80; 117.46; 111.19; 81.12; 54.76; 28.16. HRMS (ESI) *m/z*: $[M + H]^+$ calcd = 367.1765; found = 367.1766.

tert-butyl (5-(4-methoxyphenyl)-1-phenyl-1*H*-1,2,4-triazol-3-yl)carbamate (**16e**)

Yellow solid. Yield = 62%. mp = 164-167°C. ^{1}H NMR (500 MHz, $CDCl_3$) (δ ppm): 7.59 (s, 1H); 7.40 (m, 7H); 6.83 (d, 2H); 3.81 (s, 3H); 1.51 (s, 9H). ^{13}C NMR (125 MHz, $CDCl_3$) (δ ppm): 161.01; 155.94; 151.28; 138.11; 130.43; 129.25; 128.77; 125.82; 113.94; 81.34; 55.31; 28.18. (CAS number: 2364507-68-4). HRMS (ESI) *m/z*: $[M + H]^+$ calcd = 367.1765; found = 367.1763.

tert-butyl (5-(2-chlorophenyl)-1-phenyl-1*H*-1,2,4-triazol-3-yl)carbamate (**16h**)

Yellow solid. Yield = 80%. mp = 95-98°C. ^{1}H NMR (500 MHz, $CDCl_3$) (δ ppm): 7.45–7.25 (m, 9H); 1.52 (s, 9H). ^{13}C NMR (125 MHz, $CDCl_3$) (δ ppm): 156.30; 151.18; 150.58; 137.47; 133.78; 131.83; 130.07; 128.95; 128.18; 127.85; 126.97; 123.77; 111.65; 81.47; 28.19. HRMS (ESI) *m/z*: $[M + H]^+$ calcd = 371.1269; found = 371.1265.

tert-butyl (5-(3-chlorophenyl)-1-phenyl-1*H*-1,2,4-triazol-3-yl)carbamate (**16i**)

Yellow solid. Yield = 88%. mp = 127-130°C. ^{1}H NMR (500 MHz, $CDCl_3$) (δ ppm): 7.80–7.23 (m, 9H); 1.51 (s, 9H). ^{13}C NMR (125 MHz, $CDCl_3$) (δ ppm): 156.46; 151.55; 151.28; 137.61; 134.64; 130.26; 129.72; 129.39; 129.15; 128.97; 126.88; 125.72; 114.05; 81.46; 28.16. HRMS (ESI) *m/z*: $[M + H]^+$ calcd = 371.1269; found = 371.1263.

tert-butyl (5-(4-chlorophenyl)-1-phenyl-1*H*-1,2,4-triazol-3-yl)carbamate (**16j**)

Yellow solid. Yield = 67%. mp = 140-143°C. ^{1}H NMR (400 MHz, $CDCl_3$) (δ ppm): 7.94 (s, 1H); 7.41 (m, 5H); 7.38 (d, 2H); 7.29 (d, 2H); 1.48 (s, 9H). ^{13}C NMR (100 MHz, $CDCl_3$) (δ ppm): 156.47; 151.93; 151.33; 137.75; 136.37; 130.18; 129.50; 129.00; 128.82; 125.78; 125.74; 81.37; 28.14. (CAS number: 2364507-69-5). HRMS (ESI) *m/z*: $[M + H]^+$ calcd = 371.1269; found = 371.1266.

tert-butyl (5-(3-bromophenyl)-1-phenyl-1*H*-1,2,4-triazol-3-yl)carbamate (**16k**)

Yellow solid. Yield = 84%. mp = 105-108°C. ^{1}H NMR (500 MHz, $CDCl_3$) (δ ppm): 7.72–7.17 (m, 9H); 1.52 (s, 9H). ^{13}C NMR (125 MHz, $CDCl_3$) (δ ppm): 156.42; 151.47; 151.25; 137.61; 133.18; 131.84; 129.92; 129.39; 129.18; 129.16; 127.30; 125.72; 122.61; 81.49; 28.17. HRMS (ESI) *m/z*: $[M + H]^+$ calcd = 415.0764; found = 415.0763.

tert-butyl (5-(4-bromophenyl)-1-phenyl-1*H*-1,2,4-triazol-3-yl)carbamate (**16l**)

Brown solid. Yield = 81%. mp = 143-146°C. ^{1}H NMR (500 MHz, $CDCl_3$) (δ ppm): 8.07 (s, 1H); 7.47–7.34 (m, 9H); 1.50 (s, 9H). ^{13}C NMR (125 MHz, $CDCl_3$) (δ ppm): 156.47; 151.90; 151.34; 136.68; 131.82; 130.36; 129.40; 129.09; 126.11; 125.74; 124.82; 81.38; 28.15. HRMS (ESI) *m/z*: $[M + H]^+$ calcd = 415.0764; found = 415.0763.

tert-butyl (5-(3-nitrophenyl)-1-phenyl-1*H*-1,2,4-triazol-3-yl)carbamate (**16m**)

Yellow solid. Yield = 71%. mp = 153-155°C. ^{1}H NMR (500 MHz, $CDCl_3$) (δ ppm): 8.35 (s, 1H); 8.25–7.37 (m, 9H); 1.54 (s, 9H). ^{13}C NMR (125 MHz, $CDCl_3$) (δ ppm): 156.61; 151.17; 150.61; 148.16; 137.28; 134.40; 129.70; 129.67; 128.92; 125.82; 124.72; 123.77; 81.70; 28.18. HRMS (ESI) *m/z*: $[M + H]^+$ calcd = 382.1510; found = 382.1508.

tert-butyl (5-(4-nitrophenyl)-1-phenyl-1*H*-1,2,4-triazol-3-yl)carbamate (**16n**)

Yellow solid. Yield = 56%. mp = 166-168°C. ^{1}H NMR (400 MHz, $CDCl_3$) (δ ppm): 8.18 (d, 2H); 7.68 (d, 2H); 7.47–7.36 (m, 5H); 1.54 (s, 9H). ^{13}C NMR (100 MHz, $CDCl_3$) (δ ppm): 156.71; 151.10; 151.05; 148.41; 137.40; 133.22; 129.78; 129.67; 129.62; 125.79; 123.72; 81.76; 28.18. (CAS number: 2364507-70-8). HRMS (ESI) *m/z*: $[M + H]^+$ calcd = 382.1510; found = 382.1505.

tert-butyl (1-(4-nitrophenyl)-5-phenyl-1*H*-1,2,4-triazol-3-yl)carbamate (**16o**)

Yellow solid. Yield = 61%. mp = 146-150°C. ^{1}H NMR (400 MHz, $CDCl_3$) (δ ppm): 10.12 (s, 1H), 8.34 (d, 2H), 7.74 (m, 5H), 7.65 (d, 2H), 1.49 (s, 9H). ^{13}C NMR (100 MHz, $CDCl_3$) (δ ppm): 157.53, 153.88, 152.38, 147.19, 143.08, 131.21, 129.52, 129.50, 127.85, 126.48, 125.52, 80.19, 28.61. (CAS number: 2364507-71-9). HRMS (ESI) *m/z*: $[M + H]^+$ calcd = 382.1510; found = 382.1503.

tert-butyl (1-phenyl-5-(pyridin-2-yl)-1*H*-1,2,4-triazol-3-yl)carbamate (**16p**)

White solid. Yield = 85%. mp = 165-167°C. ^{1}H NMR (500 MHz, $CDCl_3$) (δ ppm): 8.49 (s, 9H), 7.94 (s, 1H), 7.83 (d, 1H), 7.75 (t, 1H), 7.38 (m, 5H), 7.30 (t, 1H), 1.49 (s, 9H). ^{13}C NMR (125 MHz, $CDCl_3$) (δ ppm): 156.12, 151.29, 149.30, 146.67, 138.42, 136.72, 128.74, 128.56, 125.77, 124.36, 124.35, 81.33, 28.14. HRMS (ESI) *m/z*: $[M + H]^+$ calcd = 338.1612; found = 338.1607.

tert-butyl (1-phenyl-5-(thiophen-2-yl)-1*H*-1,2,4-triazol-3-yl)carbamate (**16r**)

Beige solid. Yield = 69%. mp = 190-192°C. ^{1}H NMR (500 MHz, $CDCl_3$) (δ ppm): 7.61 (s, 1H), 7.50 (m, 5H), 7.37 (d, 1H), 7.04 (d, 1H), 6.94 (t, 1H), 1.50 (s, 9H). ^{13}C NMR (125 MHz, $CDCl_3$) (δ ppm): 156.21, 151.17, 148.38, 137.58, 129.86, 129.43, 128.85, 128.76, 128.16, 127.57, 127.10, 81.32, 28.16. HRMS (ESI) *m/z*: $[M + H]^+$ calcd = 343.1223; found = 343.1219.

tert-butyl (5-(naphthalen-2-yl)-1-phenyl-1*H*-1,2,4-triazol-3-yl)carbamate (**16s**)

Beige solid. Yield = 48%. mp = 115-118°C. ^{1}H NMR (500 MHz, $CDCl_3$) (δ ppm): 7.92 (m, 3H), 7.50 (t, 2H), 7.40 (m, 2H), 7.32 (s, 1H), 7.26–7.16 (m, 5H), 1.55 (s, 9H). ^{13}C NMR (125 MHz, $CDCl_3$) (δ ppm): 156.39, 152.06, 151.26, 137.52, 133.56, 131.27, 130.71, 128.98, 128.87, 128.39, 127.98, 127.29, 126.50, 125.50, 125.15, 124.84, 124.12, 81.47, 28.22. HRMS (ESI) *m/z*: $[M + H]^+$ calcd = 387.1816; found = 387.1809.

***N*-alkyl-1,2,4-triazole-*N*-Boc (17a-t)**

tert-butyl (3-bromopropyl)(1-phenyl-5-(o-tolyl)-1*H*-1,2,4-triazol-3-yl)carbamate (**17a**)

Brown oil. Yield = 65%. ^{1}H NMR (500 MHz, $CDCl_3$) (δ ppm): 7.48-7.37 (m, 7H); 7.24-7.20 (m, 2H); 4.00 (t, 2H); 3.55 (t, 2H); 2.34 (m, 2H); 2.13 (s, 3H); 1.54 (s, 9H). ^{13}C NMR (125 MHz, $CDCl_3$) (δ ppm): 137.25; 135.05; 130.33; 129.85; 129.31; 129.30; 128.73; 127.67; 126.00; 84.55; 46.75; 31.09; 30.69; 27.96; 19.40.

tert-butyl (3-bromopropyl)(5-(2-chlorophenyl)-1-phenyl-1*H*-1,2,4-triazol-3-yl)carbamate (**17h**)

Brown oil. Yield = 53%. ^{1}H NMR (500 MHz, $CDCl_3$) (δ ppm): 7.58 (s, 1H); 7.45 (m, 4H); 7.37 (m, 3H); 7.26 (m, 1H); 4.00 (t, 2H); 3.55 (t, 2H); 2.30 (m, 2H); 1.55 (s, 9H). ^{13}C NMR (125 MHz, $CDCl_3$) (δ ppm): 159.53; 153.29; 151.95; 137.62; 134.64; 130.22; 129.74; 129.49; 129.24; 129.22; 129.05; 126.91; 125.53; 81.78; 47.07; 32.10; 30.95; 28.29.

tert-butyl (3-bromopropyl)(5-(3-chlorophenyl)-1-phenyl-1*H*-1,2,4-triazol-3-yl)carbamate (**17i**)

Brown oil. Yield = 45%. ^{1}H NMR (500 MHz, $CDCl_3$) (δ ppm): 7.48 (d, 1H); 7.41 (m, 3H); 7.31 (m, 5H); 4.02 (t, 2H); 3.55 (t, 2H); 2.31 (m, 2H); 1.54 (s, 9H). ^{13}C NMR (125 MHz, $CDCl_3$) (δ ppm): 159.43; 153.32; 150.96; 137.48; 133.95; 131.86; 131.67; 130.06; 129.09; 128.29; 127.09; 123.62; 116.80; 81.68; 47.07; 32.11; 30.99; 28.28.

tert-butyl (3-bromopropyl)(5-(4-chlorophenyl)-1-phenyl-1*H*-1,2,4-triazol-3-yl)carbamate (**17j**)

Brown oil. Yield = 79%. ^{1}H NMR (500 MHz, $CDCl_3$) (δ ppm): 7.45–7.42 (m, 5H); 7.36 (d, 2H); 7.32 (d, 2H); 4.00 (t, 2H); 3.54 (t, 2H); 2.31 (m, 2H); 1.54 (s, 9H). ^{13}C NMR (125 MHz, $CDCl_3$) (δ ppm): 159.23; 153.04; 152.05; 137.48; 136.10; 129.94; 129.22; 128.86; 128.59; 125.76; 125.27; 81.47; 46.81; 31.84; 30.70; 28.03.

tert-butyl (5-(3-bromophenyl)-1-phenyl-1*H*-1,2,4-triazol-3-yl)(3-bromopropyl)carbamate (**17k**)

Brown oil. Yield = 58%. ^{1}H NMR (500 MHz, $CDCl_3$) (δ ppm): 7.75 (s, 1H); 7.53 (d, 1H); 7.45 (m, 2H); 7.37 (m, 3H); 7.31 (d, 1H); 7.18 (m, 1H); 4.00 (t, 2H); 3.55 (t, 2H); 2.31 (m, 2H); 1.55 (s, 9H). ^{13}C NMR (125 MHz, $CDCl_3$) (δ ppm): 159.53; 153.28; 151.81; 137.59; 133.13; 131.94; 129.93; 129.59; 129.49; 129.25; 127.33; 125.52; 122.02; 81.79; 47.07; 32.09; 30.95; 28.29.

tert-butyl (5-(4-bromophenyl)-1-phenyl-1*H*-1,2,4-triazol-3-yl)(3-bromopropyl)carbamate (**17l**)

Brown oil. Yield = 37%. ^{1}H NMR (500 MHz, $CDCl_3$) (δ ppm): 7.50–7.35 (m, 9H); 4.00 (t, 2H); 3.54 (t, 2H); 2.31 (m, 2H); 1.54 (s, 9H). ^{13}C NMR (125 MHz, $CDCl_3$) (δ ppm): 159.52; 153.37; 152.37; 137.74; 131.82; 130.40; 129.51; 129.16; 126.47; 125.54; 124.74; 81.76; 47.09; 32.11; 30.97; 28.30.

tert-butyl (3-bromopropyl)(5-(3-nitrophenyl)-1-phenyl-1*H*-1,2,4-triazol-3-yl)carbamate (**17m**)

Brown oil. Yield = 70%. ^{1}H NMR (500 MHz, $CDCl_3$) (δ ppm): 8.37 (s, 1H); 8.25 (d, 1H); 7.81 (d, 1H); 7.53 (t, 1H); 7.48 (m, 3H); 7.38 (m, 2H); 4.02 (t, 2H); 3.55 (t, 2H); 2.32 (m, 2H); 1.56 (s, 9H). ^{13}C NMR (125 MHz, $CDCl_3$) (δ ppm): 159.74; 153.15; 150.92; 148.15; 137.30; 134.44; 133.63; 129.77; 129.67; 129.17; 125.00; 124.66; 123.82; 81.95; 47.09; 32.03; 30.97; 28.27.

tert-butyl (3-bromopropyl)(5-(4-nitrophenyl)-1-phenyl-1*H*-1,2,4-triazol-3-yl)carbamate (**17l**)

Brown oil. Yield = 72%. ^{1}H NMR (500 MHz, $CDCl_3$) (δ ppm): 8.19 (d, 2H); 7.70 (m, 2H); 7.68 (d, 2H); 7.48 (m, 3H); 4.01 (t, 2H); 3.54 (t, 2H); 2.31 (m, 2H); 1.56 (s, 9H). ^{13}C NMR (125 MHz, $CDCl_3$) (δ ppm): 159.61; 152.86; 150.77; 148.16; 137.14; 133.14; 129.55; 129.48; 129.39; 125.35; 123.46; 81.93; 46.81; 31.77; 30.61; 28.01.

tert-butyl (3-bromopropyl)(1-(4-nitrophenyl)-5-phenyl-1*H*-1,2,4-triazol-3-yl)carbamate (**17n**)

Brown oil. Yield = 74%. ^{1}H NMR (500 MHz, $CDCl_3$) (δ ppm): 8.26 (d, 2H); 7.55 (d, 2H); 7.47 (m, 2H); 7.42 (m, 3H); 4.03 (t, 2H); 3.54 (t, 2H); 2.32 (m, 2H); 1.57 (s, 9H). ^{13}C NMR (125 MHz, $CDCl_3$) (δ ppm): 160.39; 154.18; 153.21; 147.15; 142.83; 131.11; 129.30; 129.23; 127.19; 125.50; 125.00; 82.35; 47.23; 32.30; 31.07; 28.53.

tert-butyl (3-bromopropyl)(1-phenyl-5-(pyridin-2-yl)-1*H*-1,2,4-triazol-3-yl)carbamate (**17p**)

Brown oil. Yield = 78%. ^{1}H NMR (500 MHz, $CDCl_3$) (δ ppm): 8.52 (d, 1H); 7.89 (d, 1H); 7.80 (t, 1H); 7.41 (m, 5H); 7.35 (m, 1H); 4.00 (t, 2H); 3.54 (t, 2H); 2.32 (m, 2H); 1.54 (s, 9H). ^{13}C NMR (125 MHz, $CDCl_3$) (δ ppm): 47.39; 32.27; 29.94; 28.53.

tert-butyl (3-bromopropyl)(1-phenyl-5-(thiophen-2-yl)-1*H*-1,2,4-triazol-3-yl)carbamate (**17r**)

Brown oil. Yield = 55%. ^{1}H NMR (500 MHz, $CDCl_3$) (δ ppm): 8.11 (d, 1H); 7.94 (d, 1H); 7.80–7.30 (m, 5H); 7.20 (m, 1H); 4.35 (t, 2H); 4.15 (t, 2H); 2.42 (m, 2H); 1.56 (s, 9H). ^{13}C NMR (125 MHz, $CDCl_3$) (δ ppm): 154.02; 151.86; 151.10; 142.76; 131.20; 129.42; 129.41; 128.80; 126.11; 125.80; 84.81; 45.13; 37.64; 27.68; 20.34.

***N*-propyl-benzylpiperazinyl-1,2,4-triazole-*N*-Boc (18a-t)**

tert-butyl (3-(4-benzylpiperazin-1-yl)propyl)(5-(2-methoxyphenyl)-1-phenyl-1*H*-1,2,4-triazol-3-yl)carbamate (**18d**)

Brown oil. Yield = 36%. ^{1}H NMR (500 MHz, $CDCl_3$) (δ ppm): 8.14–8.13 (t, 1H); 7.67 (t, 1H); 7.30 (m, 2H); 7.16 (t, 1H); 7.04 (d, 1H); 3.73 (s, 3H); 3.57 (s, 2H); 3.21 (t, 2H); 2.80 (m, 10H); 2.50 (m, 2H); 1.56 (s, 9H). ^{13}C NMR (125 MHz, $CDCl_3$) (δ ppm): 135.66; 133.17; 129.00; 128.96; 128.80; 128.45; 128.40; 127.51; 122.29; 111.45; 62.35; 55.96; 55.95; 49.21; 43.51; 42.60; 27.99; 20.37.

tert-butyl (3-(4-benzylpiperazin-1-yl)propyl)(5-(4-methoxyphenyl)-1-phenyl-1*H*-1,2,4-triazol-3-yl)carbamate (**18e**)

Brown oil. Yield = 38%. ^{1}H NMR (500 MHz, $CDCl_3$) (δ ppm): 7.40–7.41 (m, 5H); 7.37–7.39 (m, 2H); 7.27–7.32 (m, 5H); 6.82–6.84 (d, 2H); 3.87 (t, 2H); 3.81 (s, 3H); 3.54 (s, 2H); 2.58 (m, 10H); 2.00 (m, 2H); 1.51 (s, 9H). ^{13}C NMR (125 MHz, $CDCl_3$) (δ ppm): 160.89; 159.18; 153.53; 153.22; 138.15; 130.43; 129.34; 129.31; 128.74; 128.29; 127.26; 125.55; 119.94; 113.91; 81.30; 62.75; 55.50; 55.31; 52.28; 46.62; 46.62; 28.31; 25.56.

tert-butyl (3-(4-benzylpiperazin-1-yl)propyl)(5-(4-fluorophenyl)-1-phenyl-1*H*-1,2,4-triazol-3-yl)carbamate (**18g**)

Brown oil. Yield = 35%. ^{1}H NMR (500 MHz, $CDCl_3$) (δ ppm): 7.46 (m, 3H); 7.33 (m, 7H); 7.11 (d, 2H); 3.91 (t, 2H); 3.58 (s, 2H); 2.73 (m, 10H); 2.05 (m, 2H); 1.53 (s, 9H). ^{13}C NMR (125 MHz, $CDCl_3$) (δ ppm): 163.33; 161.34; 159.27; 153.36; 134.00; 130.20; 129.35; 128.86; 128.59; 128.33; 127.38; 127.33; 116.24; 81.53; 62.56; 55.40; 52.53; 46.41; 46.41; 28.29; 25.24.

tert-butyl (3-(4-benzylpiperazin-1-yl)propyl)(5-(4-chlorophenyl)-1-phenyl-1*H*-1,2,4-triazol-3-yl)carbamate (**18j**)

Brown oil. Yield = 41%. ^{1}H NMR (500 MHz, $CDCl_3$) (δ ppm): 7.43–7.32 (m, 14H); 4.50 (m, 2H); 3.89 (t, 2H); 3.52 (s, 2H); 2.48 (m, 10H); 1.96 (m, 2H); 1.54 (s, 9H). ^{13}C NMR (125 MHz, $CDCl_3$) (δ ppm): 159.51; 153.34; 152.11; 137.77; 136.60; 130.17; 129.43; 129.25; 129.02; 128.81; 128.20; 127.49; 126.08; 125.49; 81.34; 62.96; 55.59; 52.83; 46.76; 28.53; 28.28.

tert-butyl (3-(4-benzylpiperazin-1-yl)propyl)(5-(3-bromophenyl)-1-phenyl-1*H*-1,2,4-triazol-3-yl)carbamate (**18k**)

Brown oil. Yield = 59%. ^{1}H NMR (500 MHz, $CDCl_3$) (δ ppm): 7.73 (m, 1H); 7.53 (d, 1H); 7.44 (m, 3H); 7.31 (m, 8H); 7.16 (t, 1H); 3.89 (t, 2H); 3.55 (s, 2H); 2.59 (m, 10H); 2.01 (m, 2H); 1.52 (s, 9H). ^{13}C NMR (125 MHz, $CDCl_3$) (δ ppm): 159.46; 153.34; 151.68; 137.62; 133.09; 131.91; 129.97; 129.47; 129.35; 129.18; 128.32; 127.31; 125.51; 122.61; 81.53; 62.72; 55.48; 52.85; 46.57; 29.70; 28.30.

tert-butyl (3-(4-benzylpiperazin-1-yl)propyl)(5-(4-bromophenyl)-1-phenyl-1*H*-1,2,4-triazol-3-yl)carbamate (**18l**)

Brown oil. Yield = 54%. ^{1}H NMR (500 MHz, $CDCl_3$) (δ ppm): 7.46 (d, 2H); 7.43 (m, 3H); 7.35 (m, 4H); 7.31 (m, 5H); 3.88 (t, 2H); 3.54 (s, 2H); 2.57 (m, 10H); 2.00 (m, 2H); 1.52 (s, 9H). ^{13}C NMR (125 MHz, $CDCl_3$) (δ ppm): 159.48; 153.36; 151.88; 137.77; 131.80; 130.40; 129.48; 129.33; 129.09; 128.31; 127.29; 126.53; 125.52; 124.68; 81.47; 62.80; 55.55; 52.78; 46.62; 25.55; 28.31.

tert-butyl (3-(4-benzylpiperazin-1-yl)propyl)(5-(3-nitrophenyl)-1-phenyl-1*H*-1,2,4-triazol-3-yl)carbamate (**18m**)

Brown oil. Yield = 43%. ^{1}H NMR (500 MHz, $CDCl_3$) (δ ppm): 8.39 (s, 1H); 8.27 (d, 1H); 7.83 (d, 1H); 7.54 (t, 1H); 7.49 (m, 3H); 7.40 (m, 2H); 7.34 (m, 5H); 3.94 (t, 2H); 3.61 (s, 2H); 2.12 (m, 10H); 2.10 (m, 2H); 1.53 (s, 9H). ^{13}C NMR (125 MHz, $CDCl_3$) (δ ppm): 134.51; 129.79; 129.44; 129.11; 128.42; 127.58; 125.65; 124.68; 123.83; 62.45; 55.37; 52.44; 46.36; 43.92; 29.70; 28.29.

tert-butyl (3-(4-benzylpiperazin-1-yl)propyl)(1-phenyl-5-(pyridin-2-yl)-1*H*-1,2,4-triazol-3-yl)carbamate (**18p**)

Brown oil. Yield = 48%. ^{1}H NMR (500 MHz, $CDCl_3$) (δ ppm): 8.47 (d, 1H); 7.92 (d, 1H); 7.78 (t, 1H); 7.40 (m, 5H); 7.32 (m, 5H); 7.26 (t, 1H); 3.89 (t, 2H); 3.52 (s, 2H); 2.50 (m, 10H); 1.96 (m, 2H); 1.53 (s, 9H). ^{13}C NMR (125 MHz, $CDCl_3$) (δ ppm): 159.22; 153.45; 151.70; 149.22; 138.58; 136.70; 129.25; 128.73; 128.48; 128.19; 127.06; 125.53; 124.40; 124.37; 81.26; 62.96; 55.62; 52.98; 46.81; 28.29; 25.89.

tert-butyl (3-(4-benzylpiperazin-1-yl)propyl)(1-phenyl-5-(thiophen-2-yl)-1*H*-1,2,4-triazol-3-yl)carbamate (**18r**)

Brown oil. Yield = 55%. ^{1}H NMR (500 MHz, $CDCl_3$) (δ ppm): 7.50 (d, 1H); 7.41 (d, 1H); 7.31 (m, 10H); 7.15 (m, 1H); 3.83 (t, 2H); 3.55 (s, 2H); 2.55 (m, 10H); 1.94 (m, 2H); 1.50 (s, 9H). ^{13}C NMR (125 MHz, $CDCl_3$) (δ ppm): 159.01; 154.06; 153.76; 136.84; 135.92; 130.25; 129.55; 129.37; 129.20; 128.51; 128.33; 127.48; 126.79; 126.20; 81.53; 62.99; 55.71; 53.01; 46.77; 28.50; 25.67.

tert-butyl (3-(4-benzylpiperazin-2-yl)propyl)(5-(naphthalen-1-yl)-1-phenyl-1*H*-1,2,4-triazol-3-yl)carbamate (**18s**)

Brown oil. Yield = 51%. ^{1}H NMR (500 MHz, $CDCl_3$) (δ ppm): 7.59–7.36 (m, 14H); 4.01 (t, 2H); 3.61 (s, 2H); 2.71 (m, 10H); 2.12 (m, 2H); 1.61 (s, 9H). ^{13}C NMR (125 MHz, $CDCl_3$) (δ ppm): 159.39; 153.52; 152.24; 133.56; 131.37; 130.67; 129.30; 128.97; 128.39; 128.29; 128.07; 127.24; 126.49; 125.51; 125.16; 124.83; 123.84; 81.46; 62.62; 55.49; 52.64; 46.62; 28.34; 25.43.

Copies of ^{1}H, ^{13}C NMR

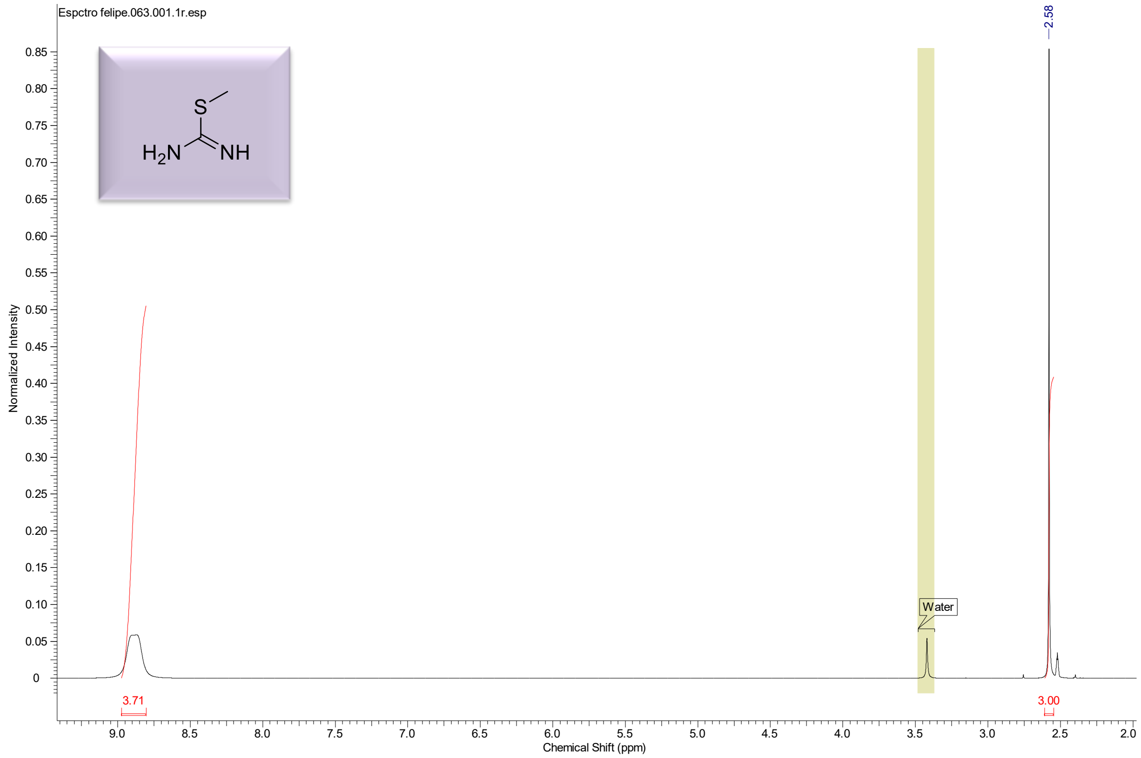

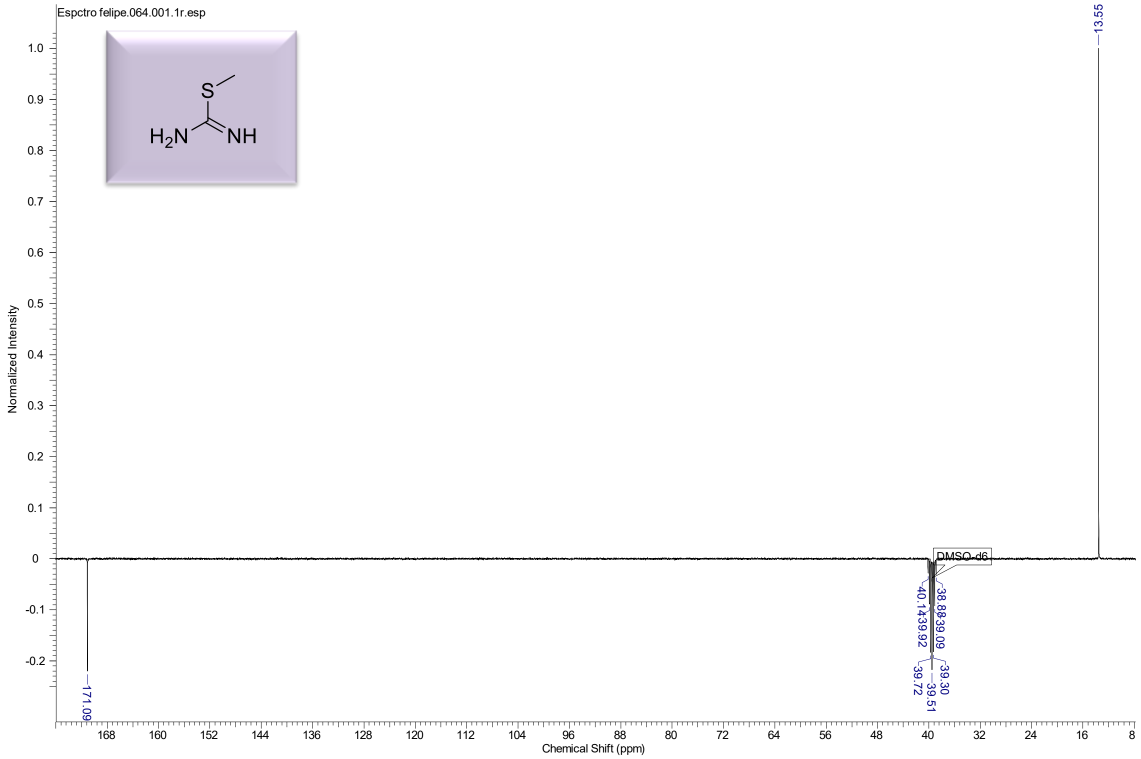

Figure S2: ^{1}H NMR (500 MHz) and ^{13}C NMR (125 MHz) spectra of compound **13** in DMSO-d_6.

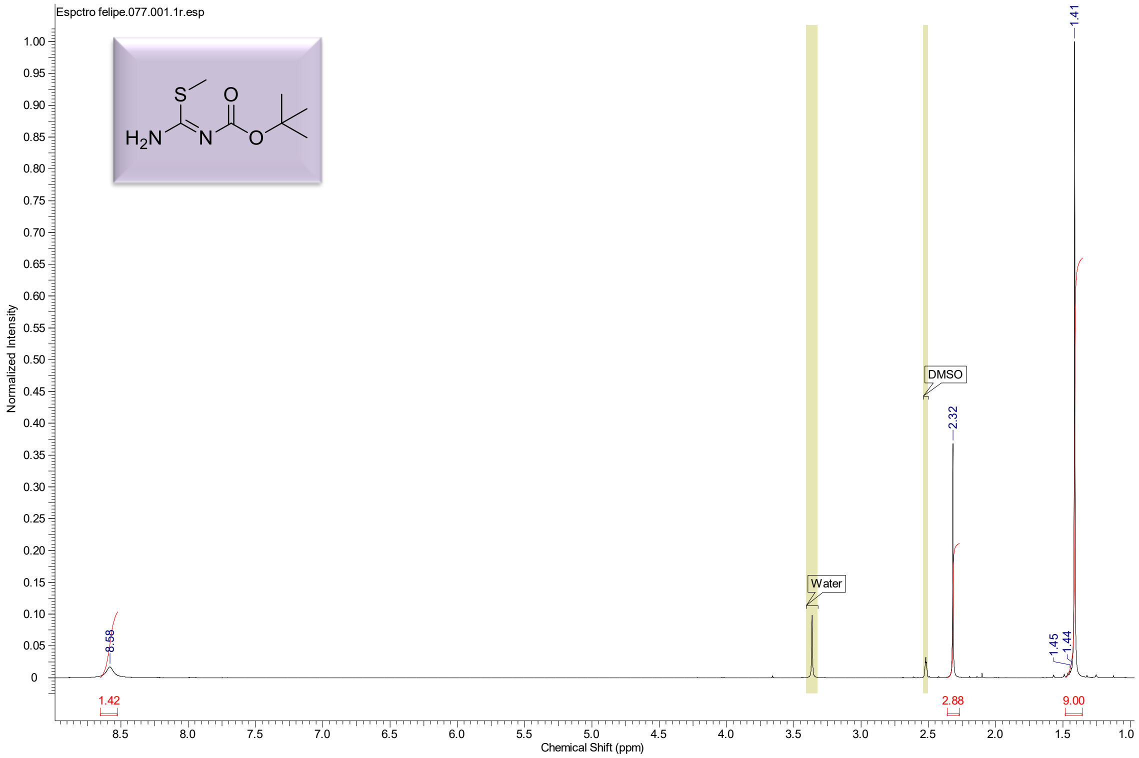

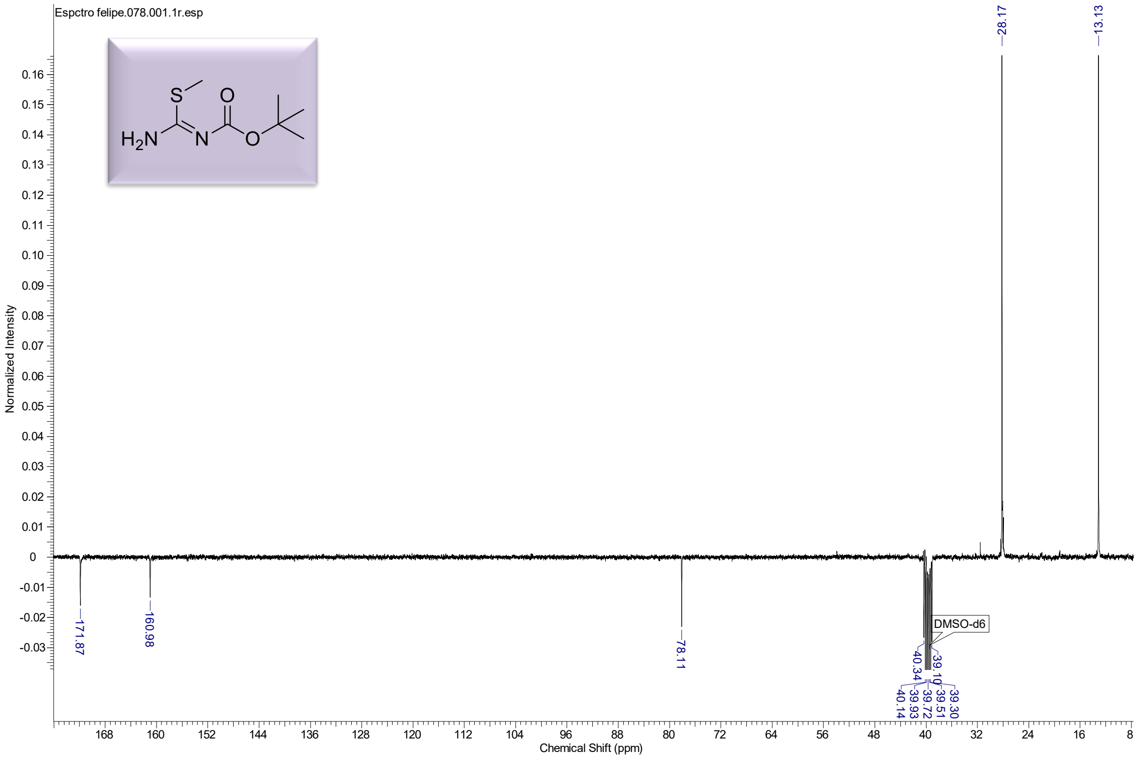

Figure S2: ^{1}H NMR (400 MHz) and ^{13}C NMR (100 MHz) spectra of compound **14** in DMSO-d_6.

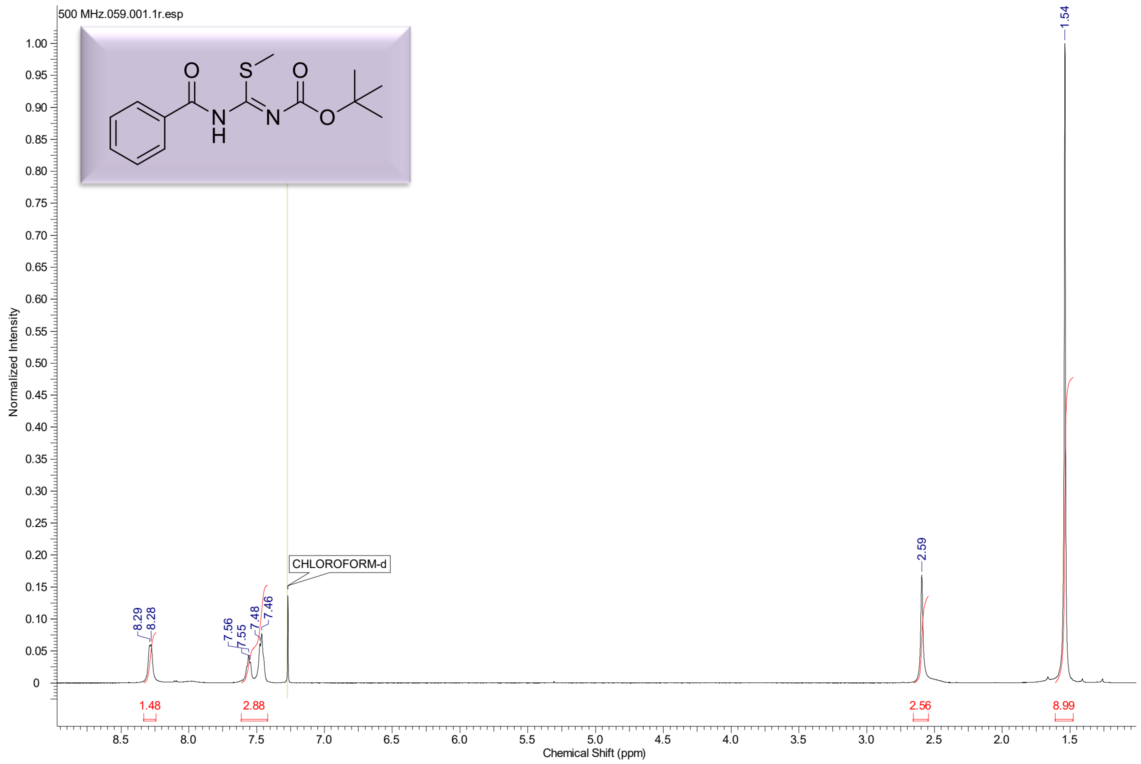

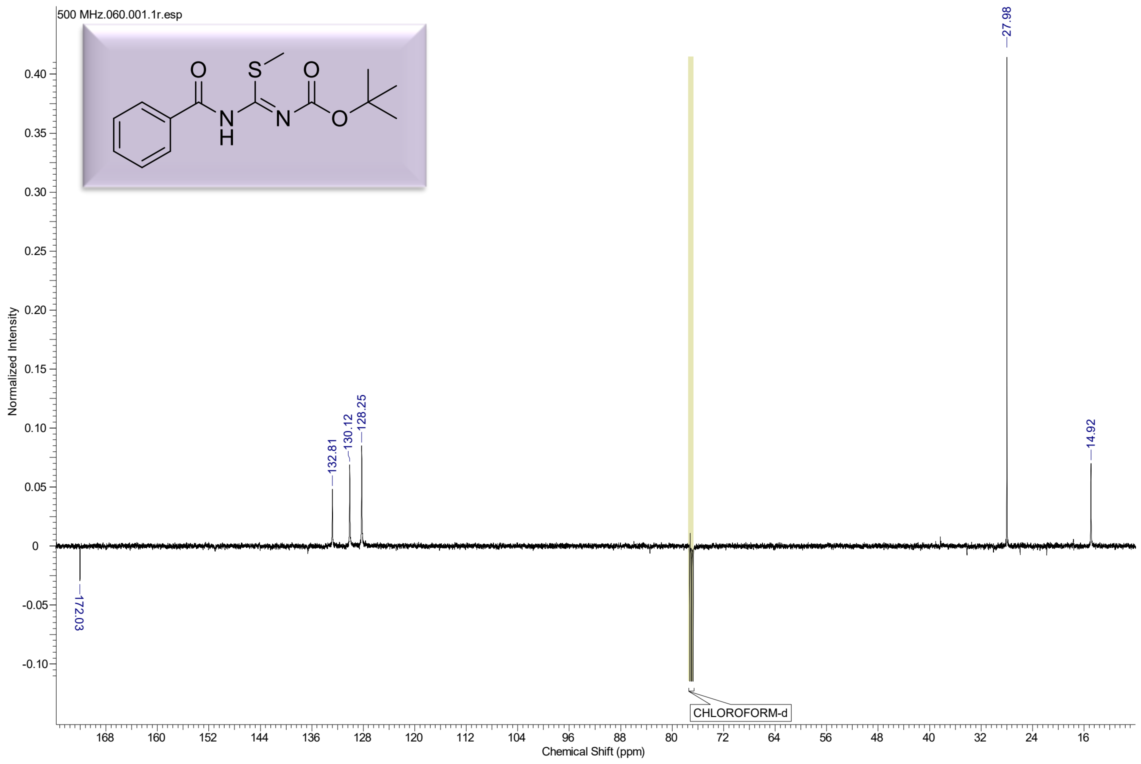

Figure S3: ^{1}H NMR (500 MHz) and ^{13}C NMR (125 MHz) spectra of compound **15a** in $CDCl_3$.

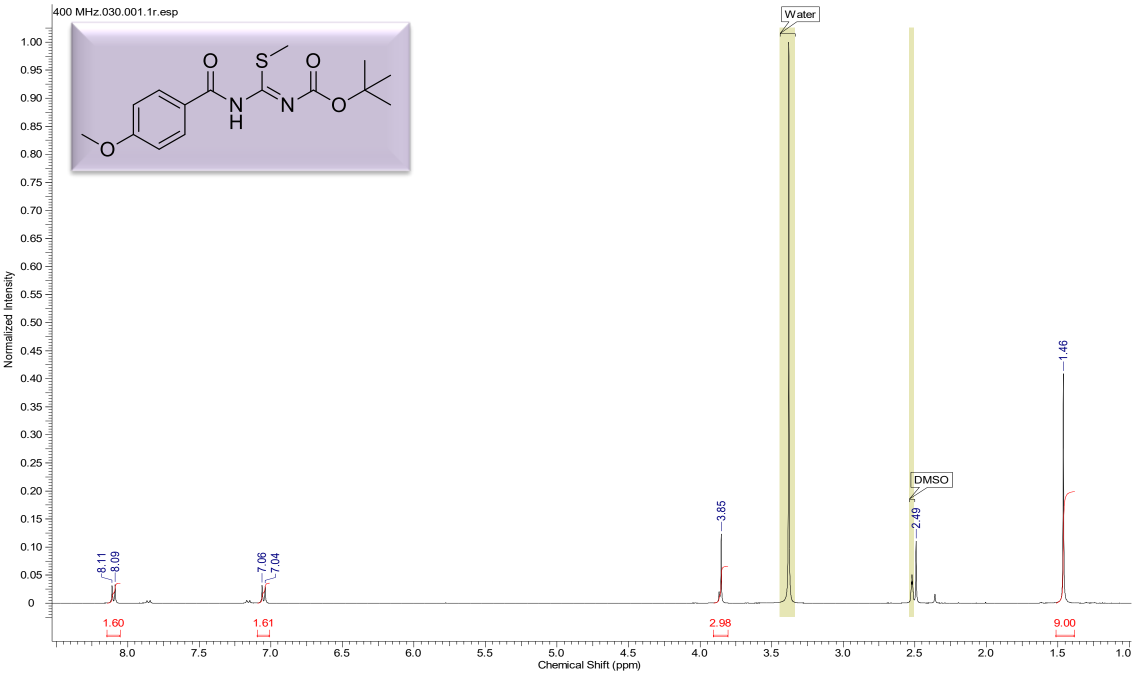

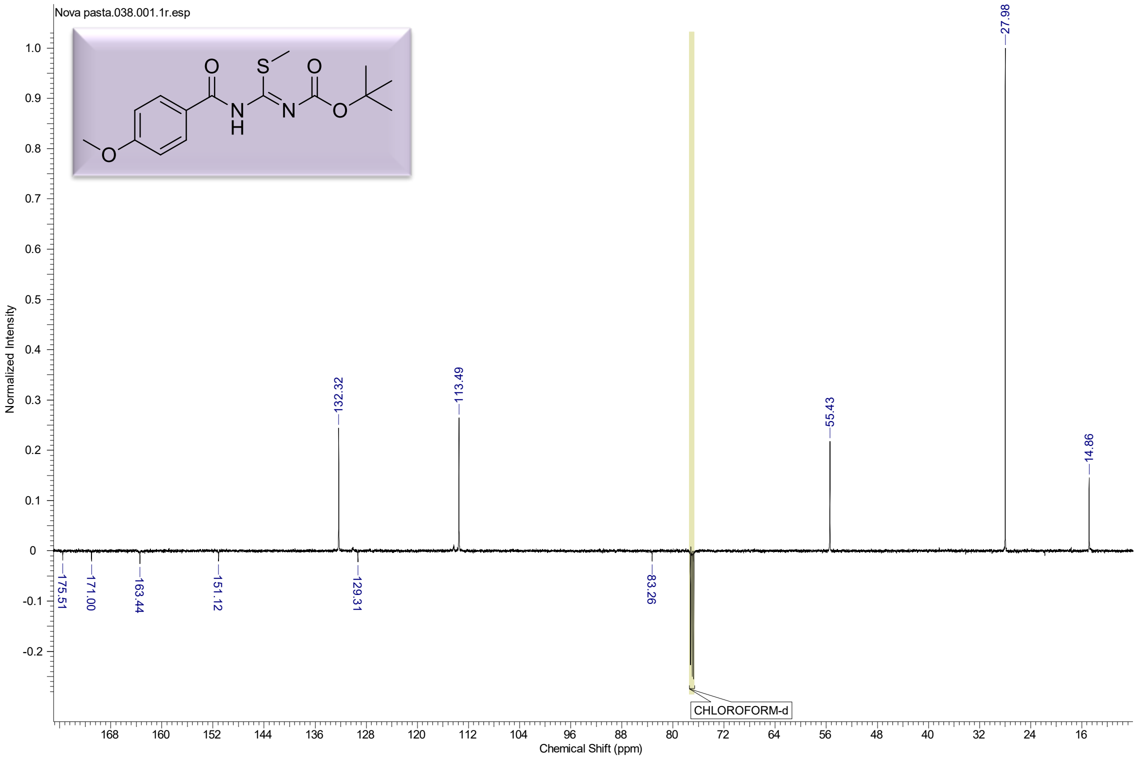

Figure S4: ^{1}H NMR (400 MHz) and ^{13}C NMR (100 MHz) spectra of compound **15b** in DMSO-d_6.

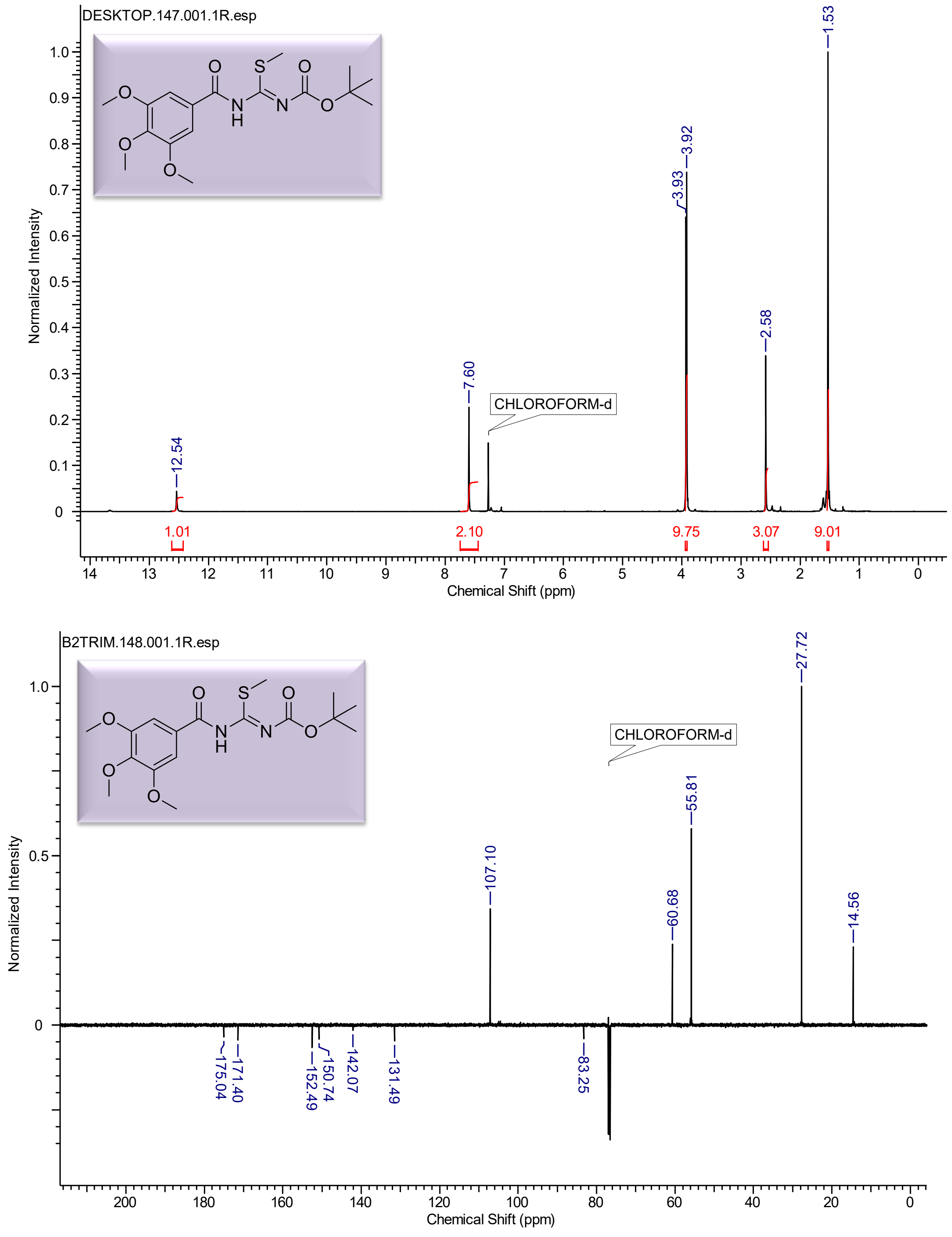

Figure S5: ^{1}H NMR (400 MHz) and ^{13}C NMR (100 MHz) spectra of compound **15c** in $CDCl_3$.

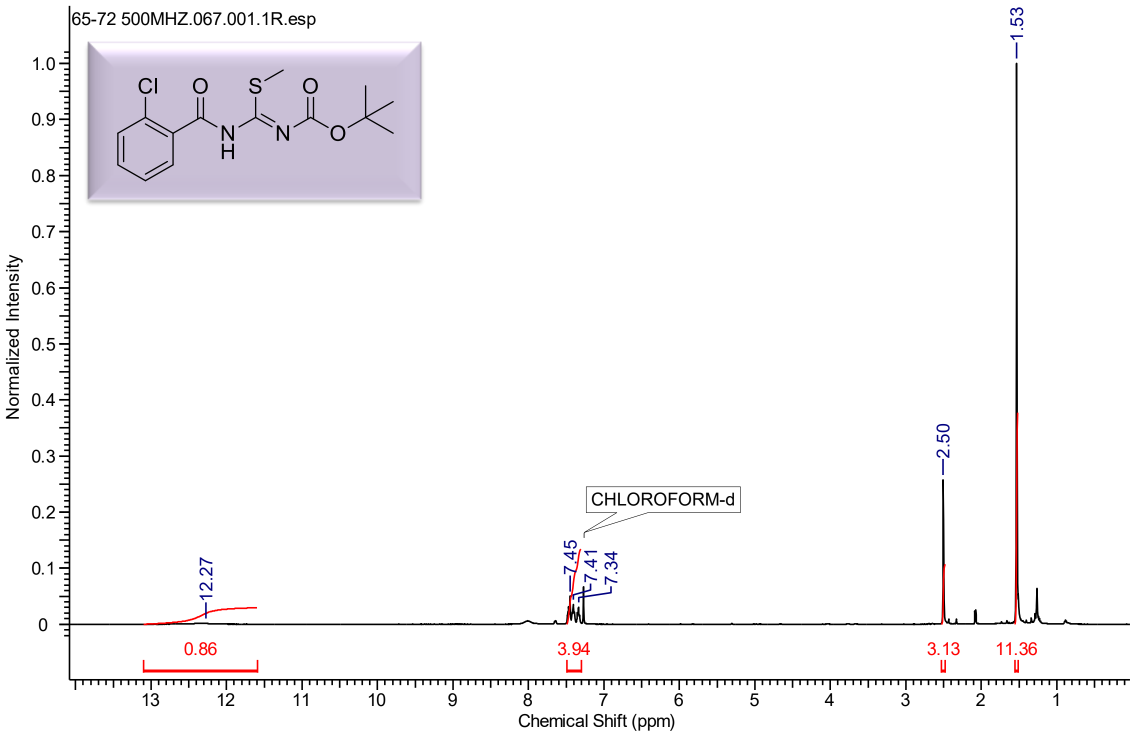

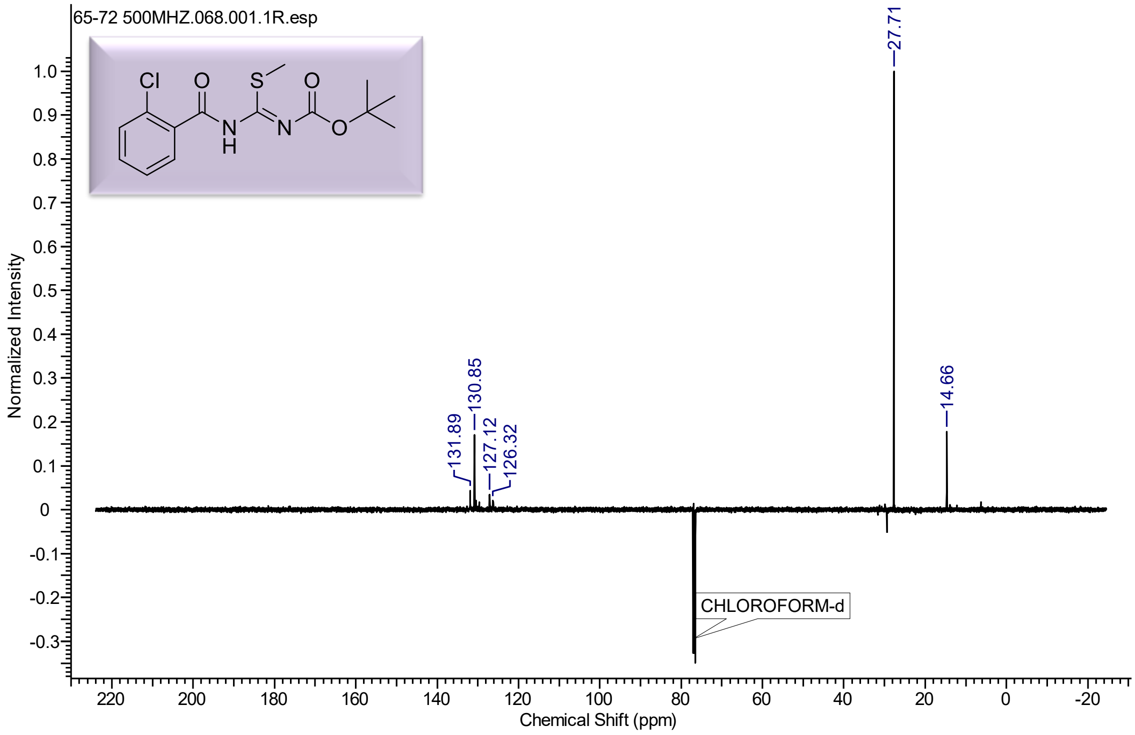

Figure S6: ^{1}H NMR (400 MHz) and ^{13}C NMR (100 MHz) spectra of compound **15d** in $CDCl_3$.

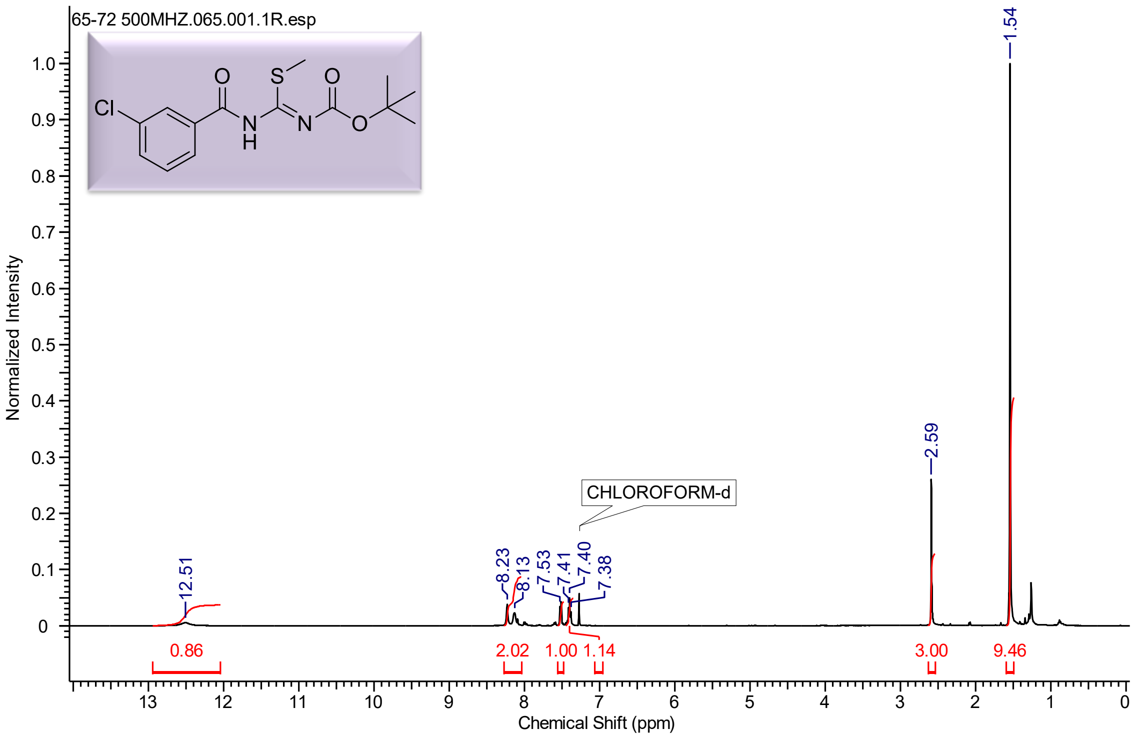

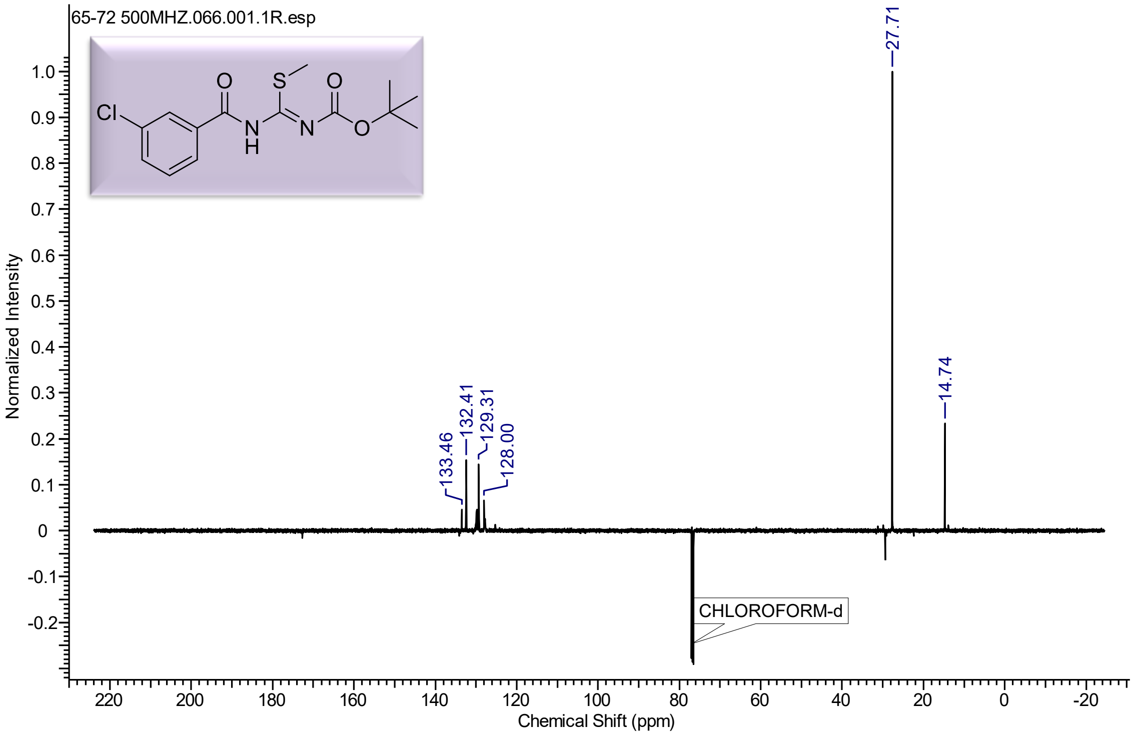

Figure S7: ^{1}H NMR (400 MHz) and ^{13}C NMR (100 MHz) spectra of compound **15e** in $CDCl_3$.

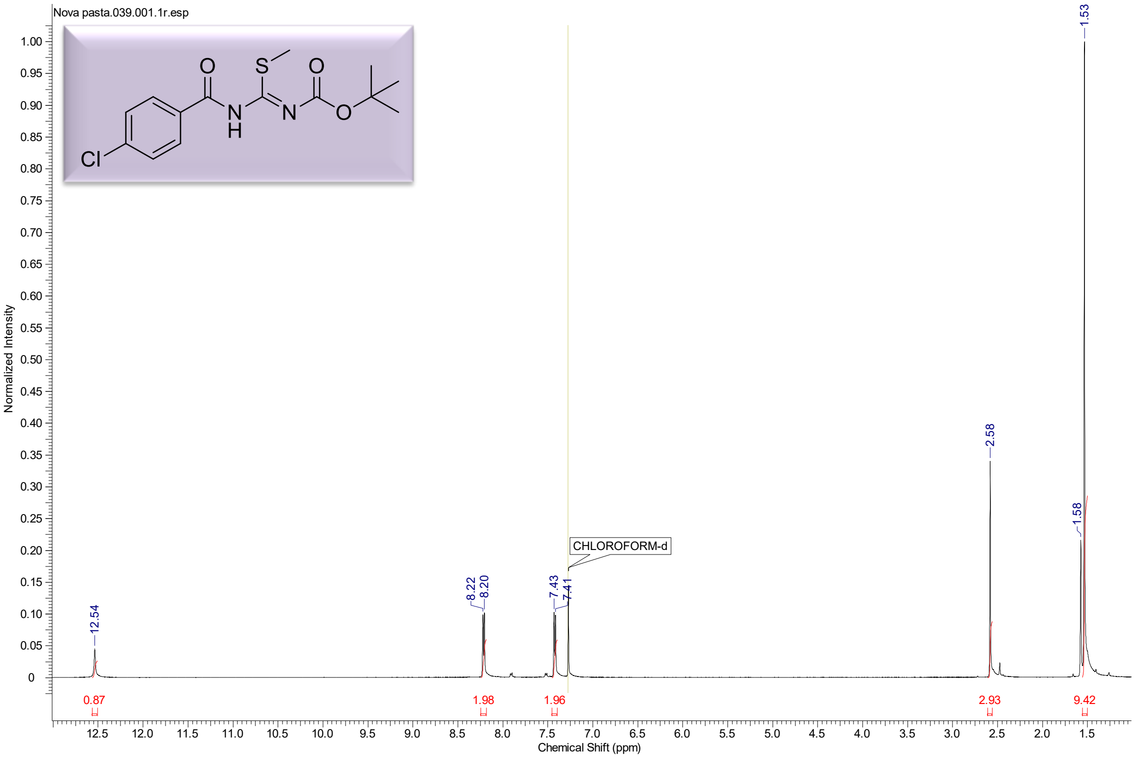

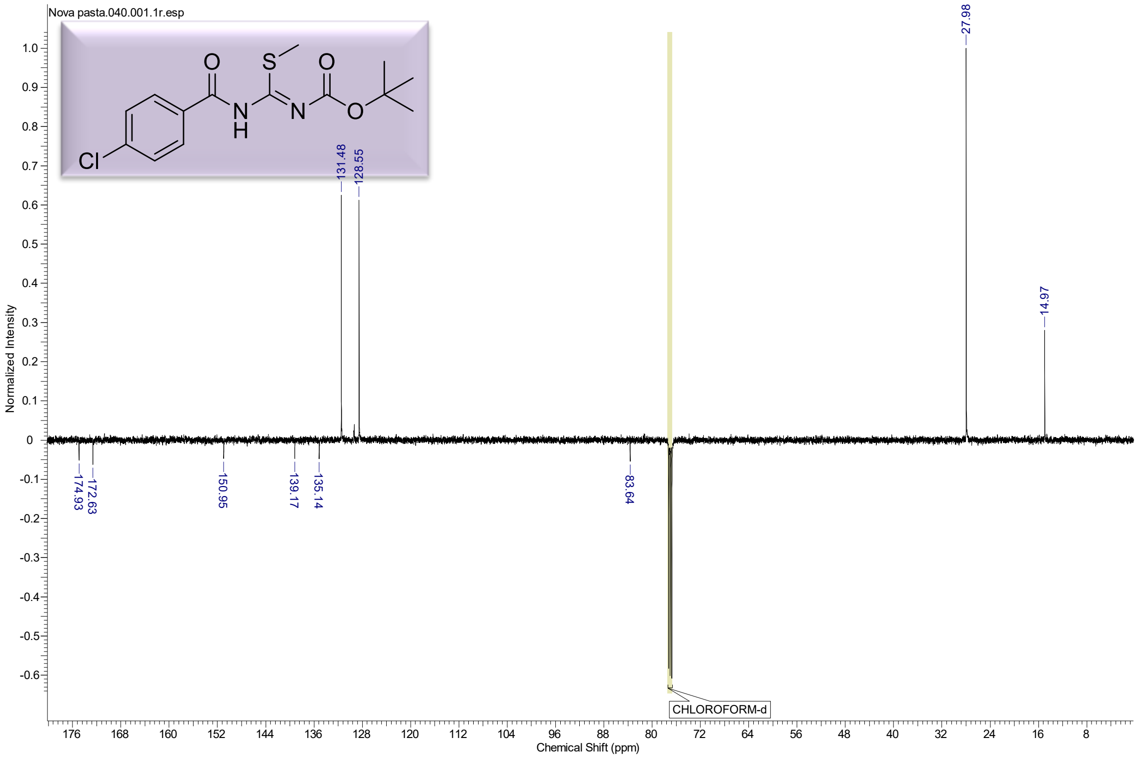

Figure S8: ^{1}H NMR (500 MHz) and ^{13}C NMR (125 MHz) spectra of compound **15f** in $CDCl_3$.

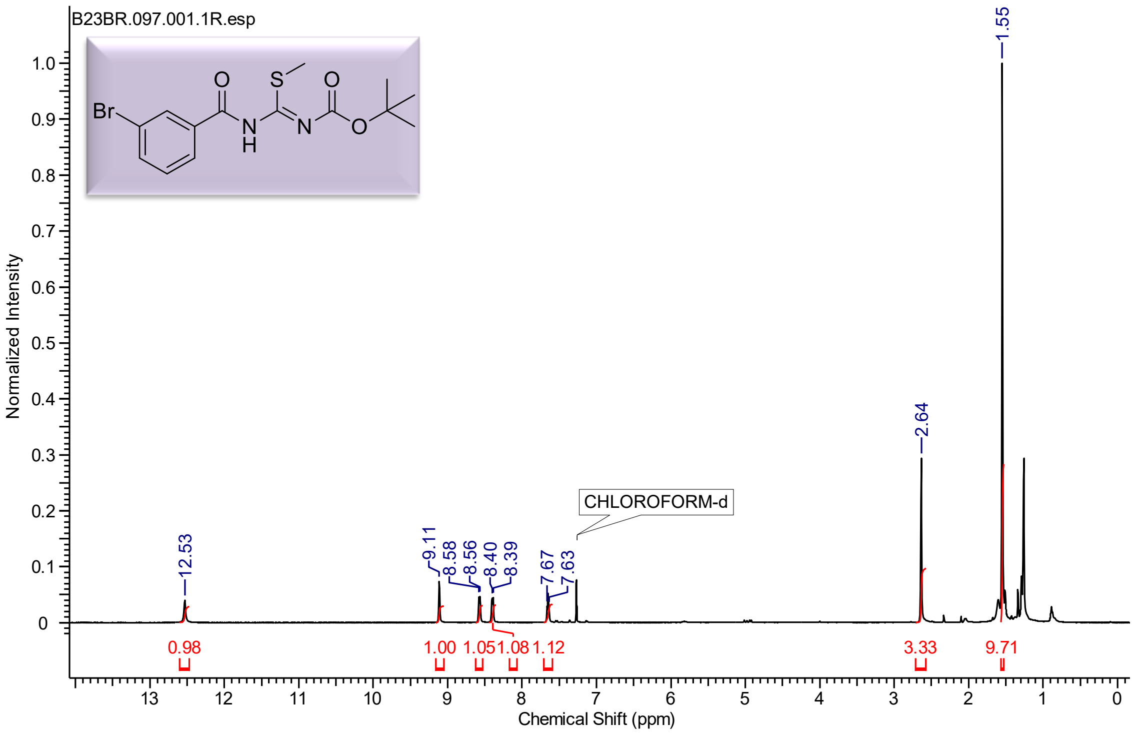

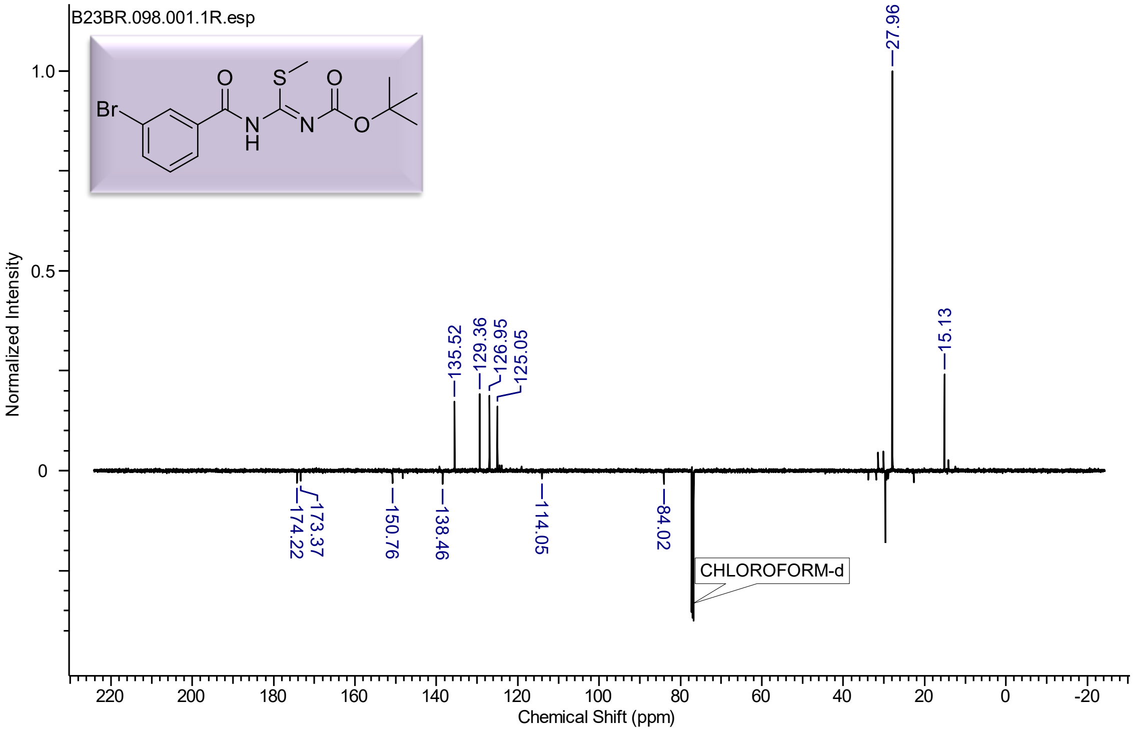

Figure S9: ^{1}H NMR (500 MHz) and ^{13}C NMR (125 MHz) spectra of compound **15g** in $CDCl_3$.

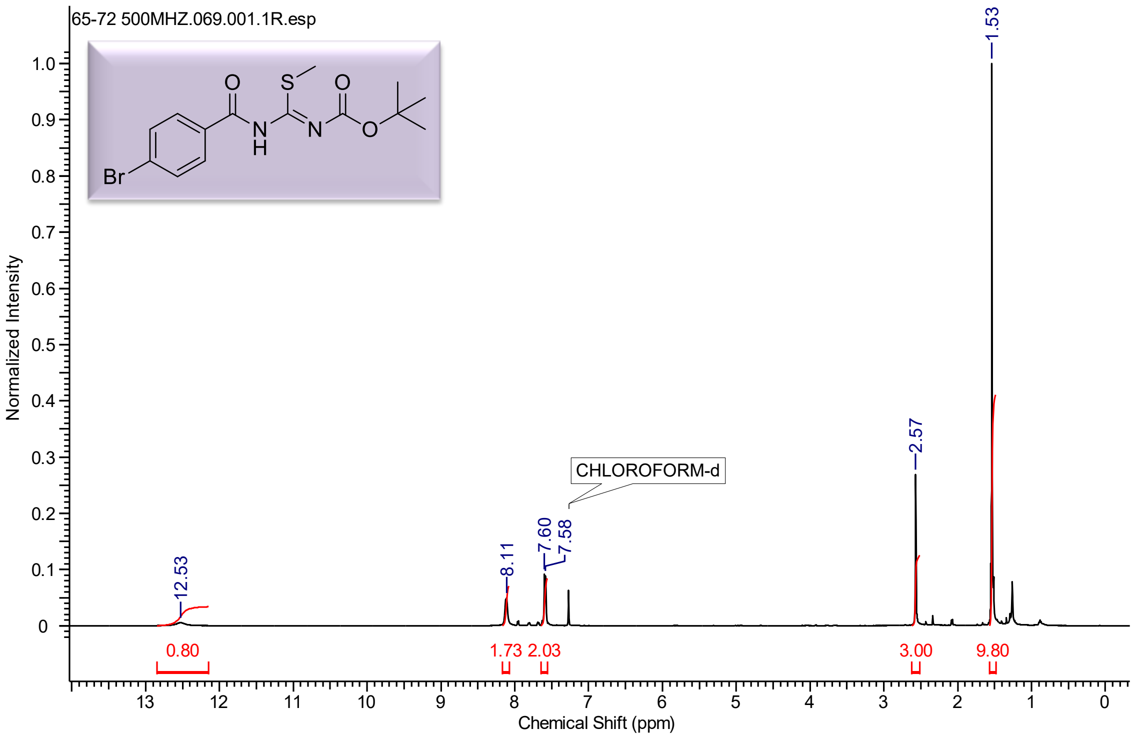

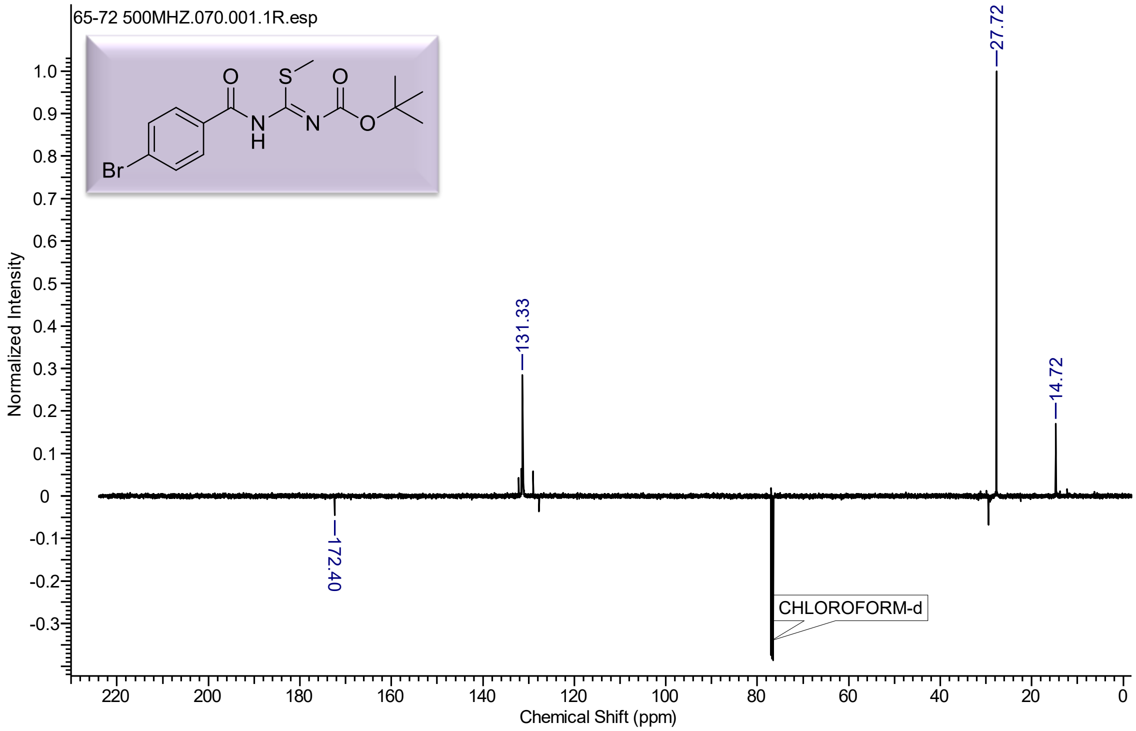

Figure S10: ^{1}H NMR (500 MHz) and ^{13}C NMR (125 MHz) spectra of compound **15h** in $CDCl_3$.

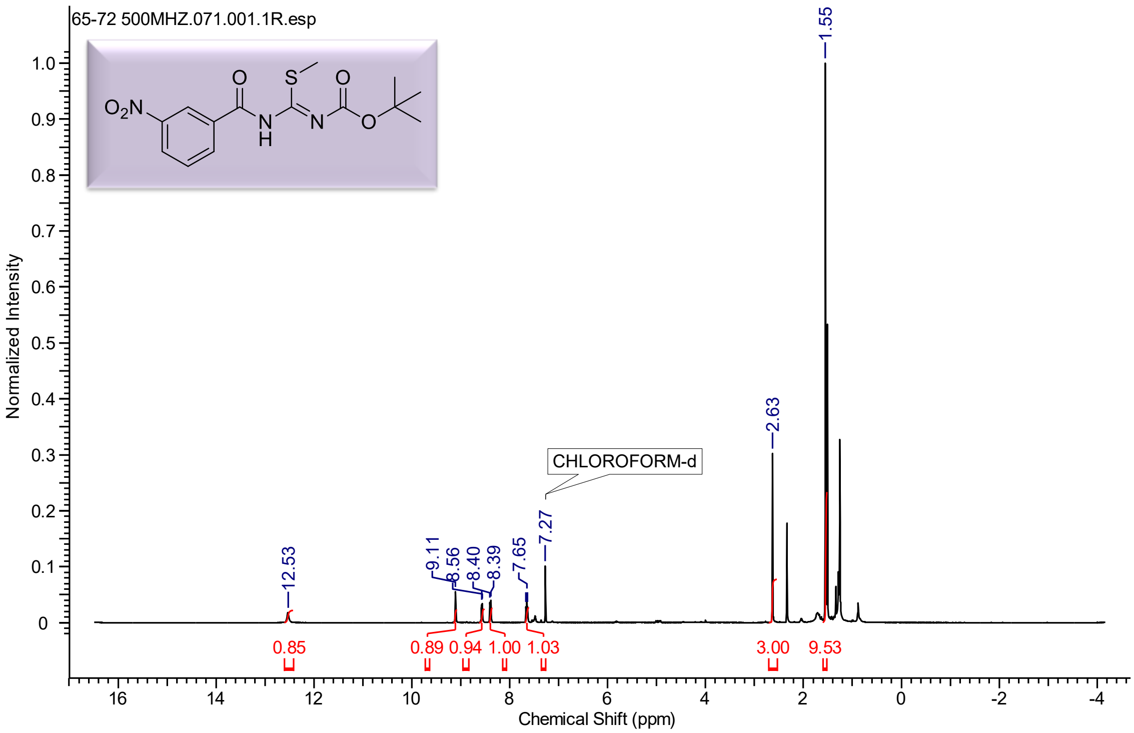

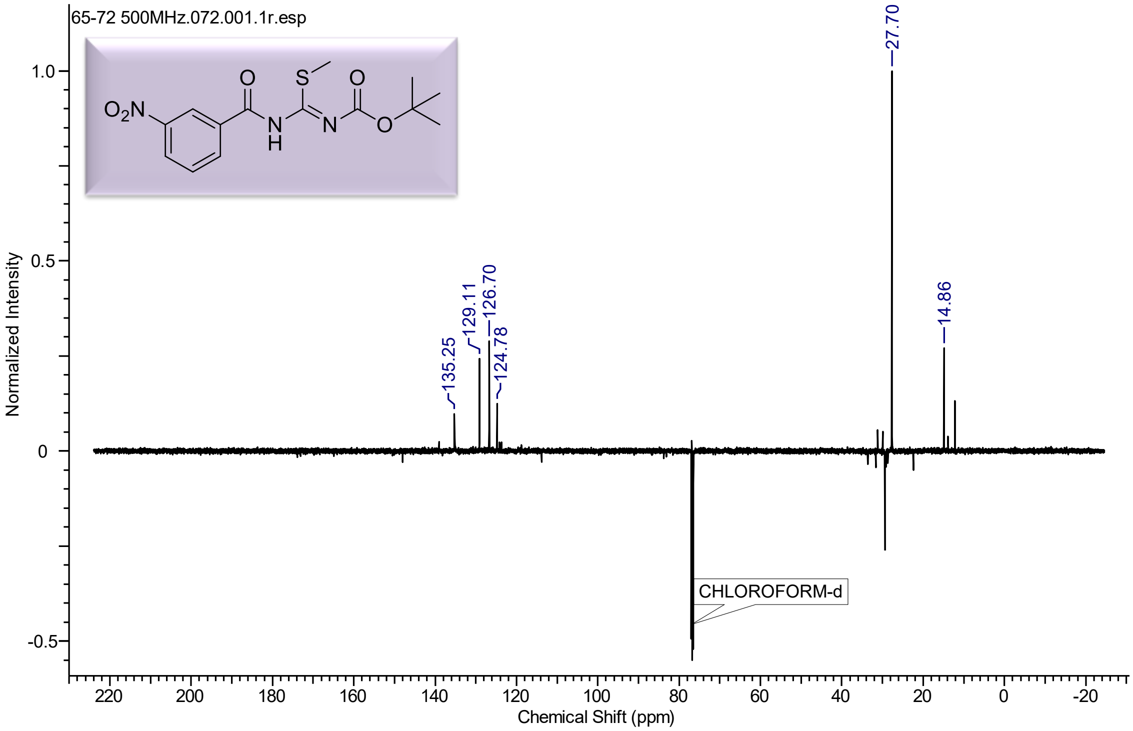

Figure S11: ^{1}H NMR (500 MHz) and ^{13}C NMR (125 MHz) spectra of compound **15i** in $CDCl_3$.

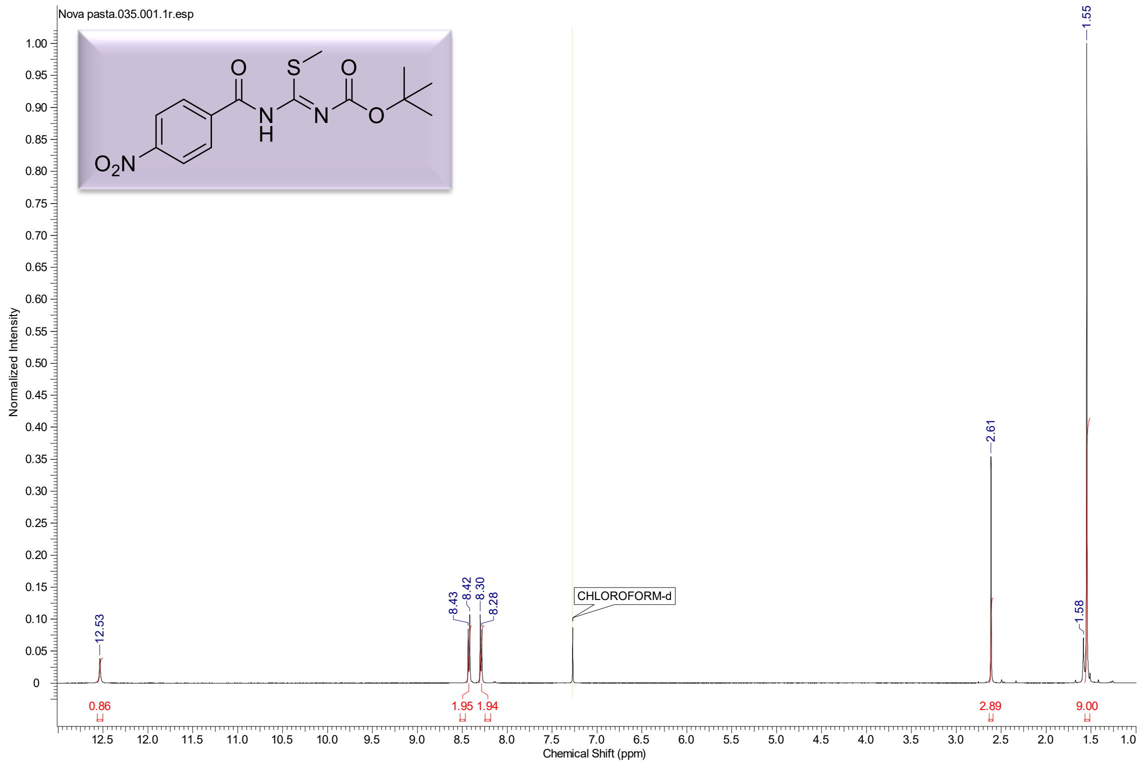

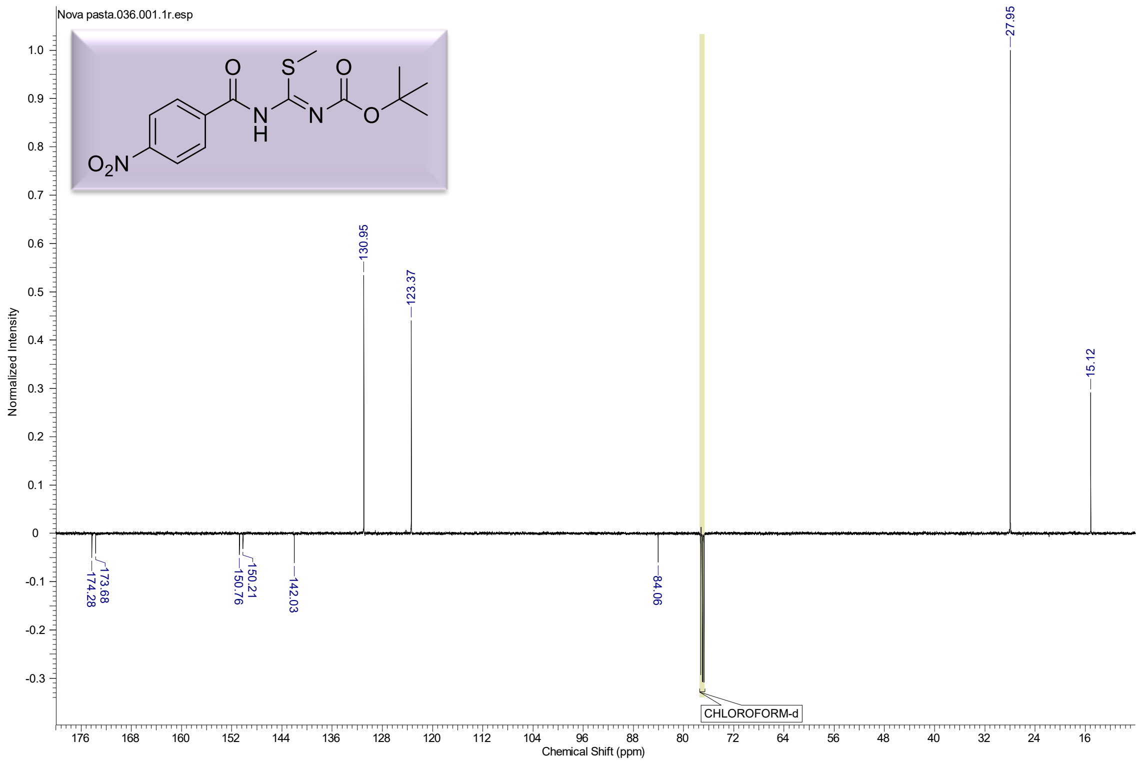

Figure S12: ^{1}H NMR (500 MHz) and ^{13}C NMR (125 MHz) spectra of compound **15j** in $CDCl_3$.

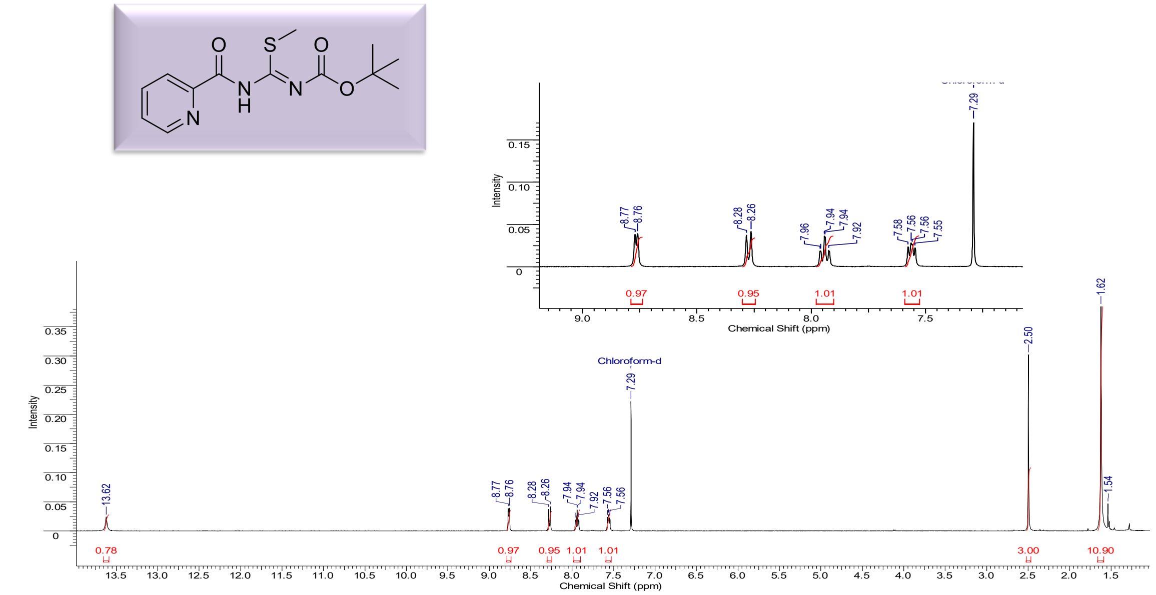

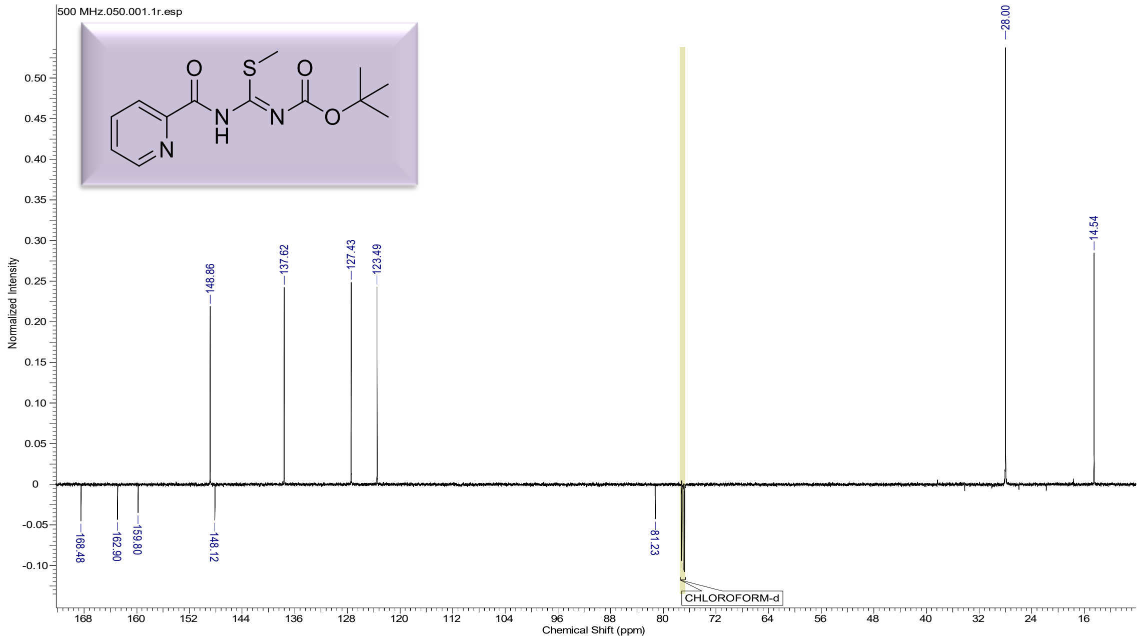

Figure S13: ^{1}H NMR (500 MHz) and ^{13}C NMR (125 MHz) spectra of compound **15k** in $CDCl_3$.

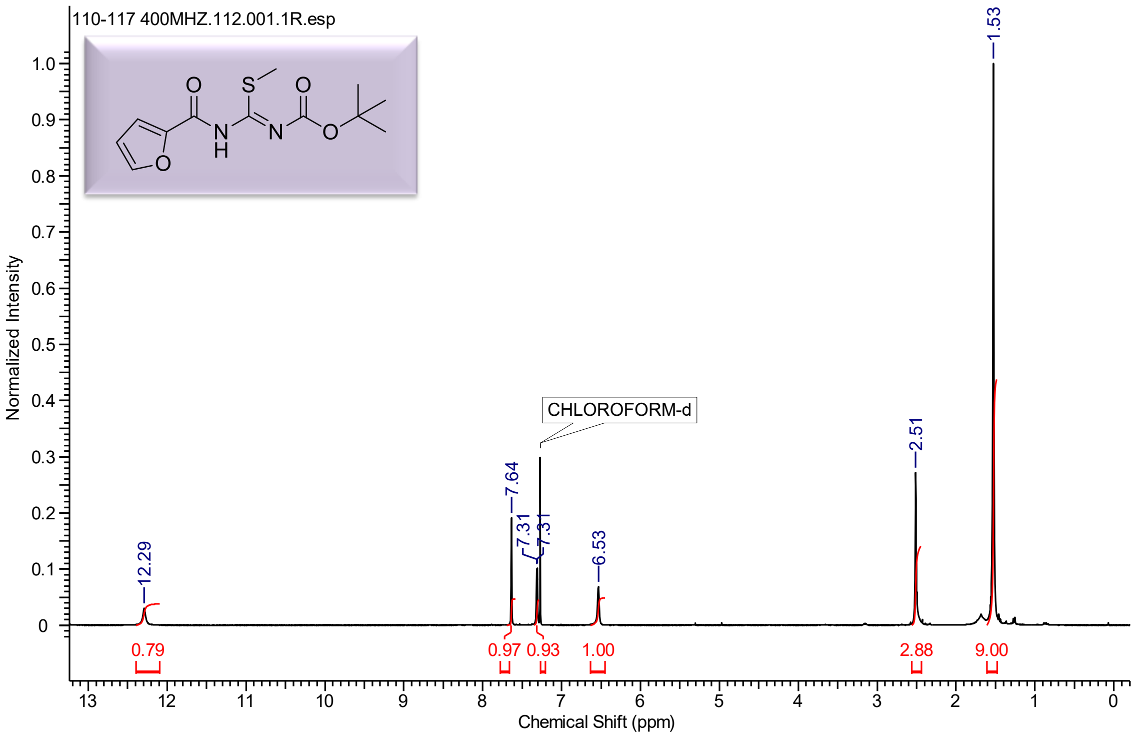

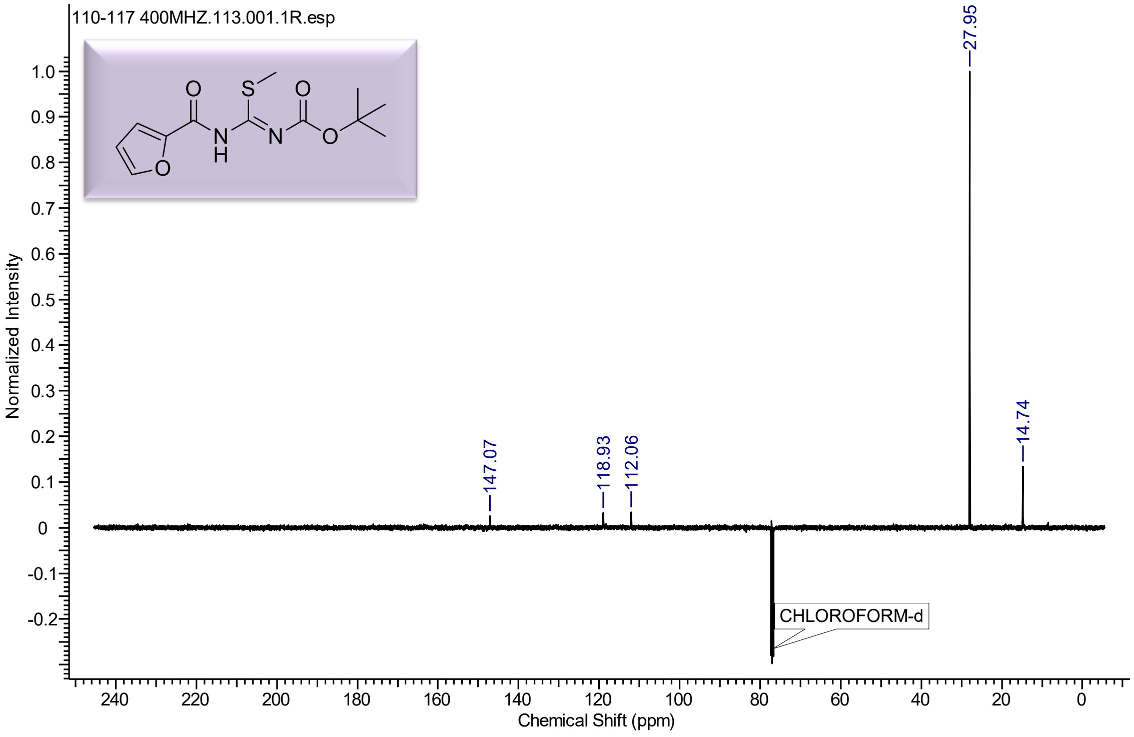

Figure S14: ^{1}H NMR (500 MHz) and ^{13}C NMR (125 MHz) spectra of compound **15l** in $CDCl_3$.

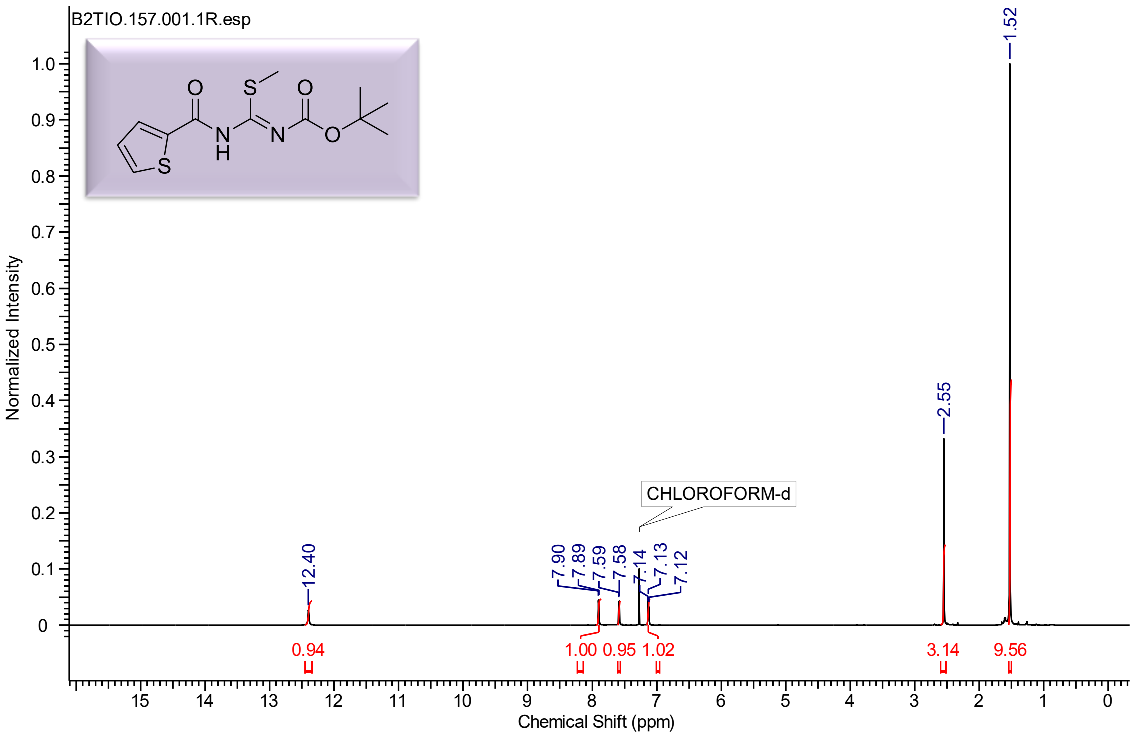

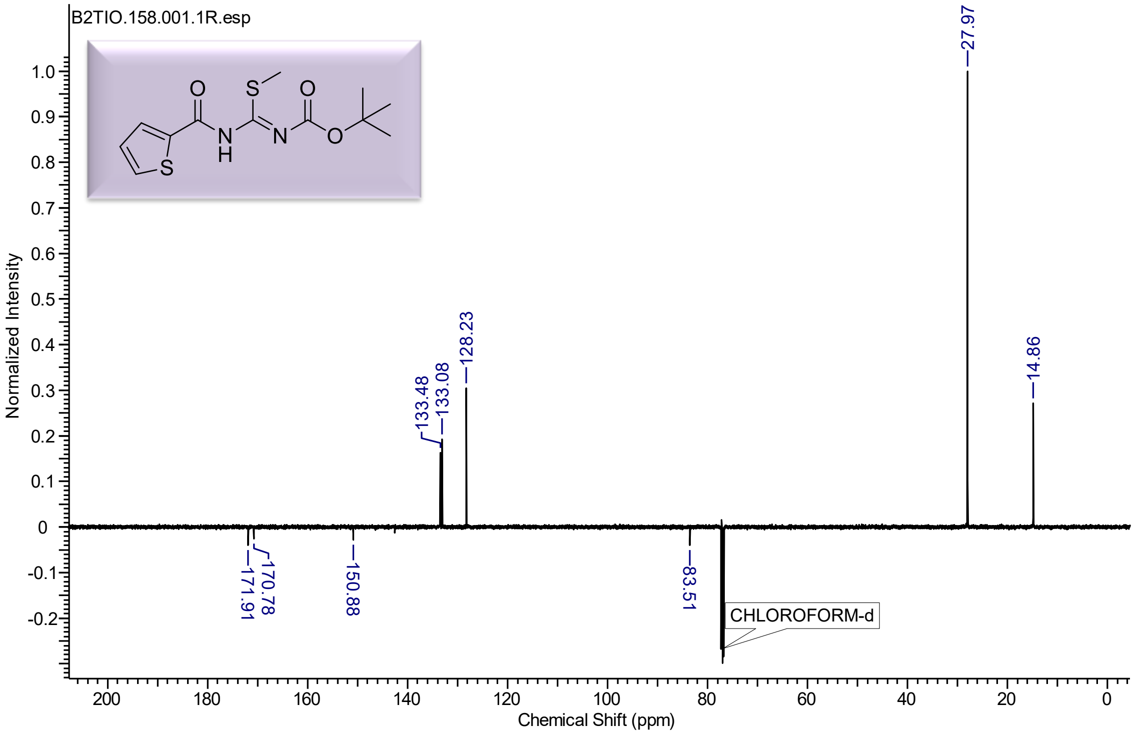

Figure S15: ^{1}H NMR (500 MHz) and ^{13}C NMR (125 MHz) spectra of compound **15m** in $CDCl_3$.

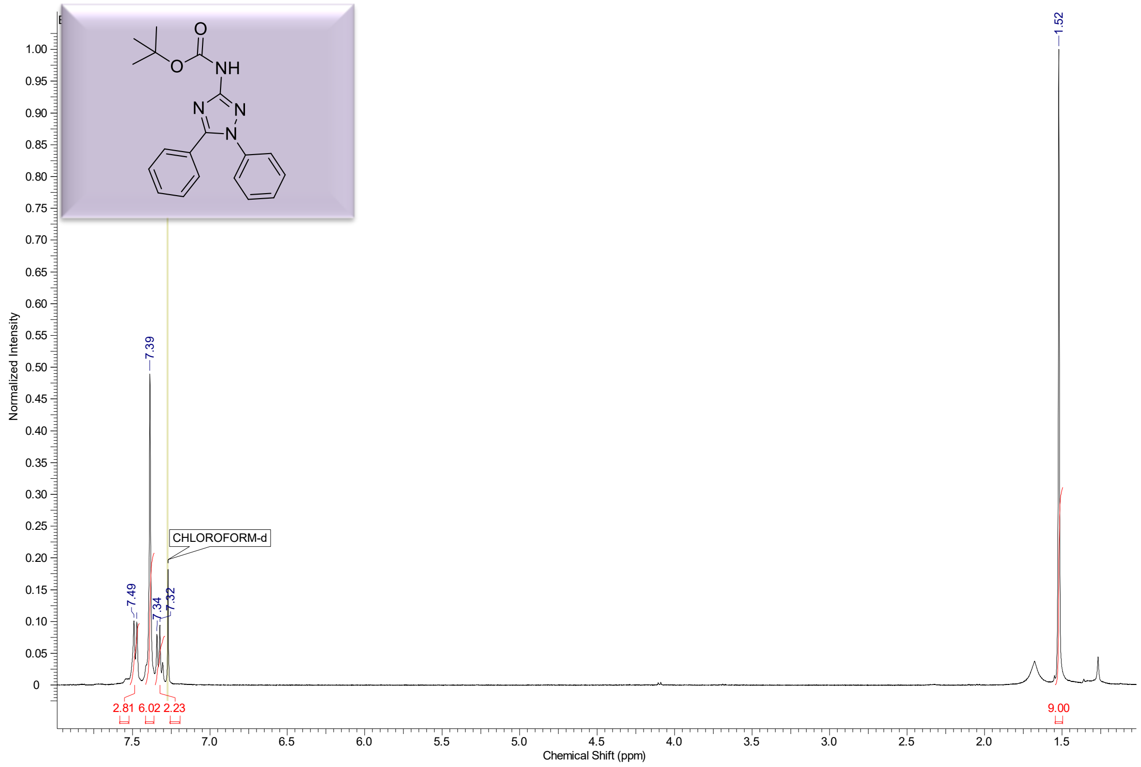

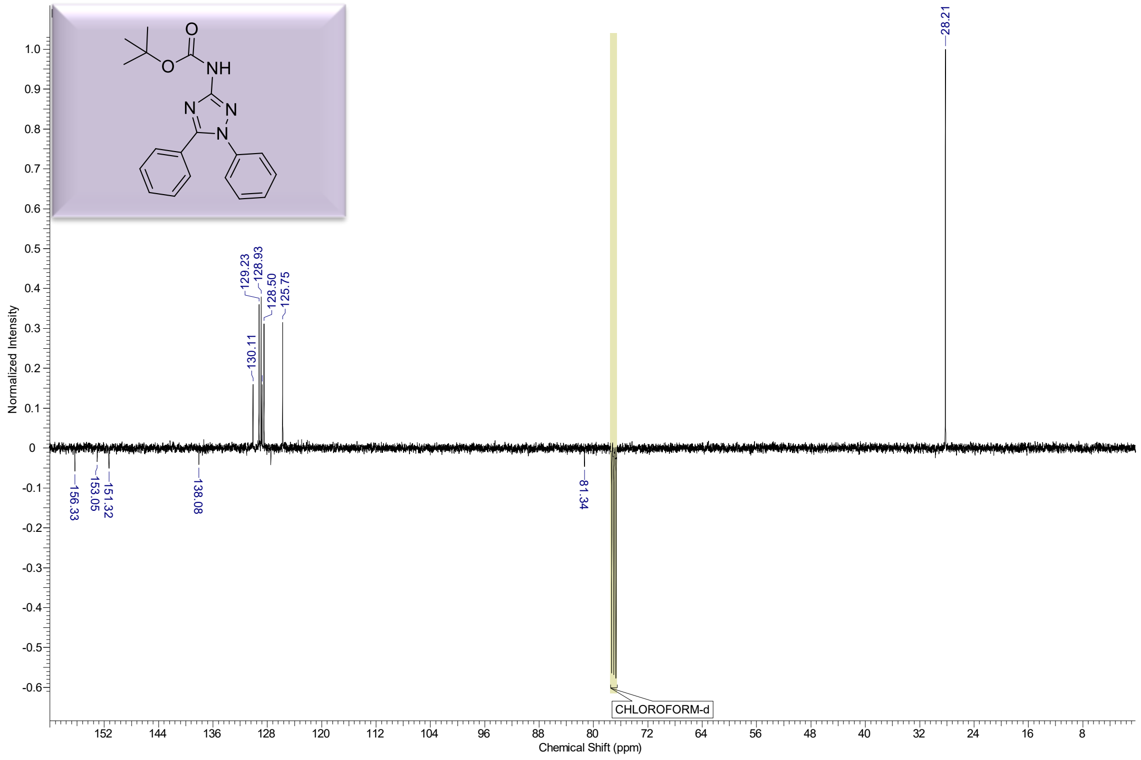

Figure S16: ^{1}H NMR (400 MHz) and ^{13}C NMR (100 MHz) spectra of compound **16a** in $CDCl_3$.

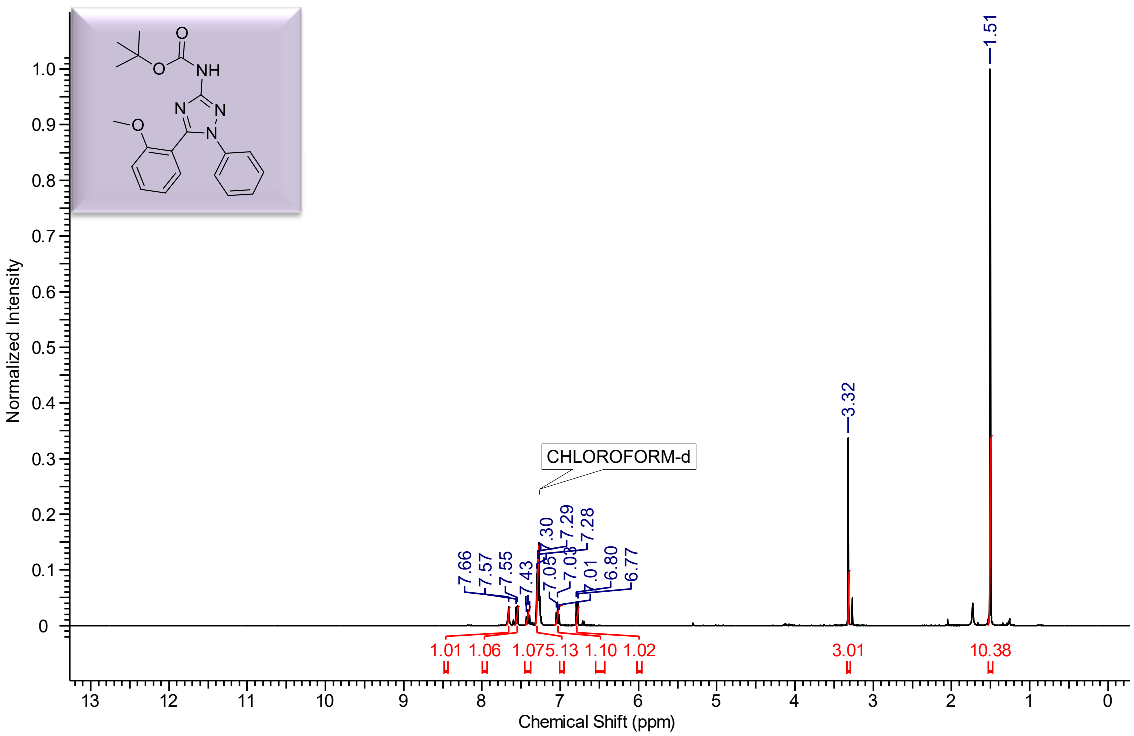

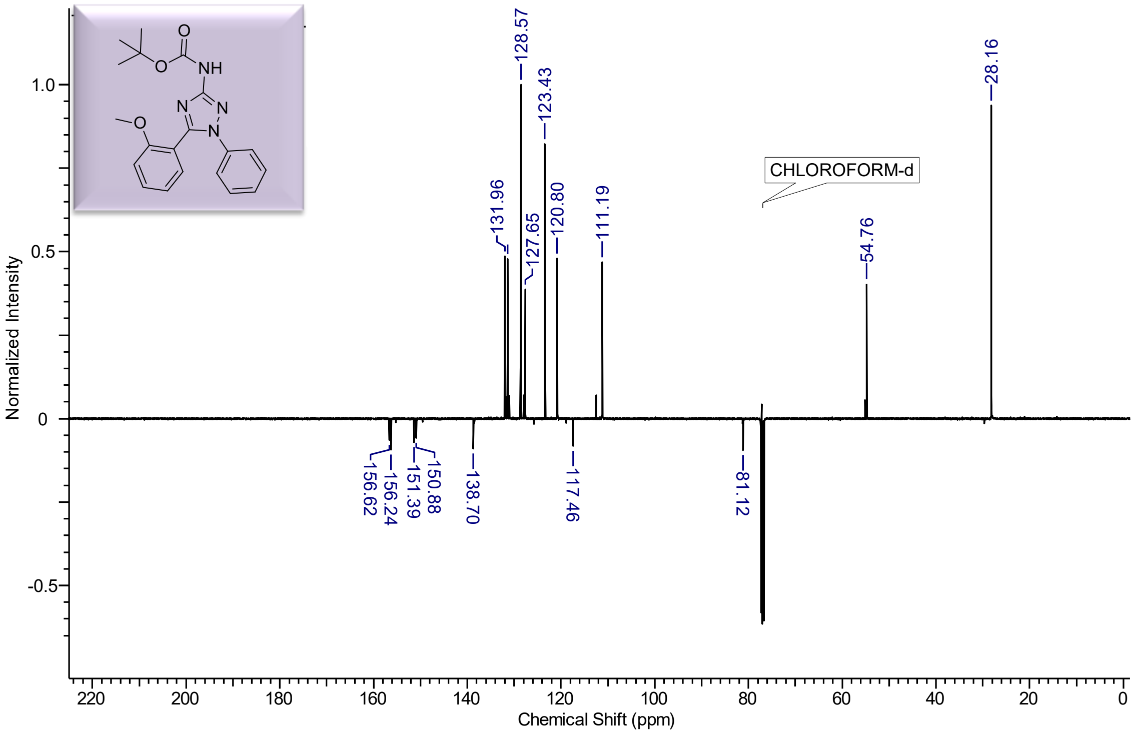

Figure S17: ^{1}H NMR (400 MHz) and ^{13}C NMR (100 MHz) spectra of compound **16d** in $CDCl_3$.

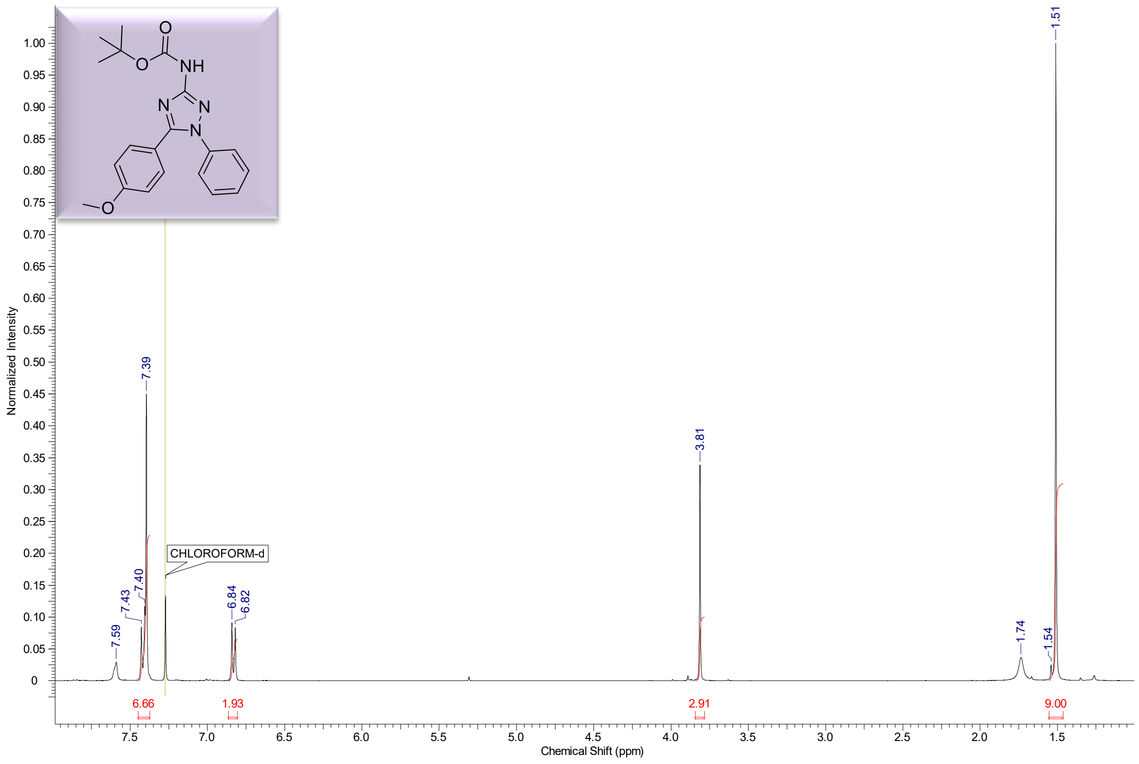

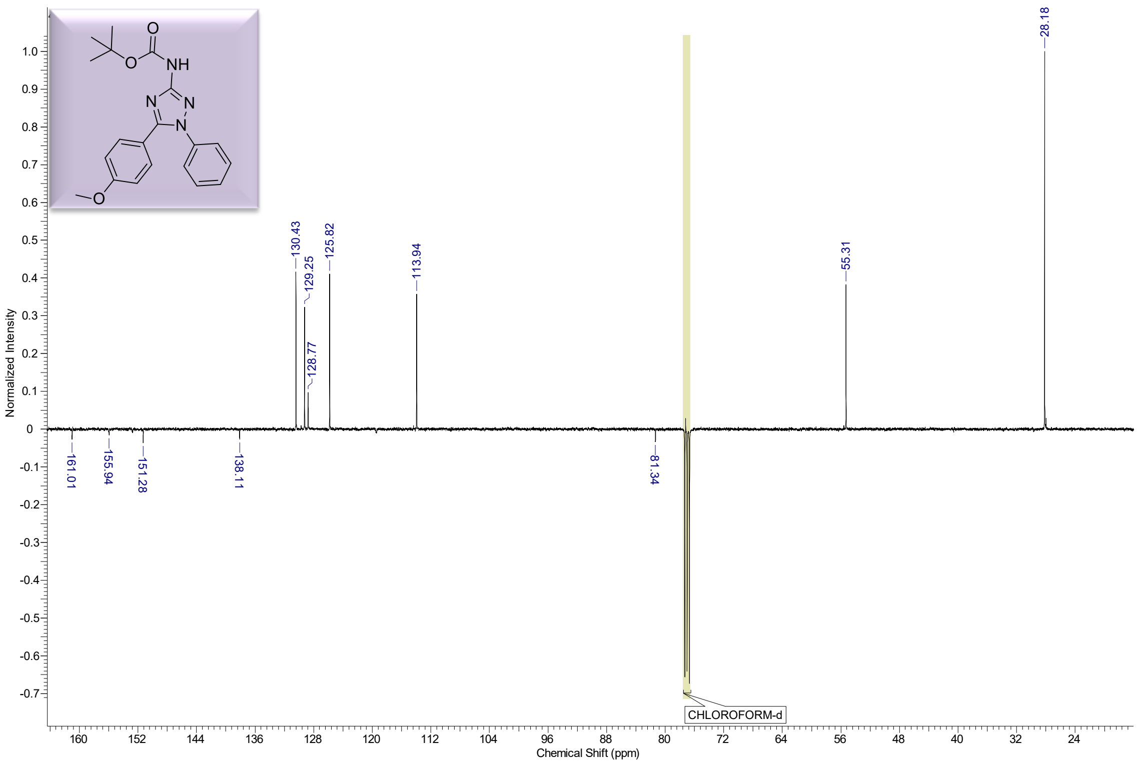

Figure S18: ^{1}H NMR (500 MHz) and ^{13}C NMR (125 MHz) spectra of compound **16e** in $CDCl_3$.

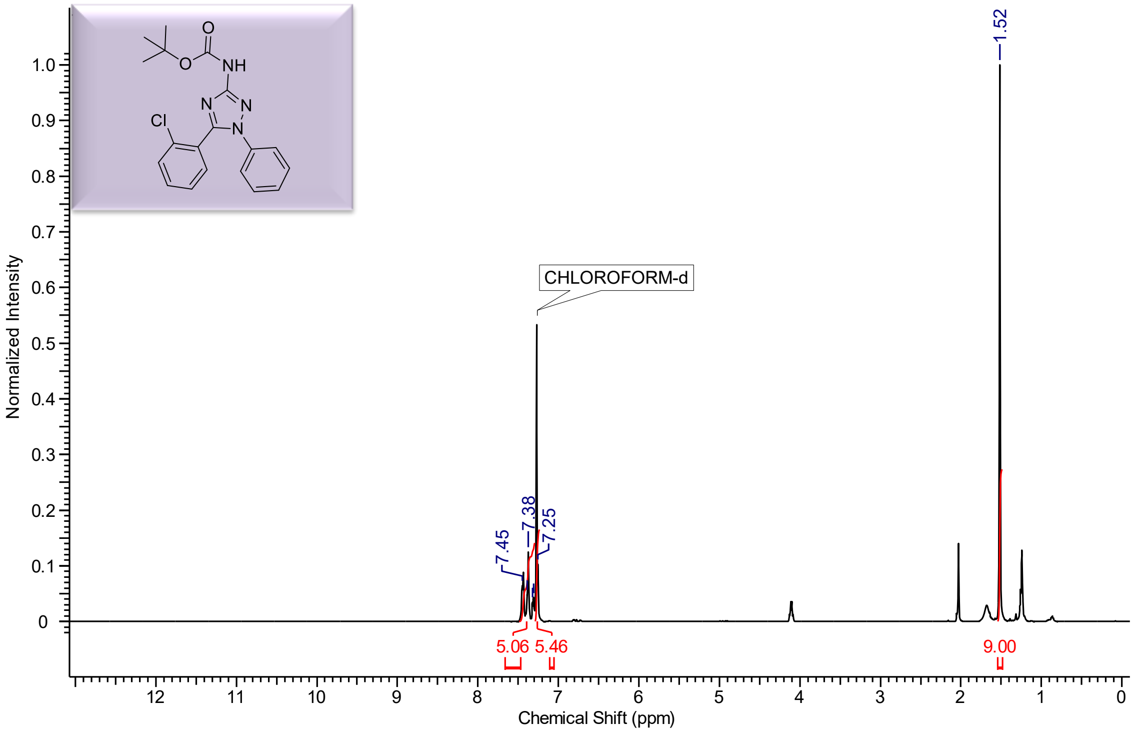

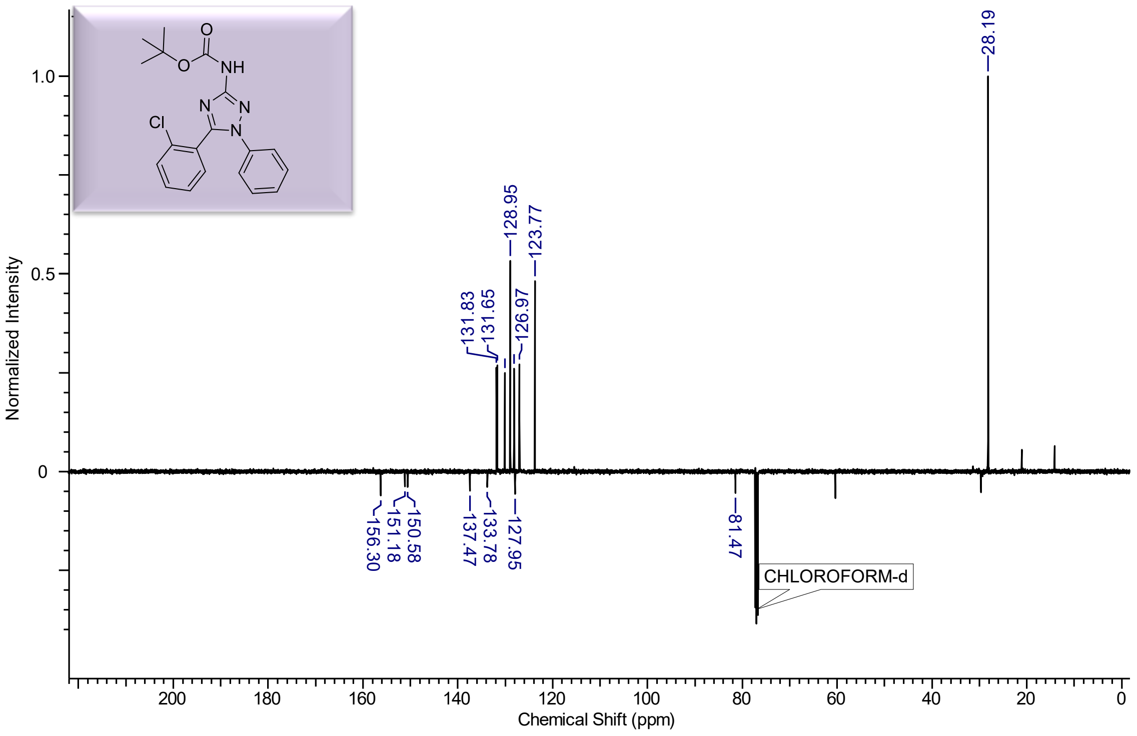

Figure S19: ^{1}H NMR (500 MHz) and ^{13}C NMR (125 MHz) spectra of compound **16h** in $CDCl_3$.

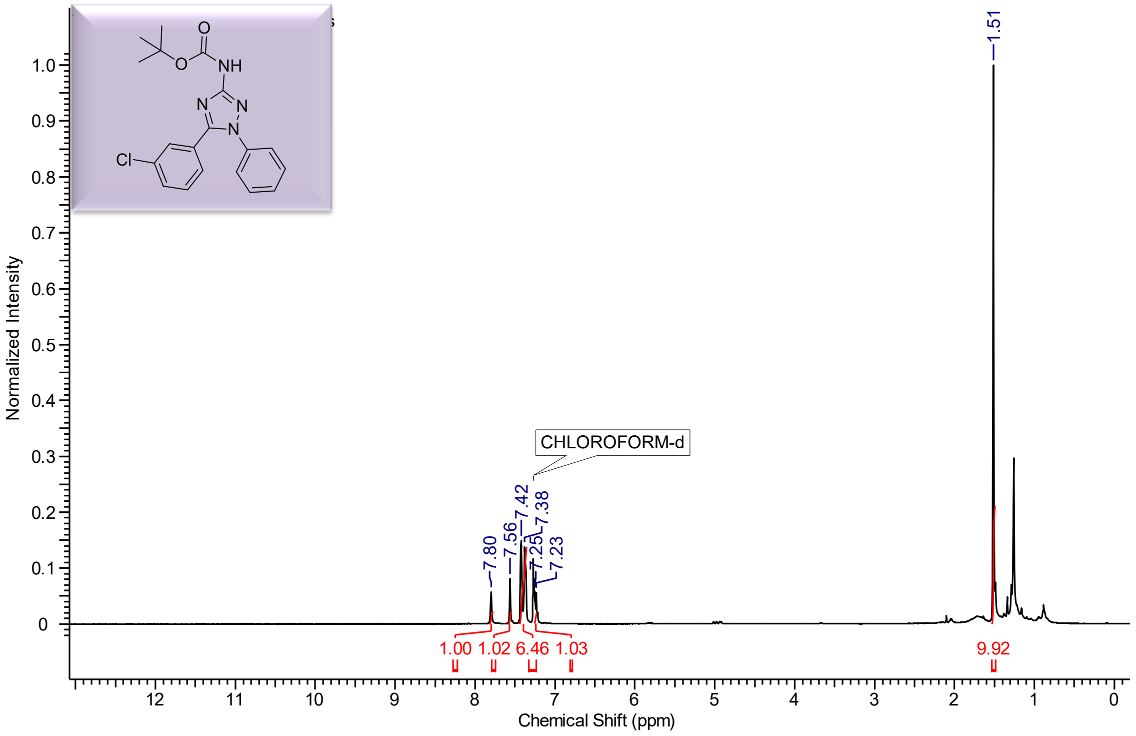

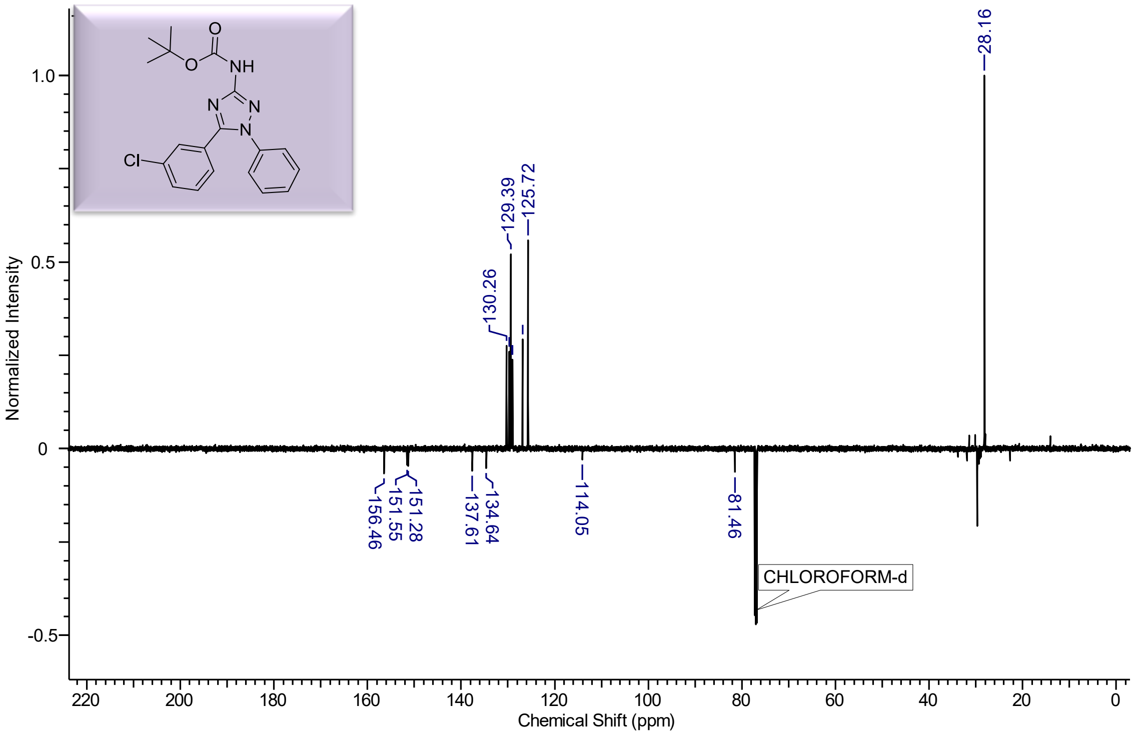

Figure S20: ^{1}H NMR (500 MHz) and ^{13}C NMR (125 MHz) spectra of compound **6-16i** in $CDCl_3$.

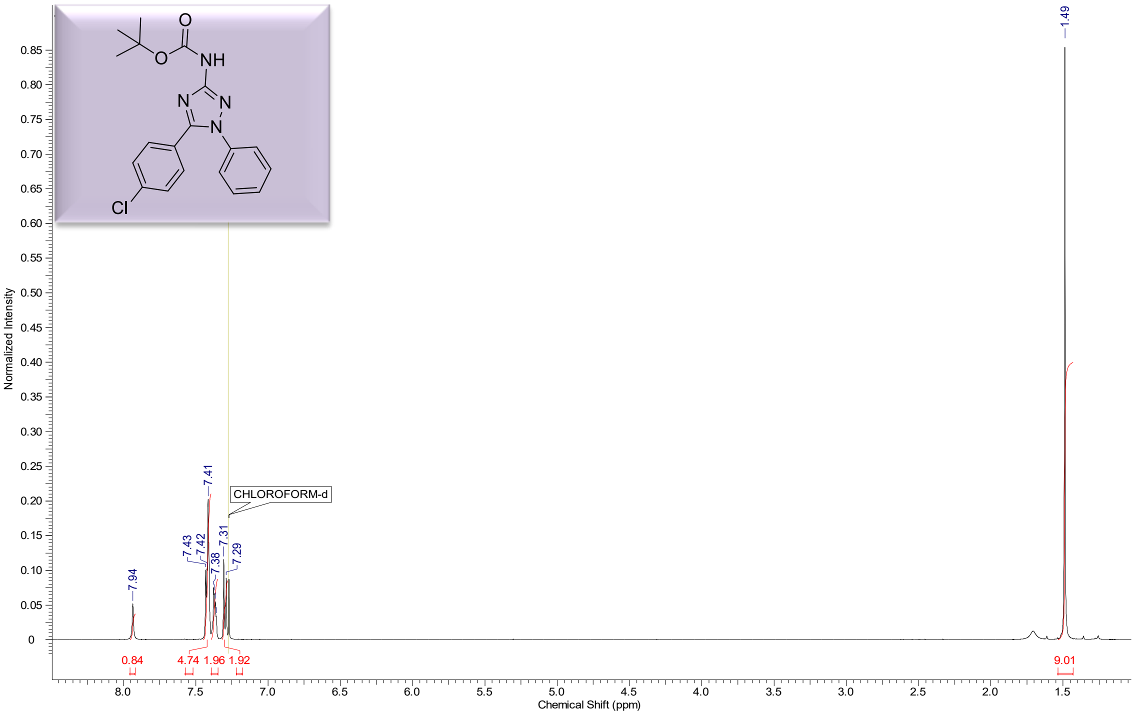

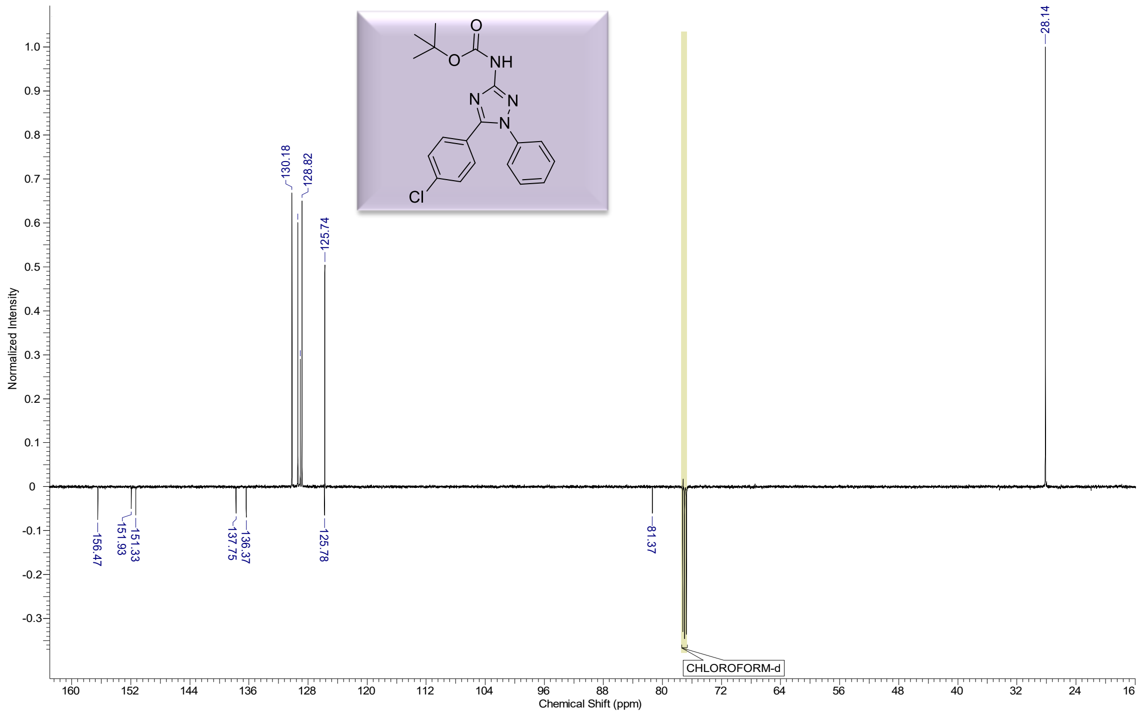

Figure S21: ^{1}H NMR (400 MHz) and ^{13}C NMR (100 MHz) spectra of compound **16j** in $CDCl_3$.

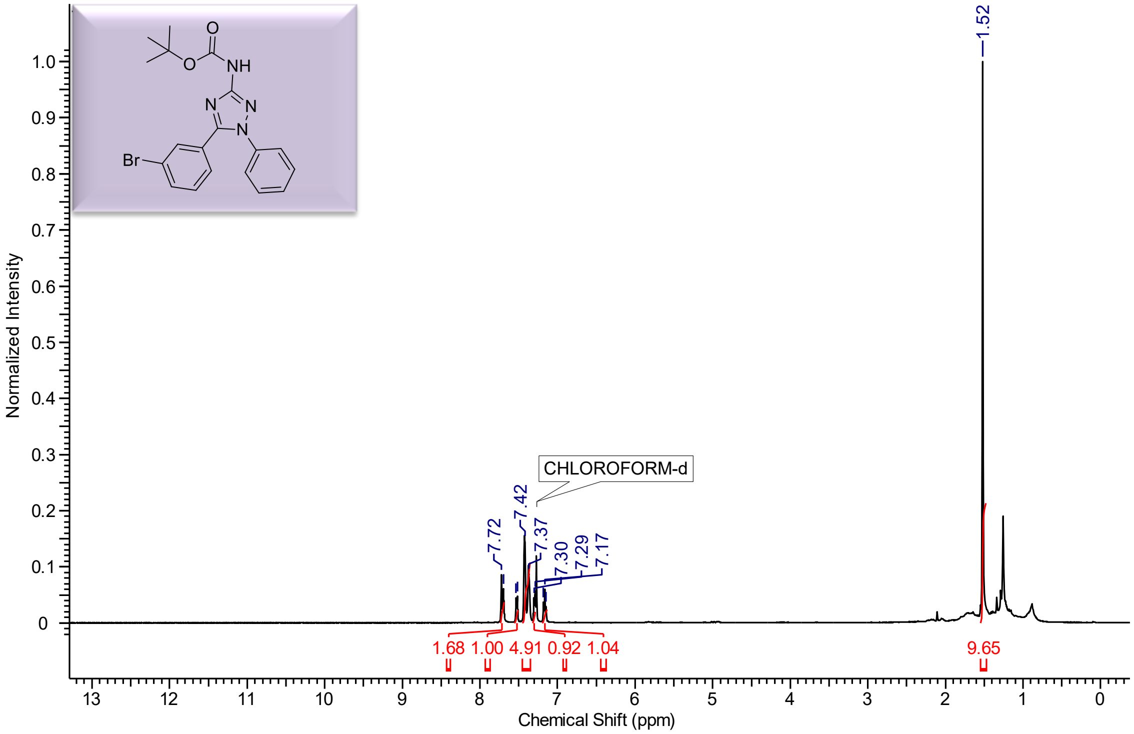

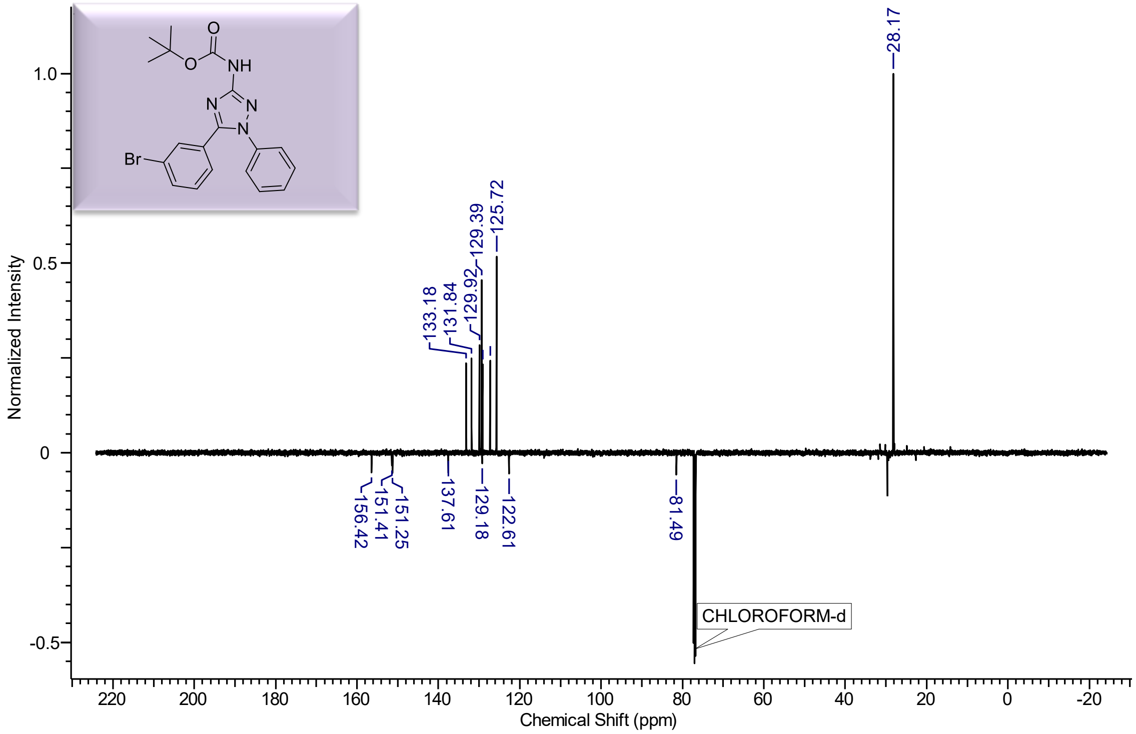

Figure S22: ^{1}H NMR (500 MHz) and ^{13}C NMR (125 MHz) spectra of compound **16k** in $CDCl_3$.

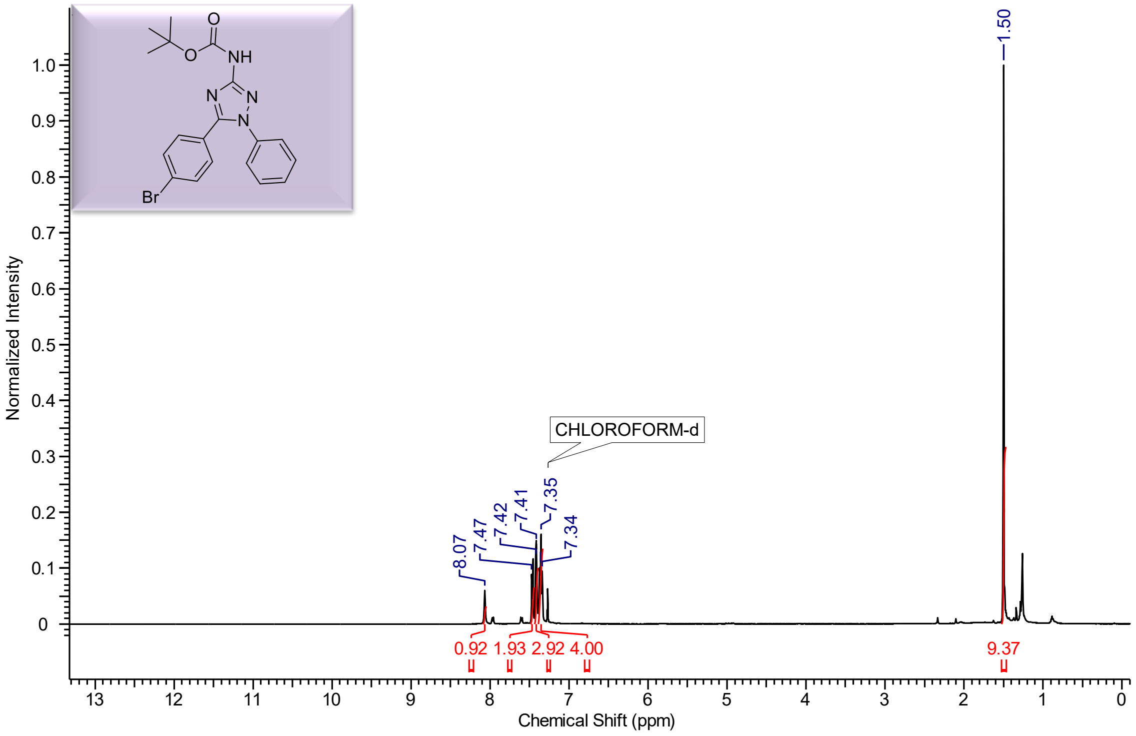

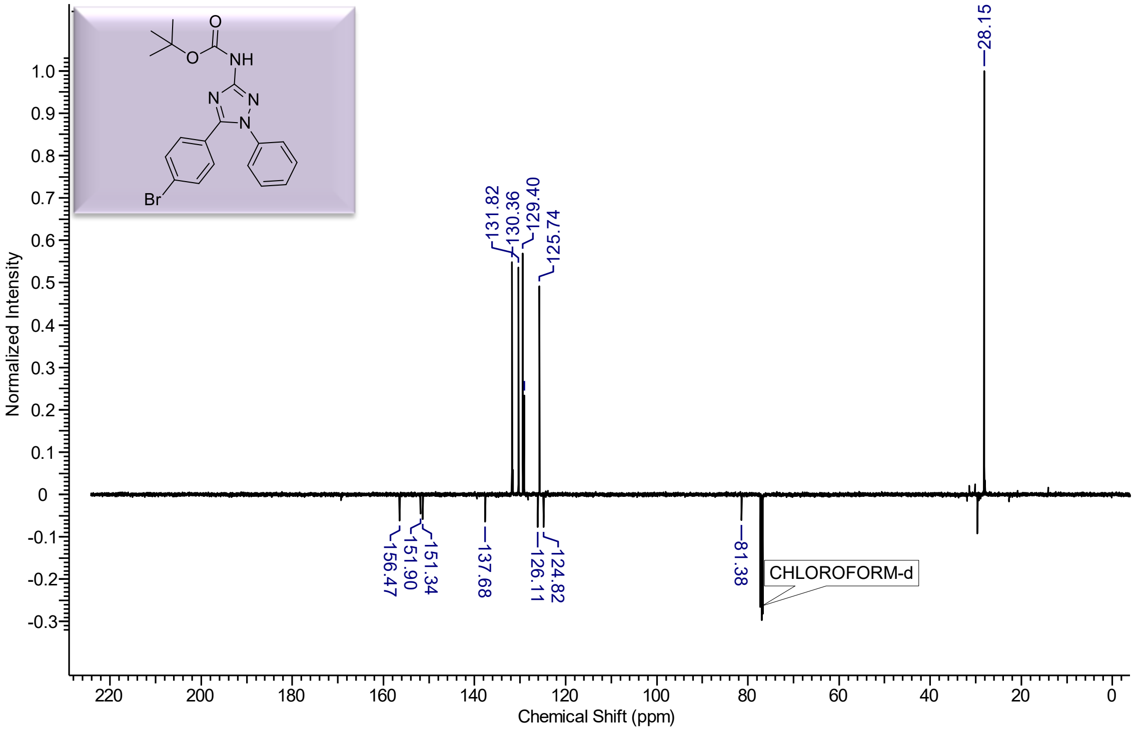

Figure S23: ^{1}H NMR (500 MHz) and ^{13}C NMR (125 MHz) spectra of compound **16l** in $CDCl_3$.

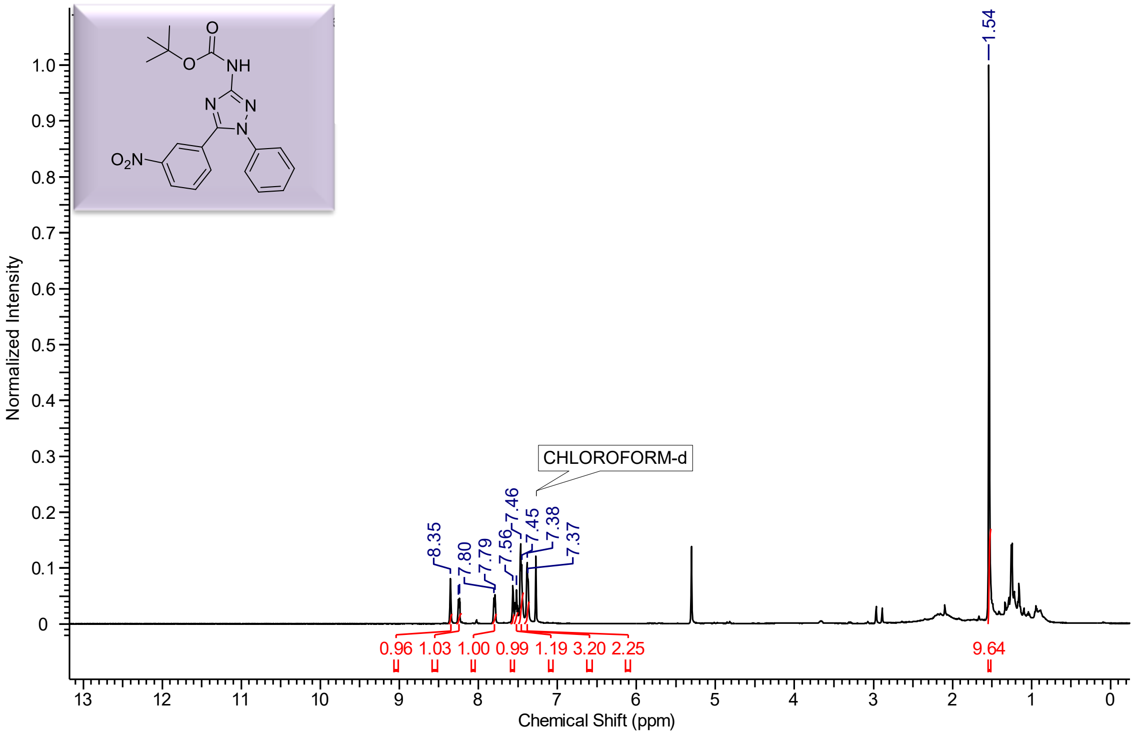

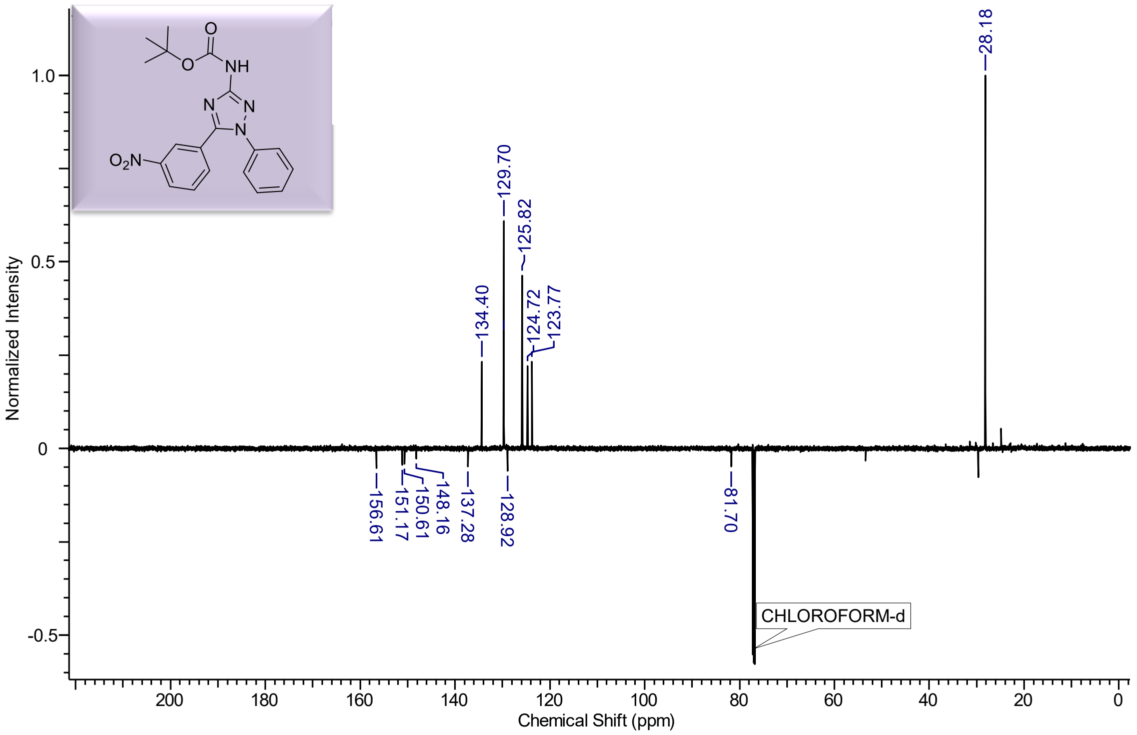

Figure S24: ^{1}H NMR (500 MHz) and ^{13}C NMR (125 MHz) spectra of compound **16m** in $CDCl_3$.

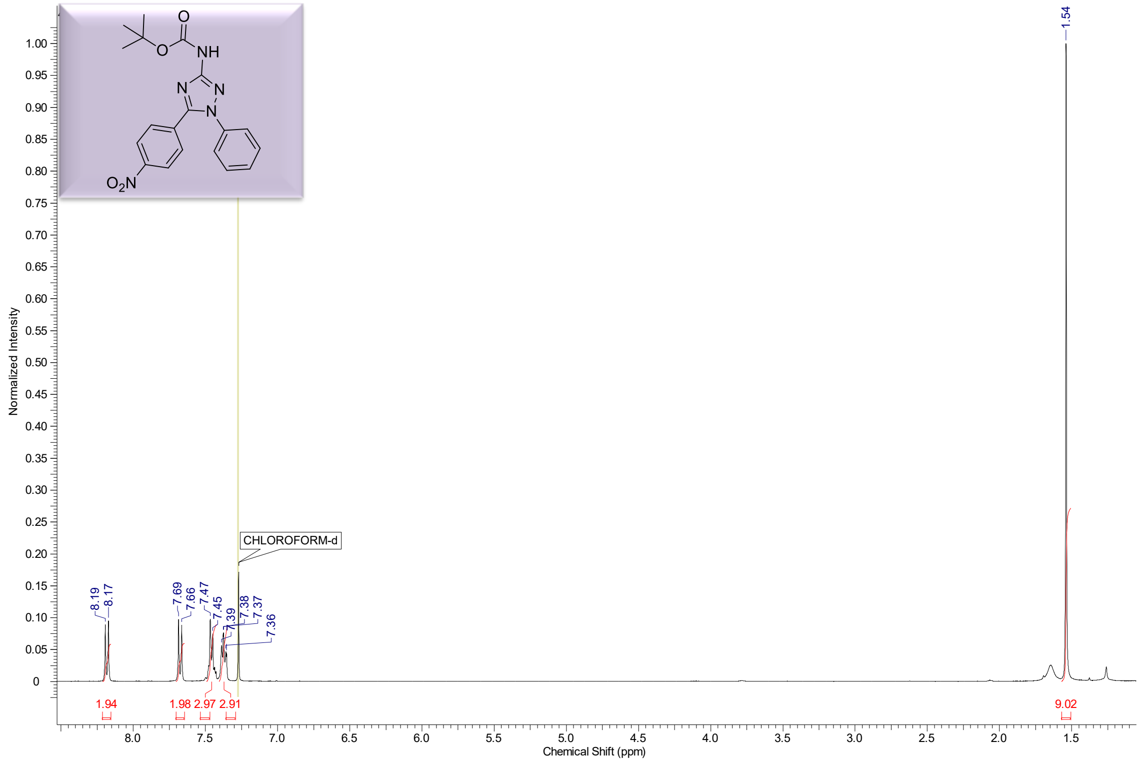

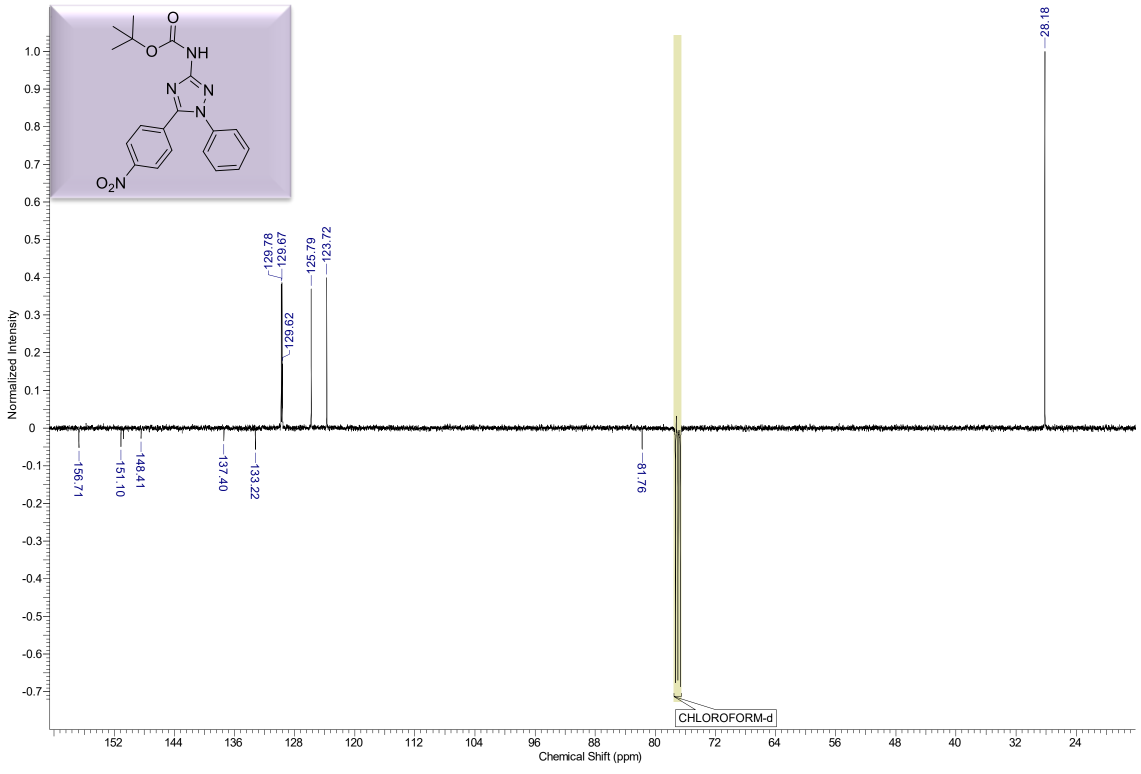

Figure S25: ^{1}H NMR (400 MHz) and ^{13}C NMR (100 MHz) spectra of compound **16n** in $CDCl_3$.

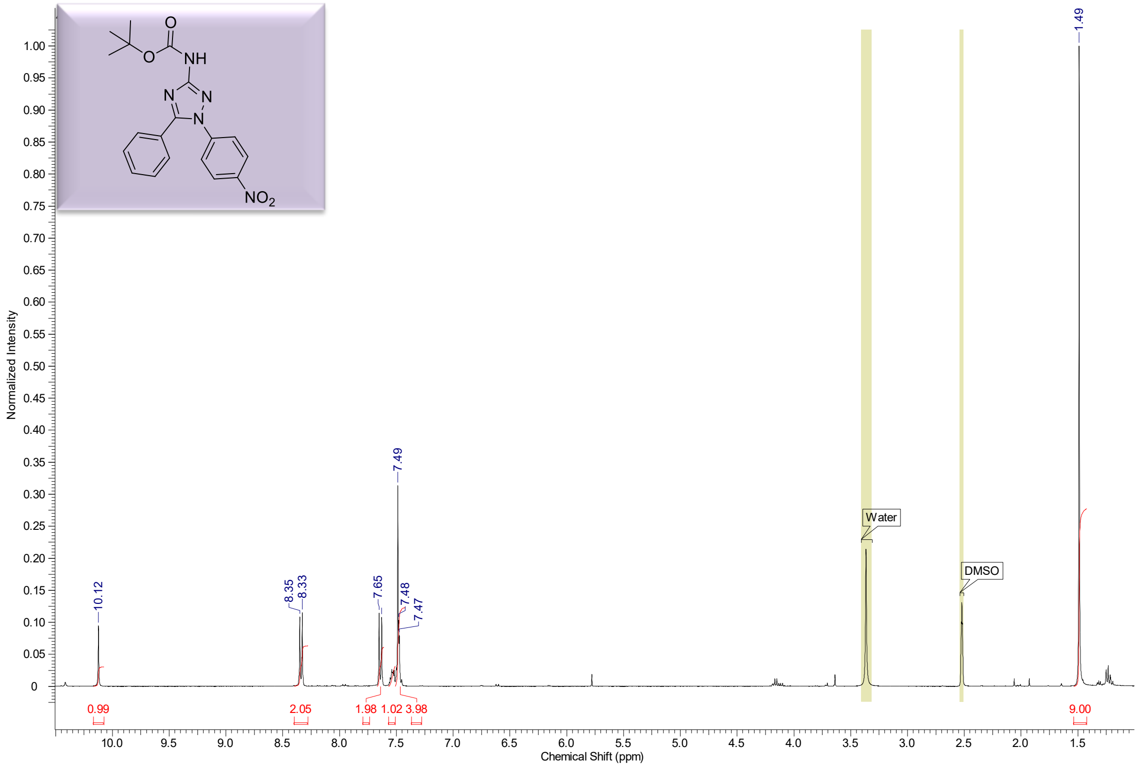

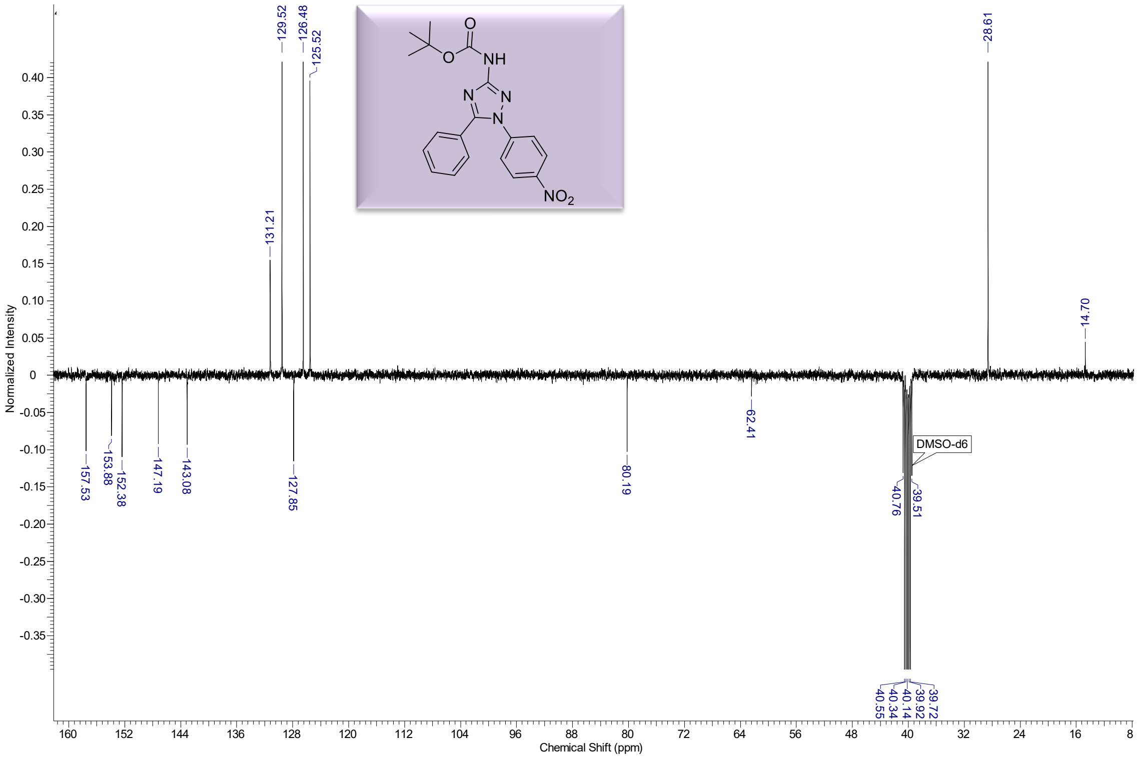

Figure S26: ^{1}H NMR (400 MHz) and ^{13}C NMR (100 MHz) spectra of compound **16o** in DMSO-d_6.

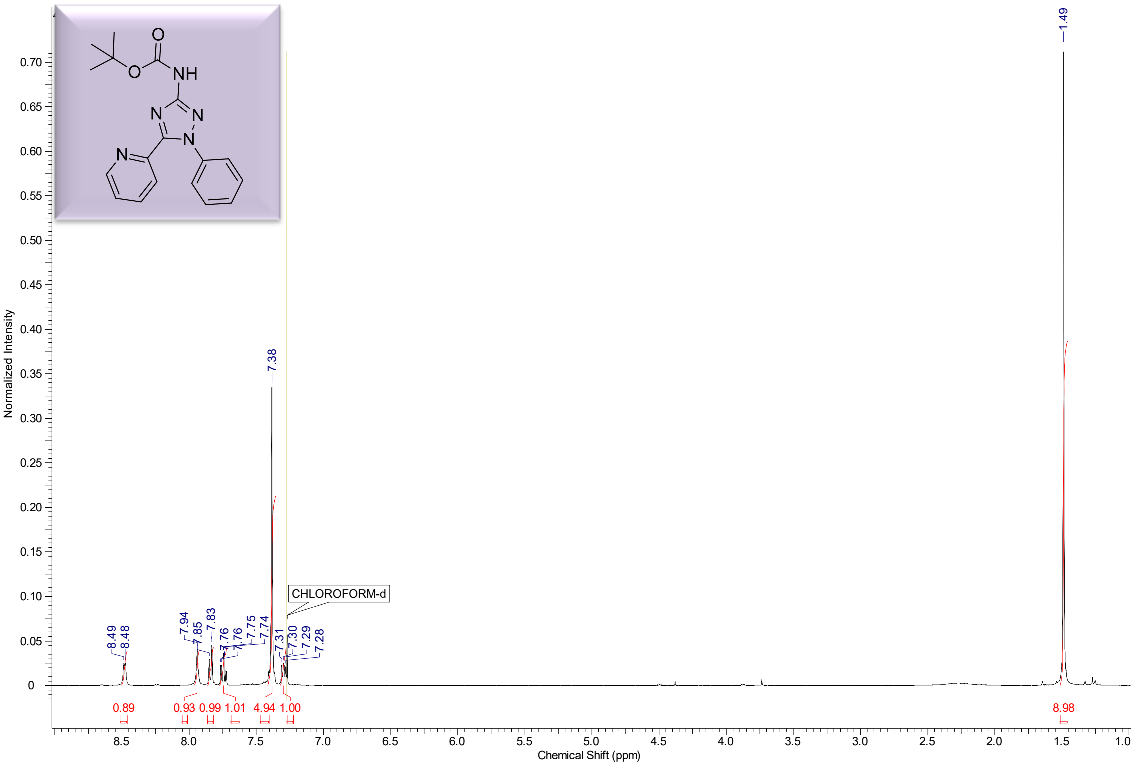

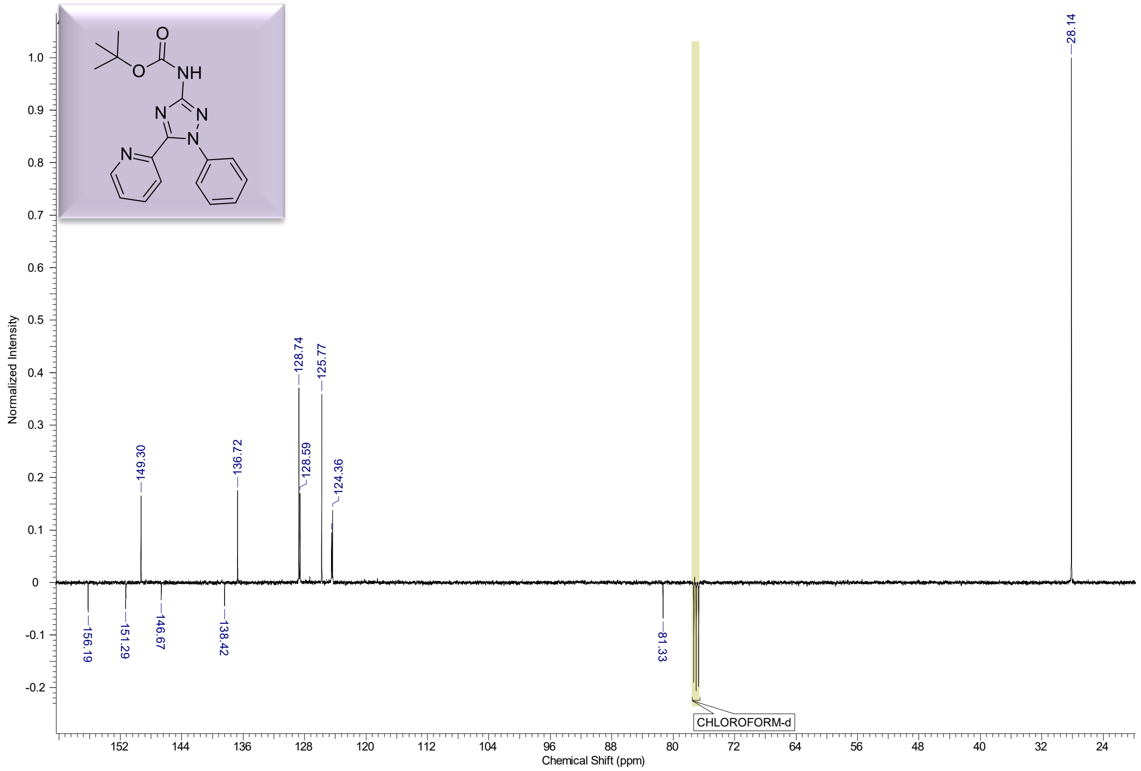

Figure S27: ^{1}H NMR (500 MHz) and ^{13}C NMR (125 MHz) spectra of compound **16p** in $CDCl_3$.

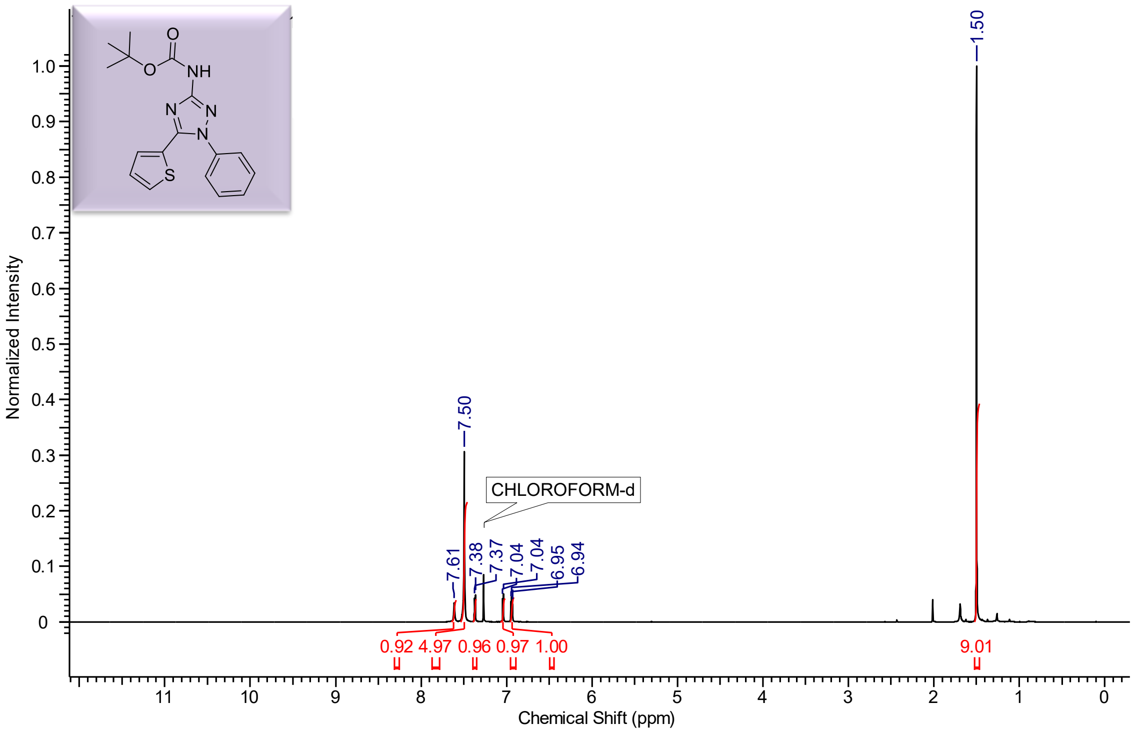

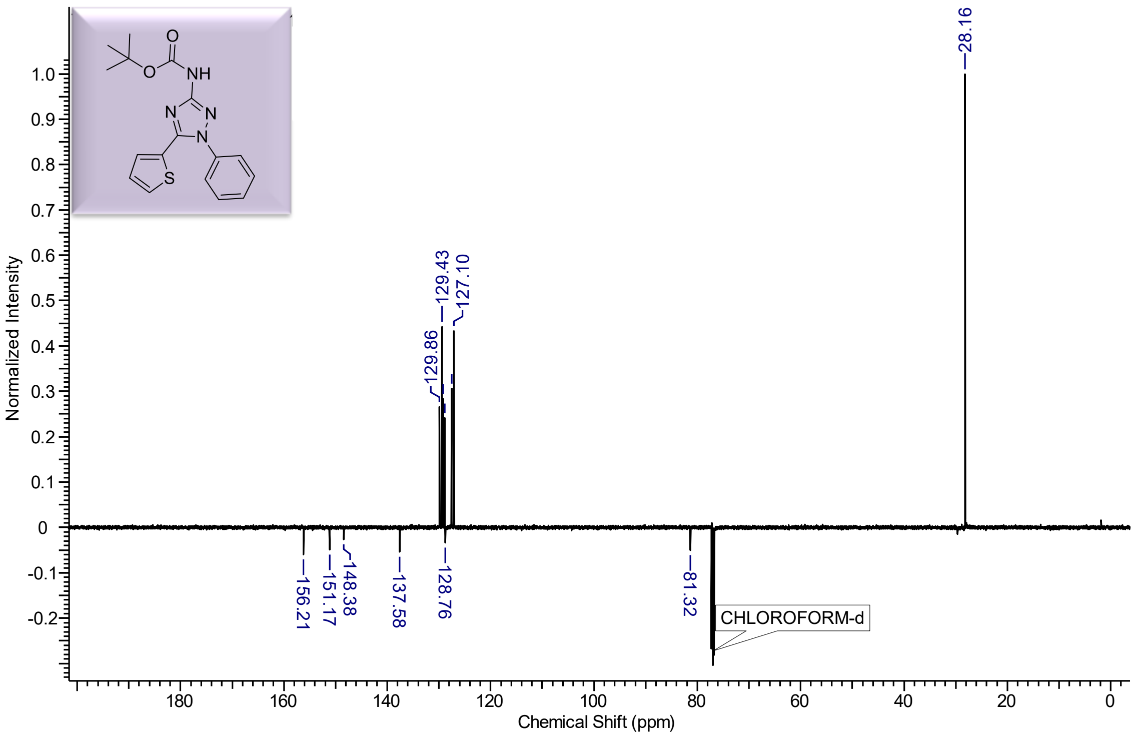

Figure S28: ^{1}H NMR (500 MHz) and ^{13}C NMR (125 MHz) spectra of compound **16r** in $CDCl_3$.

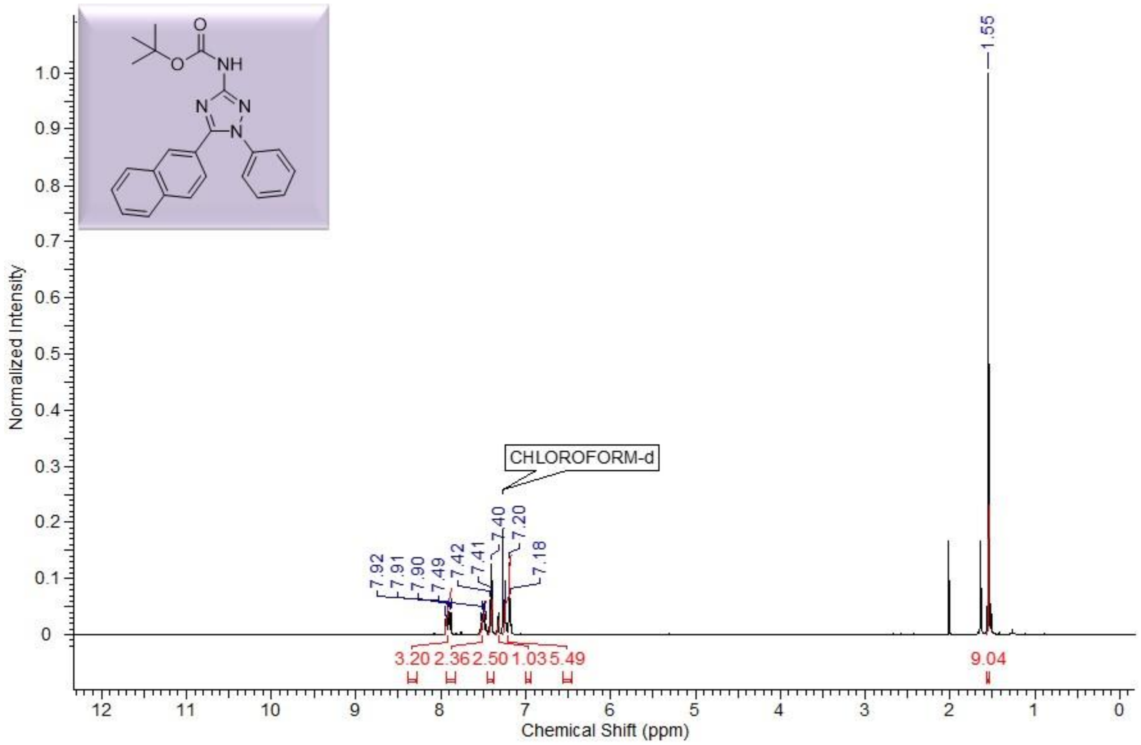

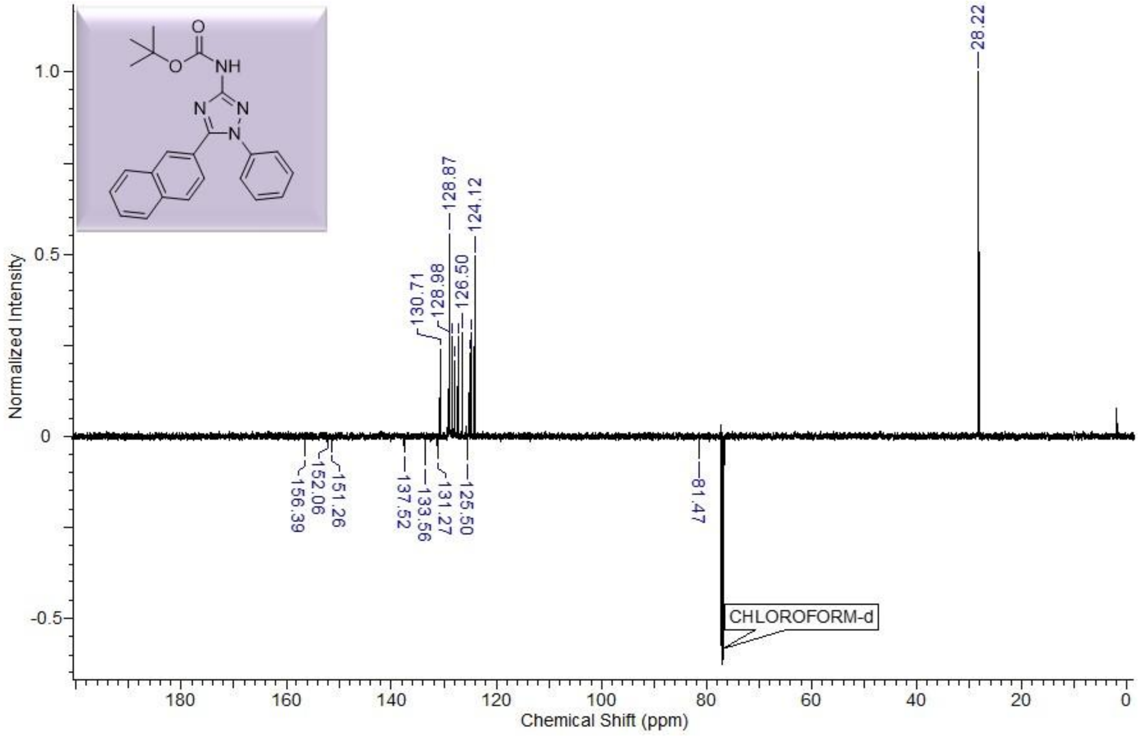

Figure S29: ^{1}H NMR (500 MHz) and ^{13}C NMR (125 MHz) spectra of compound **16s** in $CDCl_3$.

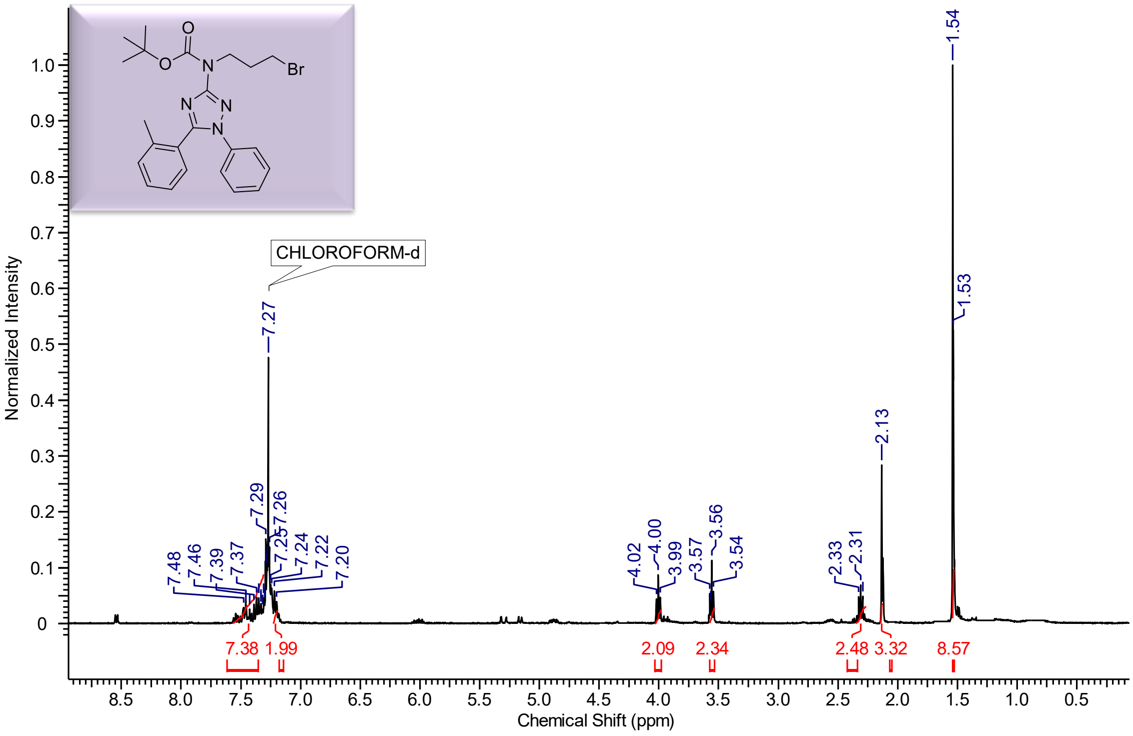

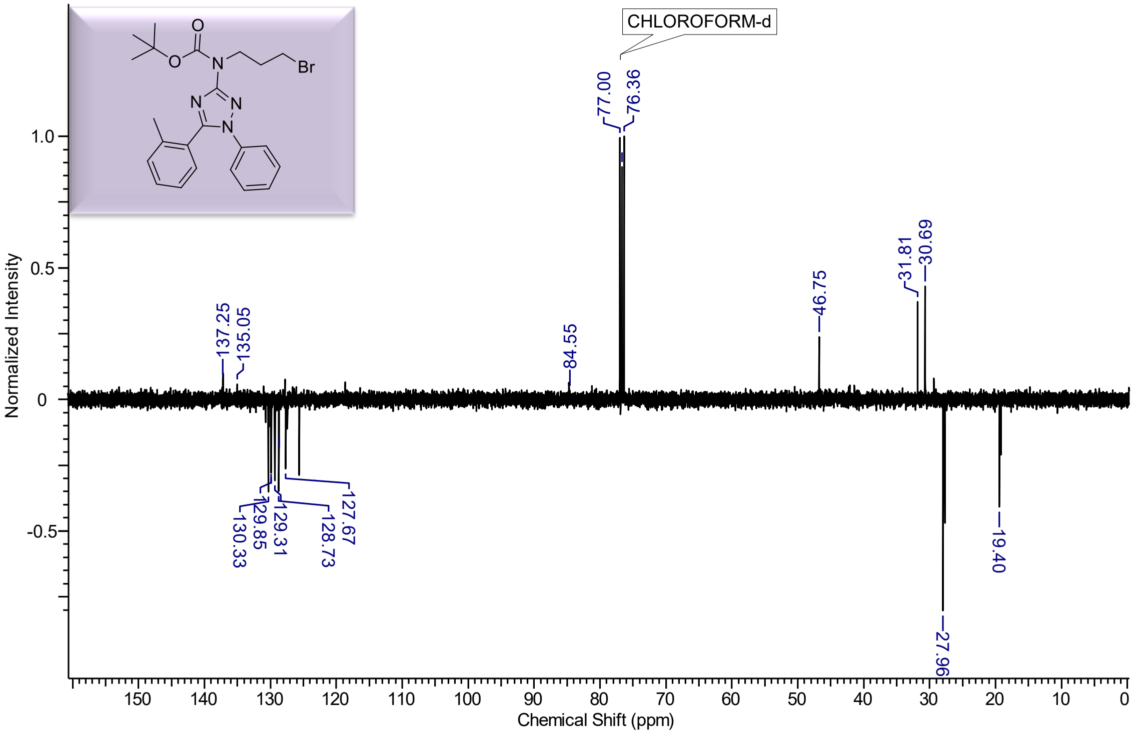

Figure S30: ^{1}H NMR (500 MHz) and ^{13}C NMR (125 MHz) spectra of compound **17a** in $CDCl_3$.

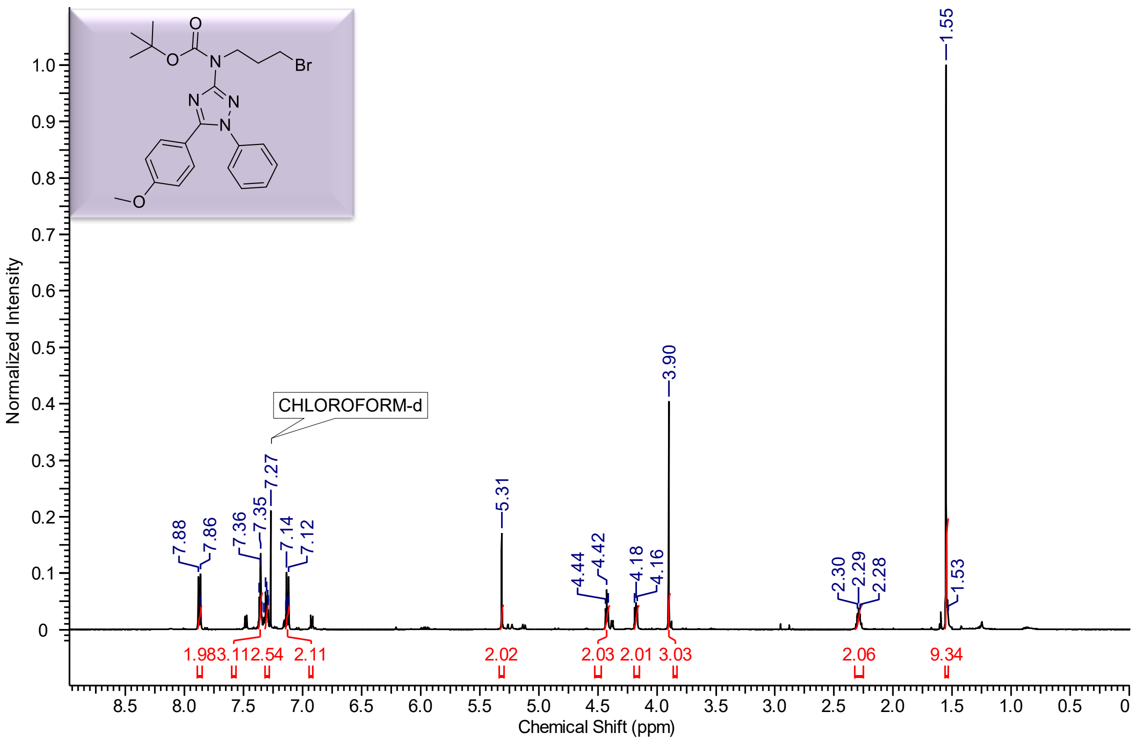

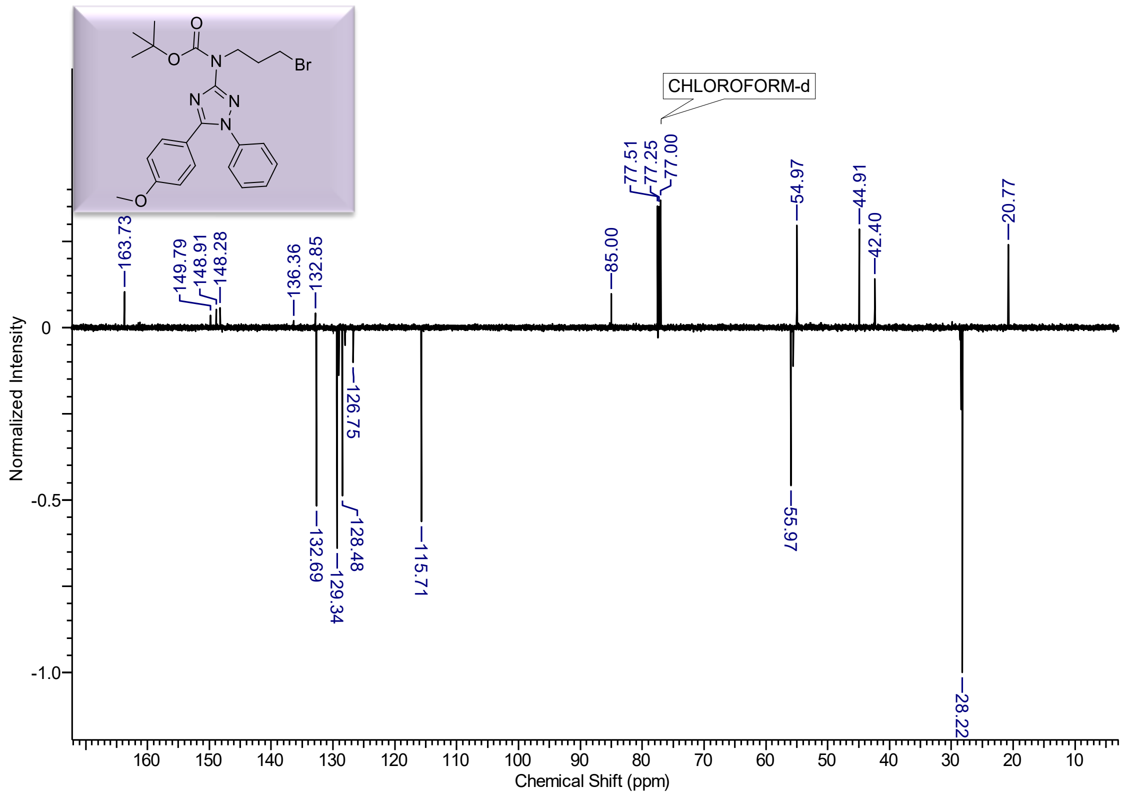

Figure S31: ^{1}H NMR (500 MHz) and ^{13}C NMR (125 MHz) spectra of compound **17e** in $CDCl_3$.

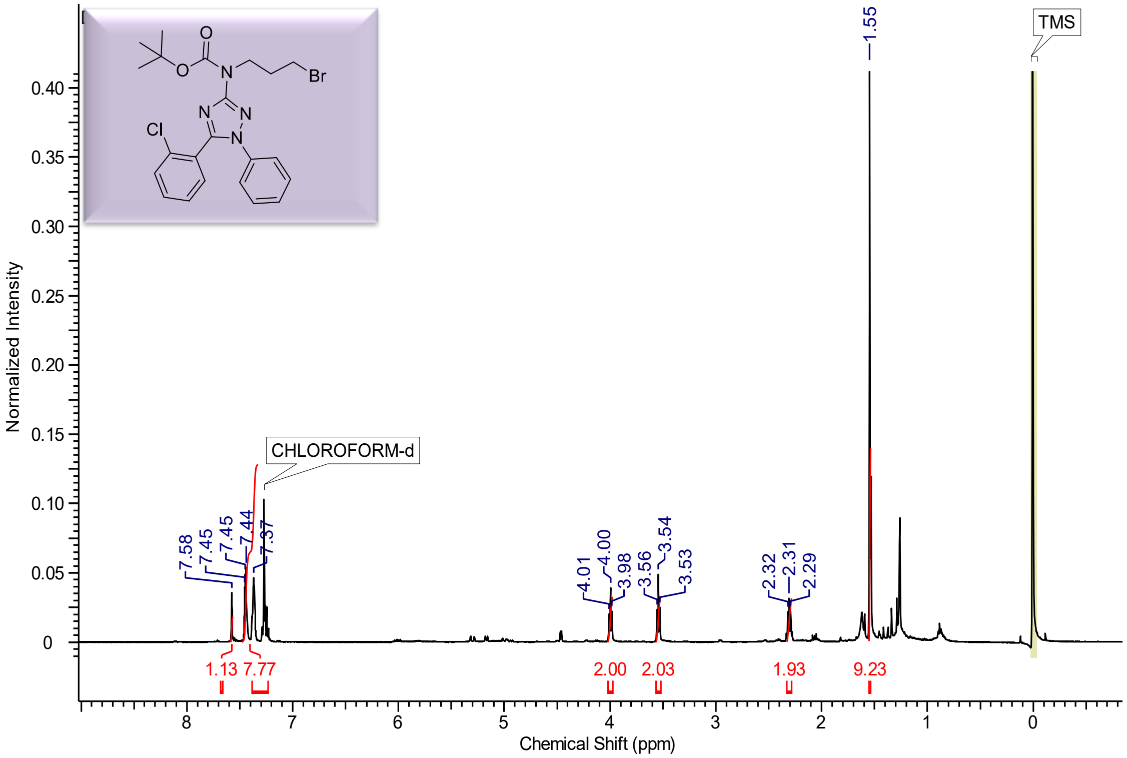

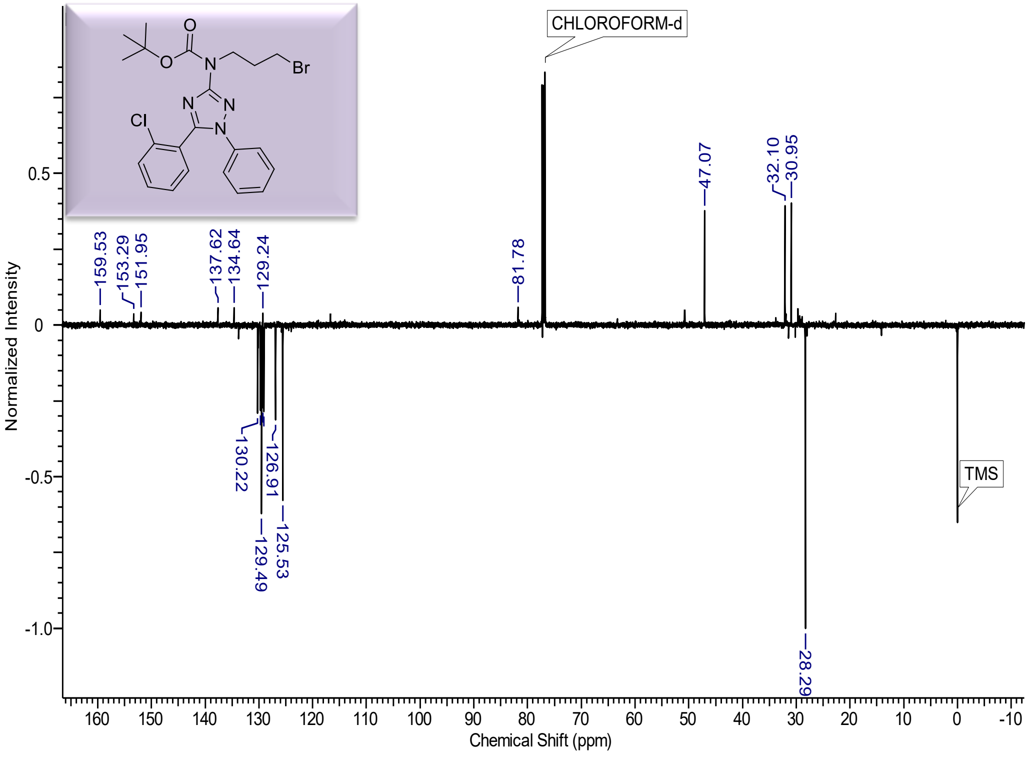

Figure S32: ^{1}H NMR (500 MHz) and ^{13}C NMR (125 MHz) spectra of compound **17h** in $CDCl_3$.

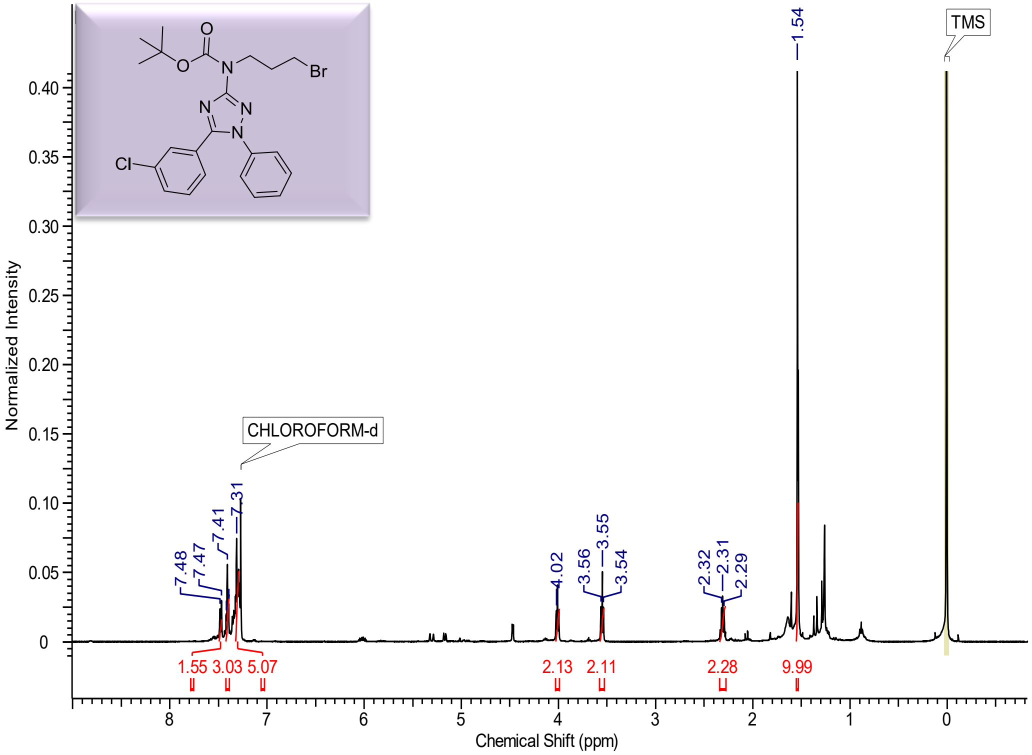

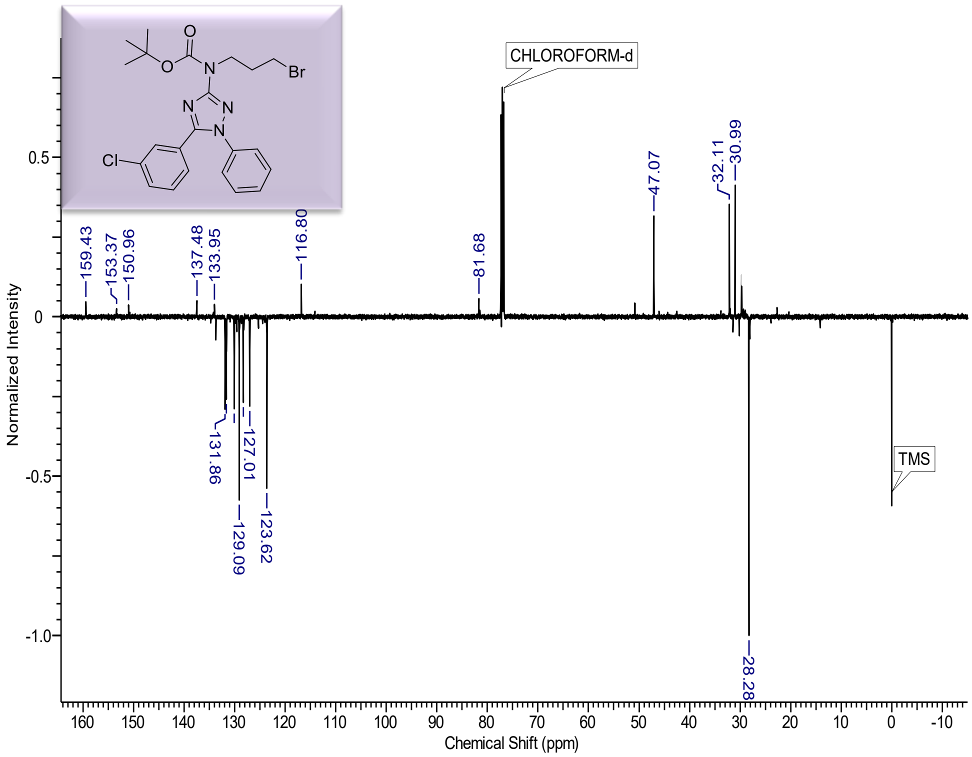

Figure S33: ^{1}H NMR (500 MHz) and ^{13}C NMR (125 MHz) spectra of compound **17i** in $CDCl_3$.

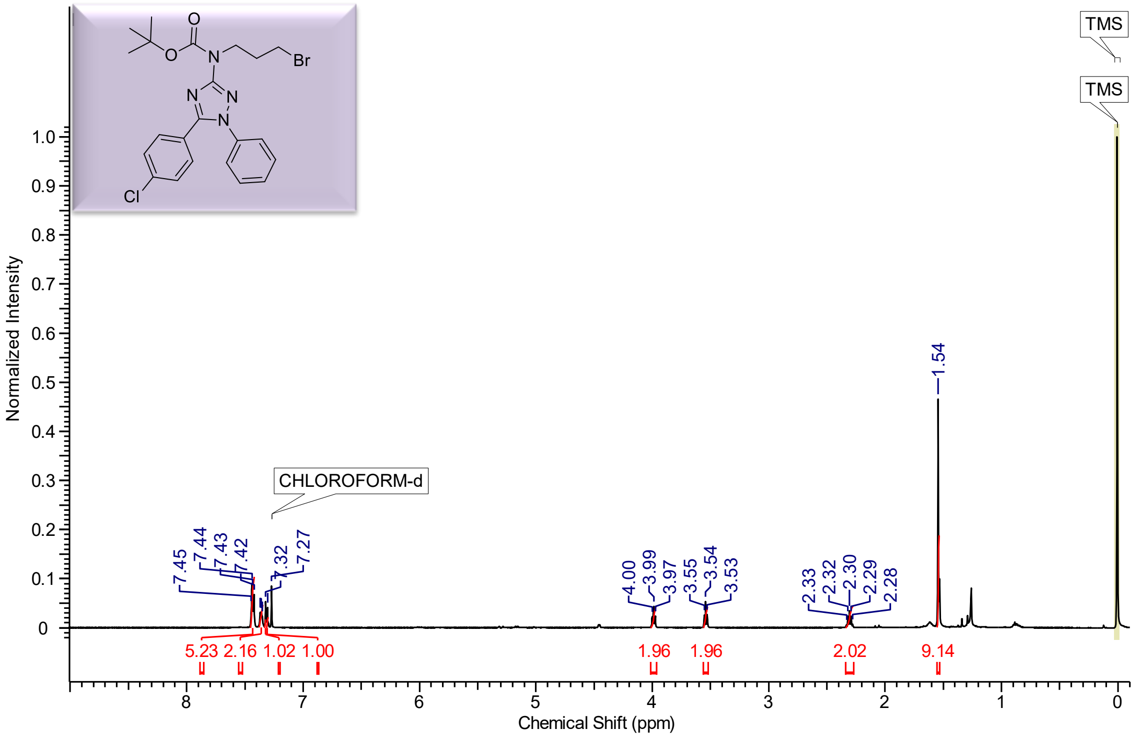

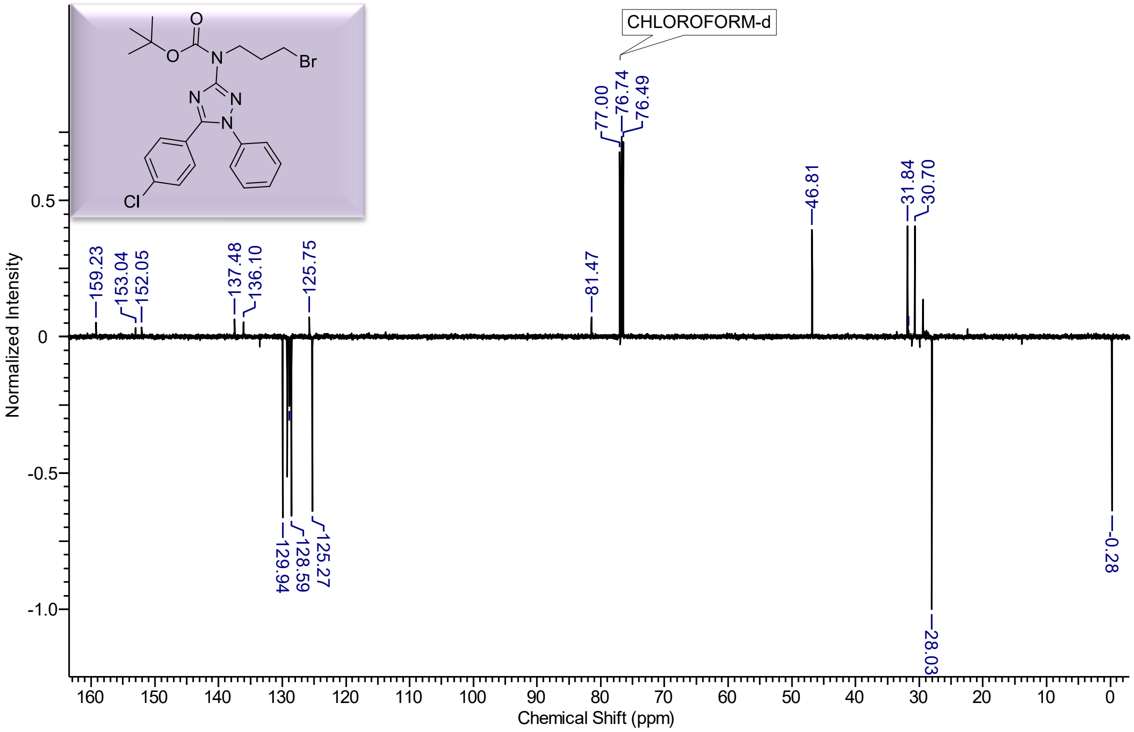

Figure S34: ^{1}H NMR (500 MHz) and ^{13}C NMR (125 MHz) spectra of compound **17j** in $CDCl_3$.

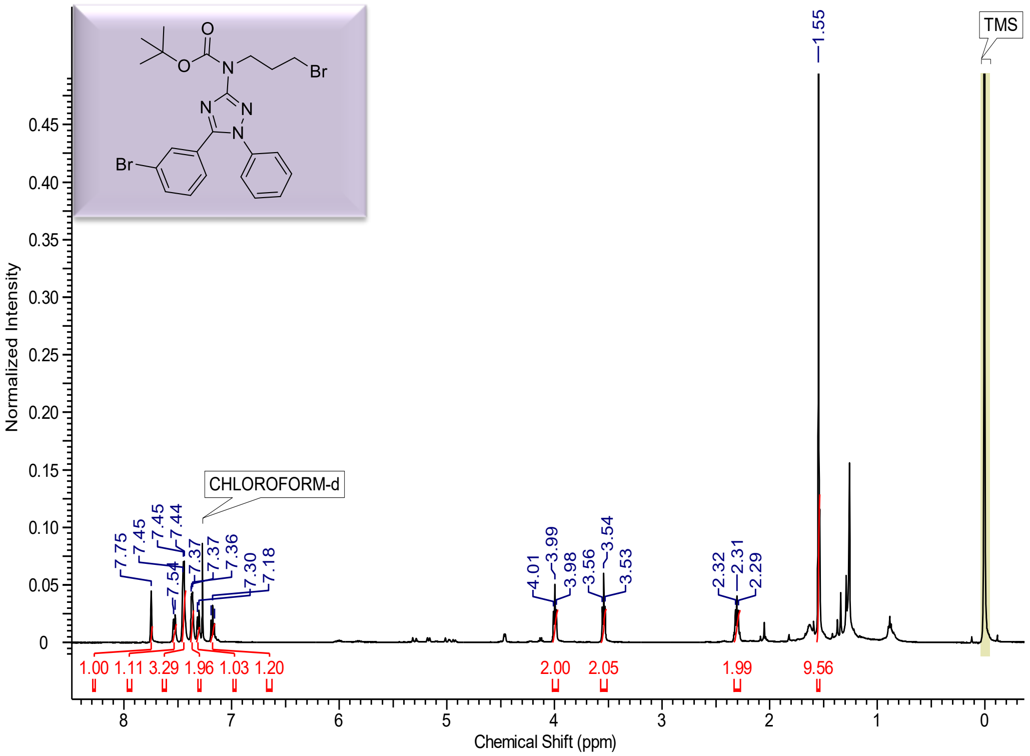

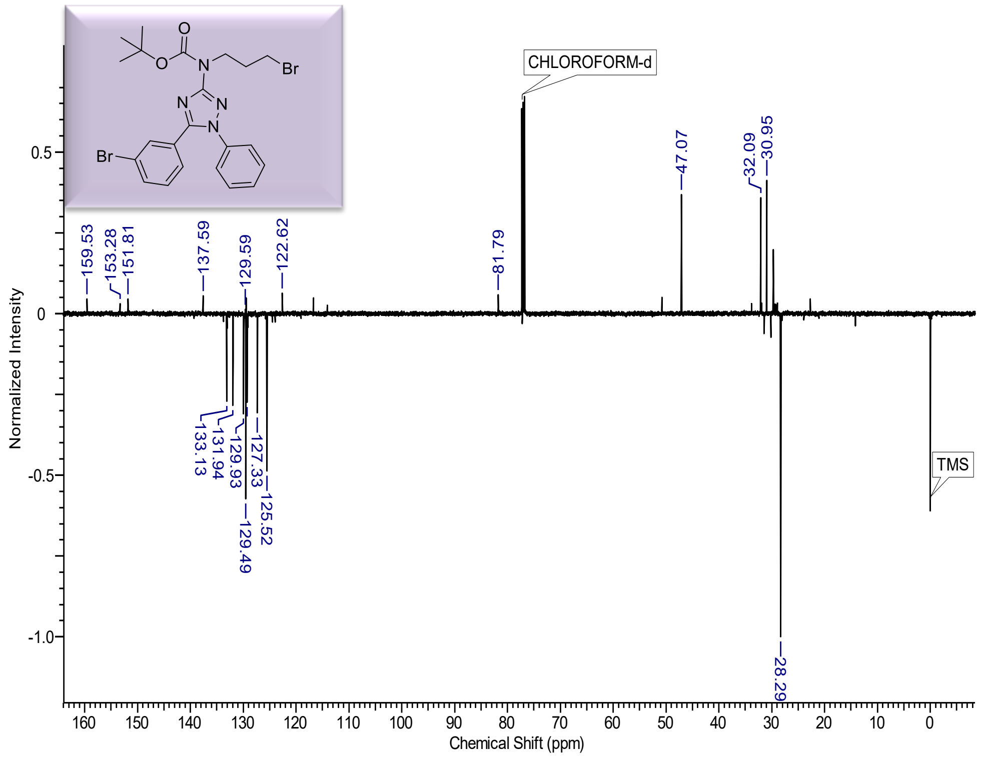

Figure S35: ^{1}H NMR (500 MHz) and ^{13}C NMR (125 MHz) spectra of compound **17k** in $CDCl_3$.

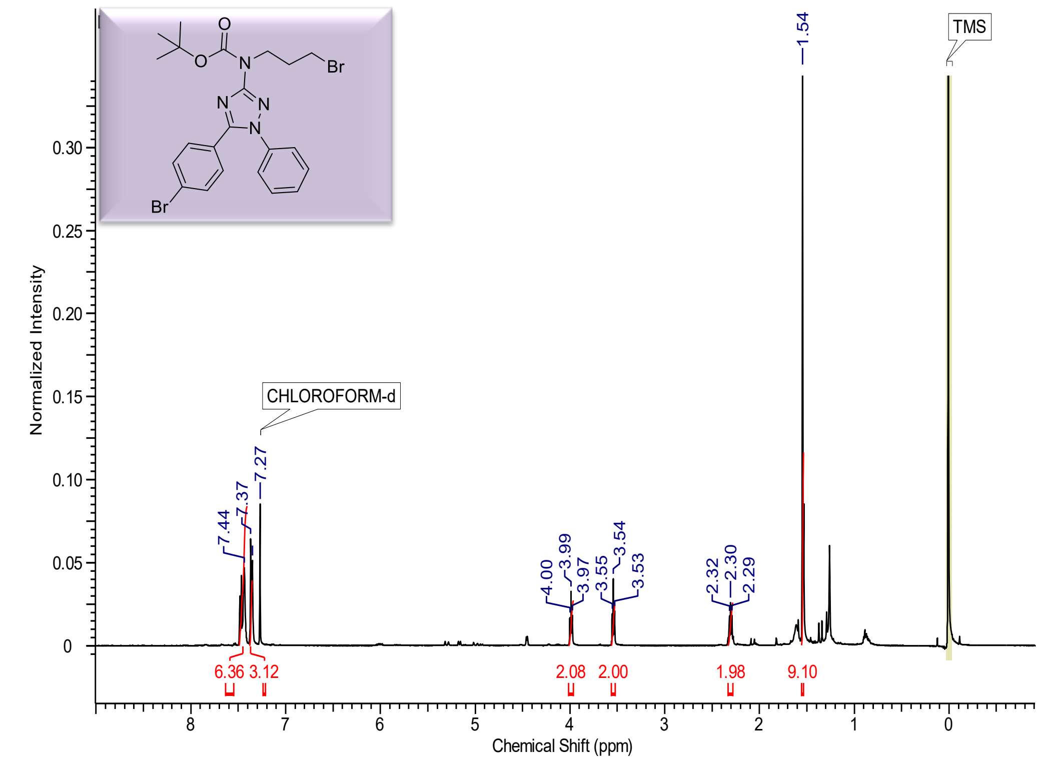

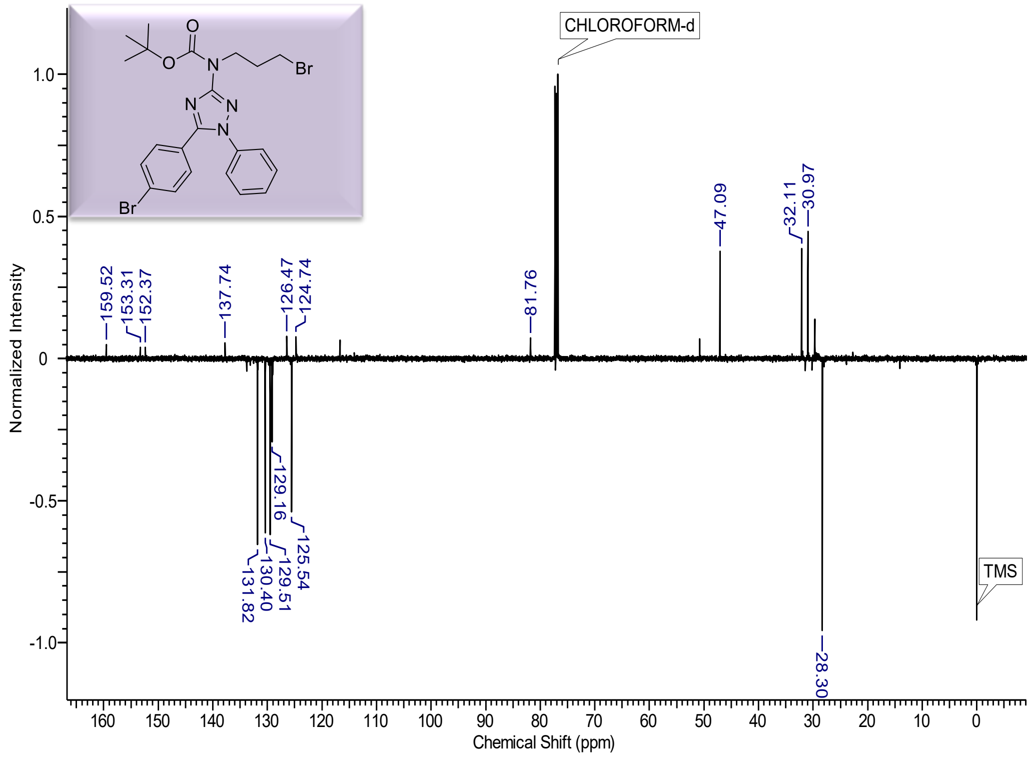

Figure S36: ^{1}H NMR (500 MHz) and ^{13}C NMR (125 MHz) spectra of compound **17l** in $CDCl_3$.

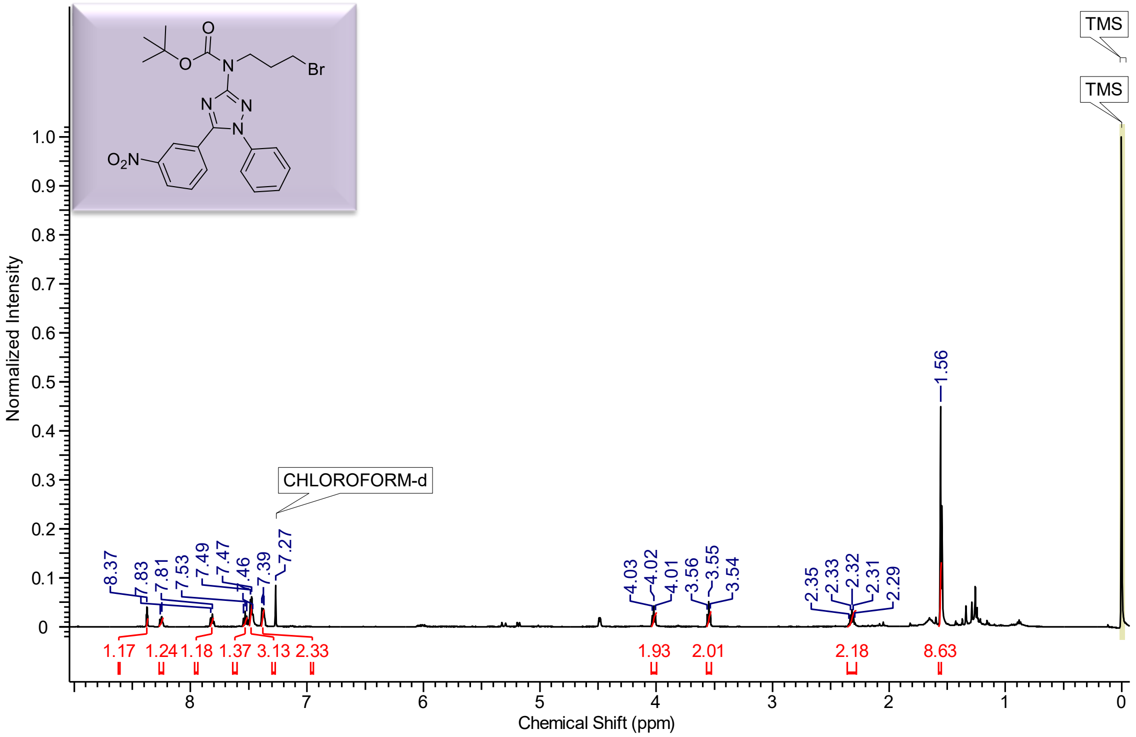

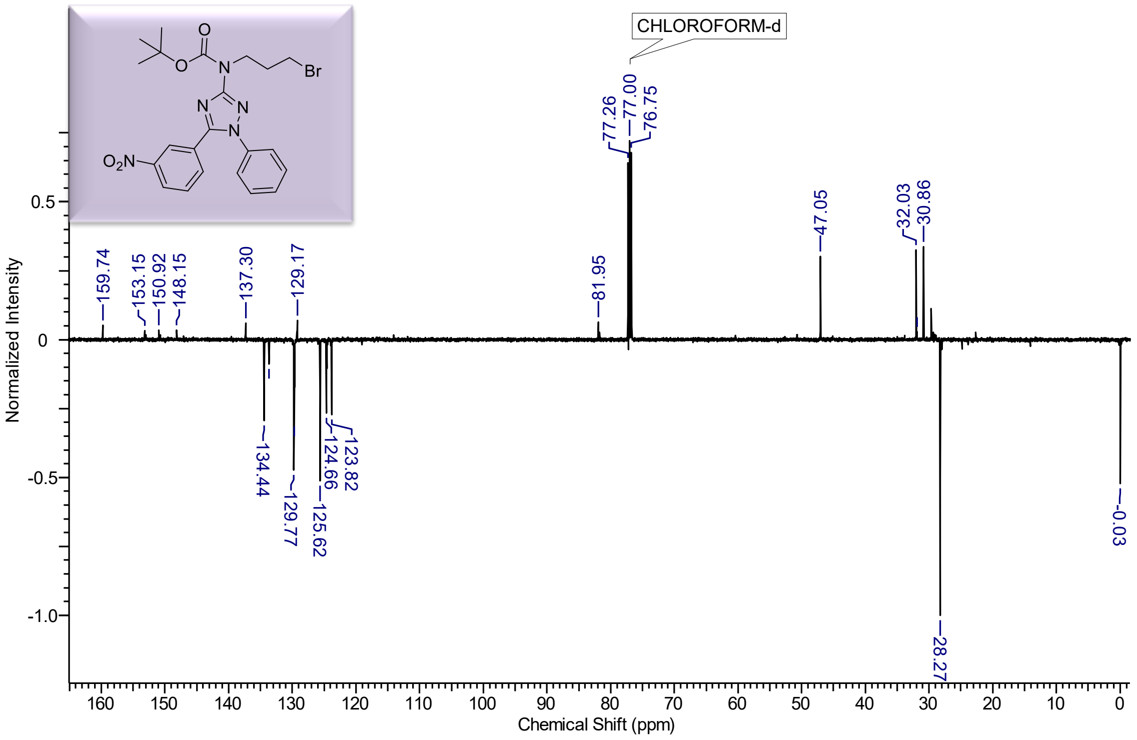

Figure S37: ^{1}H NMR (500 MHz) and ^{13}C NMR (125 MHz) spectra of compound **17m** in $CDCl_3$.

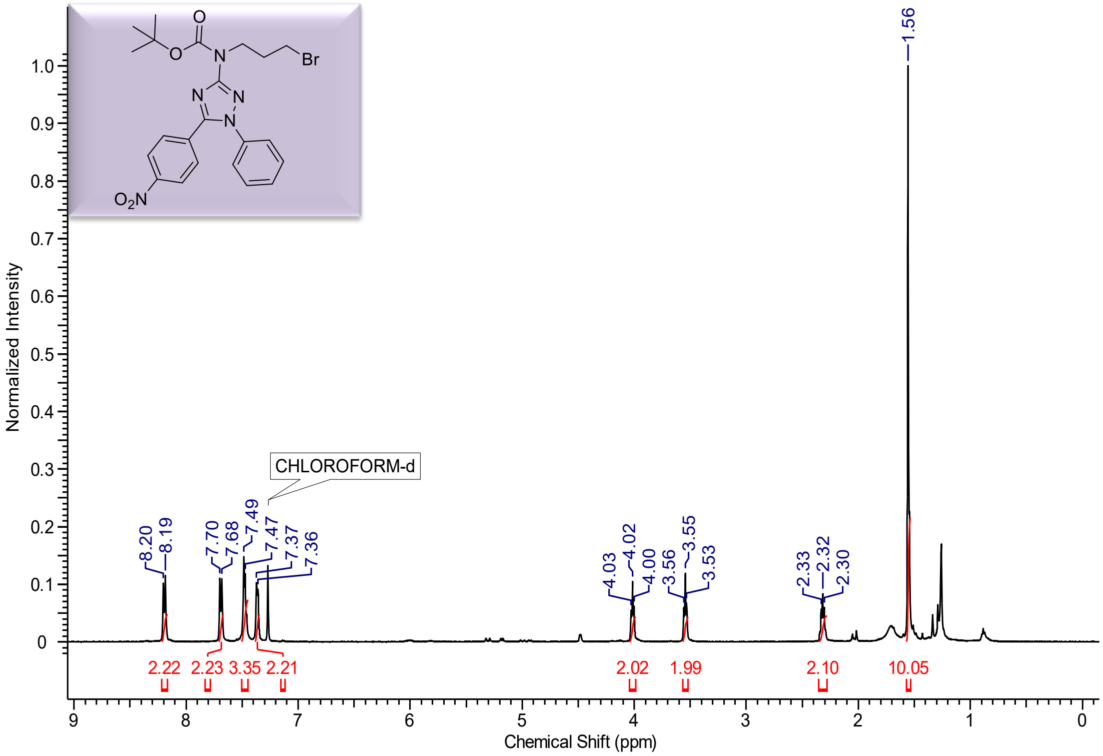

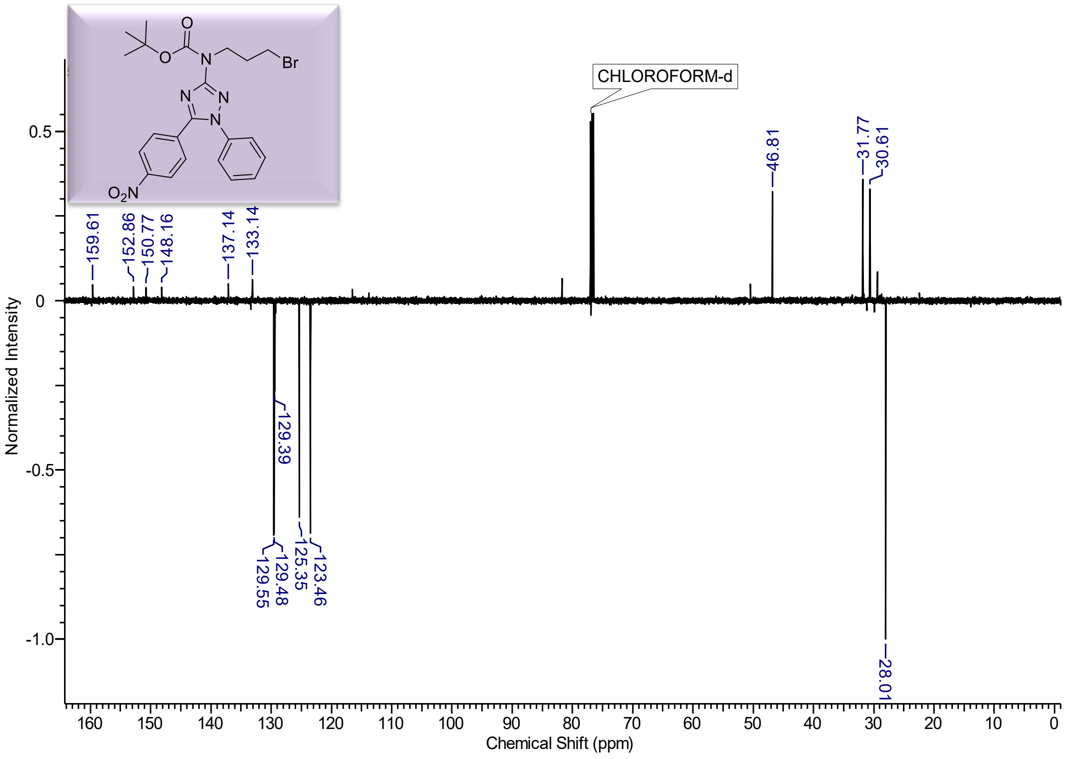

Figure S38: ^{1}H NMR (500 MHz) and ^{13}C NMR (125 MHz) spectra of compound **17n** in $CDCl_3$.

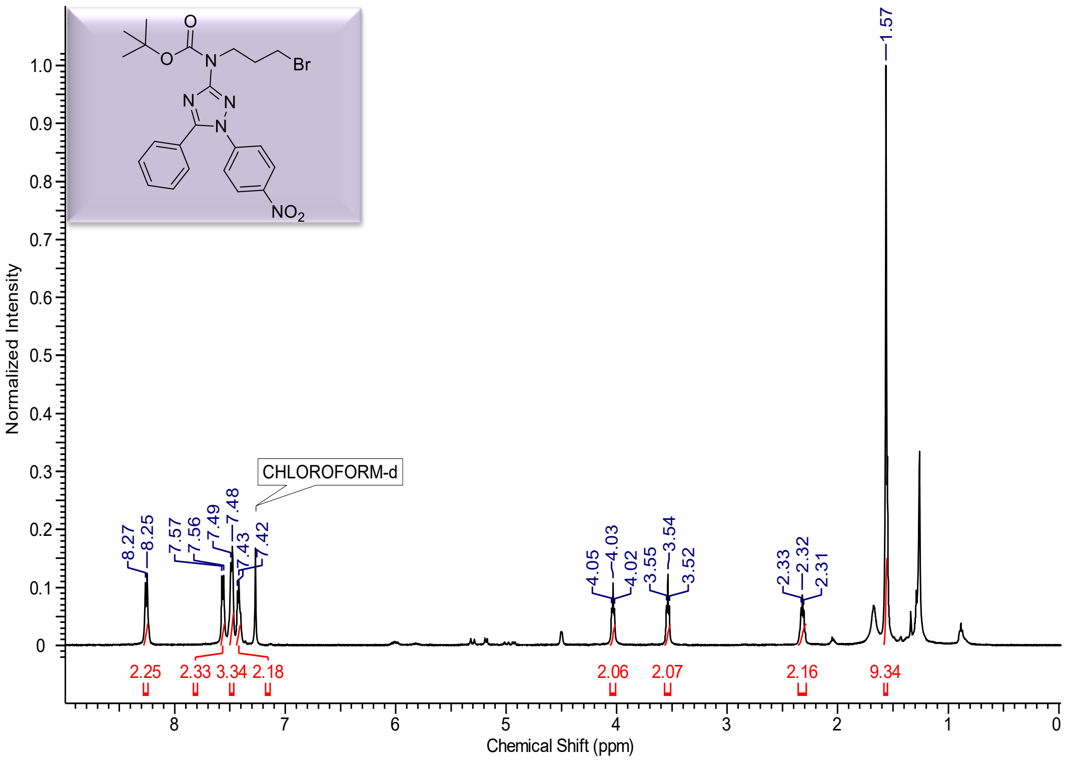

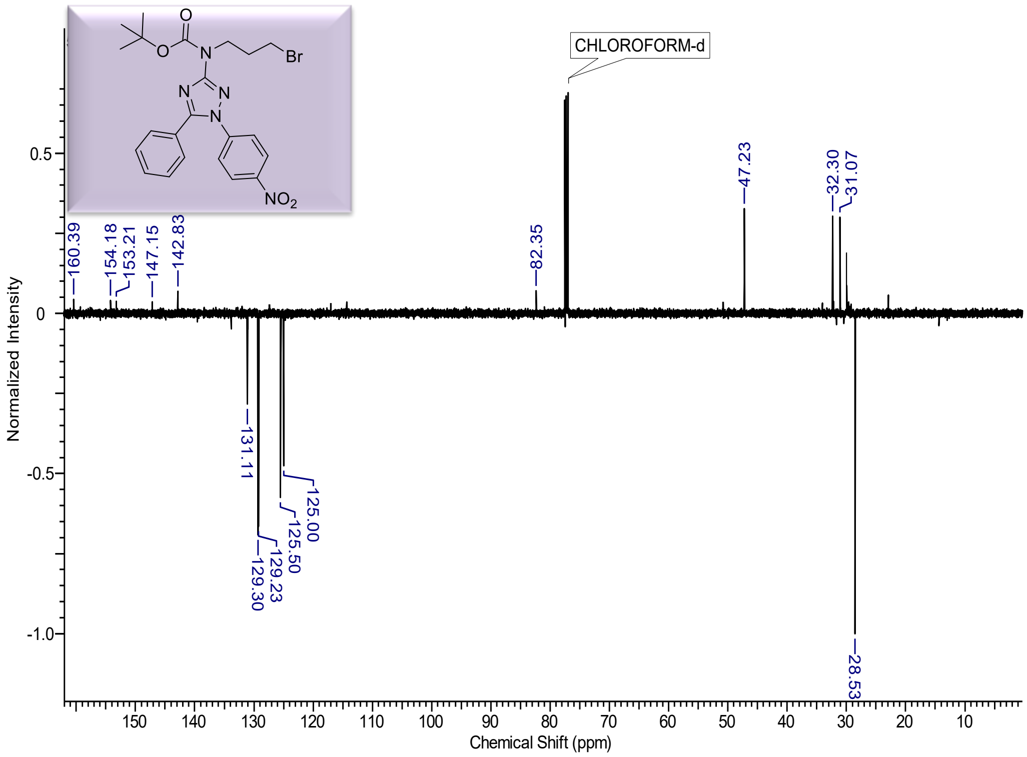

Figure S39: ^{1}H NMR (500 MHz) and ^{13}C NMR (125 MHz) spectra of compound **17o** in $CDCl_3$.

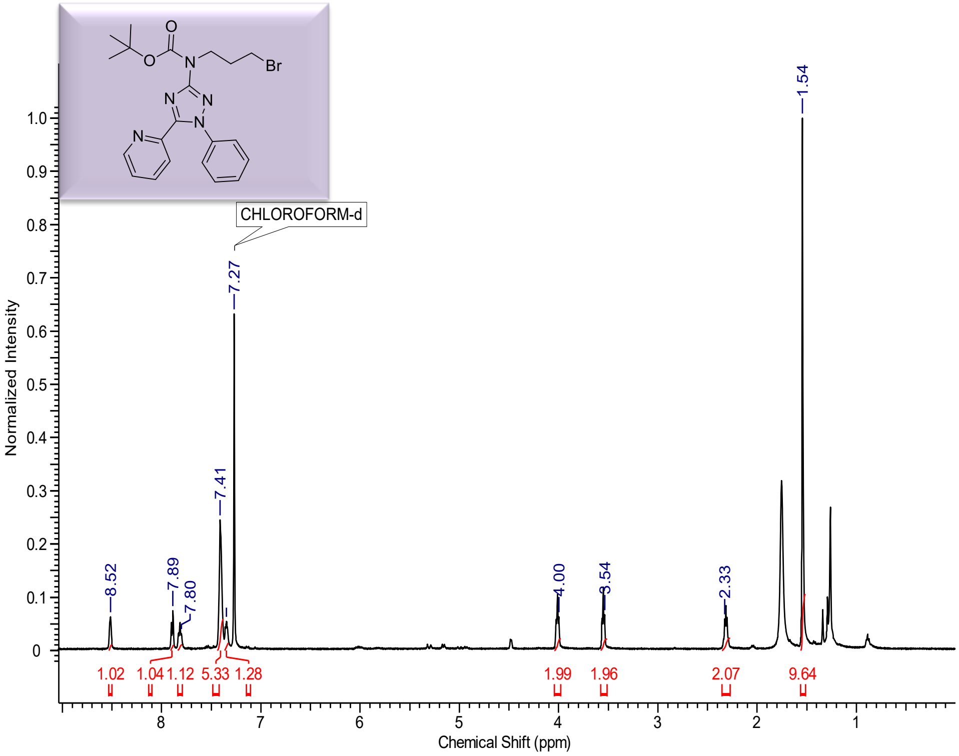

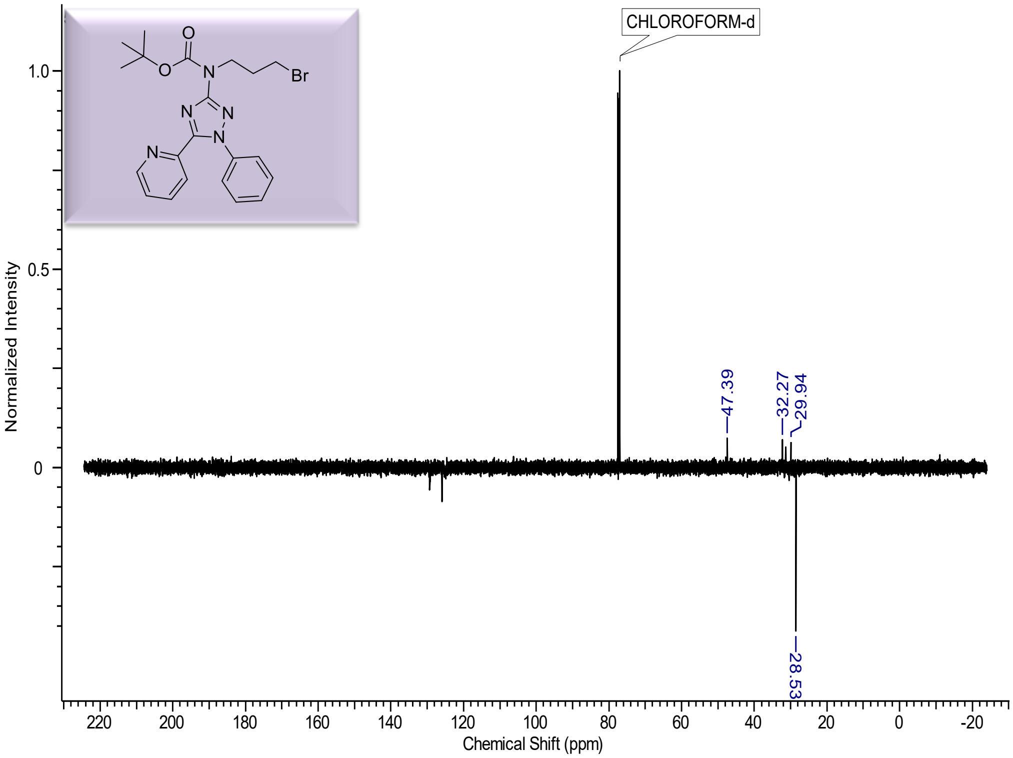

Figure S40: ^{1}H NMR (500 MHz) and ^{13}C NMR (125 MHz) spectra of compound **17p** in $CDCl_3$.

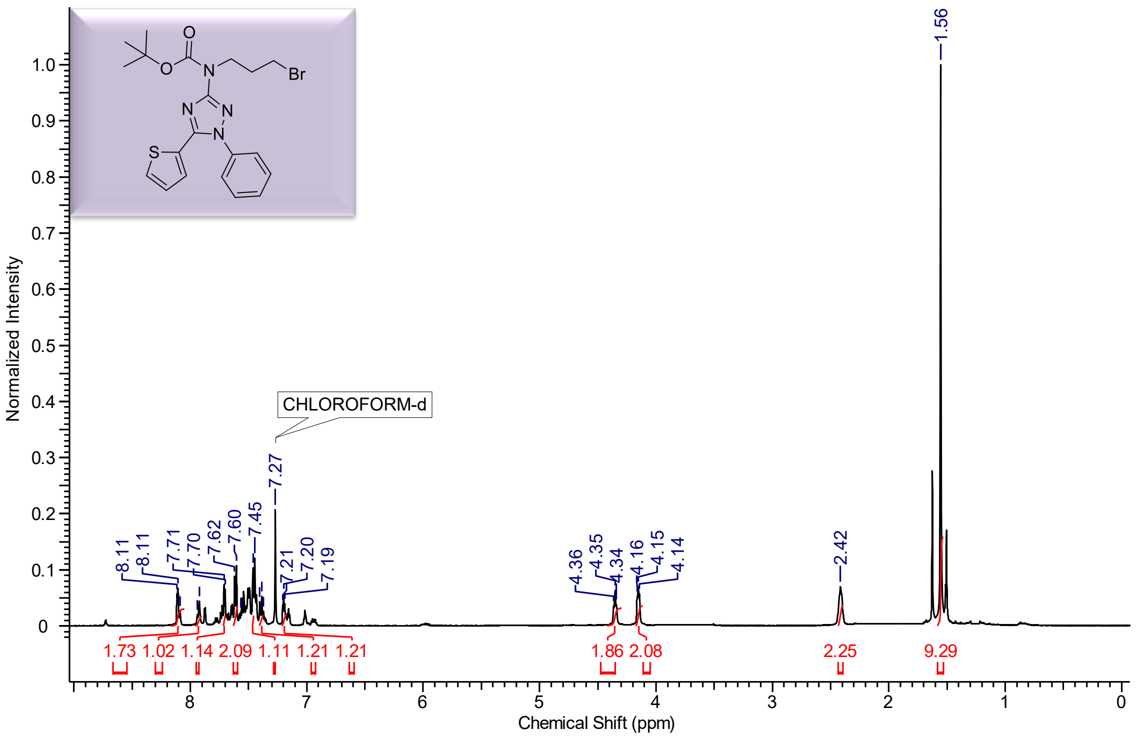

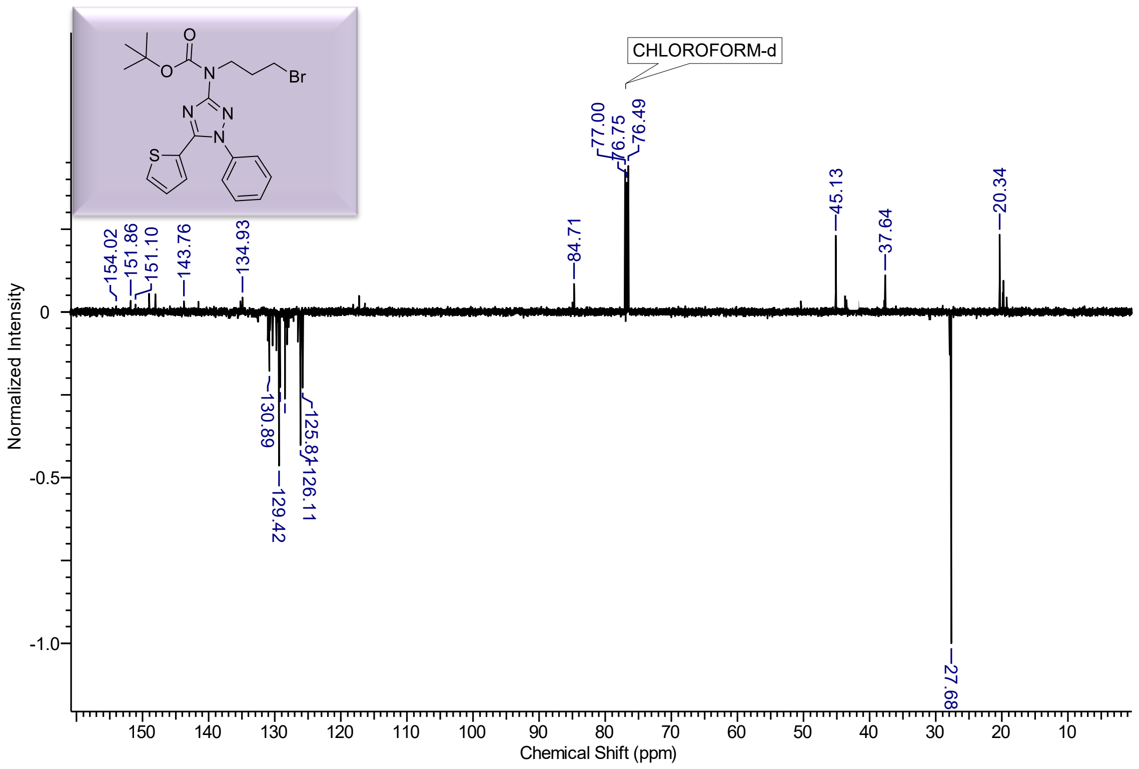

Figure S41: ^{1}H NMR (500 MHz) and ^{13}C NMR (125 MHz) spectra of compound **17r** in $CDCl_3$.

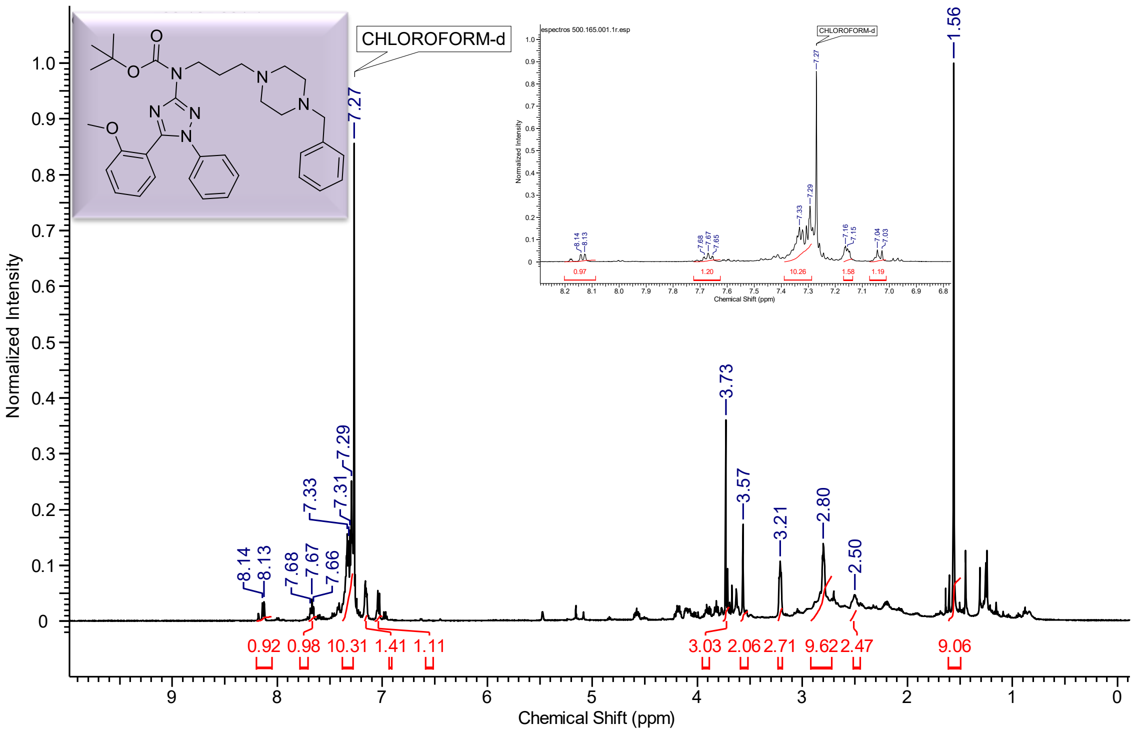

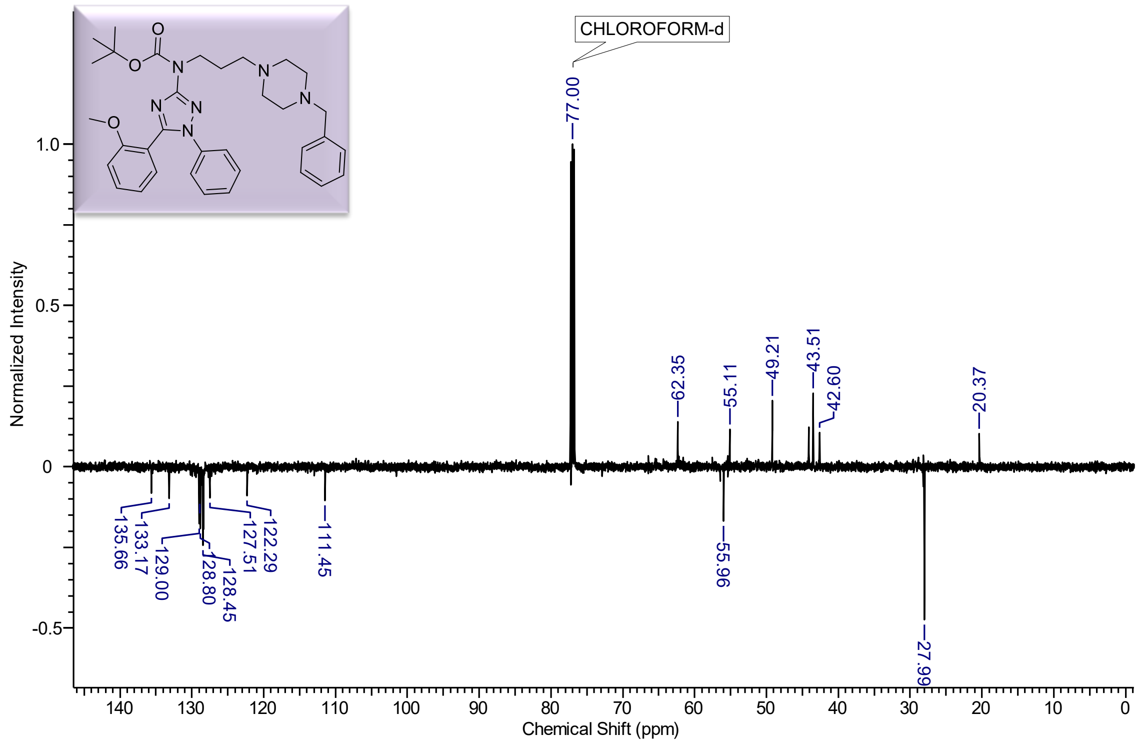

Figure S42: ^{1}H NMR (500 MHz) and ^{13}C NMR (125 MHz) spectra of compound **18d** in $CDCl_3$.

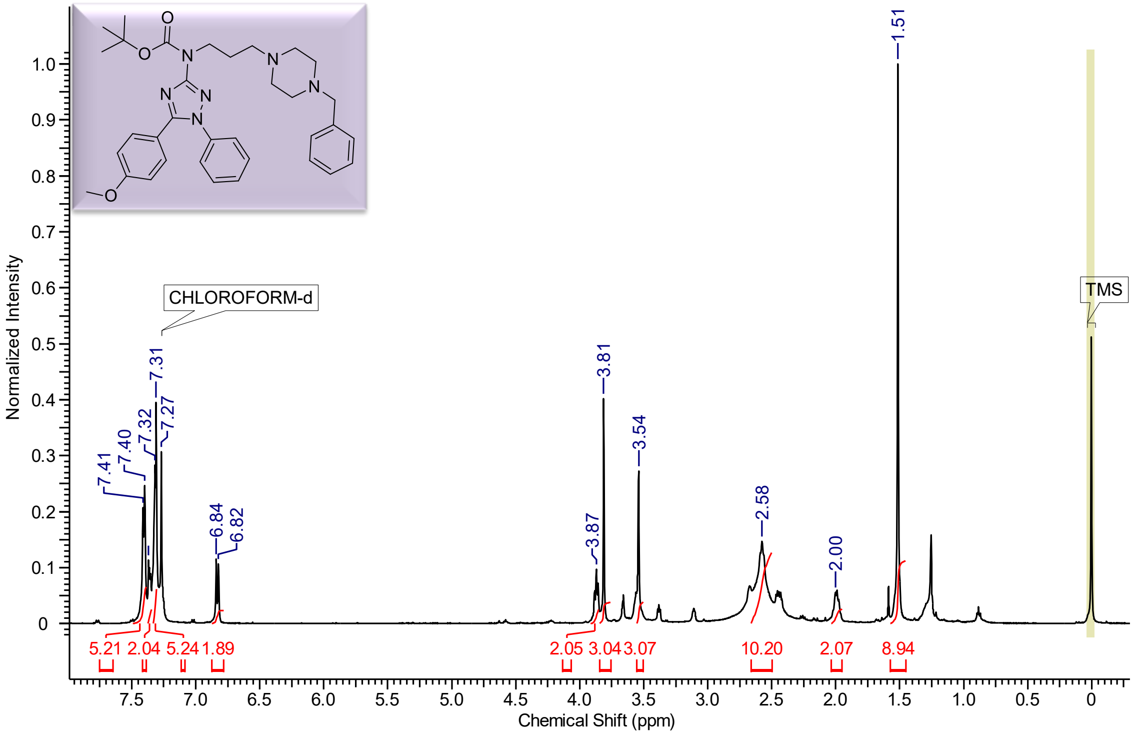

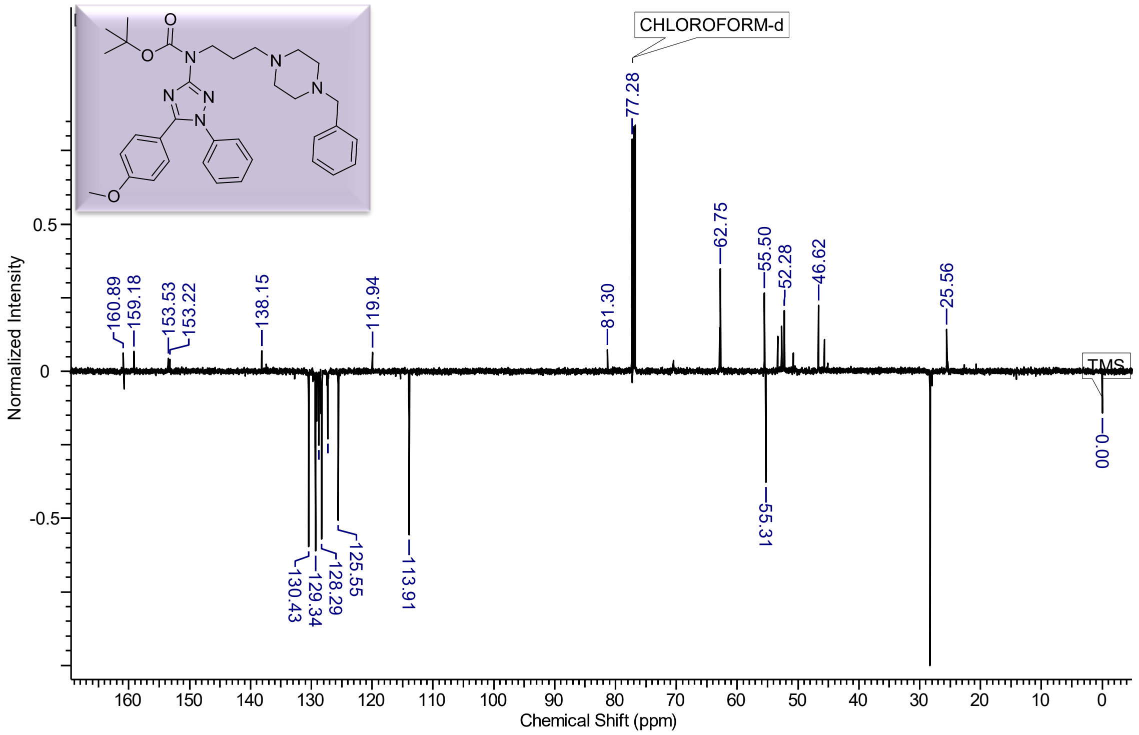

Figure S43: ^{1}H NMR (500 MHz) and ^{13}C NMR (125 MHz) spectra of compound **18e** in $CDCl_3$.

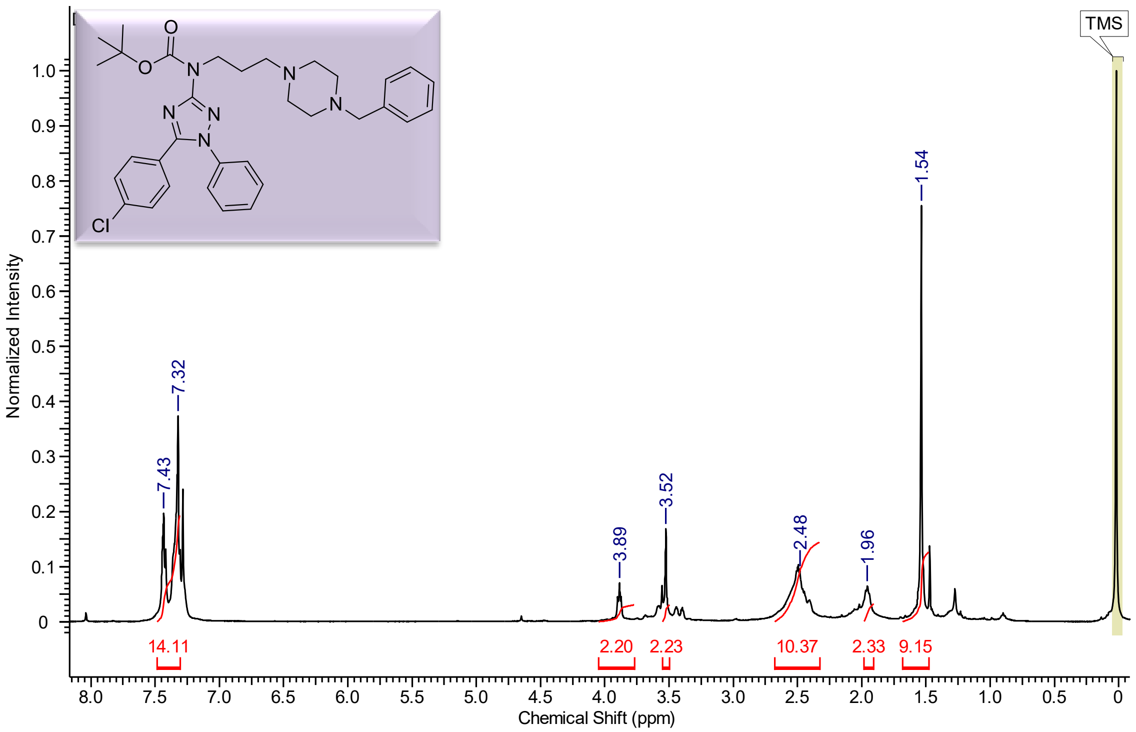

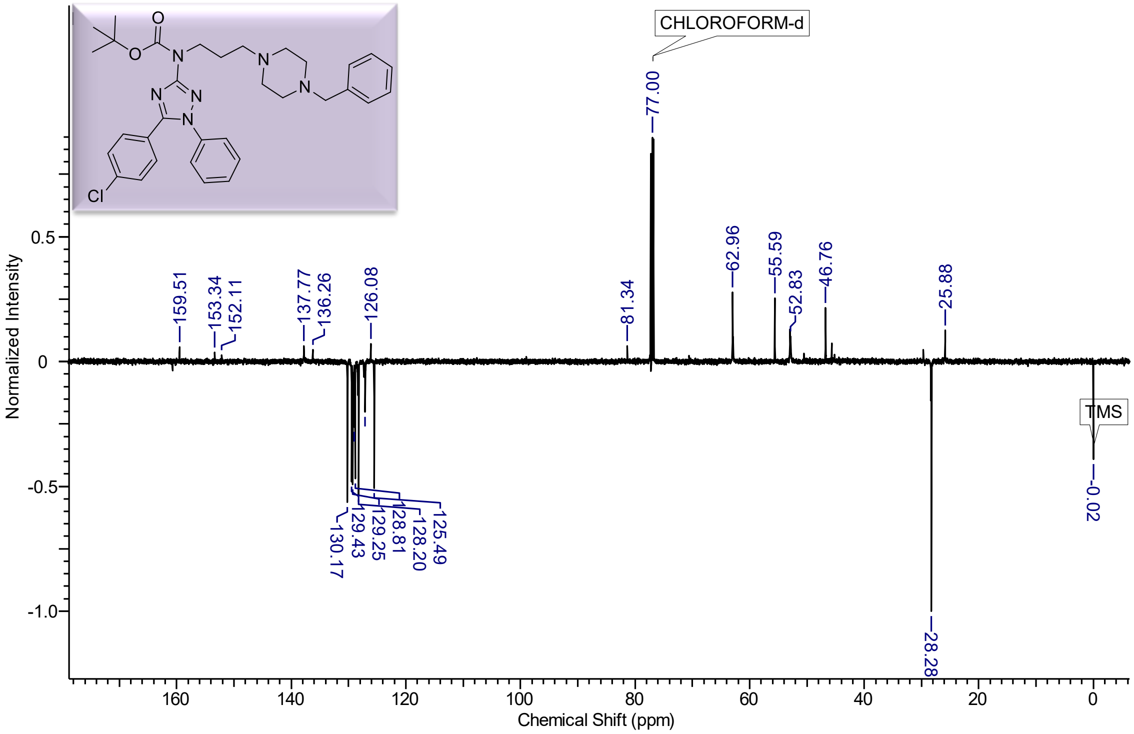

Figure S44: ^{1}H NMR (500 MHz) and ^{13}C NMR (125 MHz) spectra of compound **18j** in $CDCl_3$.

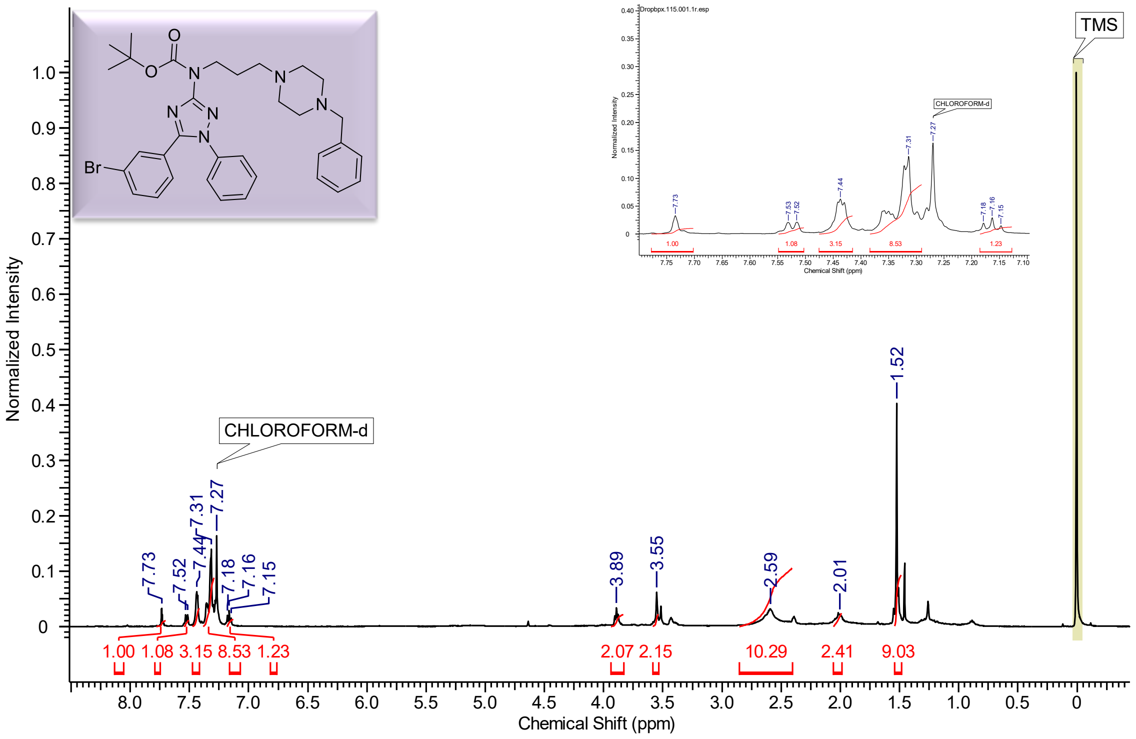

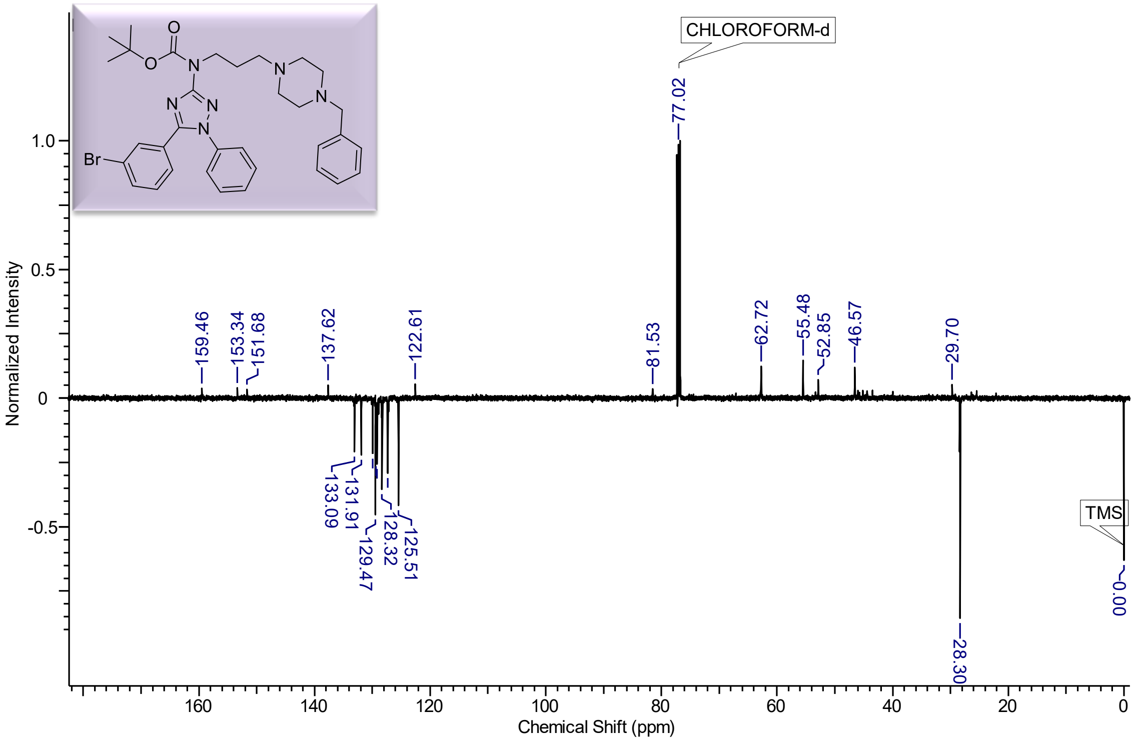

Figure S45: ^{1}H NMR (500 MHz) and ^{13}C NMR (125 MHz) spectra of compound **18k** in $CDCl_3$.

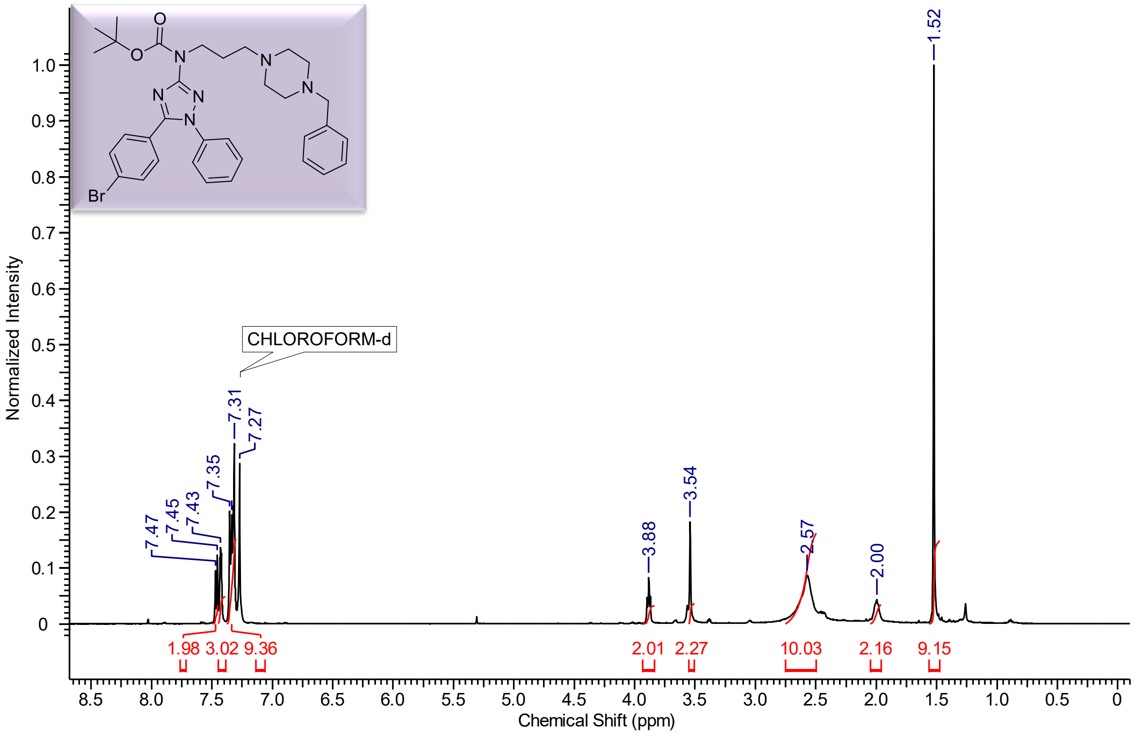

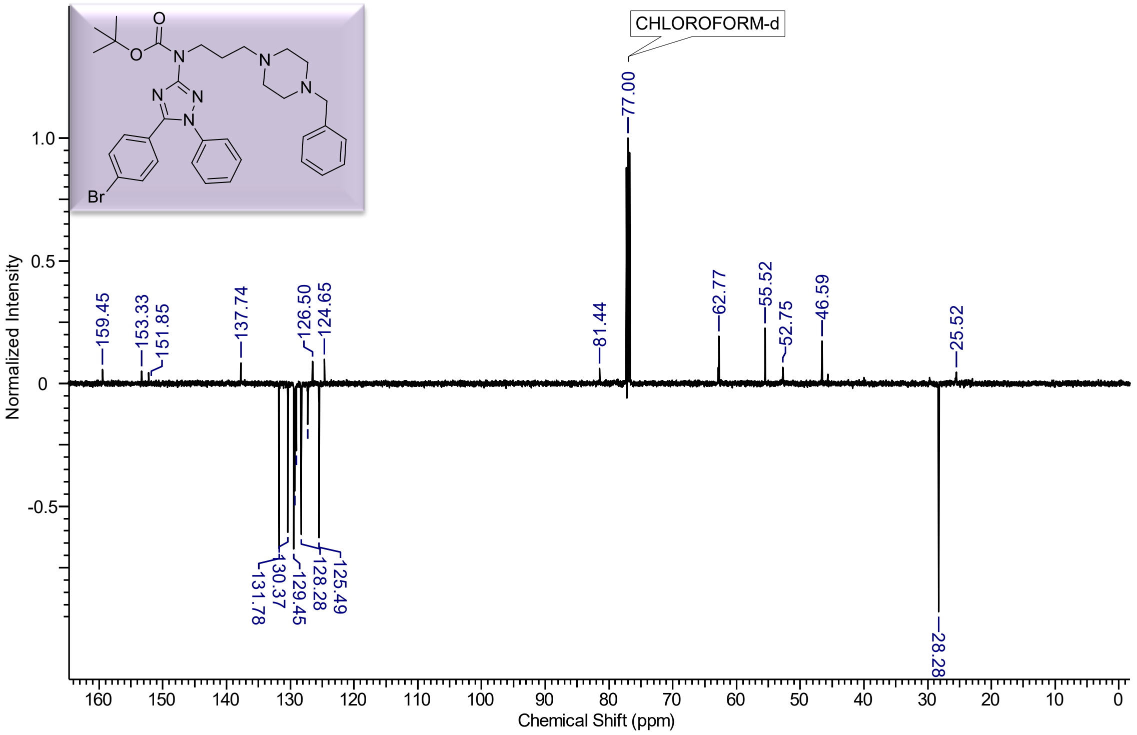

Figure S46: ^{1}H NMR (500 MHz) and ^{13}C NMR (125 MHz) spectra of compound **18l** in $CDCl_3$.

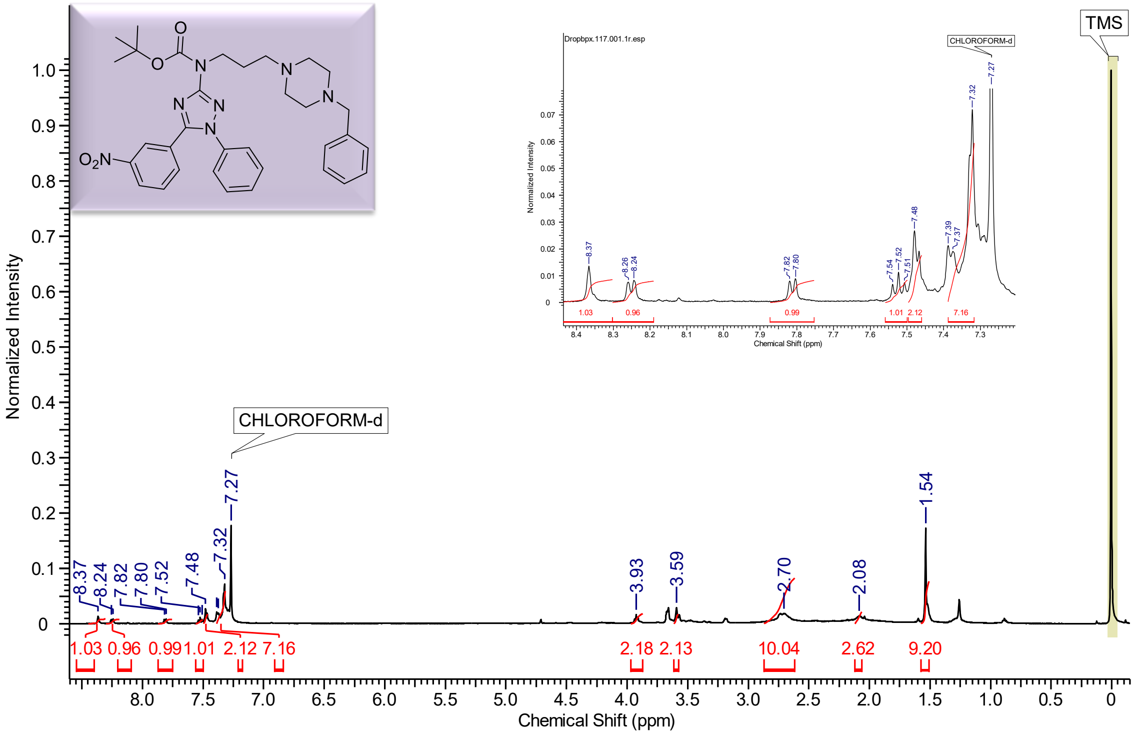

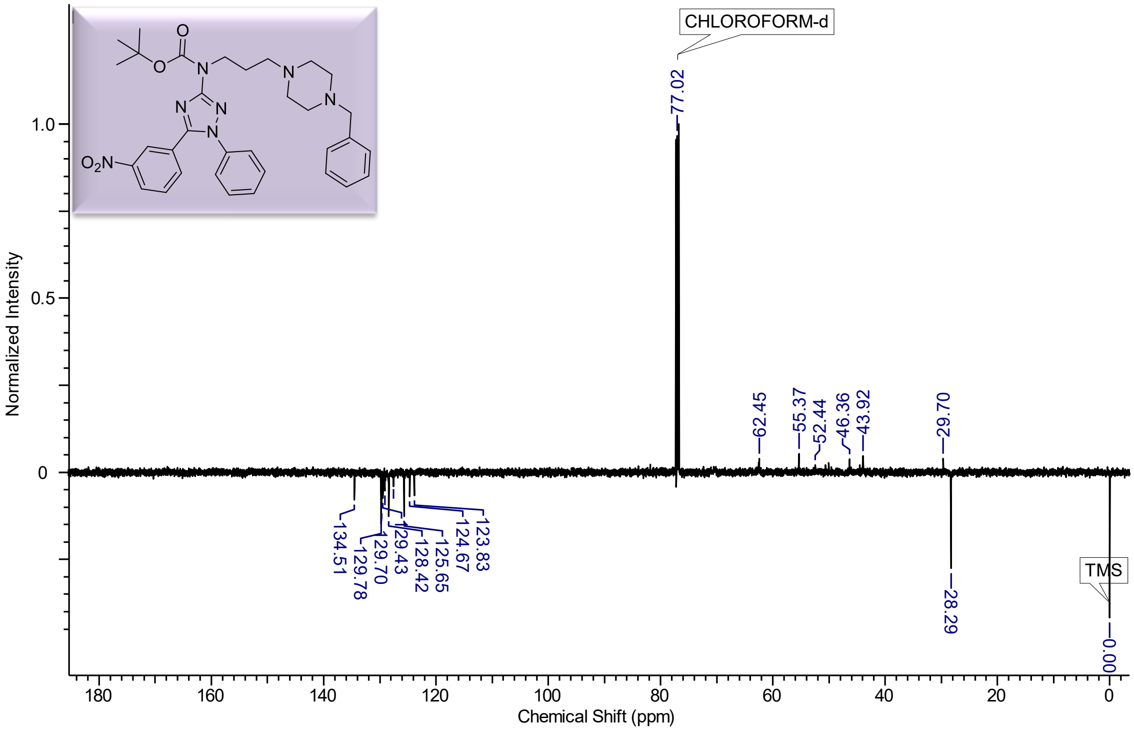

Figure S47: ^{1}H NMR (500 MHz) and ^{13}C NMR (125 MHz) spectra of compound **18m** in $CDCl_3$.

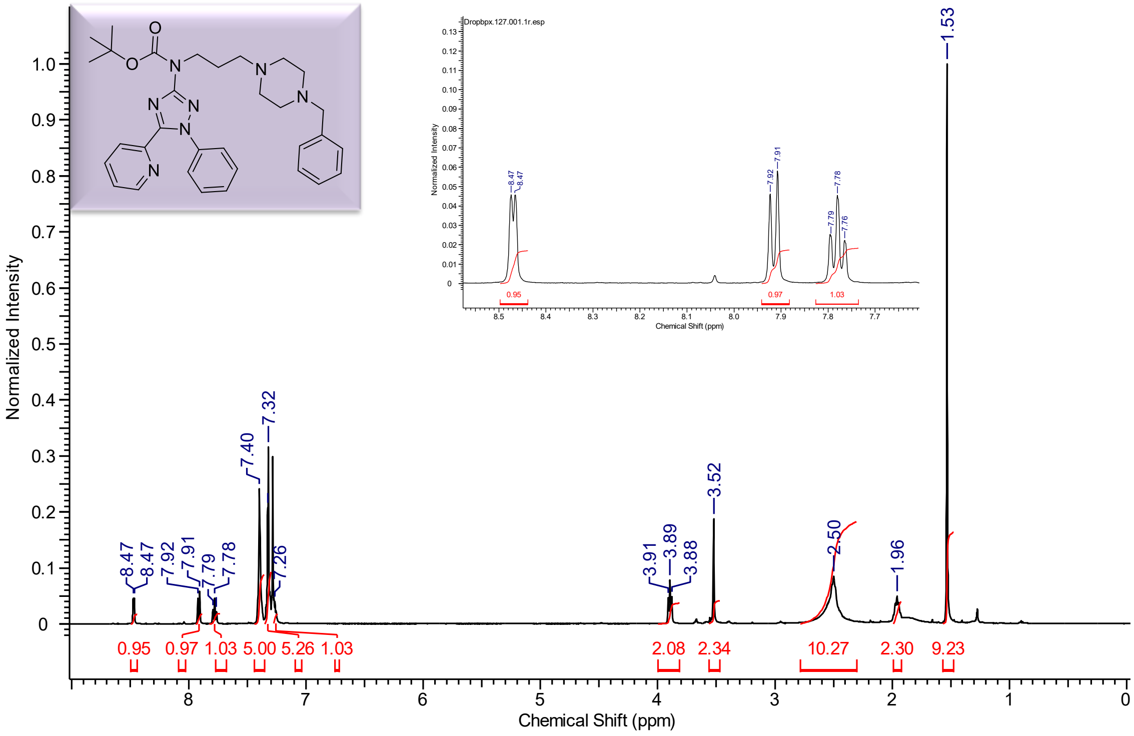

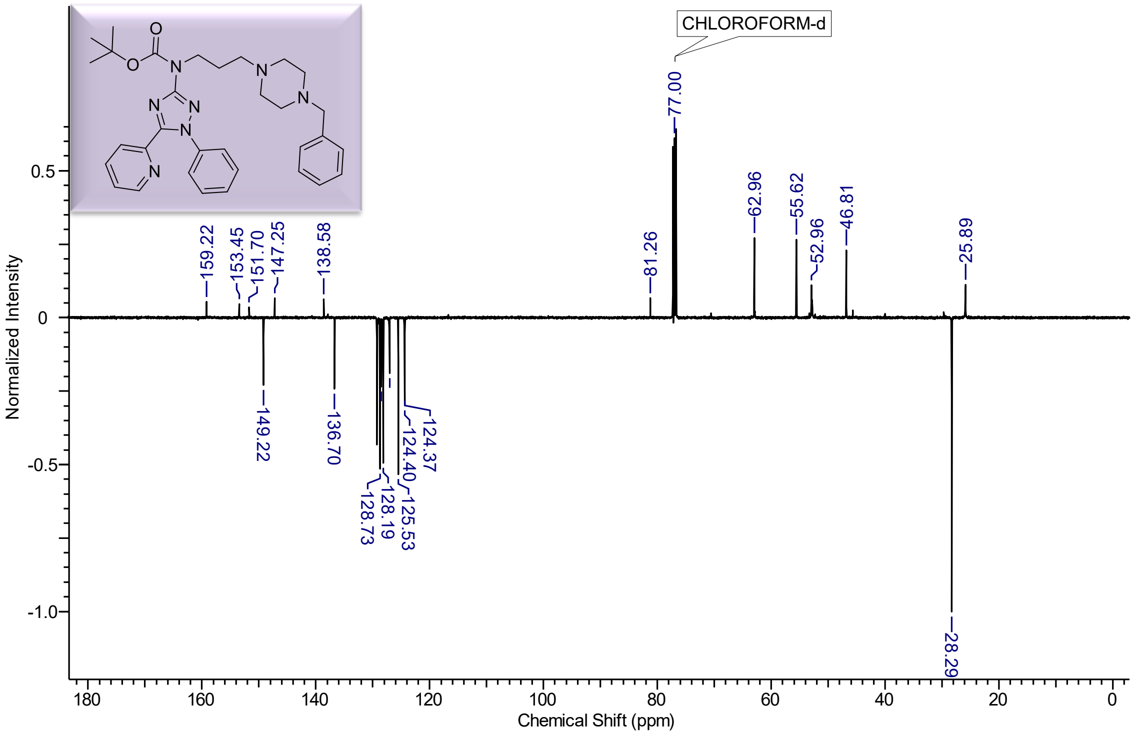

Figure S48: ^{1}H NMR (500 MHz) and ^{13}C NMR (125 MHz) spectra of compound **18p** in $CDCl_3$.

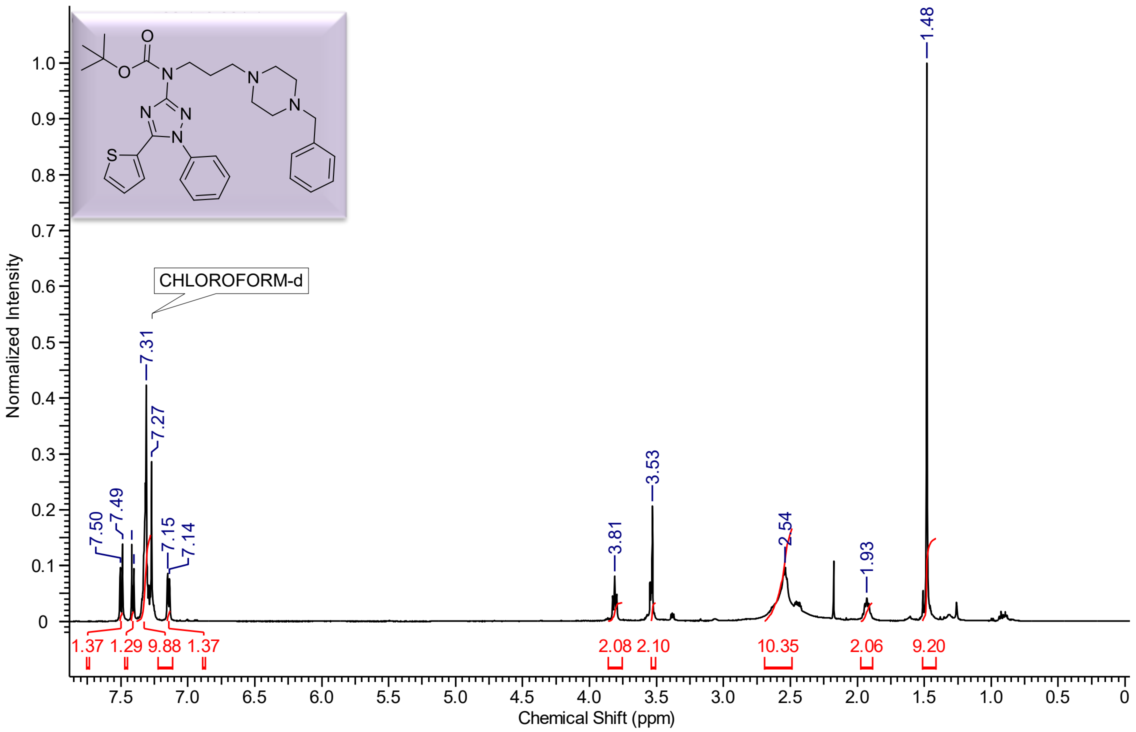

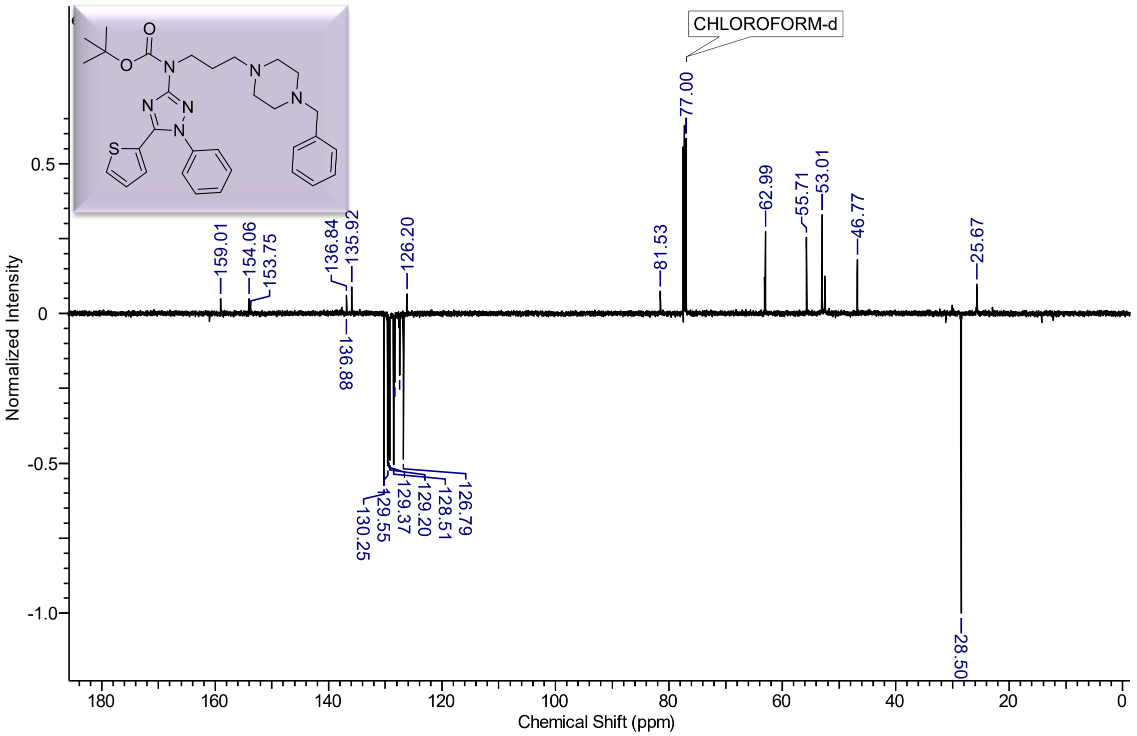

Figure S49: ^{1}H NMR (500 MHz) and ^{13}C NMR (125 MHz) spectra of compound **18r** in $CDCl_3$.

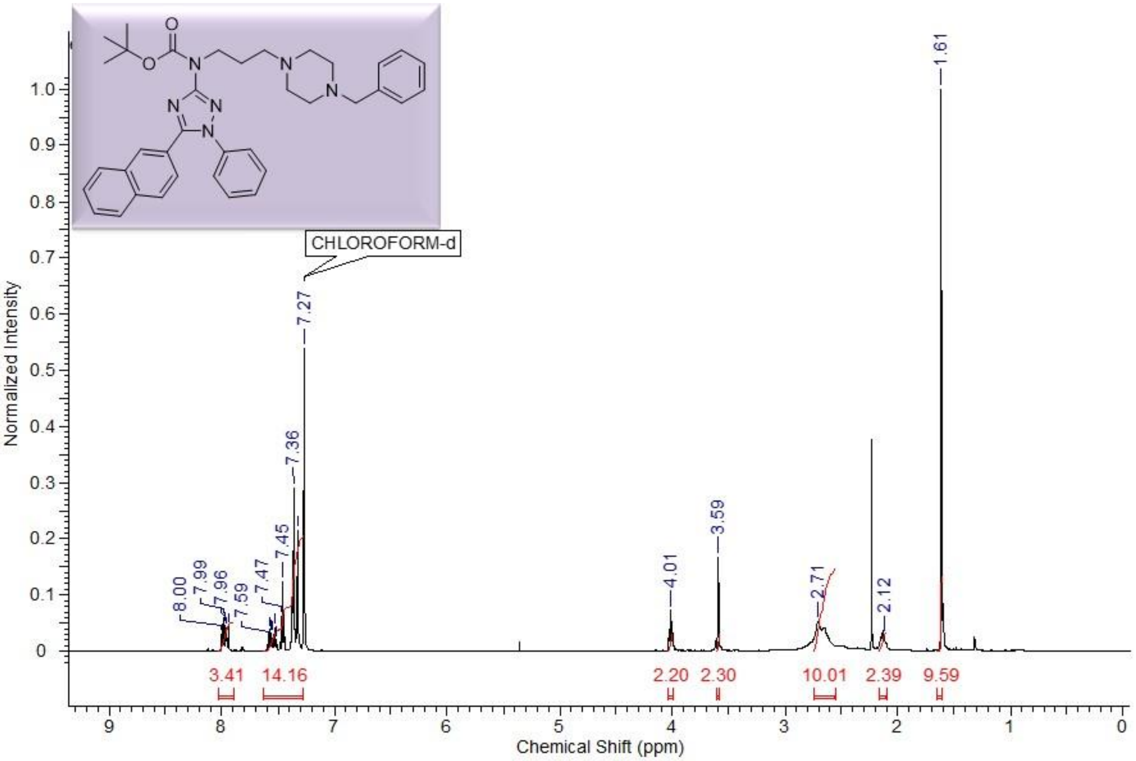

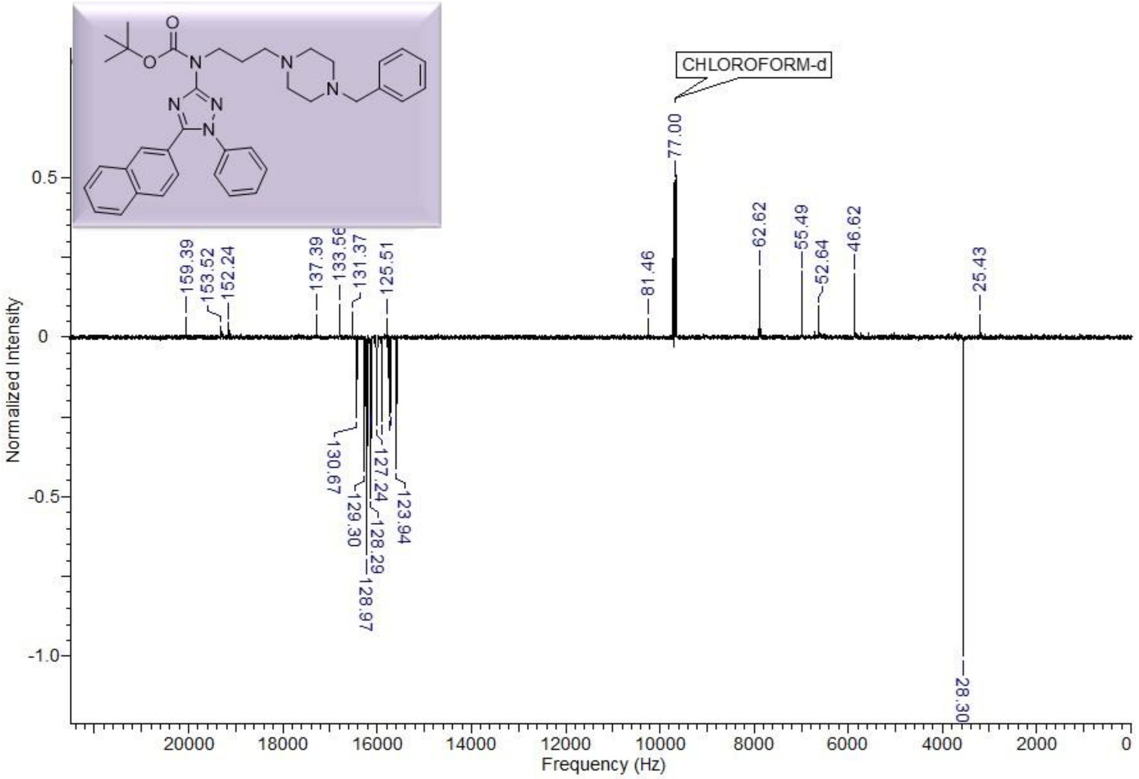

Figure S50: ^{1}H NMR (500 MHz) and ^{13}C NMR (125 MHz) spectra of compound **18s** in $CDCl_3$.

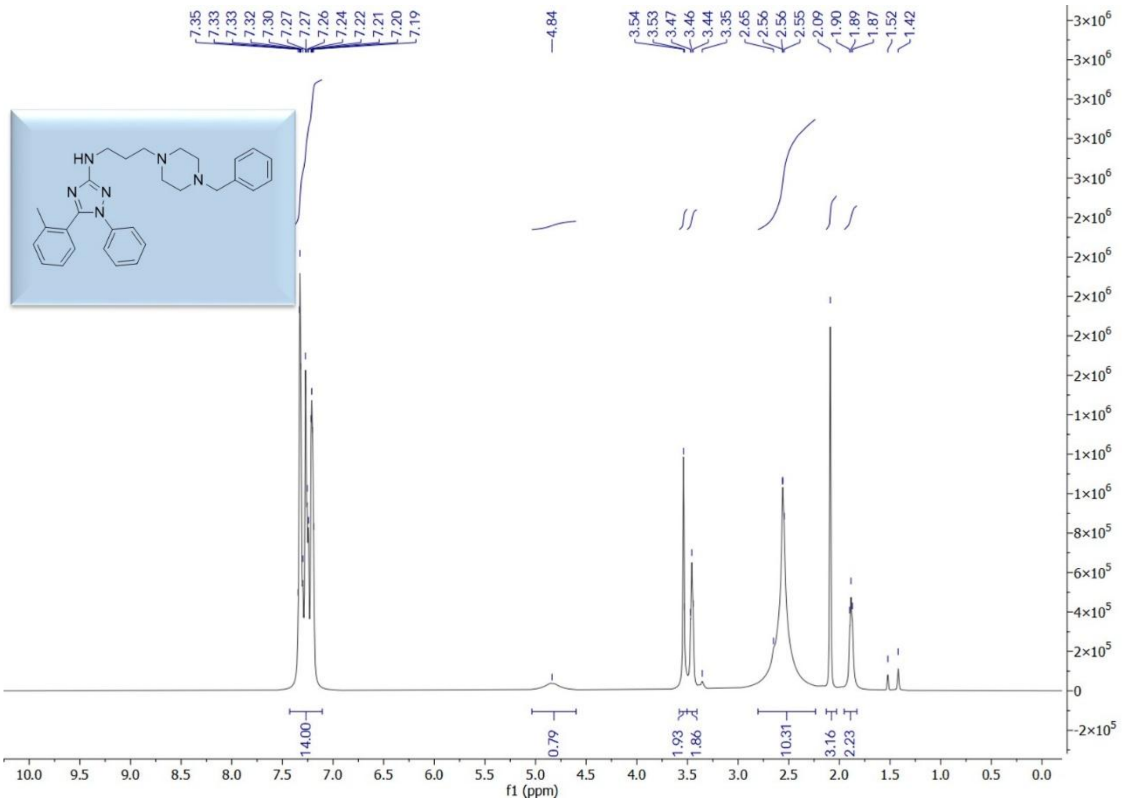
7.35
7.33
7.33
7.32
7.30
7.27
7.27
7.26
7.24
7.22
7.21
7.20
7.19
4.84
3.54
3.53
3.47
3.46
3.44
3.35
2.65
2.56
2.56
2.55
2.09
1.90
1.89
1.87
1.52
1.42
HN
N
N
N
N
N
14.00
0.79
1.93
1.86
10.31
3.16
2.23
f1 (ppm)

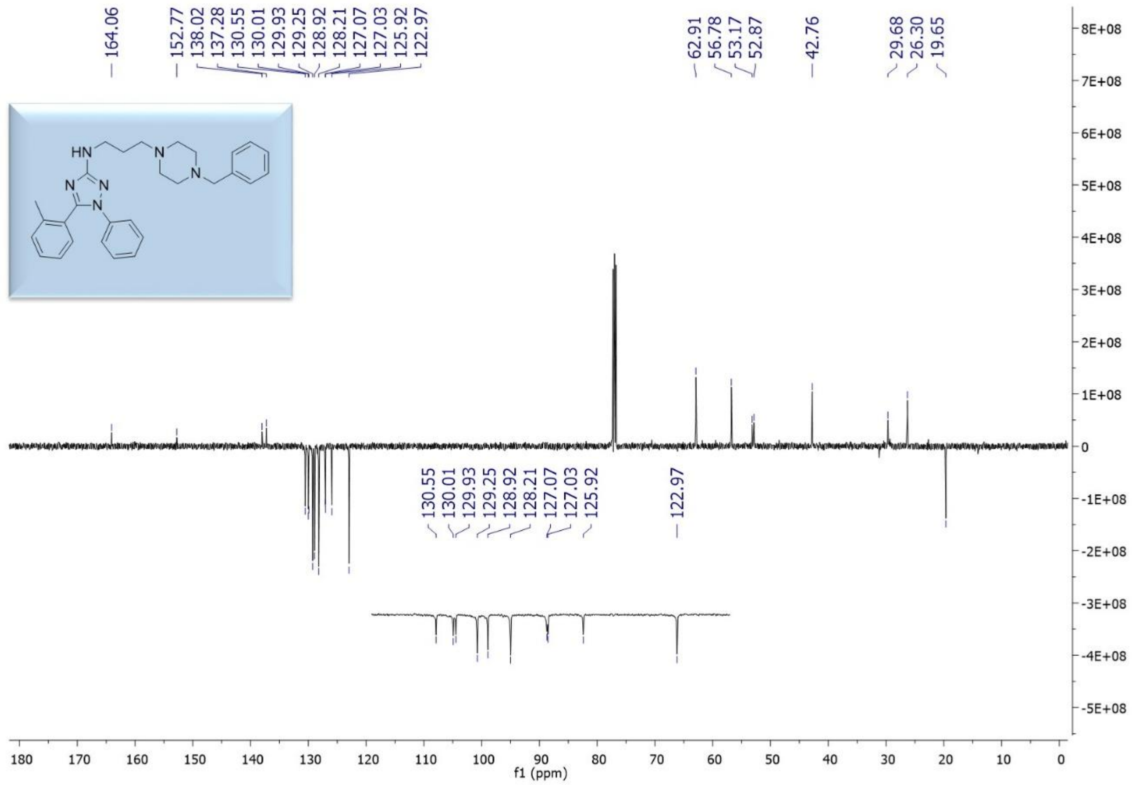
164.06
152.77
138.02
137.28
130.55
130.01
129.93
129.25
128.92
128.21
127.07
127.03
125.92
122.97
62.91
56.78
53.17
52.87
42.76
29.68
26.30
19.65
HN
N
N
N
N
N
f1 (ppm)

Figure S51: ^{1}H NMR (500 MHz) and ^{13}C NMR (125 MHz) spectra of compound **11a** in $CDCl_3$.

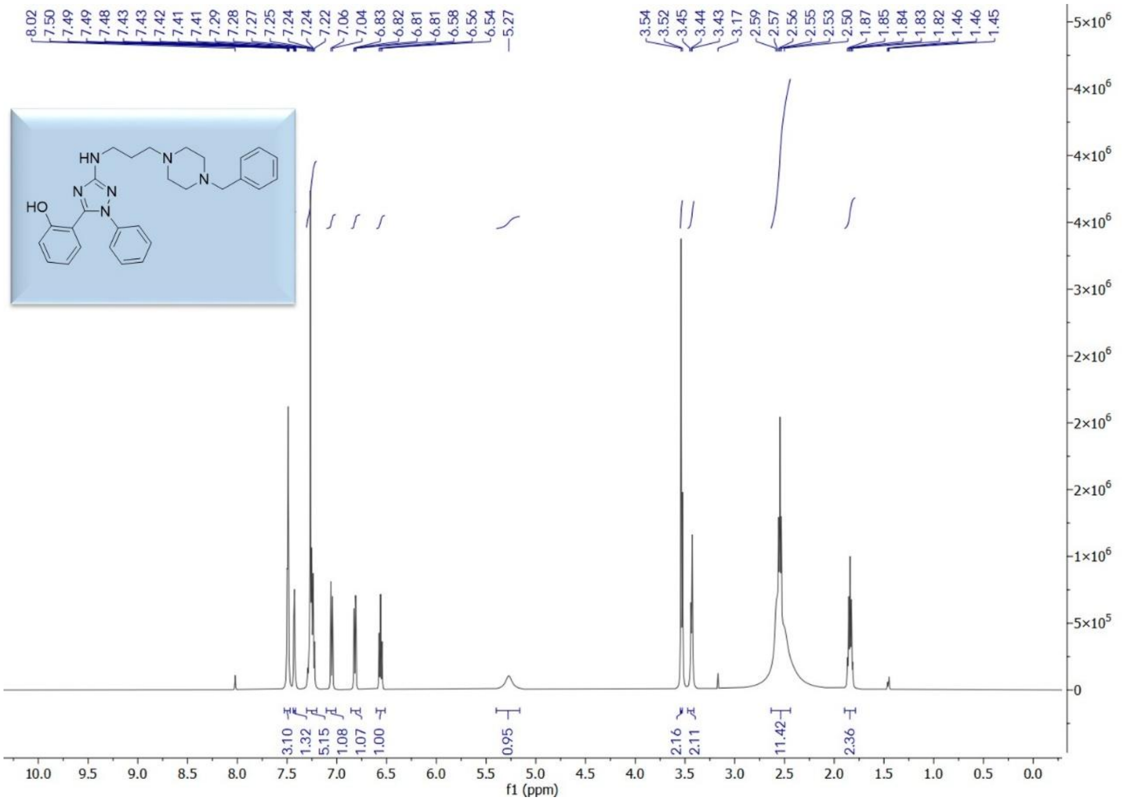

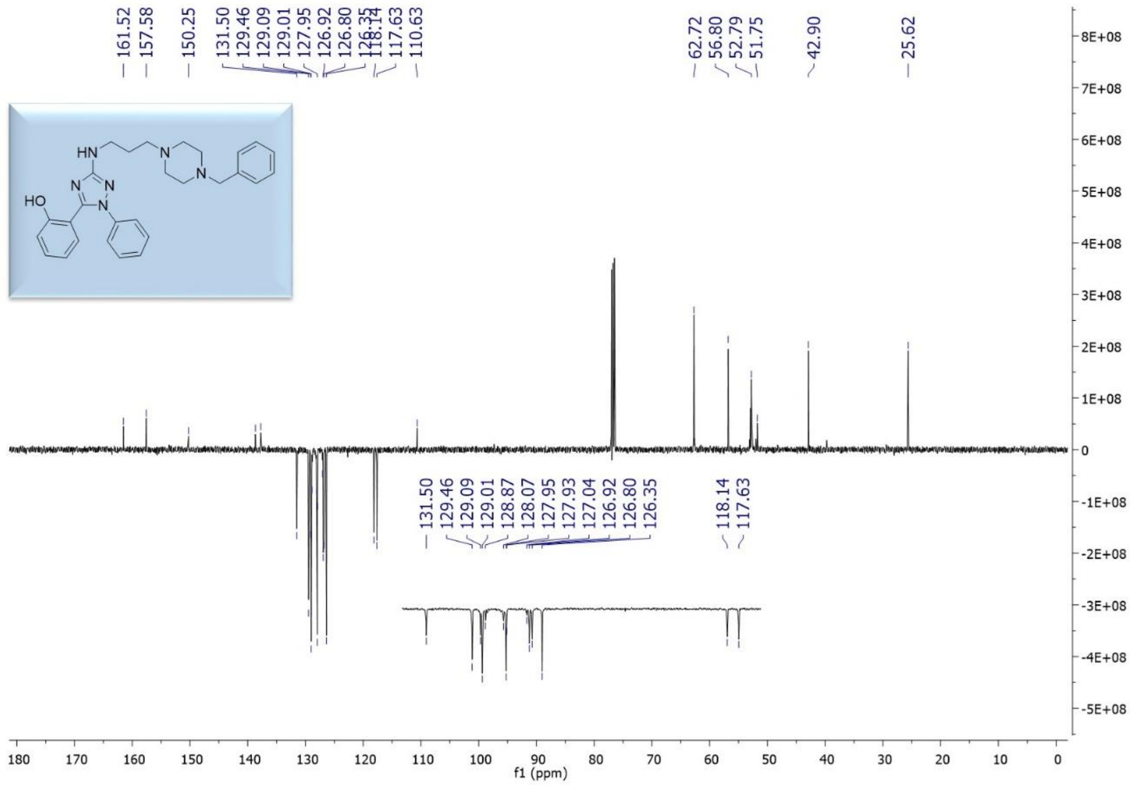

Figure S52: ^{1}H NMR (500 MHz) and ^{13}C NMR (125 MHz) spectra of compound **11b** in $CDCl_3$.

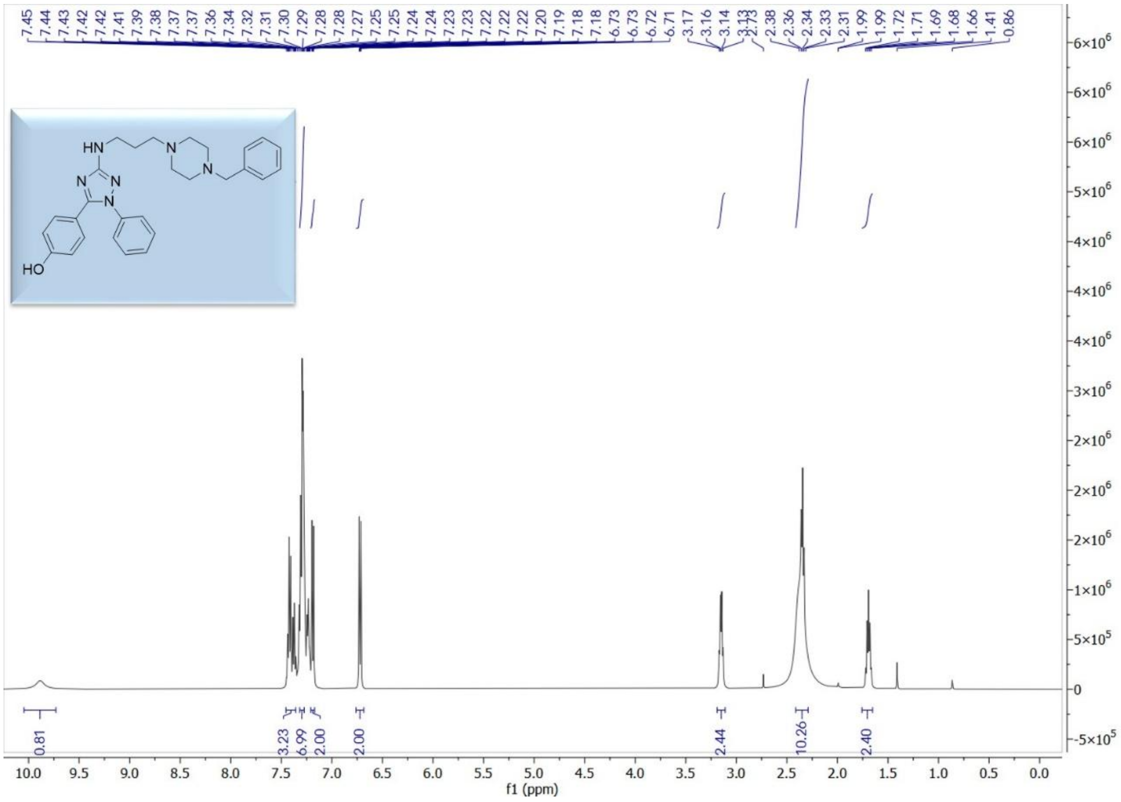

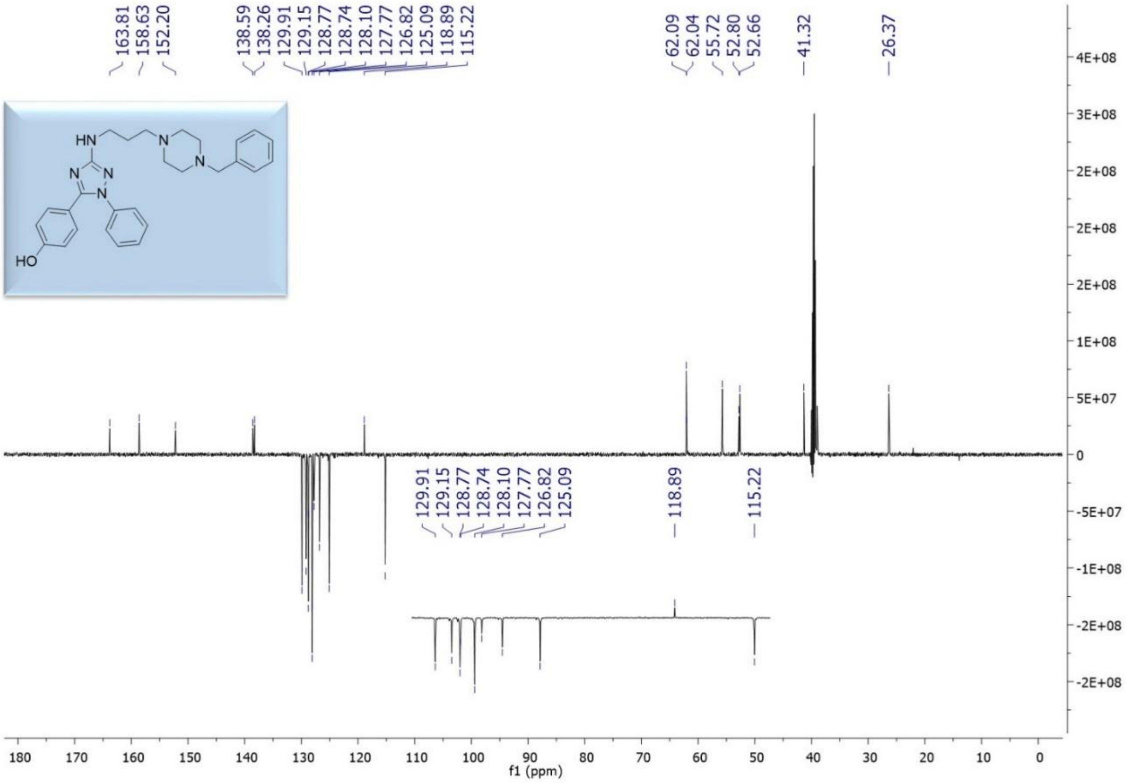

Figure S53: ^{1}H NMR (500 MHz) and ^{13}C NMR (125 MHz) spectra of compound **11c** in DMSO-d_6.

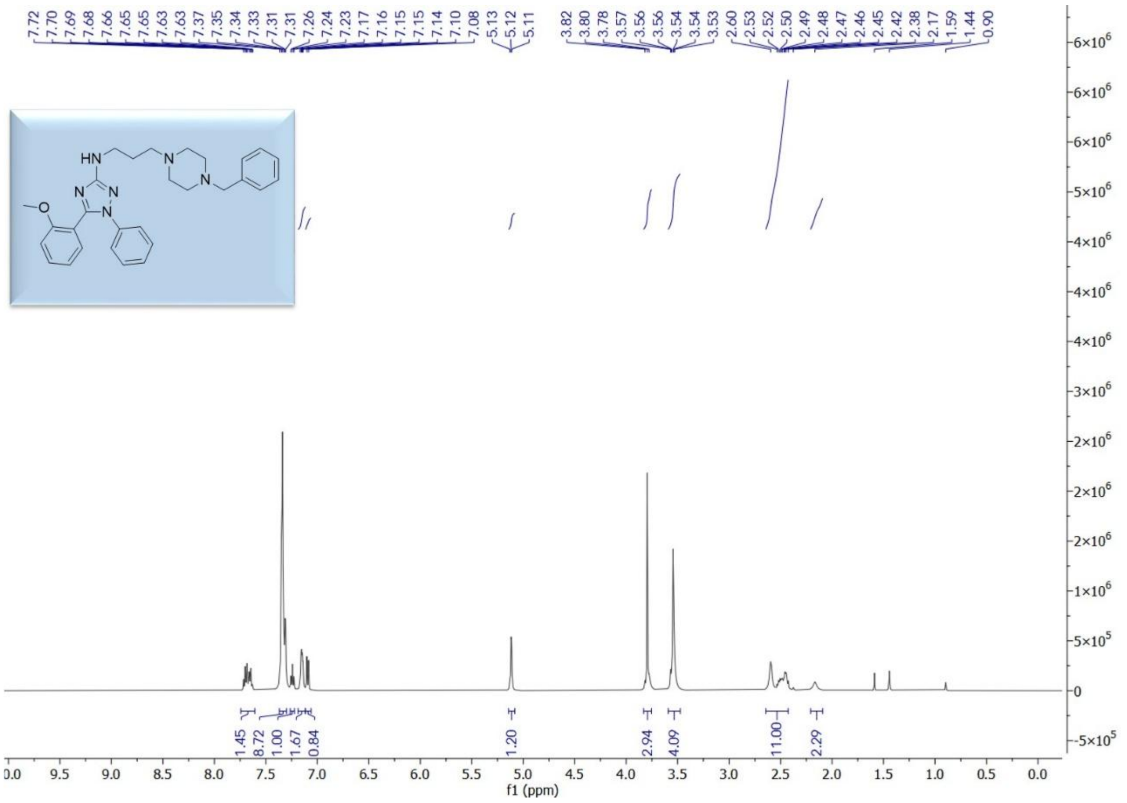

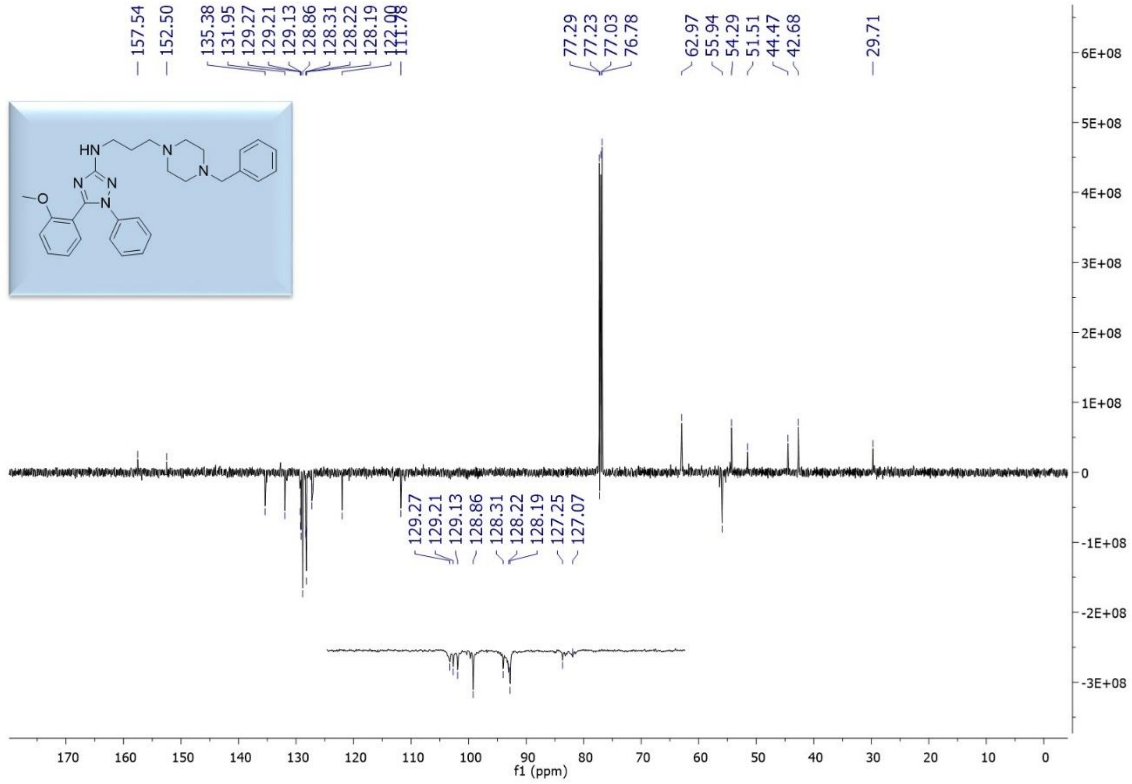

Figure S54: ^{1}H NMR (500 MHz) and ^{13}C NMR (125 MHz) spectra of compound **11d** in $CDCl_3$.

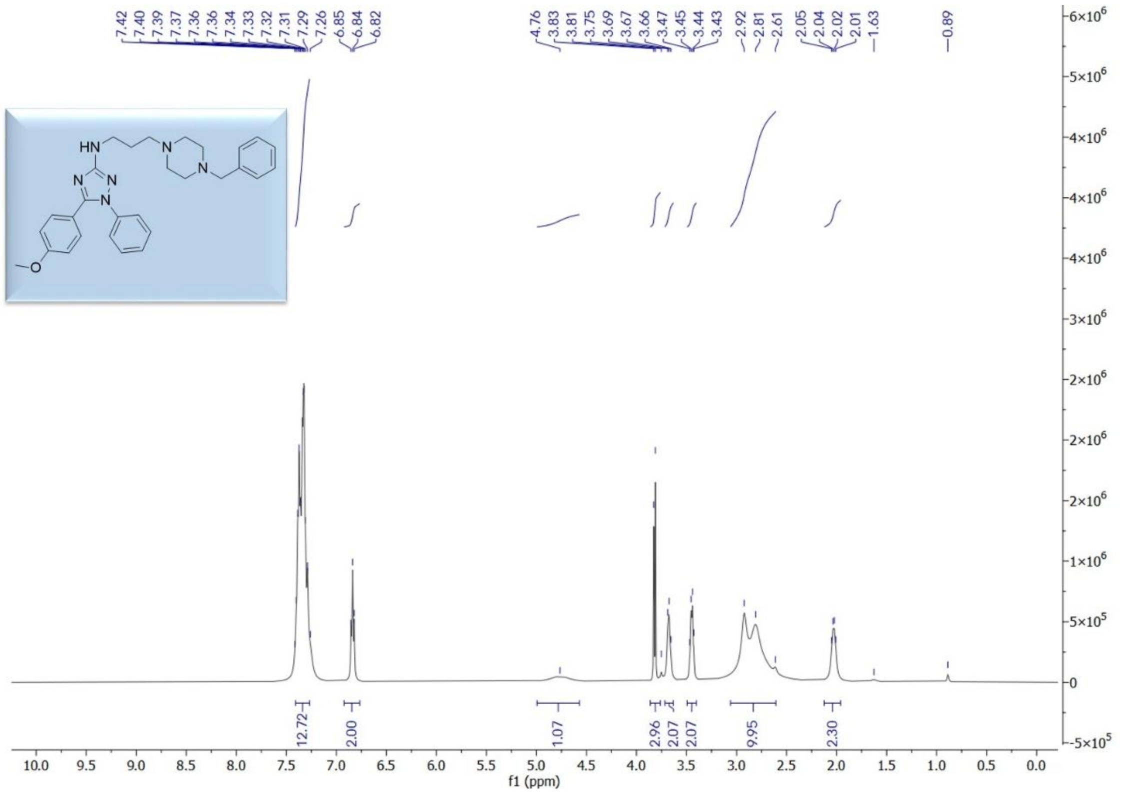

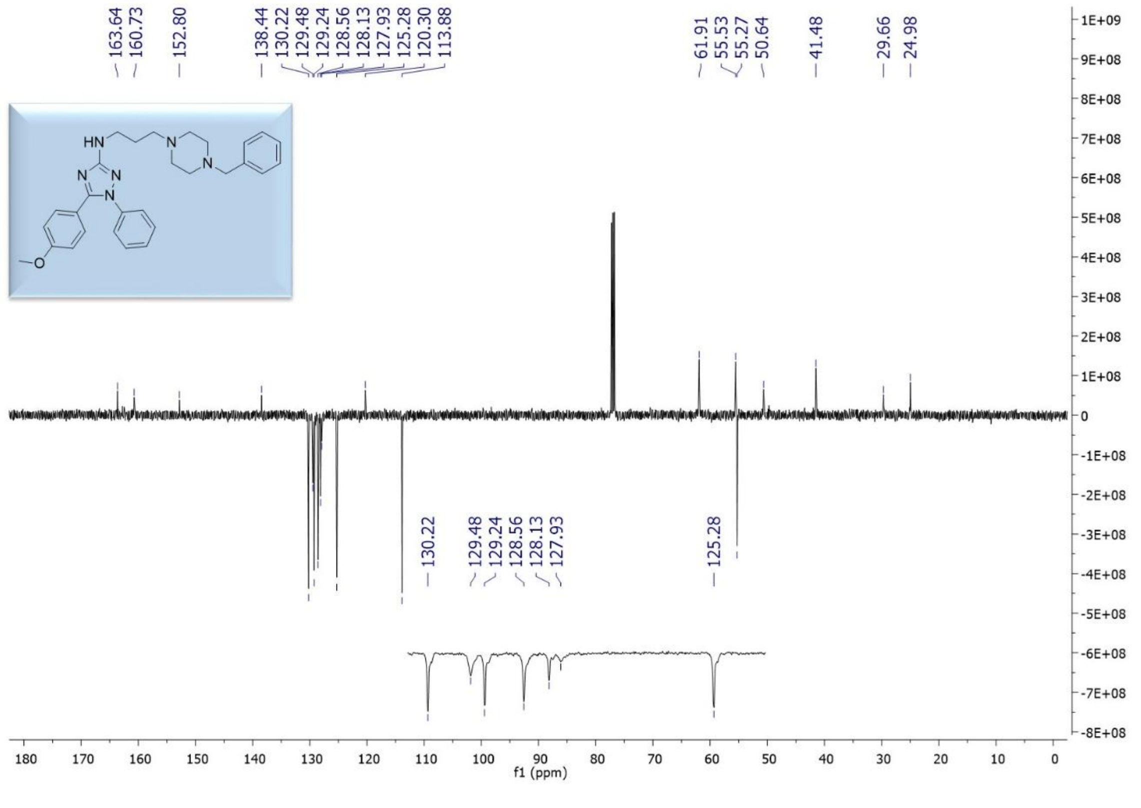

Figure S55: ^{1}H NMR (500 MHz) and ^{13}C NMR (125 MHz) spectra of compound **11e** in $CDCl_3$.

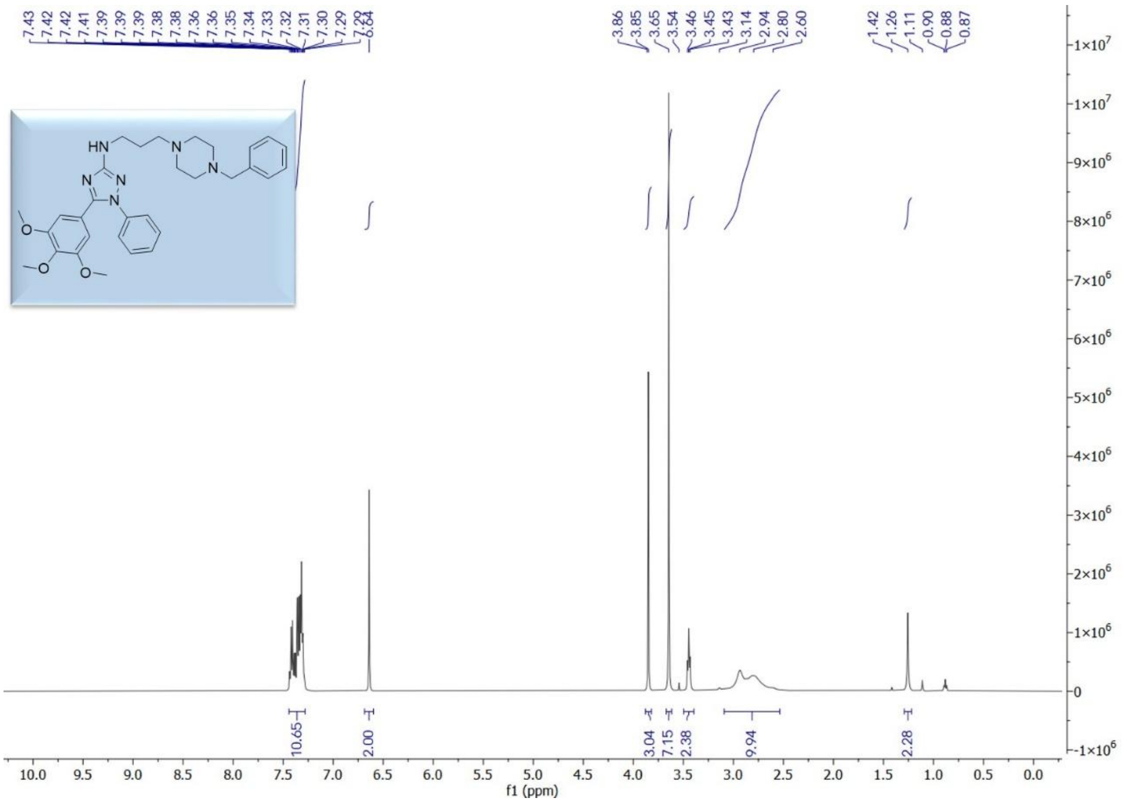

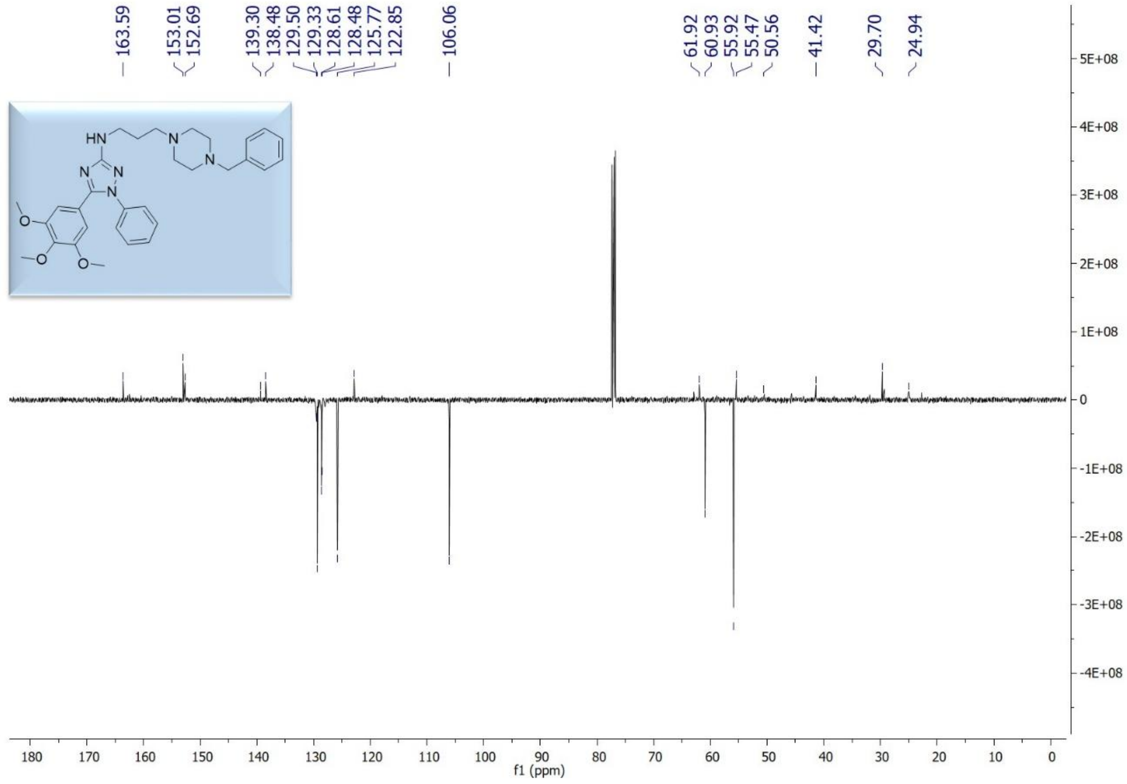

Figure S56: ^{1}H NMR (500 MHz) and ^{13}C NMR (125 MHz) spectra of compound **11f** in $CDCl_3$.

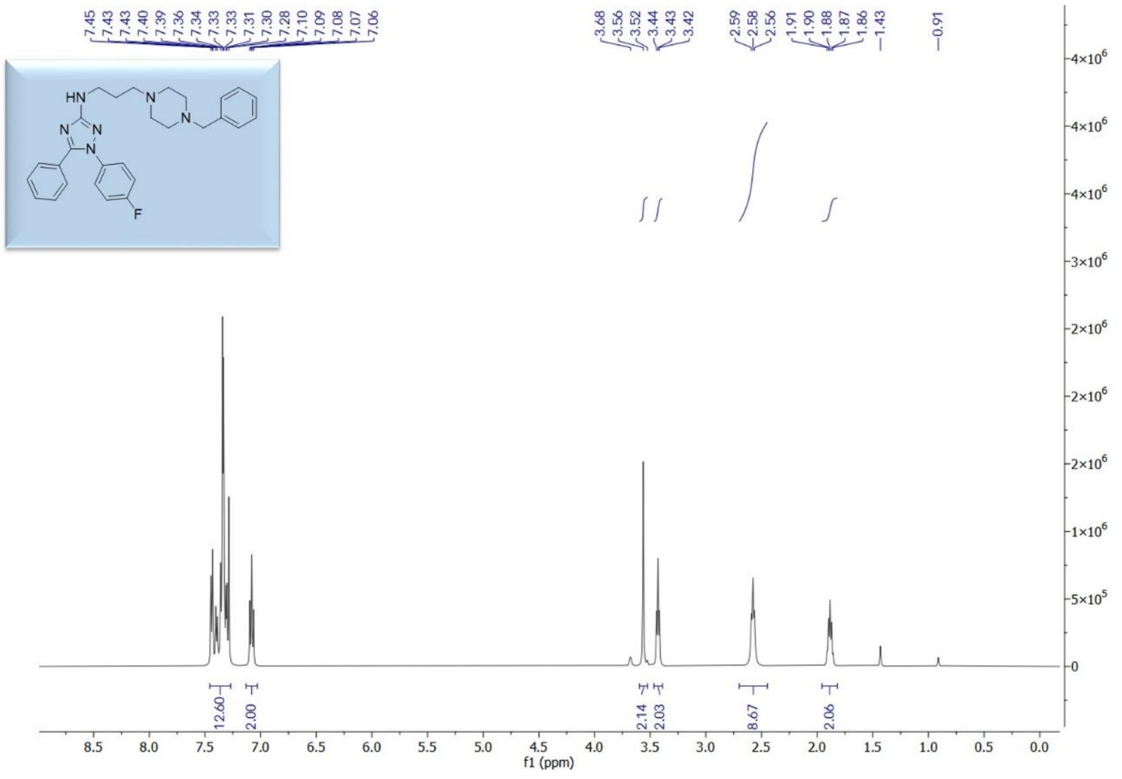

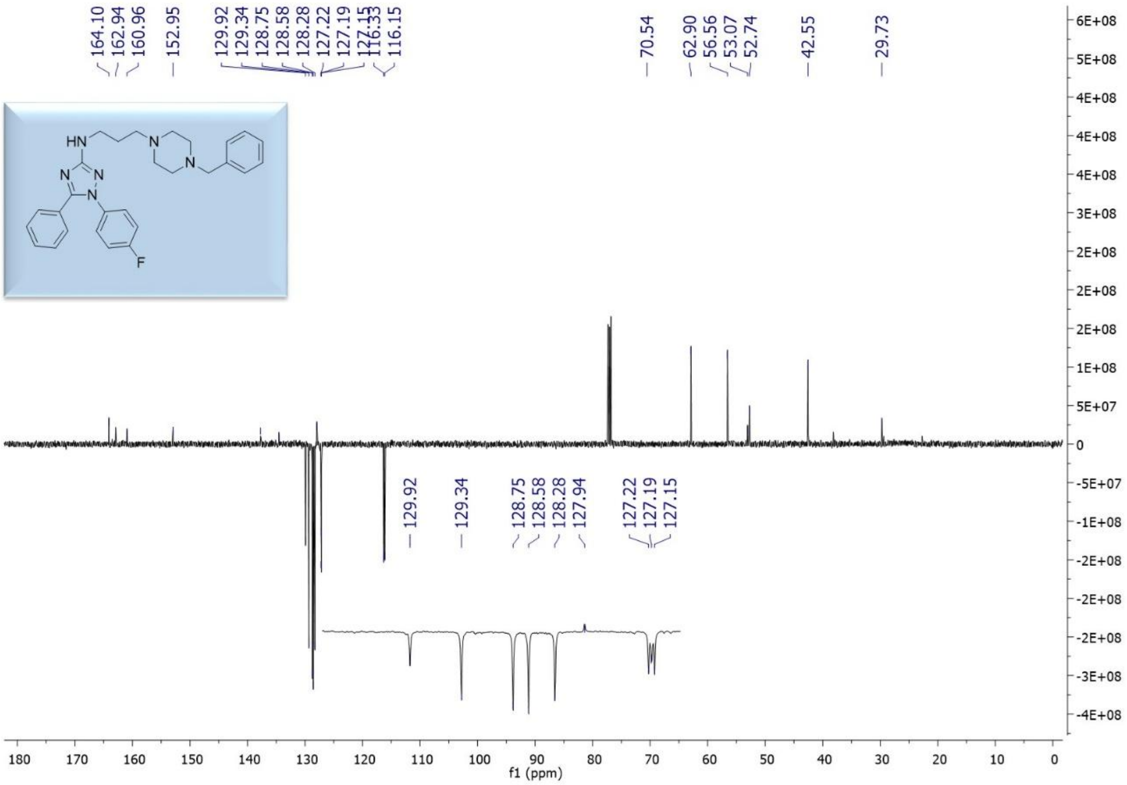

Figure S57: ^{1}H NMR (500 MHz) and ^{13}C NMR (125 MHz) spectra of compound **11g** in $CDCl_3$.

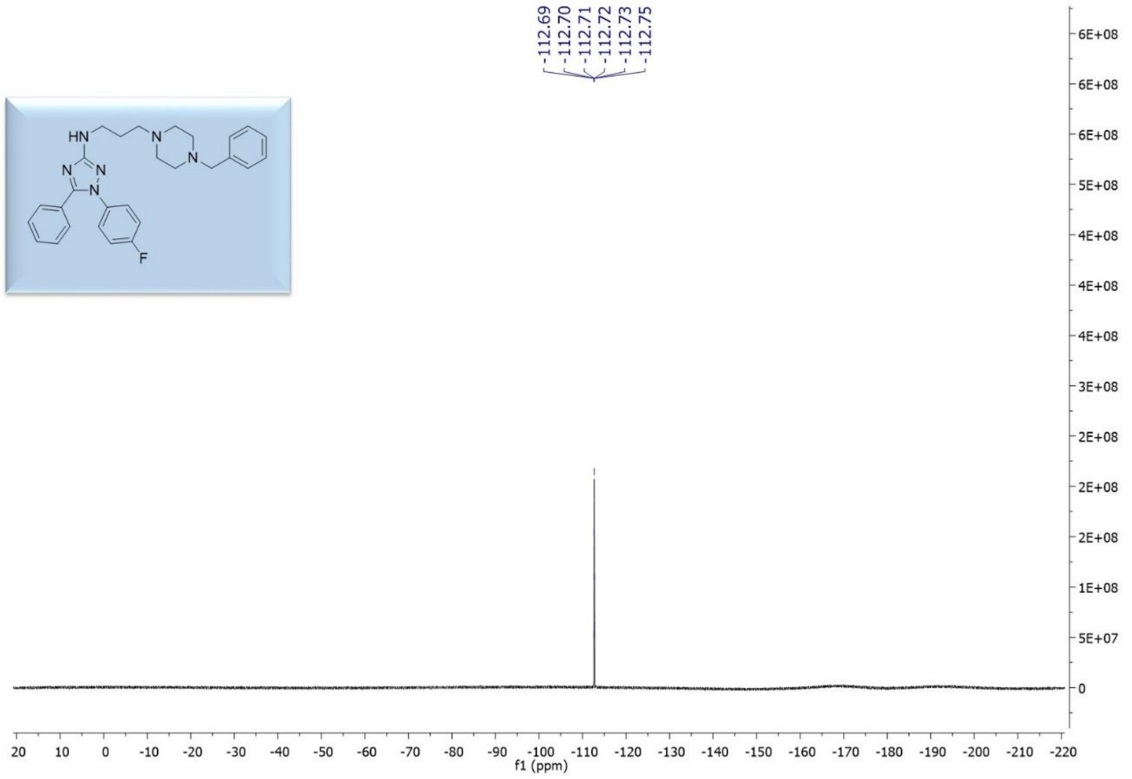

Figure S58: ^{19}F NMR (470 MHz) spectra of compound **11g** in $CDCl_3$.

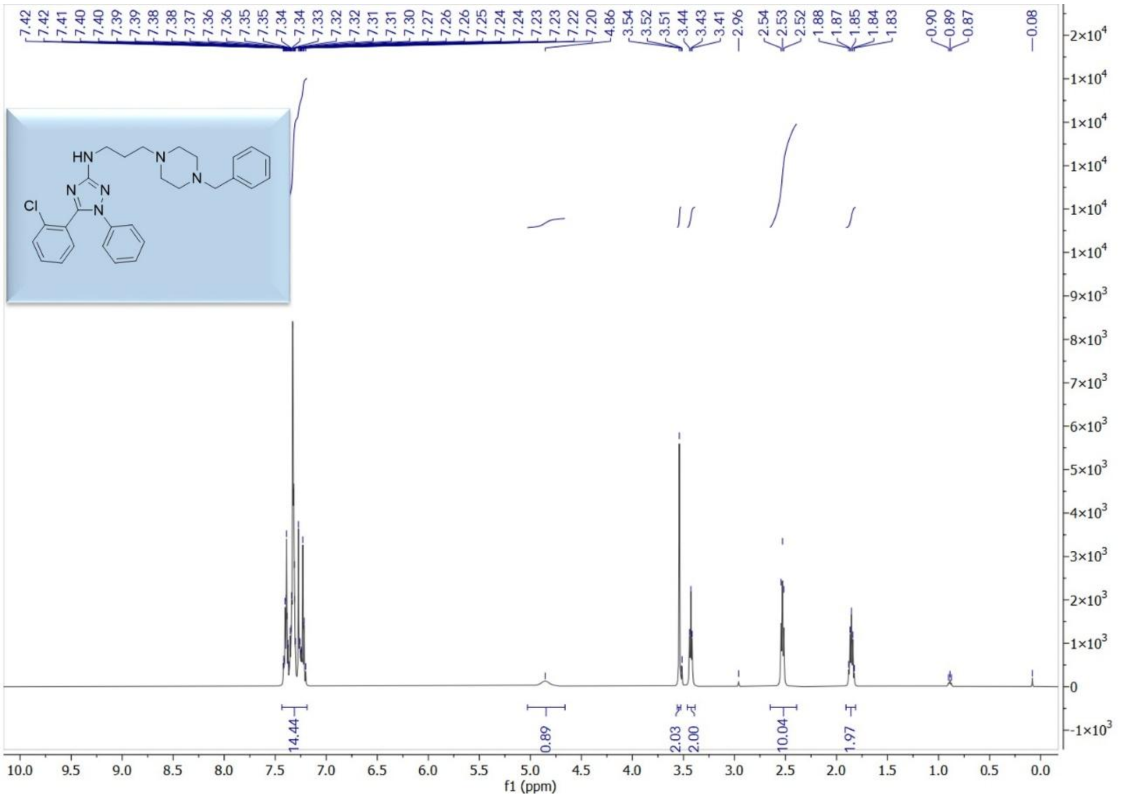

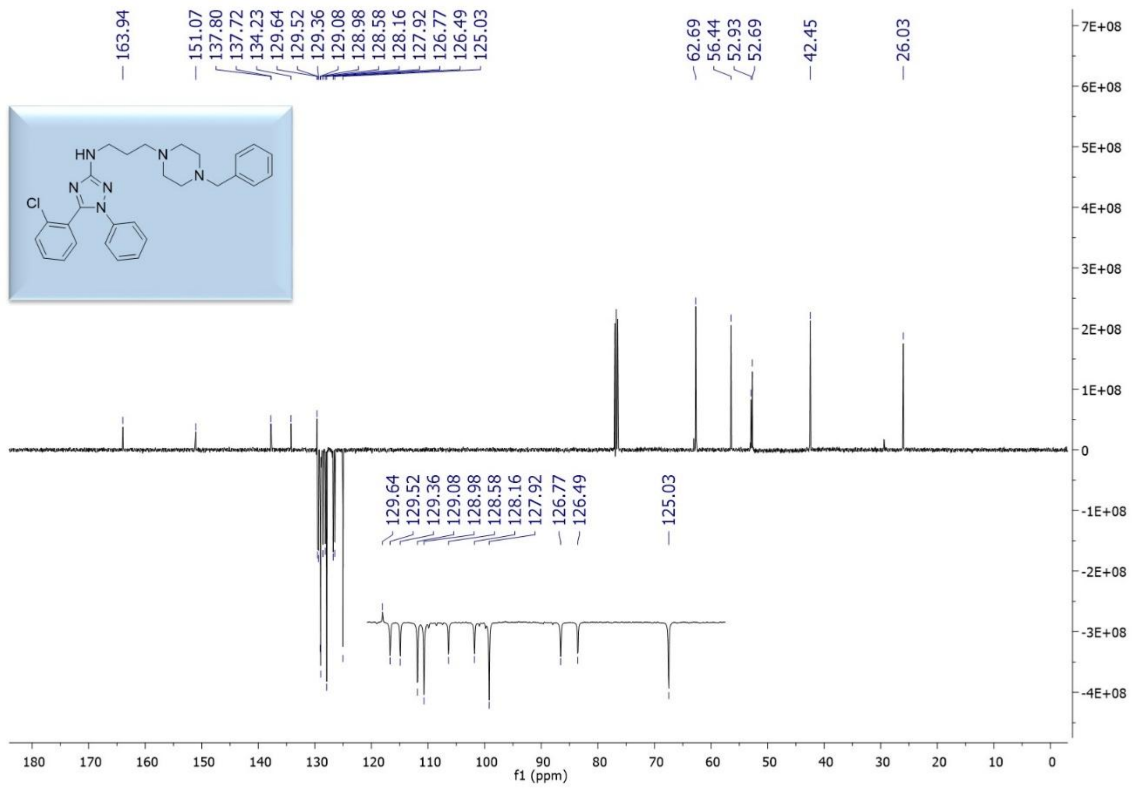

Figure S59: ^{1}H NMR (500 MHz) and ^{13}C NMR (125 MHz) spectra of compound **11h** in $CDCl_3$.

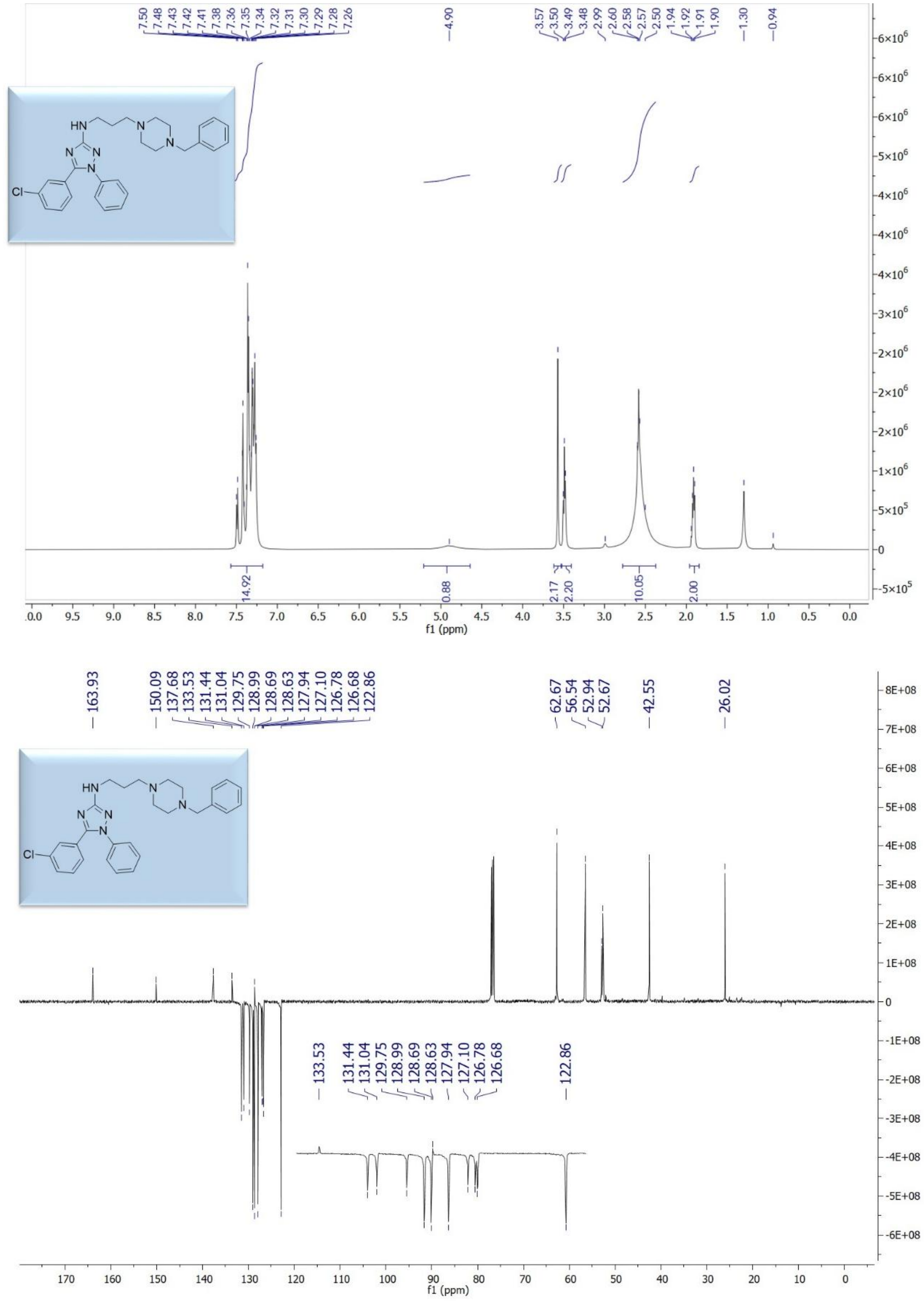

Figure S60: ^{1}H NMR (500 MHz) and ^{13}C NMR (125 MHz) spectra of compound **11i** in $CDCl_3$.

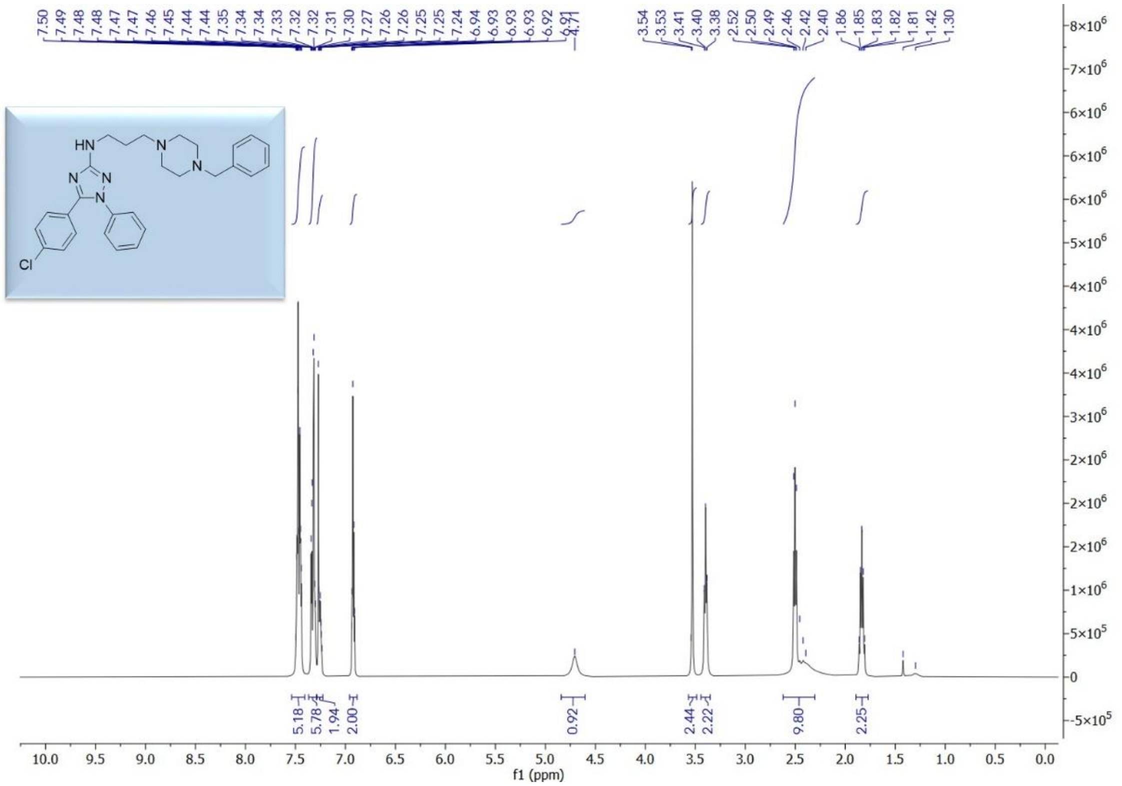

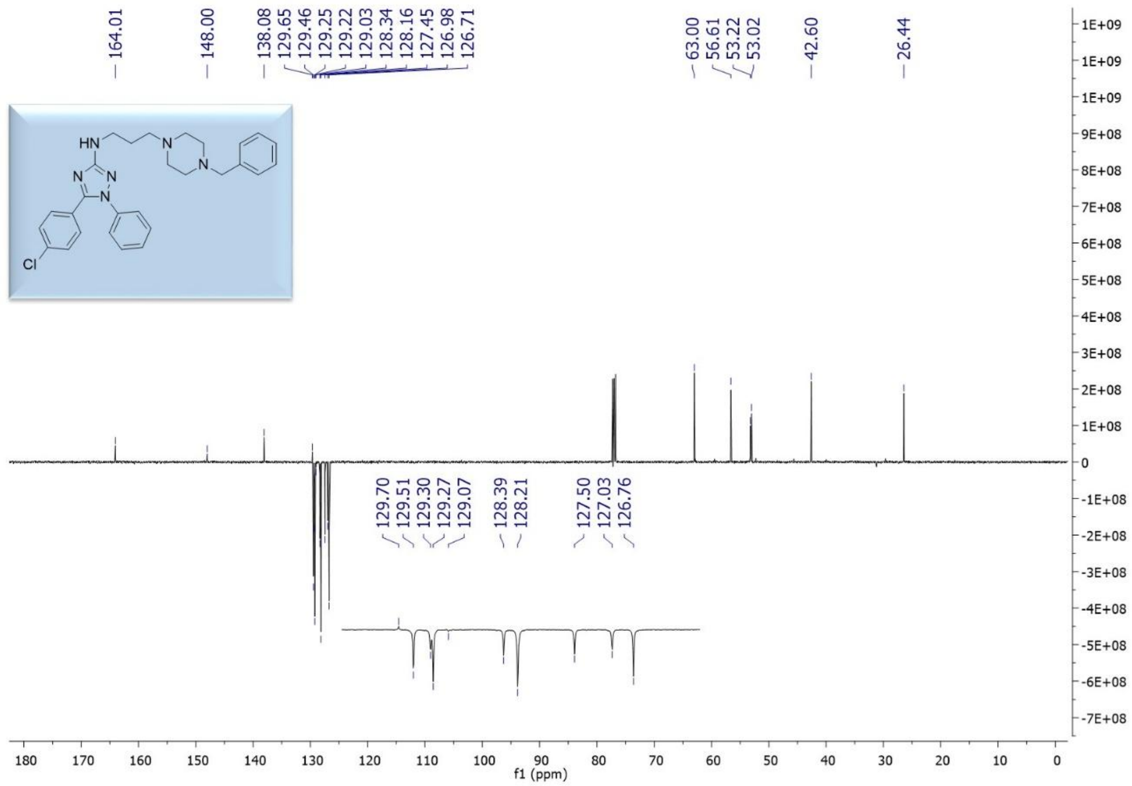

Figure S61: ^{1}H NMR (500 MHz) and ^{13}C NMR (125 MHz) spectra of compound **11j** in $CDCl_3$.

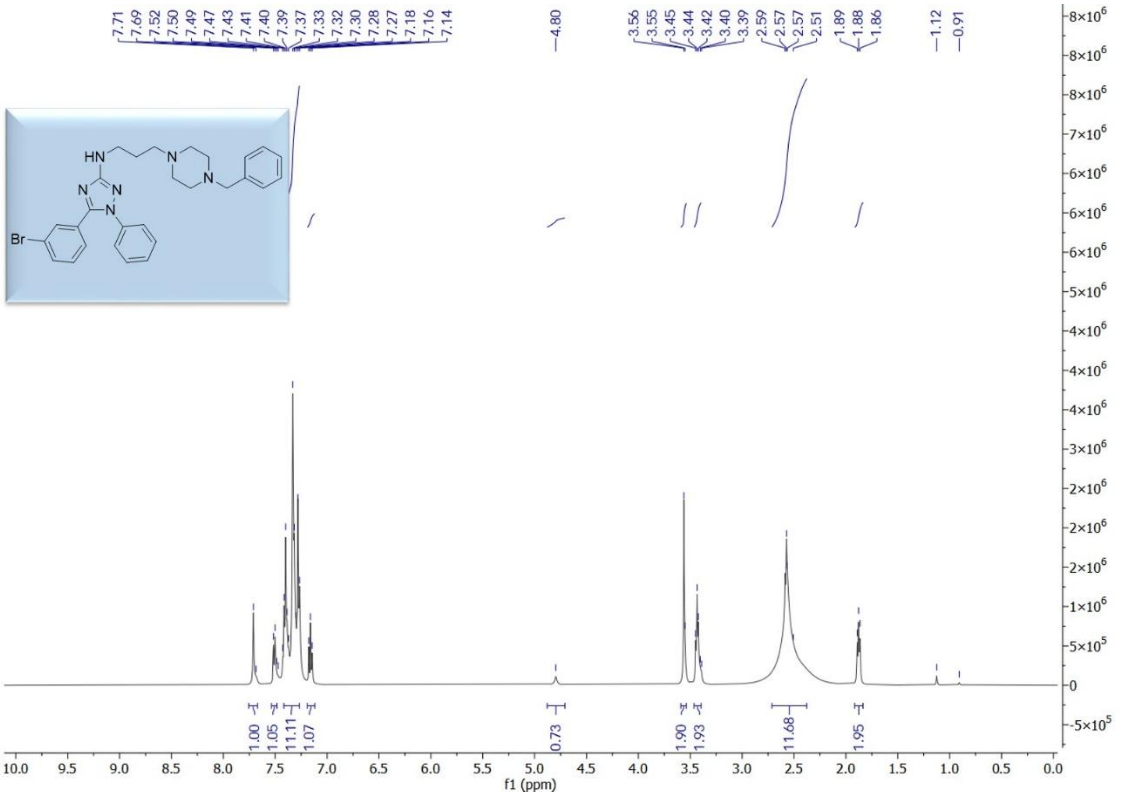

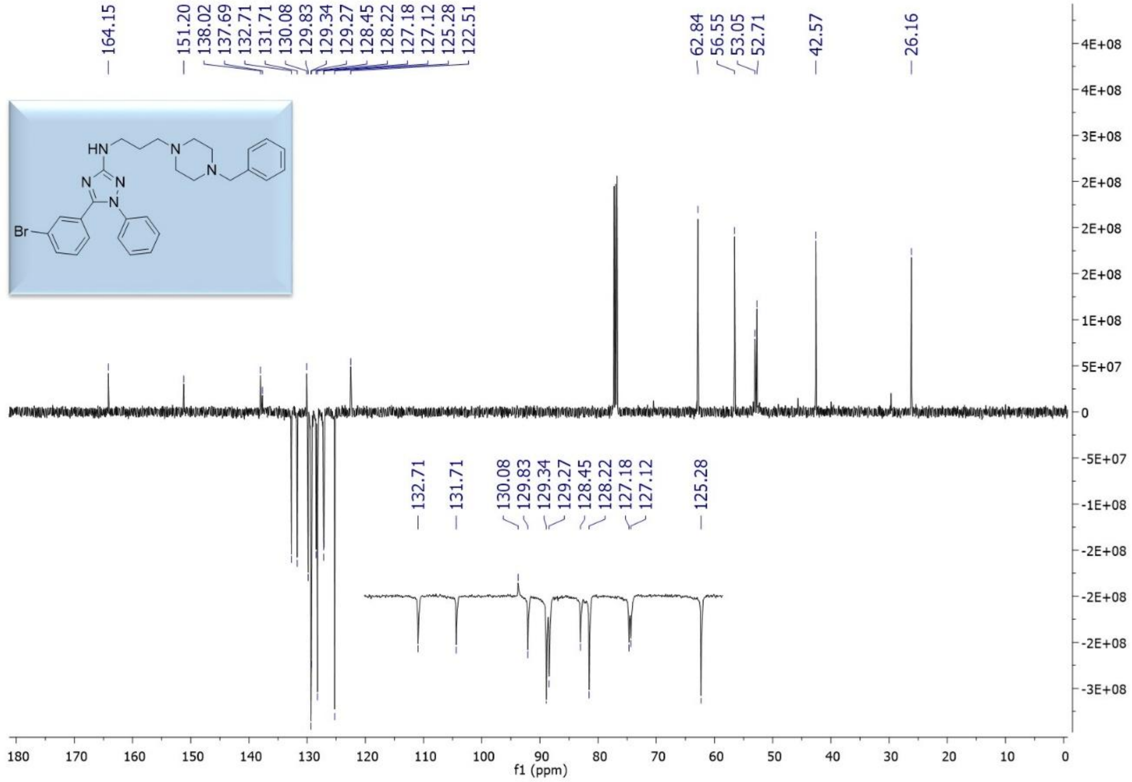

Figure S62: ^{1}H NMR (500 MHz) and ^{13}C NMR (125 MHz) spectra of compound **11k** in $CDCl_3$.

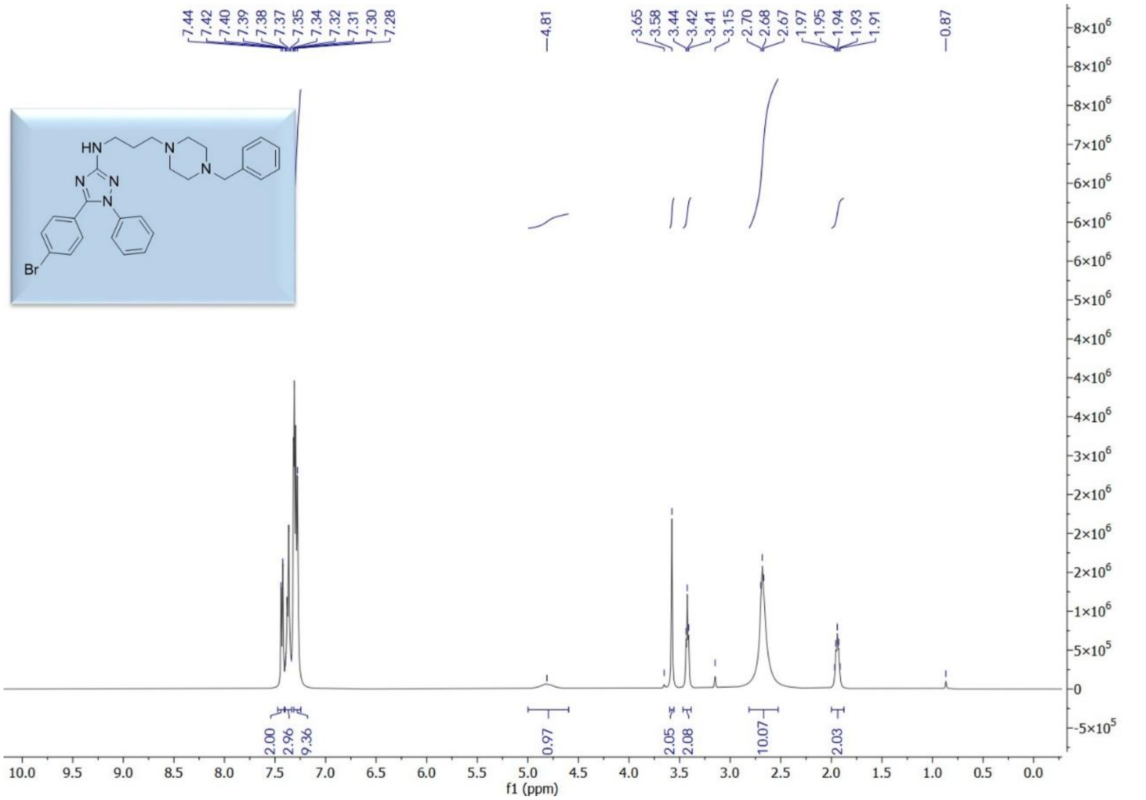

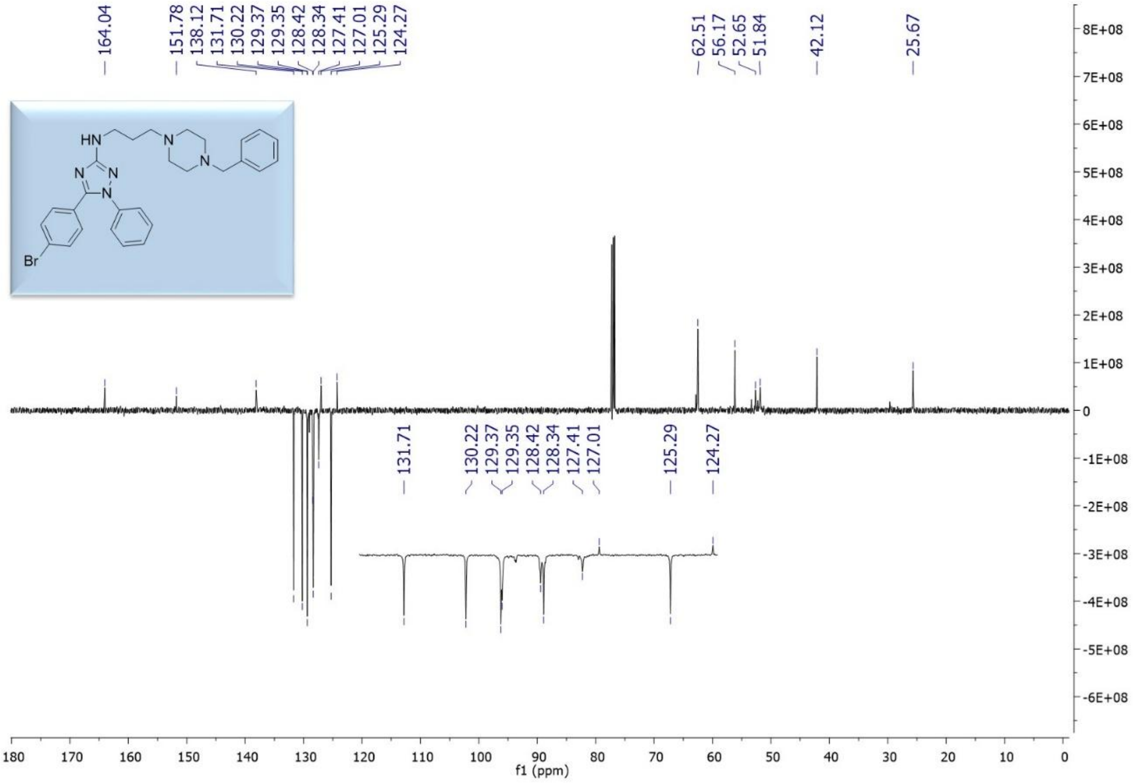

Figure S63: ^{1}H NMR (500 MHz) and ^{13}C NMR (125 MHz) spectra of compound **11l** in $CDCl_3$.

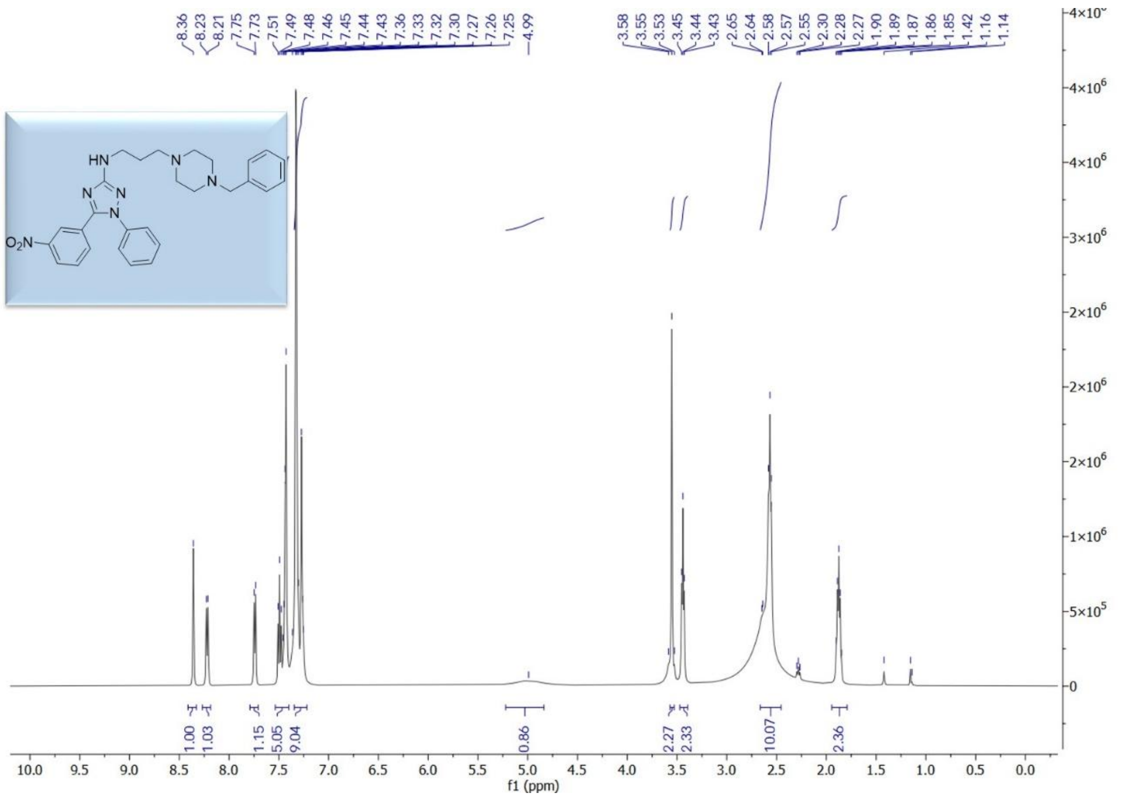

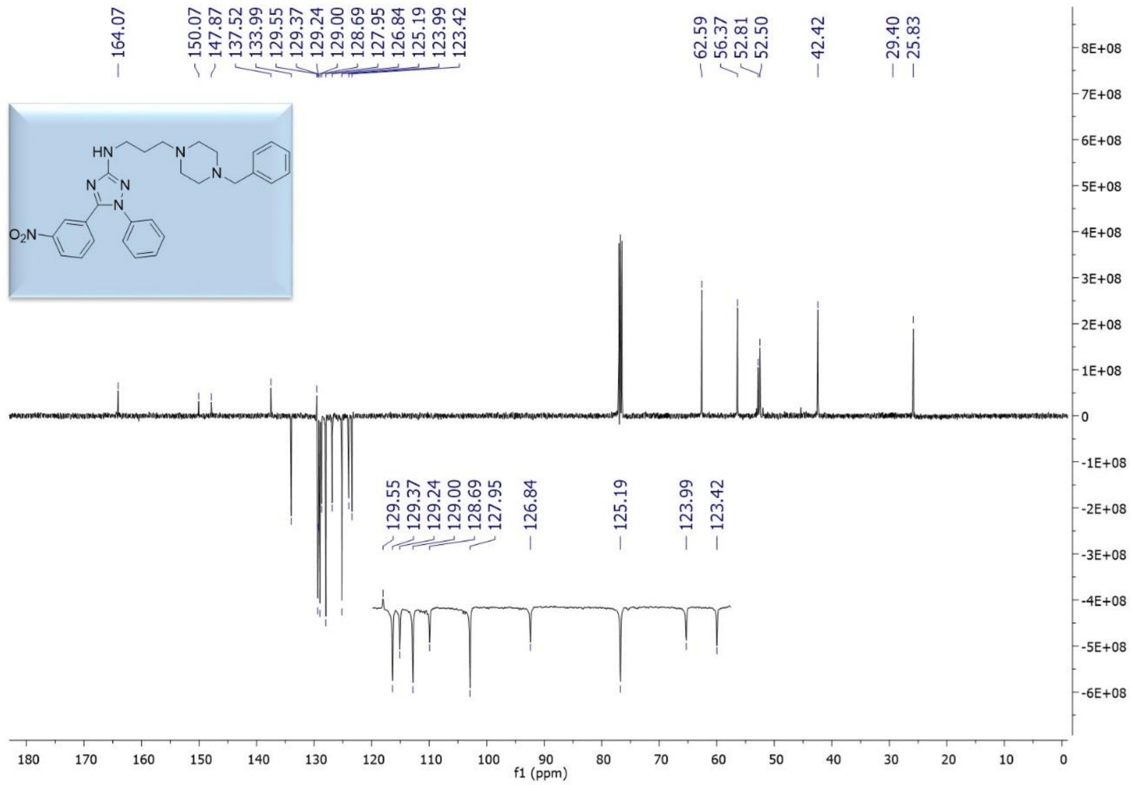

Figure S64: ^{1}H NMR (500 MHz) and ^{13}C NMR (125 MHz) spectra of compound **11m** in $CDCl_3$.

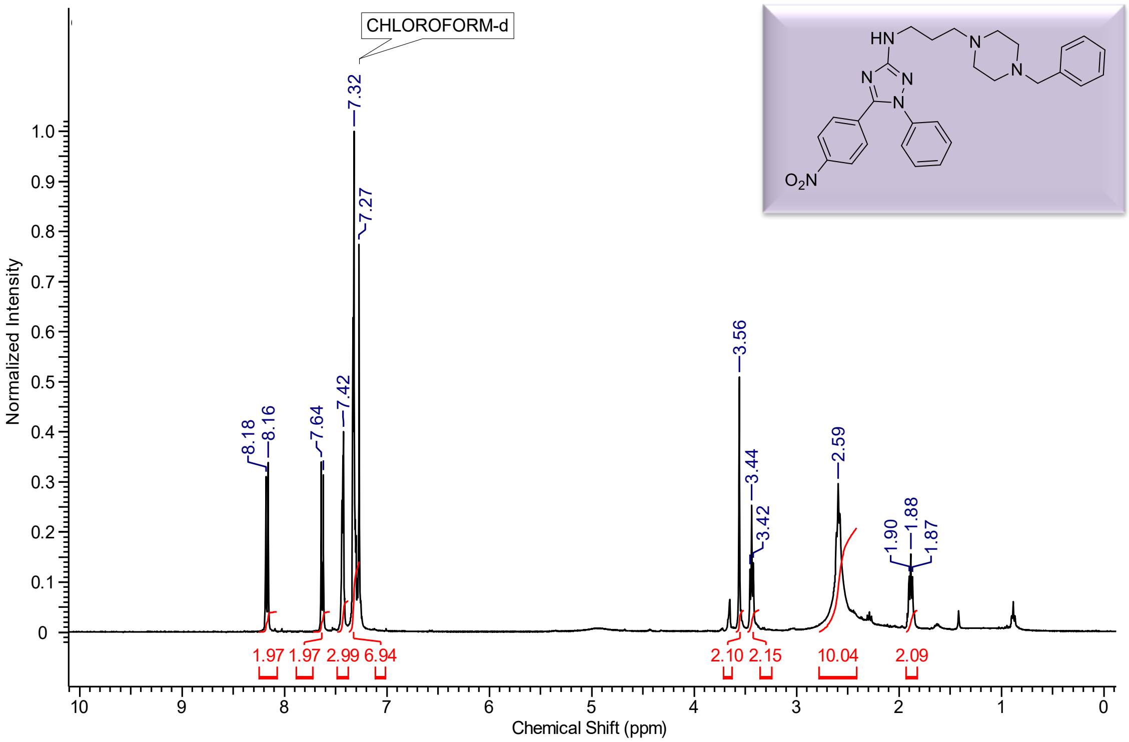

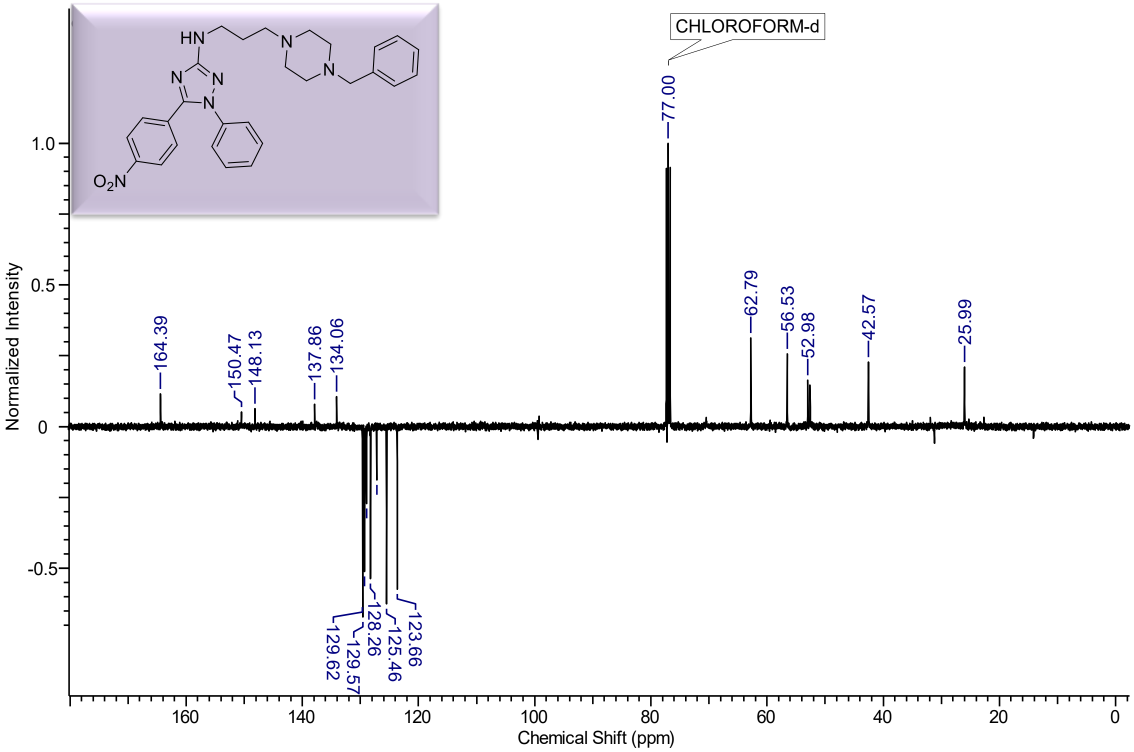

Figure S65: ^{1}H NMR (500 MHz) and ^{13}C NMR (125 MHz) spectra of compound **11n** in $CDCl_3$.

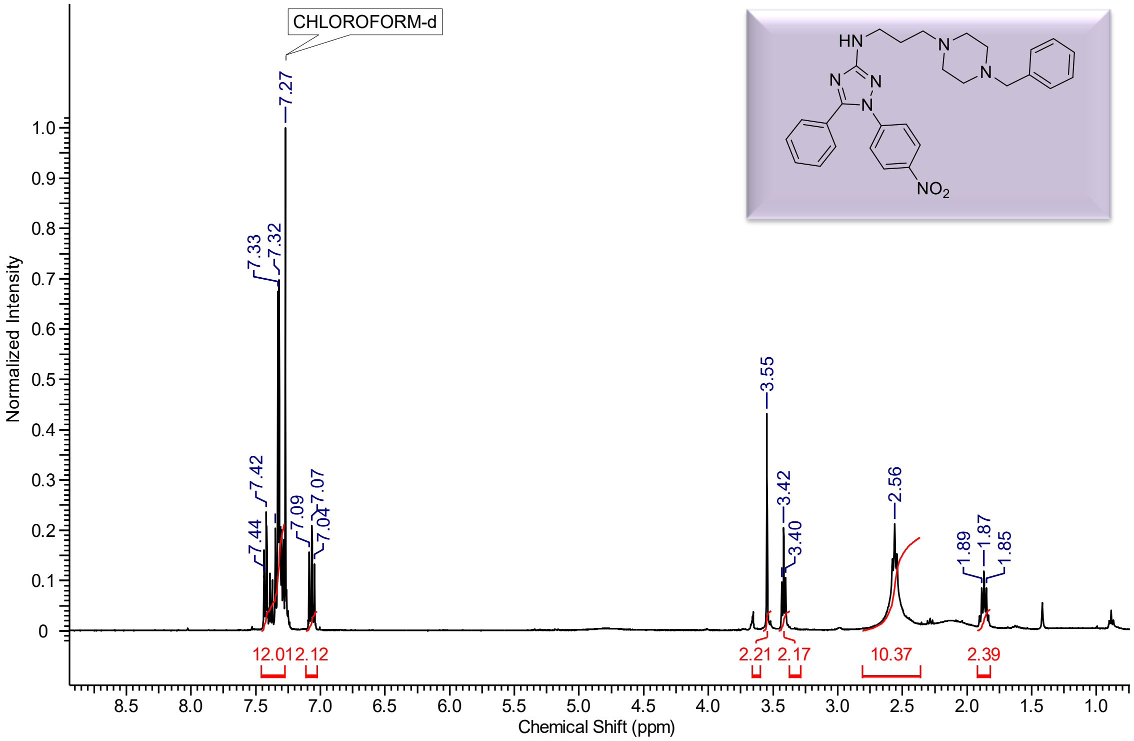

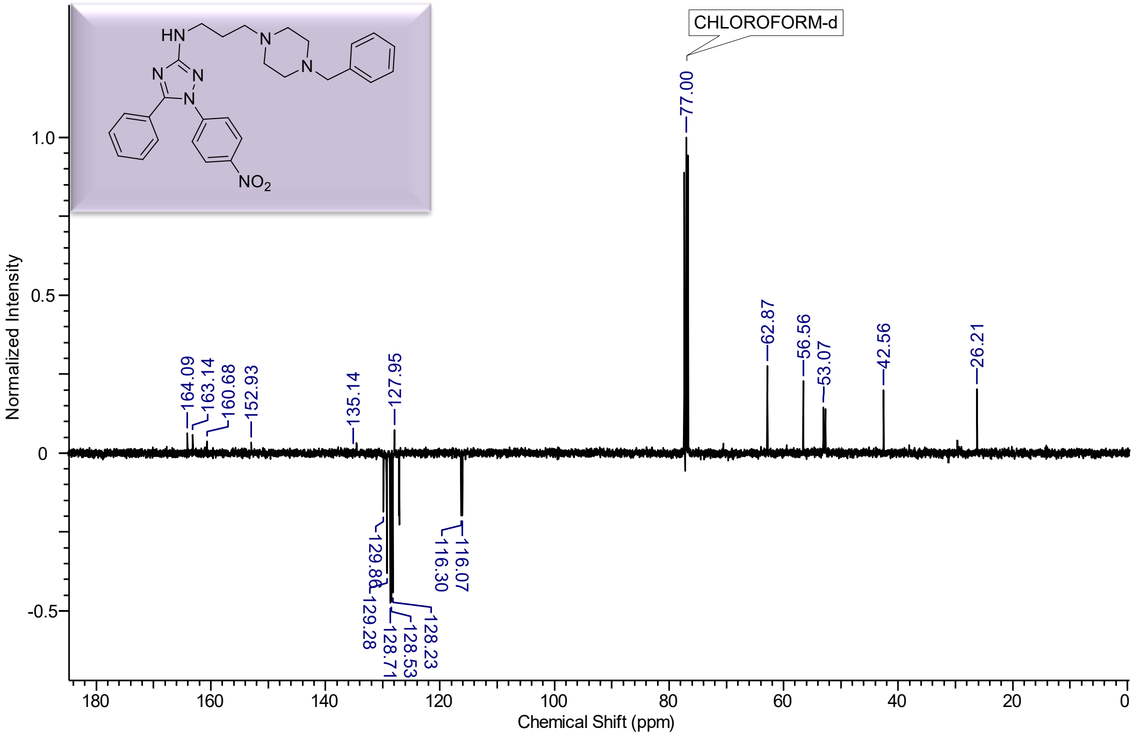

Figure S66: ^{1}H NMR (500 MHz) and ^{13}C NMR (125 MHz) spectra of compound **11o** in $CDCl_3$.

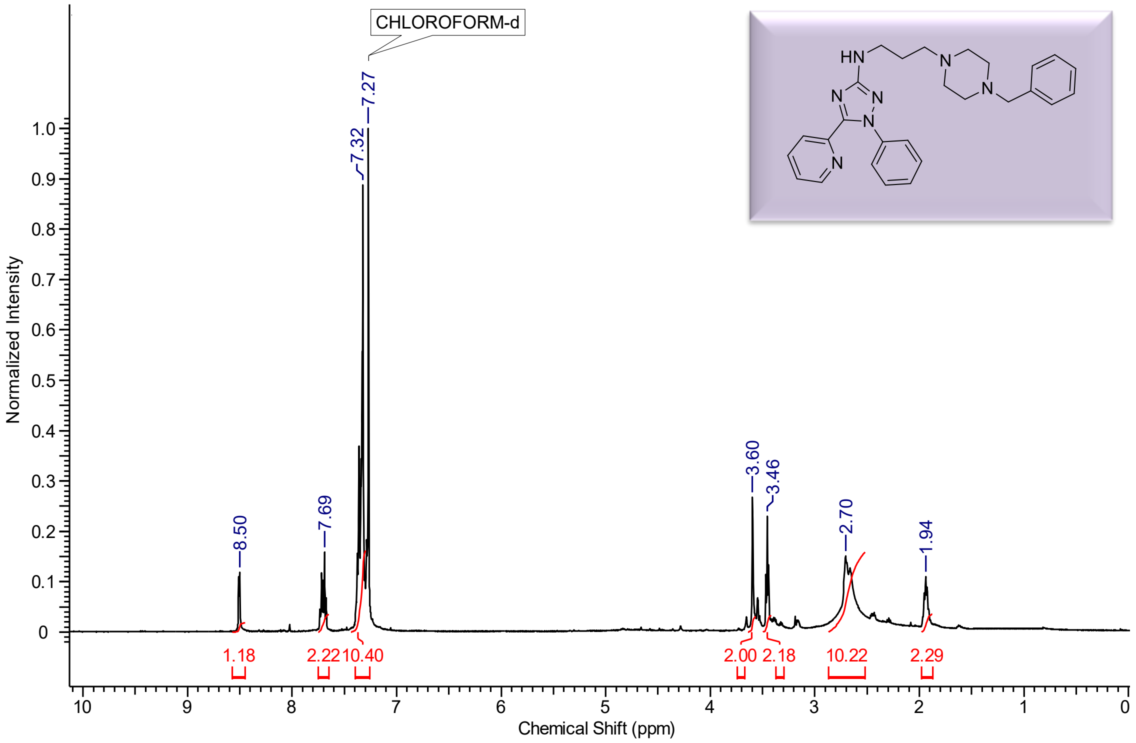

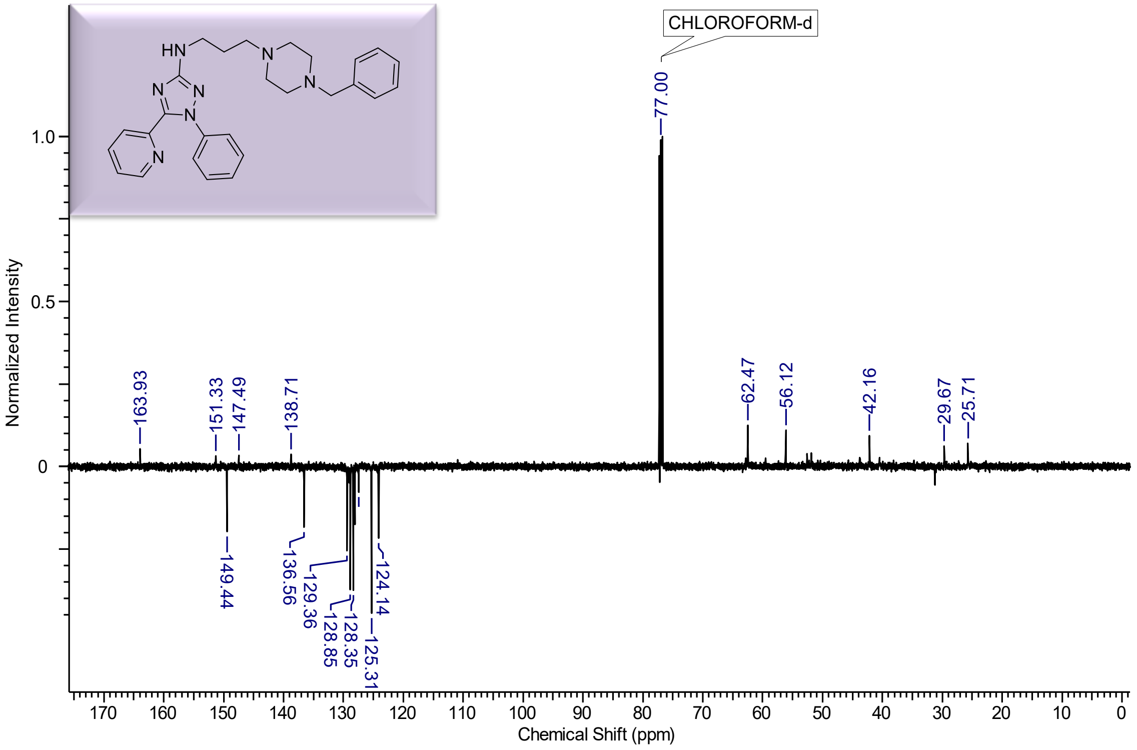

Figure S67: ^{1}H NMR (500 MHz) and ^{13}C NMR (125 MHz) spectra of compound **11p** in $CDCl_3$.

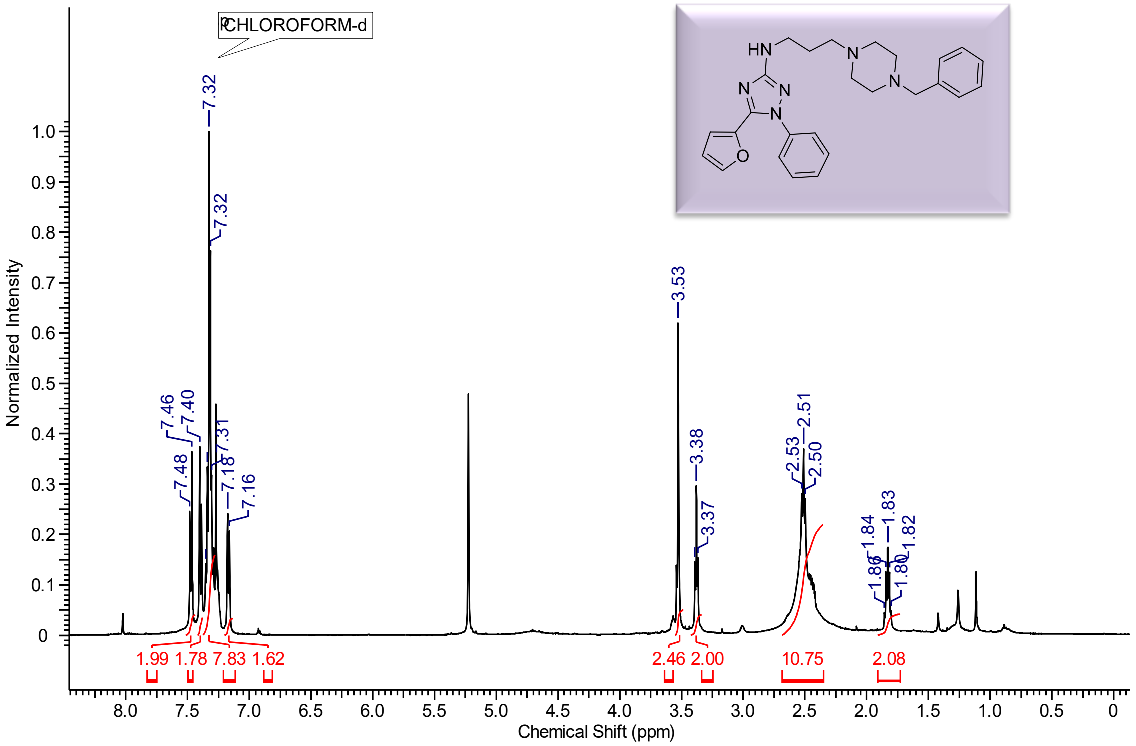

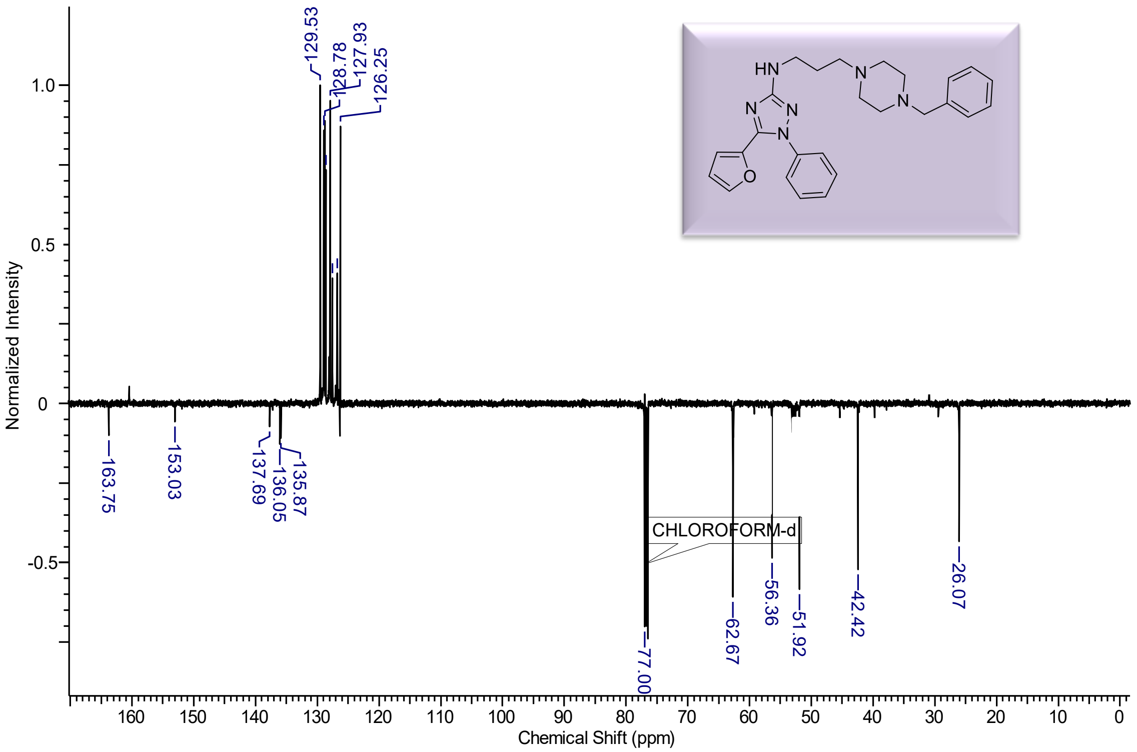

Figure S68: ^{1}H NMR (500 MHz) and ^{13}C NMR (125 MHz) spectra of compound **11q** in $CDCl_3$.

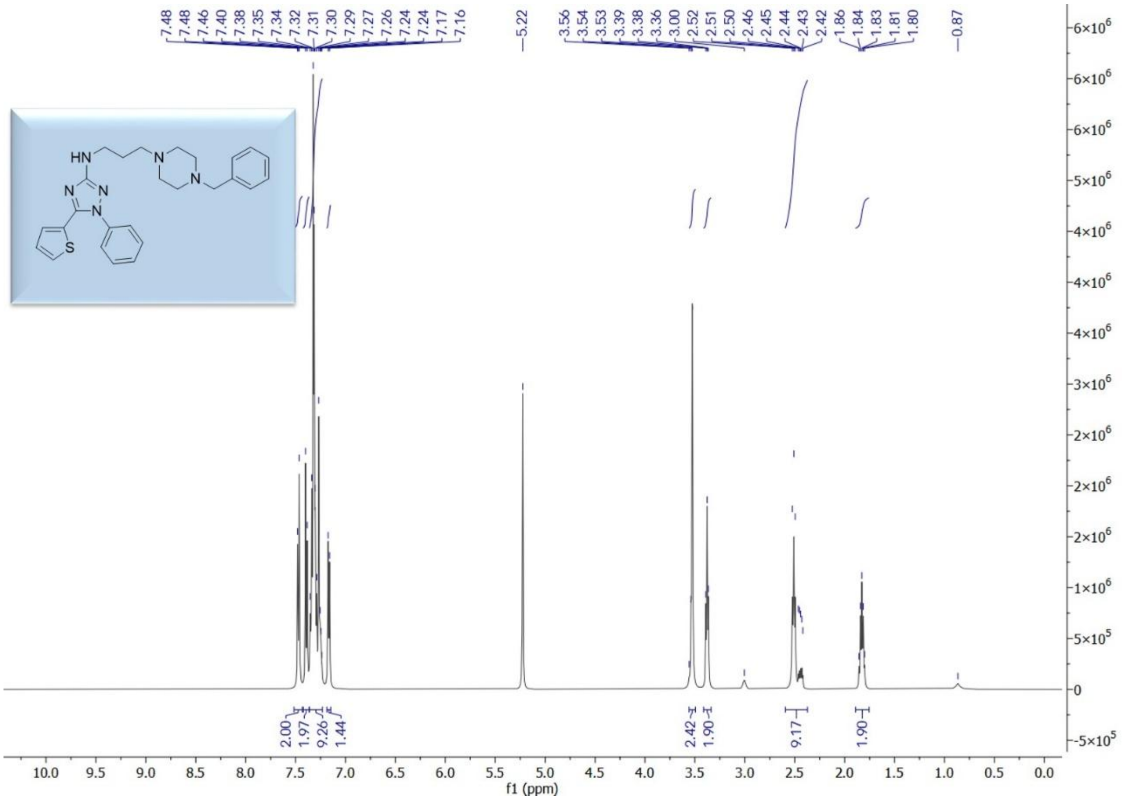

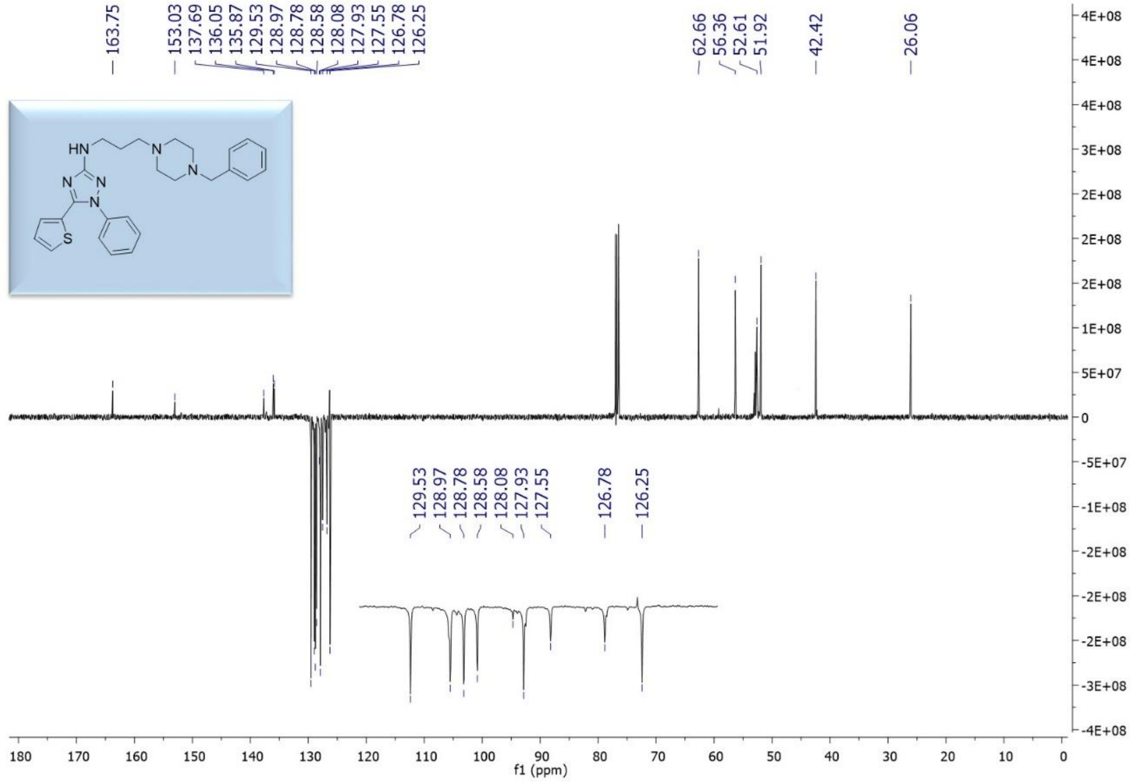

Figure S69: ^{1}H NMR (500 MHz) and ^{13}C NMR (125 MHz) spectra of compound **11r** in $CDCl_3$.

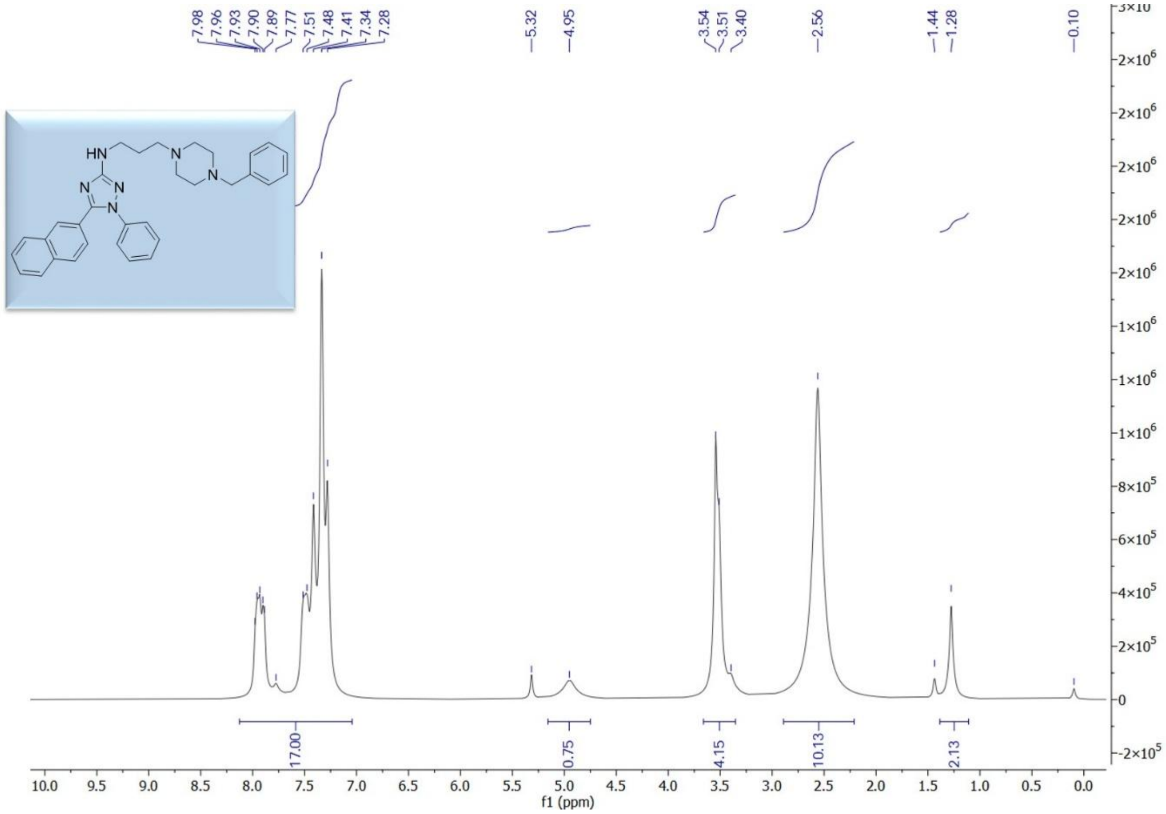

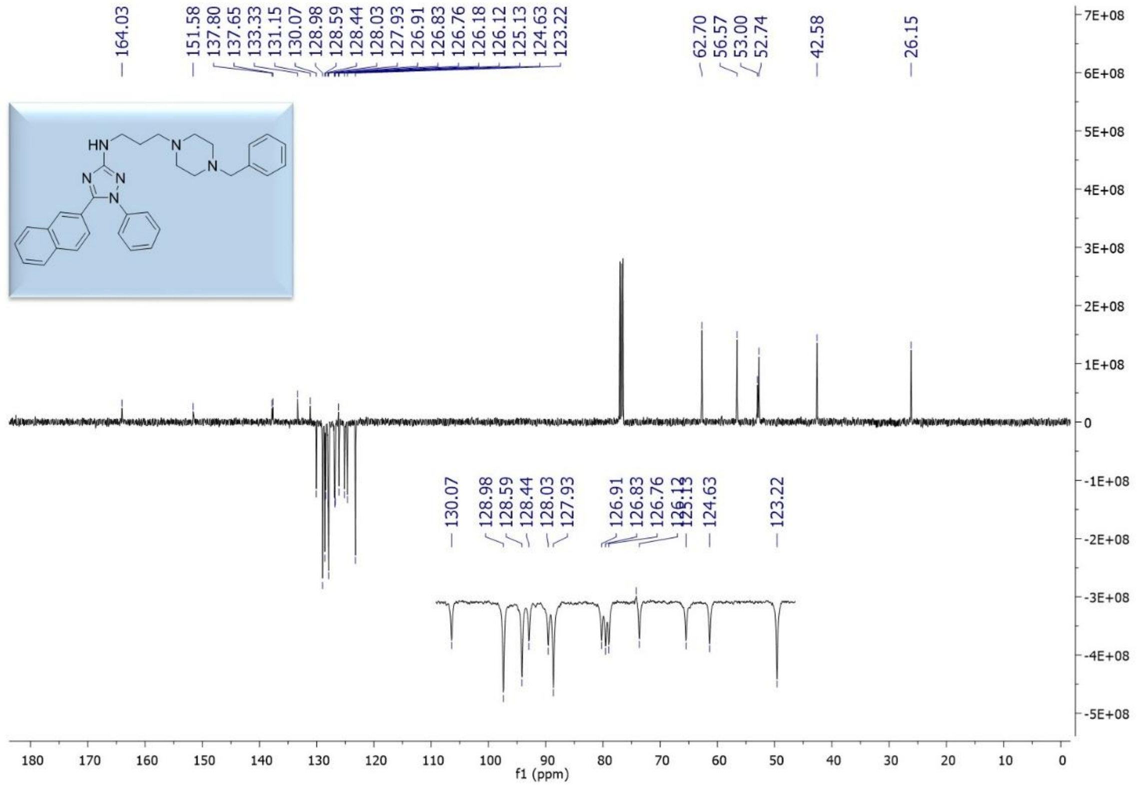

Figure S70: ^{1}H NMR (500 MHz) and ^{13}C NMR (125 MHz) spectra of compound **11s** in $CDCl_3$.

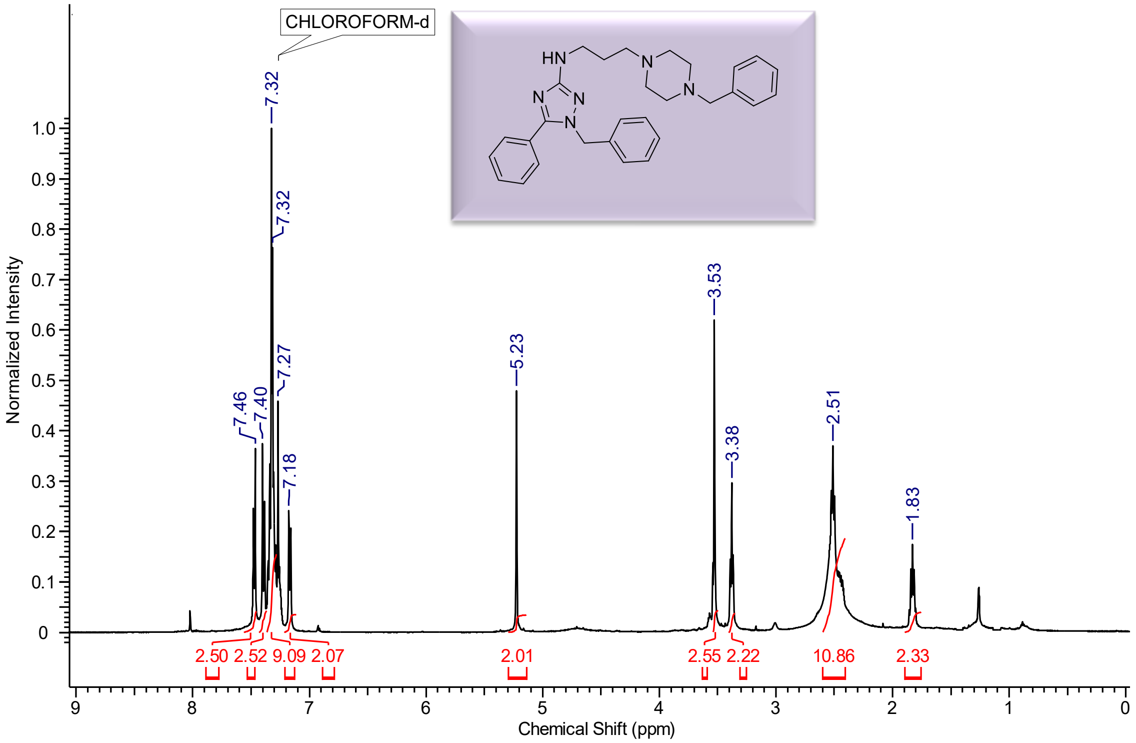

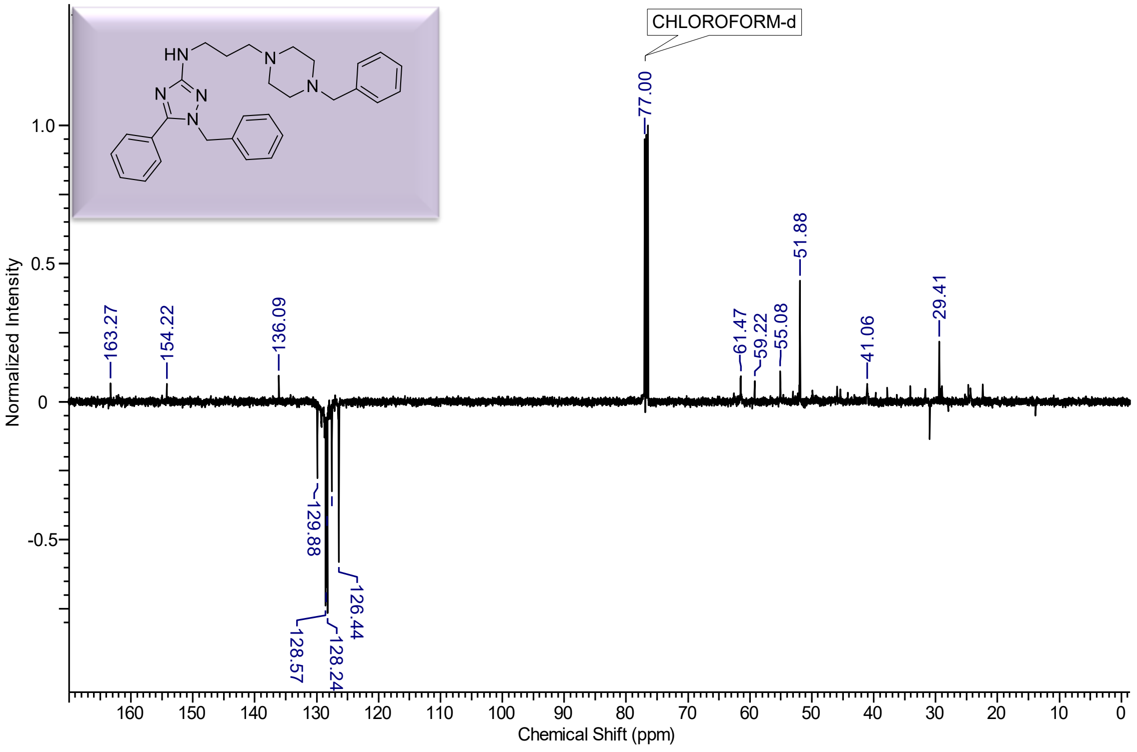

Figure S71: ^{1}H NMR (500 MHz) and ^{13}C NMR (125 MHz) spectra of compound **11t** in $CDCl_3$.

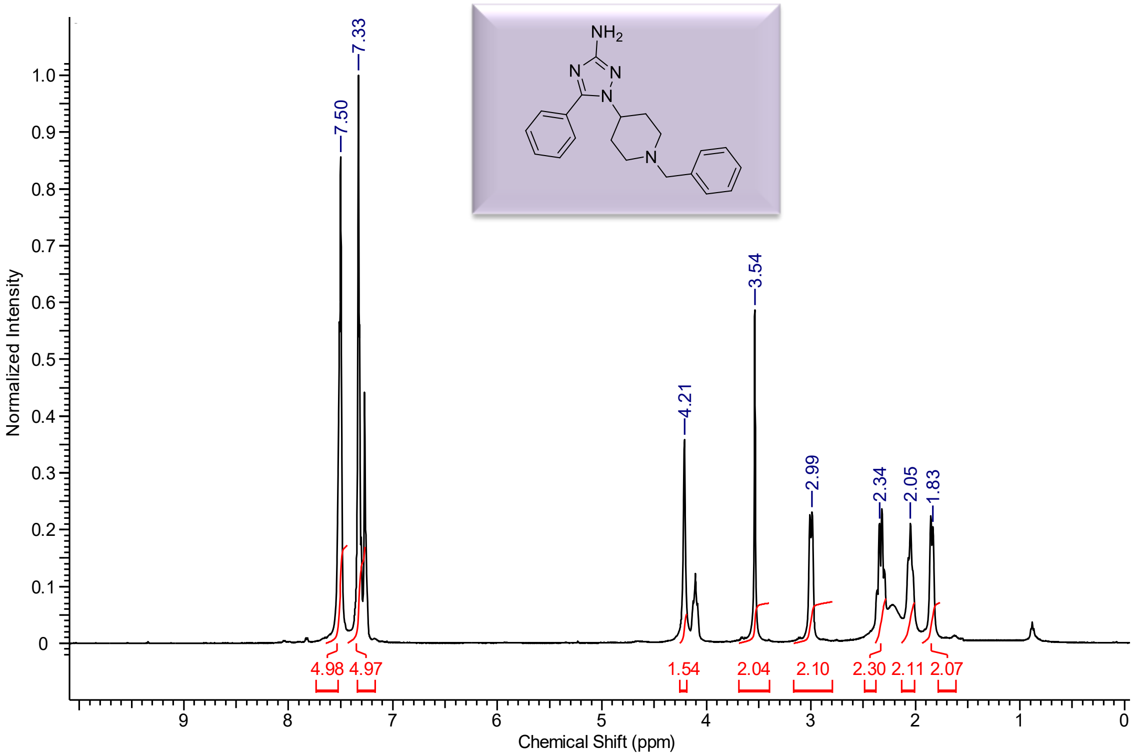

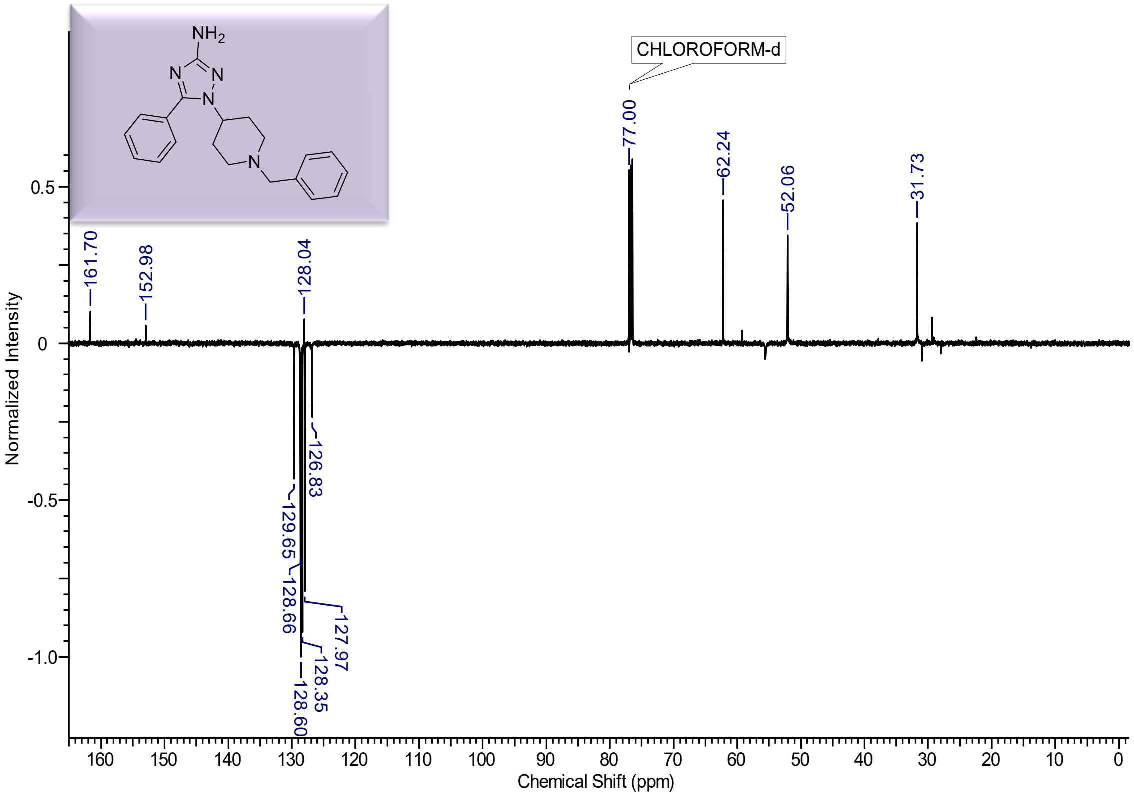

Figure S72: ^{1}H NMR (500 MHz) and ^{13}C NMR (125 MHz) spectra of compound **21** in $CDCl_3$.

SHIMADZU LabSolutions **Analysis Report**

<Sample Information>

Sample Name	: 2-Me-Ph		
Data Filename	: 2-Me-Ph.lcd		
Method Filename	: Daiana MeOH-Acido 20-80%.lcm		
Vial #	: 1-1	Sample Type	: Unknown
Injection Volume	: 10 uL		
Date Acquired	: 29/08/2025 11:01:02	Acquired by	: HPLC
Date Processed	: 29/08/2025 12:04:21	Processed by	: HPLC

<Chromatogram>

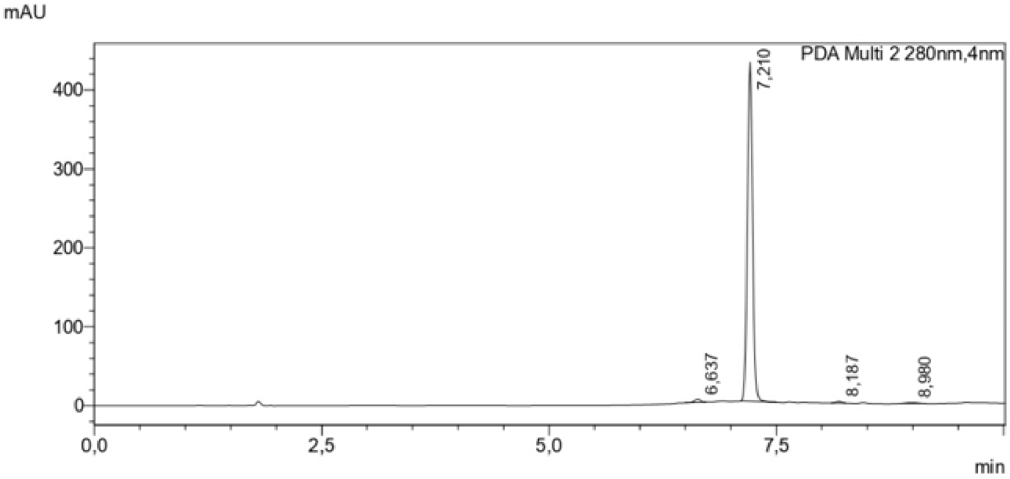

<Peak Table>

PDA Ch2 280nm

Peak#	Ret. Time	Area	Height	Area%	Name
1	6,637	19287	4048	1,076	
2	7,210	1754942	428721	97,866	
3	8,187	8843	2058	0,493	
4	8,980	10141	1036	0,566	
Total		1793212	435864	100,000	

UV Spectrum

Peak# : 2

Retention Time : 7,210 min

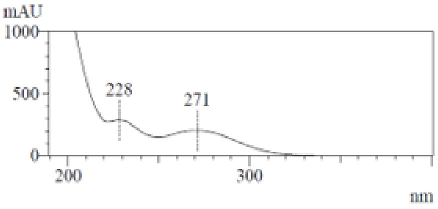

Figure S73: HPLC chromatogram of **11a**.

SHIMADZU LabSolutions **Analysis Report**

<Sample Information>

Sample Name	: 2-OH-Ph		
Data Filename	: 2-OH.Ph.lcd		
Method Filename	: Daiana MeOH-Acido 20-80%.lcm		
Vial #	: 1-1	Sample Type	: Unknown
Injection Volume	: 10 uL		
Date Acquired	: 30/08/2025 13:42:21	Acquired by	: HPLC
Date Processed	: 30/08/2025 14:07:35	Processed by	: HPLC

<Chromatogram>

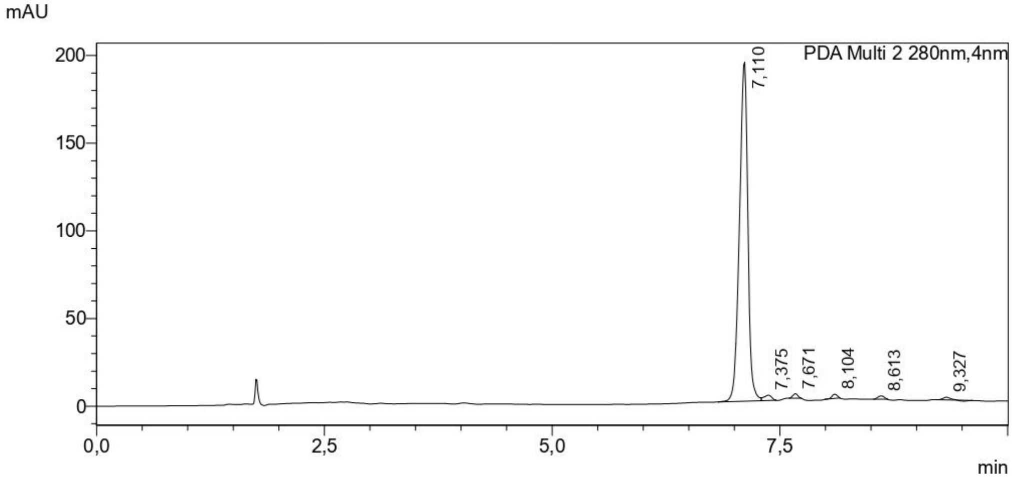

<Peak Table>

PDA Ch2 280nm

Peak#	Ret. Time	Area	Height	Area%	Name
1	7,110	1238993	193257	95,855	
2	7,375	17948	3059	1,389	
3	7,671	9779	2571	0,757	
4	8,104	9773	2379	0,756	
5	8,613	9178	1885	0,710	
6	9,327	6894	1529	0,533	
Total		1292566	204682	100,000	

UV Spectrum

Peak# : 1

Retention Time : 7,110 min

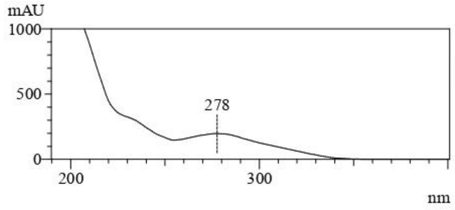

Figure S74: HPLC chromatogram of **11b**.

SHIMADZU LabSolutions

Analysis Report

<Sample Information>

Sample Name	: 4-OH-Ph		
Data Filename	: 4-OH-Ph.lcd		
Method Filename	: Daiana MeOH-Acido 20-80%.lcm		
Vial #	: 1-1	Sample Type	: Unknown
Injection Volume	: 10 uL		
Date Acquired	: 29/08/2025 12:15:50	Acquired by	: HPLC
Date Processed	: 29/08/2025 12:25:53	Processed by	: HPLC

<Chromatogram>

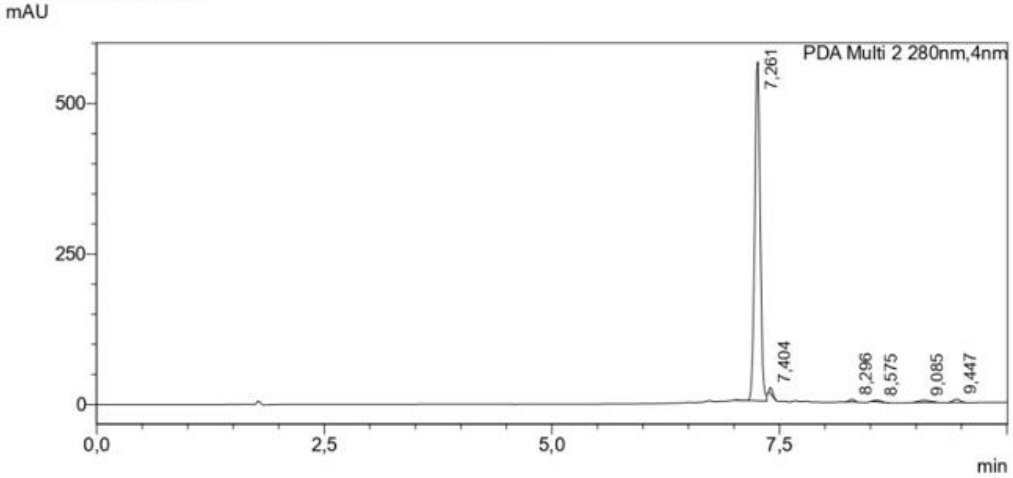

<Peak Table>

PDA Ch2 280nm

Peak#	Ret. Time	Area	Height	Area%	Name
1	7,261	2319184	562592	95,823	
2	7,404	21086	10650	0,871	
3	8,296	15725	4325	0,650	
4	8,575	11622	2526	0,480	
5	9,085	25143	3092	1,039	
6	9,447	27531	5516	1,138	
Total		2420291	588701	100,000	

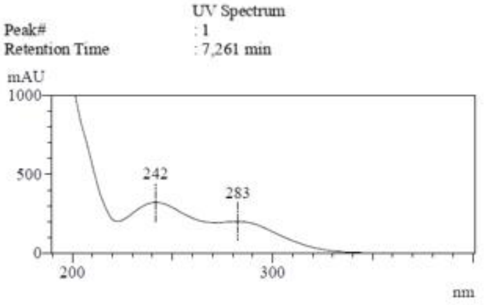

Figure S75: HPLC chromatogram of **11c**.

SHIMADZU LabSolutions

Analysis Report

<Sample Information>

Sample Name	: 2-MeO-Ph		
Data Filename	: 2-MeO-Ph.lcd		
Method Filename	: Daiana MeOH-Acido 20-80%.lcm		
Vial #	: 1-1	Sample Type	: Unknown
Injection Volume	: 10 uL		
Date Acquired	: 29/08/2025 12:30:44	Acquired by	: HPLC
Date Processed	: 29/08/2025 12:40:46	Processed by	: HPLC

<Chromatogram>

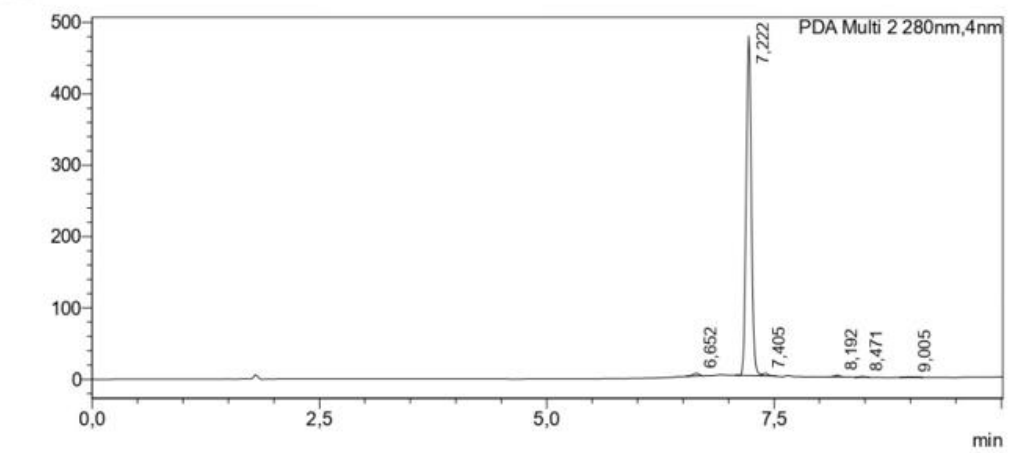

<Peak Table>

PDA Ch2 280nm

Peak#	Ret. Time	Area	Height	Name	Area%
1	6,652	17286	3761		0,890
2	7,222	1889346	475469		97,222
3	7,405	16084	3839		0,828
4	8,192	9226	2159		0,475
5	8,471	4920	1111		0,253
6	9,005	6475	686		0,333
Total		1943337	487026		100,000

UV Spectrum

Peak# : 2

Retention Time : 7,222 min

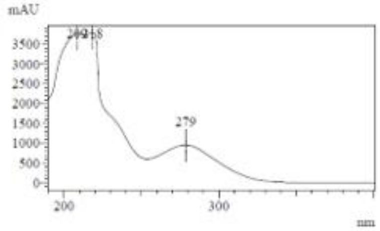

Figure S76: HPLC chromatogram of **11d**.

SHIMADZU LabSolutions **Analysis Report**

<Sample Information>

Sample Name	: 4-MeO-Ph.		
Data Filename	: 4-MeO-Ph..lcd		
Method Filename	: Daiana MeOH-Acido 20-80%.lcm		
Vial #	: 1-1	Sample Type	: Unknown
Injection Volume	: 10 uL		
Date Acquired	: 30/08/2025 14:25:45	Acquired by	: HPLC
Date Processed	: 30/08/2025 14:35:48	Processed by	: HPLC

<Chromatogram>

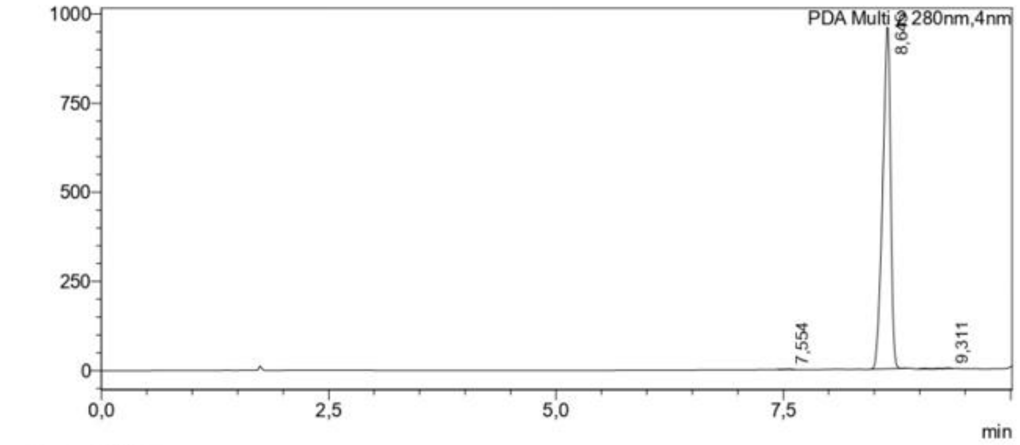

<Peak Table>

PDA Ch2 280nm

Peak#	Ret. Time	Area	Height	Area%	Name
1	7,554	7610	1734	0,130	
2	8,645	5852983	957382	99,740	
3	9,311	7636	1443	0,130	
Total		5868228	960559	100,000	

UV Spectrum

Peak# : 2

Retention Time : 8,645 min

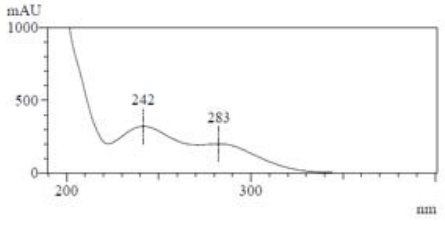

Figure S77: HPLC chromatogram of **11e**.

SHIMADZU LabSolutions

Analysis Report

<Sample Information>

Sample Name	: TriMeO-Ph		
Data Filename	: TriMeO-Ph.lcd		
Method Filename	: Daiana MeOH-Acido 20-80%.lcm		
Vial #	: 1-1	Sample Type	: Unknown
Injection Volume	: 10 uL		
Date Acquired	: 29/08/2025 14:19:56	Acquired by	: HPLC
Date Processed	: 29/08/2025 14:29:59	Processed by	: HPLC

<Chromatogram>

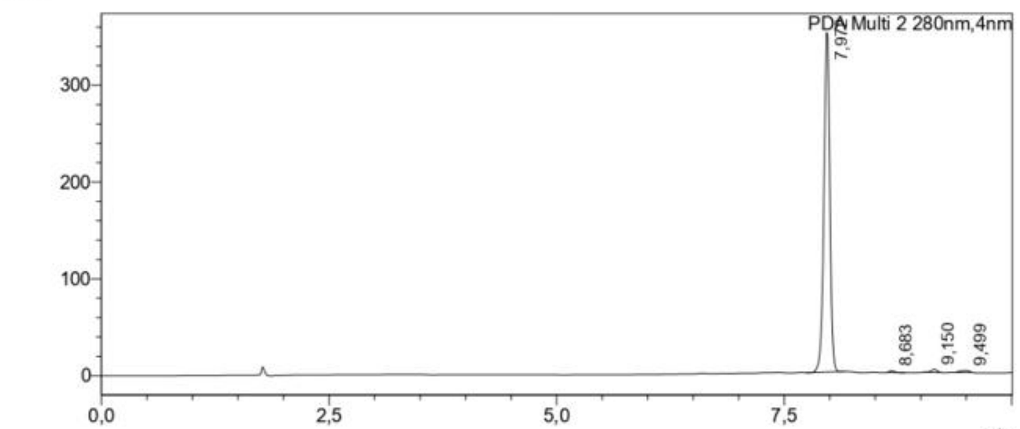

<Peak Table>

PDA Ch2 280nm

Peak#	Ret. Time	Area	Height	Area%	Name
1	7,972	1587513	350321	97,798	
2	8,683	7319	1820	0,451	
3	9,150	13869	3194	0,854	
4	9,499	14561	2015	0,897	
Total		1623262	357350	100,000	

UV Spectrum

Peak# : 1

Retention Time : 7,972 min

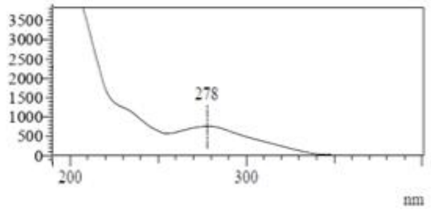

Figure S78: HPLC chromatogram of **11f**.

Analysis Report

SHIMADZU LabSolutions

<Sample Information>

Sample Name	: 4F		
Data Filename	: 4F.lcd		
Method Filename	: NAH-Finais MeOH-HAc 20-80%.lcm		
Vial #	: 1-1	Sample Type	: Unknown
Injection Volume	: 10 uL		
Date Acquired	: 28/08/2025 13:32:31	Acquired by	: HPLC
Date Processed	: 29/08/2025 13:01:18	Processed by	: HPLC

<Chromatogram>

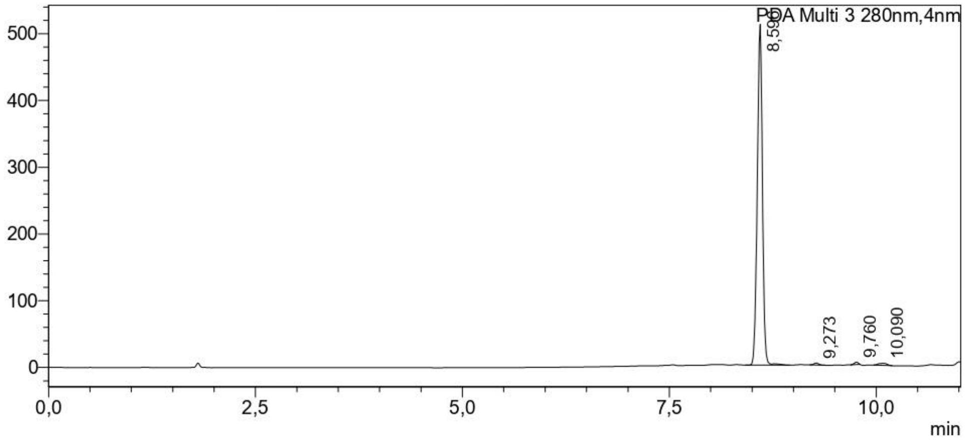

<Peak Table>

PDA Ch3 280nm

Peak#	Ret. Time	Area	Height	Area%	Name
1	8,596	2199979	510337	97,937	
2	9,273	12475	3062	0,555	
3	9,760	11757	3535	0,523	
4	10,090	22120	3157	0,985	
Total		2246331	520090	100,000	

UV Spectrum

Peak# : 1

Retention Time : 8,596 min

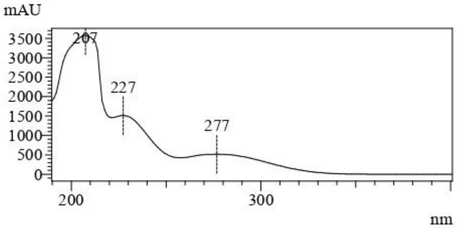

Figure S79: HPLC chromatogram of **11g**.

Analysis Report

SHIMADZU LabSolutions

<Sample Information>

Sample Name	: 2-Cl-Ph		
Data Filename	: 2-Cl-Ph.lcd		
Method Filename	: Daiana MeOH-Acido 20-80%.lcm		
Vial #	: 1-1	Sample Type	: Unknown
Injection Volume	: 10 uL		
Date Acquired	: 29/08/2025 13:54:18	Acquired by	: HPLC
Date Processed	: 29/08/2025 14:04:20	Processed by	: HPLC

<Chromatogram>

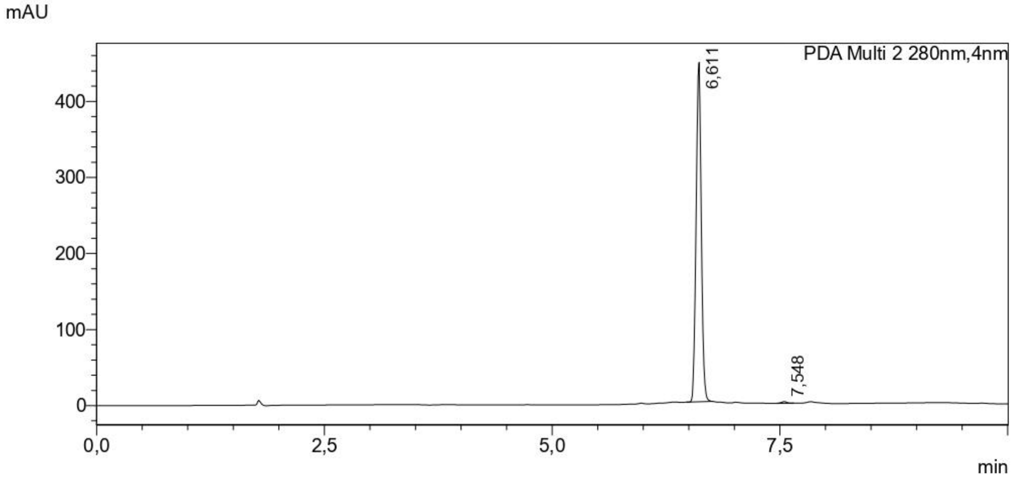

<Peak Table>

PDA Ch2 280nm

Peak#	Ret. Time	Area	Height	Area%	Name
1	6,611	1767766	446090	99,523	
2	7,548	8464	2034	0,477	
Total		1776230	448124	100,000	

UV Spectrum

Peak# : 1

Retention Time : 6,611 min

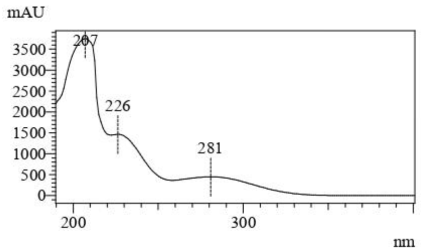

Figure S80: HPLC chromatogram of **11h**.

Analysis Report

SHIMADZU LabSolutions

<Sample Information>

Sample Name	: 3-Cl-Ph		
Data Filename	: 3-Cl-Ph.lcd		
Method Filename	: Daiana MeOH-Acido 20-80%.lcm		
Vial #	: 1-1	Sample Type	: Unknown
Injection Volume	: 10 uL		
Date Acquired	: 29/08/2025 13:41:40	Acquired by	: HPLC
Date Processed	: 29/08/2025 13:51:42	Processed by	: HPLC

<Chromatogram>

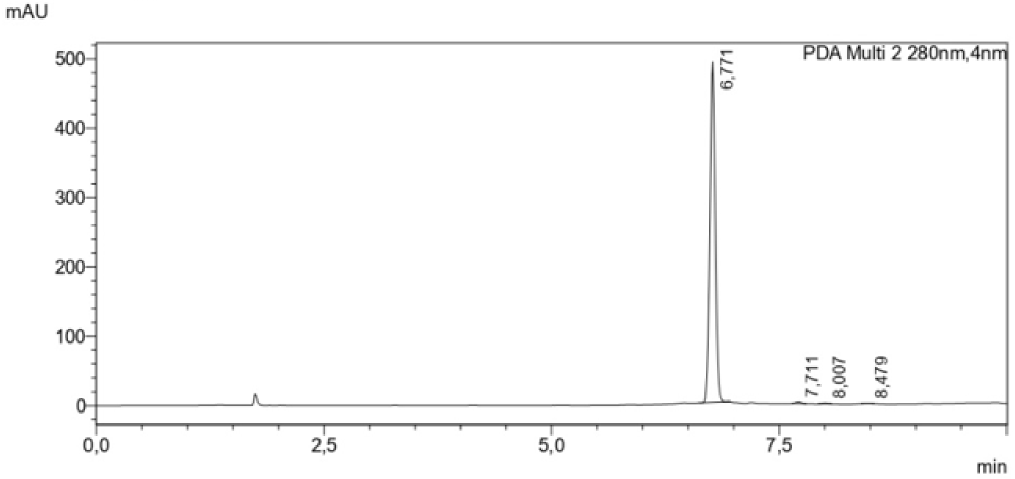

<Peak Table>

PDA Ch2 280nm

Peak#	Ret. Time	Area	Height	Area%	Name
1	6,771	1995216	490464	99,187	
2	7,711	8589	2191	0,427	
3	8,007	5036	1148	0,250	
4	8,479	2725	522	0,135	
Total		2011565	494325	100,000	

UV Spectrum

Peak# : 1
Retention Time : 6,771 min

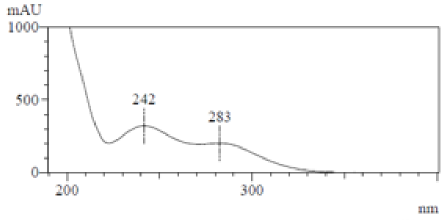

Figure S81: HPLC chromatogram of **11i**.

SHIMADZU LabSolutions

Analysis Report

<Sample Information>

Sample Name	: 4-Cl-Ph		
Data Filename	: 4-Cl.Ph.lcd		
Method Filename	: Daiana MeOH-Acido 20-80%.lcm		
Vial #	: 1-1	Sample Type	: Unknown
Injection Volume	: 10 uL		
Date Acquired	: 30/08/2025 13:57:00	Acquired by	: HPLC
Date Processed	: 30/08/2025 14:07:03	Processed by	: HPLC

<Chromatogram>

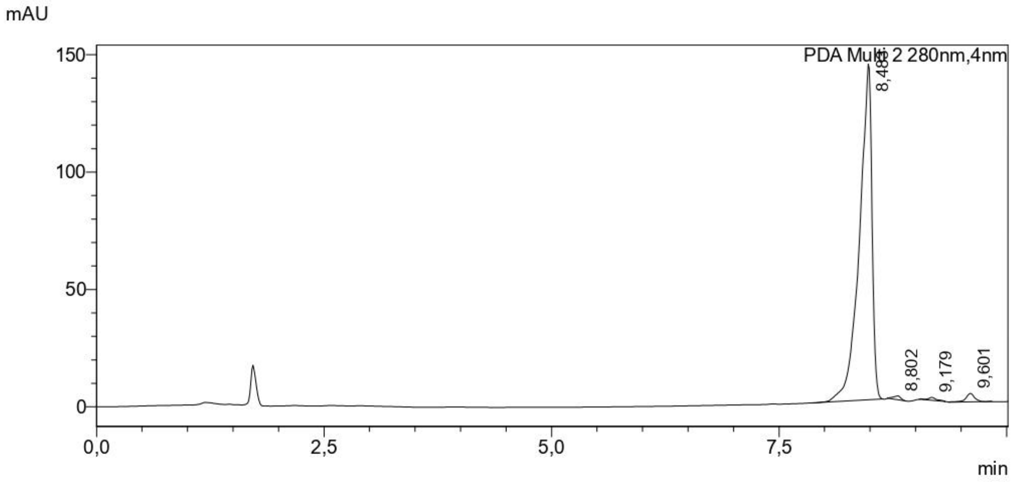

<Peak Table>

PDA Ch2 280nm

Peak#	Ret. Time	Area	Height	Area%	Name
1	8,483	1363587	142898	97,083	
2	8,802	9343	1659	0,665	
3	9,179	7403	1189	0,527	
4	9,601	24226	3550	1,725	
Total		1404559	149296	100,000	

UV Spectrum

Peak# : 1

Retention Time : 8,483 min

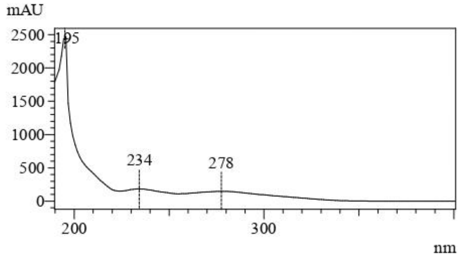

Figure S82: HPLC chromatogram of **11j**.

SHIMADZU LabSolutions Analysis Report

<Sample Information>

Sample Name	: 3-Br		
Data Filename	: 3-Br.lcd		
Method Filename	: Daiana MeOH-Acido 20-80%.lcm		
Vial #	: 1-1	Sample Type	: Unknown
Injection Volume	: 10 uL		
Date Acquired	: 28/08/2025 15:48:06	Acquired by	: HPLC
Date Processed	: 28/08/2025 16:03:26	Processed by	: HPLC

<Chromatogram>

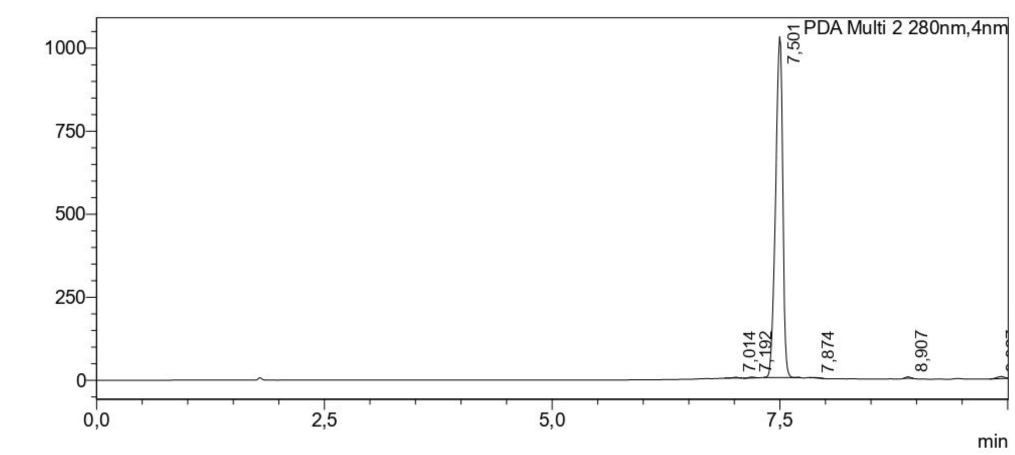

<Peak Table>

PDA Ch2 280nm

Peak#	Ret. Time	Area	Height	Area%	Name
1	7,014	10008	2372	0,178	
2	7,192	11289	2516	0,200	
3	7,501	5555238	1025657	98,615	
4	7,874	7003	1418	0,124	
5	8,907	14721	4459	0,261	
6	9,927	35004	6117	0,621	
Total		5633263	1042540	100,000	

UV Spectrum

Peak# : 3

Retention Time : 7,501 min

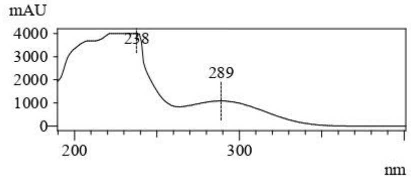

Figure S83: HPLC chromatogram of **11k**.

Analysis Report

SHIMADZU LabSolutions

<Sample Information>

Sample Name	: 4-Br-Ph		
Data Filename	: 4-Br-Ph.lcd		
Method Filename	: Daiana MeOH-Acido 20-80%.lcm		
Vial #	: 1-1	Sample Type	: Unknown
Injection Volume	: 10 uL		
Date Acquired	: 29/08/2025 13:26:59	Acquired by	: HPLC
Date Processed	: 29/08/2025 13:37:02	Processed by	: HPLC

<Chromatogram>

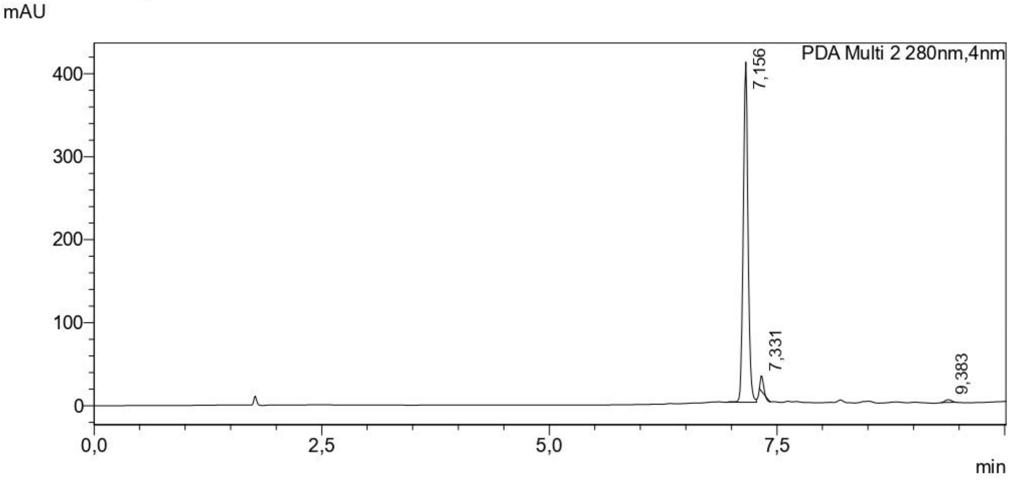

<Peak Table>

PDA Ch2 280nm

Peak#	Ret. Time	Area	Height	Area%	Name
1	7,156	1491406	409806	96,776	
2	7,331	33417	18759	2,168	
3	9,383	16272	3315	1,056	
Total		1541094	431881	100,000	

UV Spectrum

Peak# : 1

Retention Time : 7,156 min

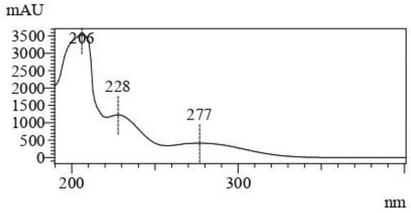

Figure S84: HPLC chromatogram of **11l**.

SHIMADZU LabSolutions **Analysis Report**

<Sample Information>

Sample Name	: 3-NO2		
Data Filename	: 3-NO2.lcd		
Method Filename	: Daiana MeOH-Acido 20-80%.lcm		
Vial #	: 1-1	Sample Type	: Unknown
Injection Volume	: 10 uL		
Date Acquired	: 28/08/2025 16:02:07	Acquired by	: HPLC
Date Processed	: 29/08/2025 13:09:52	Processed by	: HPLC

<Chromatogram>

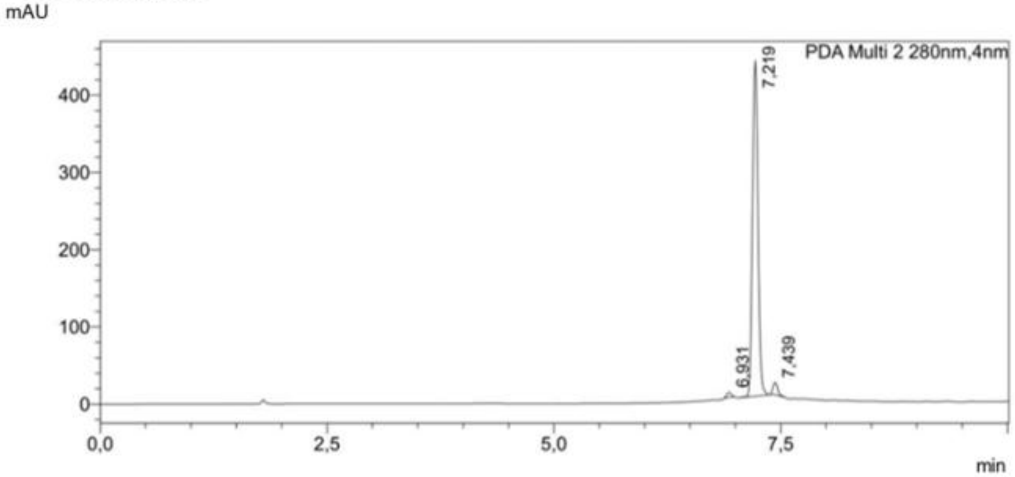

<Peak Table>

PDA Ch2 280nm

Peak#	Ret. Time	Area	Height	Area%	Name
1	6,931	31611	7541	1,644	
2	7,219	1835000	434510	95,406	
3	7,439	56752	16111	2,951	
Total		1923363	458161	100,000	

UV Spectrum

Peak# : 2

Retention Time : 7,219 min

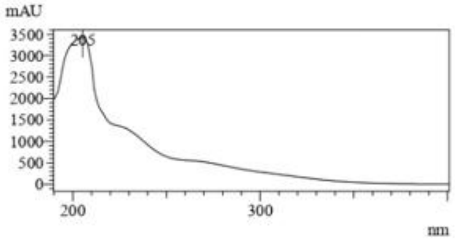

Figure S85: HPLC chromatogram of **11m**.

SHIMADZU LabSolutions Analysis Report

<Sample Information>

Sample Name	: 4-NO2-Ph		
Data Filename	: 4-NO2-Ph.lcd		
Method Filename	: Daiana MeOH-Acido 20-80%.lcm		
Vial #	: 1-1	Sample Type	: Unknown
Injection Volume	: 10 uL		
Date Acquired	: 29/08/2025 13:13:26	Acquired by	: HPLC
Date Processed	: 29/08/2025 13:23:28	Processed by	: HPLC

<Chromatogram>

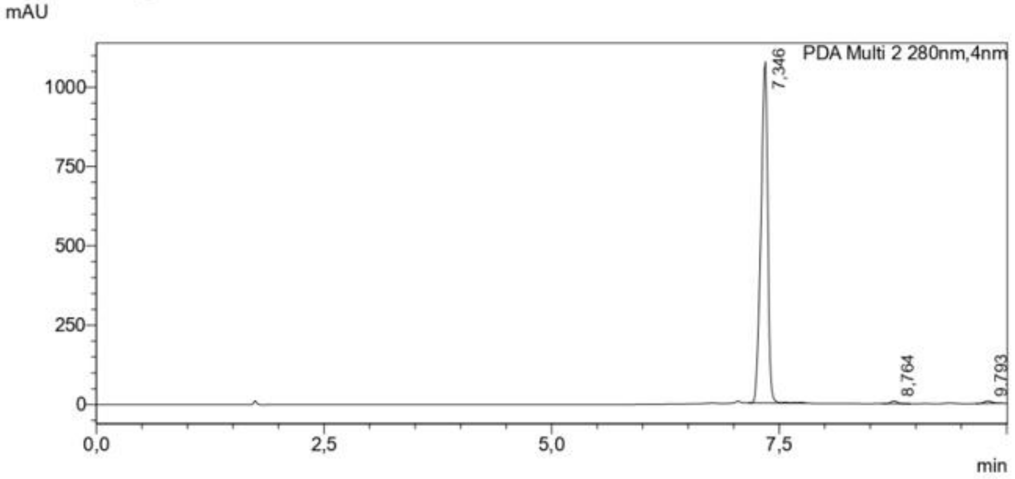

<Peak Table>

PDA Ch2 280nm

Peak#	Ret. Time	Area	Height	Area%	Name
1	7,346	5597808	1073896	98,336	
2	8,764	45848	8139	0,805	
3	9,793	48850	7372	0,858	
Total		5692506	1089407	100,000	

UV Spectrum

Peak# : 1

Retention Time : 7,346 min

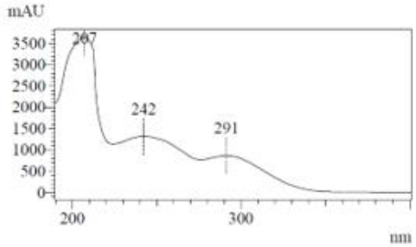

Figure S86: HPLC chromatogram of **11n**.

SHIMADZU LabSolutions

Analysis Report

<Sample Information>

Sample Name	: (N)4-NO2-Ph		
Data Filename	: (N)4-NO2-Ph.lcd		
Method Filename	: Daiana MeOH-Acido 20-80%.lcm		
Vial #	: 1-1	Sample Type	: Unknown
Injection Volume	: 10 uL		
Date Acquired	: 29/08/2025 12:57:48	Acquired by	: HPLC
Date Processed	: 29/08/2025 13:23:56	Processed by	: HPLC

<Chromatogram>

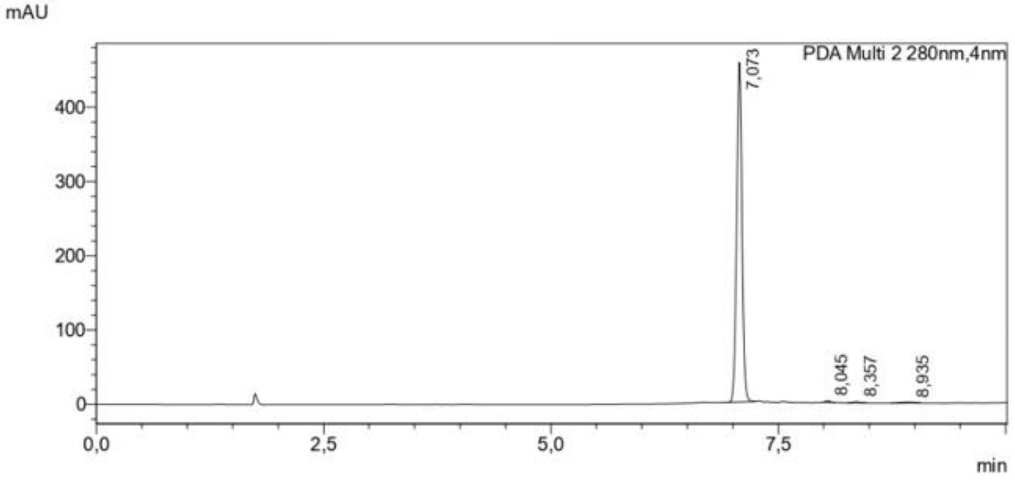

<Peak Table>

PDA Ch2 280nm

Peak#	Ret. Time	Area	Height	Area%	Name
1	7,073	1843348	456834	98,647	
2	8,045	7910	2031	0,423	
3	8,357	6463	1300	0,346	
4	8,935	10911	1068	0,584	
Total		1868632	461233	100,000	

UV Spectrum

Peak# : 1

Retention Time : 7,073 min

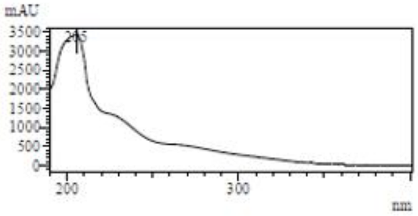

Figure S87: HPLC chromatogram of **11o**.

SHIMADZU LabSolutions Analysis Report

<Sample Information>

Sample Name	: 2-Py.1		
Data Filename	: 2-Py.1.lcd		
Method Filename	: Daiana MeOH-Acido 20-80%.lcm		
Vial #	: 1-1	Sample Type	: Unknown
Injection Volume	: 10 uL		
Date Acquired	: 29/08/2025 14:34:38	Acquired by	: HPLC
Date Processed	: 29/08/2025 14:44:41	Processed by	: HPLC

<Chromatogram>

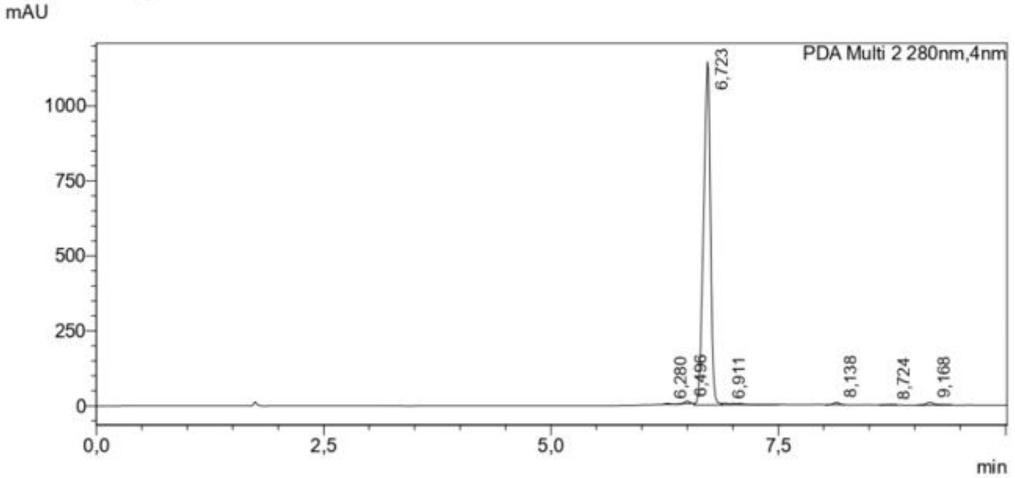

<Peak Table>

PDA Ch2 280nm

Peak#	Ret. Time	Area	Height	Area%	Name
1	6,280	11683	2934	0,189	
2	6,496	33452	8244	0,541	
3	6,723	5970045	1141944	96,549	
4	6,911	61280	5435	0,991	
5	8,138	33446	7314	0,541	
6	8,724	10866	1984	0,176	
7	9,168	62640	7902	1,013	
Total		6183413	1175759	100,000	

UV Spectrum

Peak# : 3

Retention Time : 6,723 min

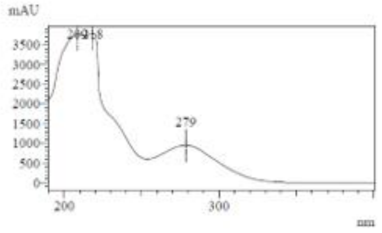

Figure S88: HPLC chromatogram of **11p**.

SHIMADZU LabSolutions Analysis Report

<Sample Information>

Sample Name	: Furan		
Data Filename	: Furan.lcd		
Method Filename	: Daiana MeOH-Acido 20-80%.lcm		
Vial #	: 1-1	Sample Type	: Unknown
Injection Volume	: 10 uL		
Date Acquired	: 30/08/2025 13:14:30	Acquired by	: HPLC
Date Processed	: 30/08/2025 13:24:32	Processed by	: HPLC

<Chromatogram>

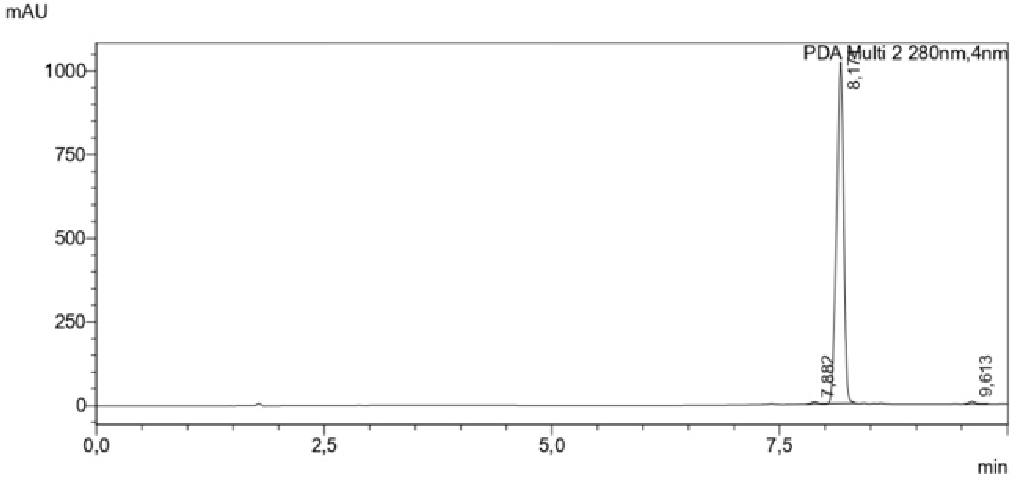

<Peak Table>

PDA Ch2 280nm

Peak#	Ret. Time	Area	Height	Area%	Name
1	7,882	17443	5277	0,316	
2	8,171	5471665	1019268	99,117	
3	9,613	31290	6796	0,567	
Total		5520397	1031341	100,000	

UV Spectrum

Peak# : 2

Retention Time : 8,171 min

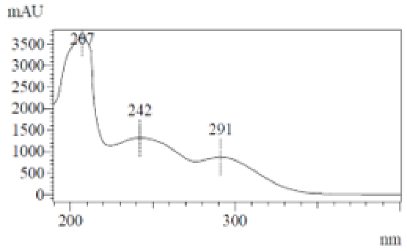

Figure S89: HPLC chromatogram of **11q**.

SHIMADZU LabSolutions

Analysis Report

<Sample Information>

Sample Name	: Thio.2		
Data Filename	: Thio.2.lcd		
Method Filename	: Daiana MeOH-Acido 20-80%.lcm		
Vial #	: 1-1	Sample Type	: Unknown
Injection Volume	: 10 uL		
Date Acquired	: 30/08/2025 13:02:17	Acquired by	: HPLC
Date Processed	: 30/08/2025 13:12:20	Processed by	: HPLC

<Chromatogram>

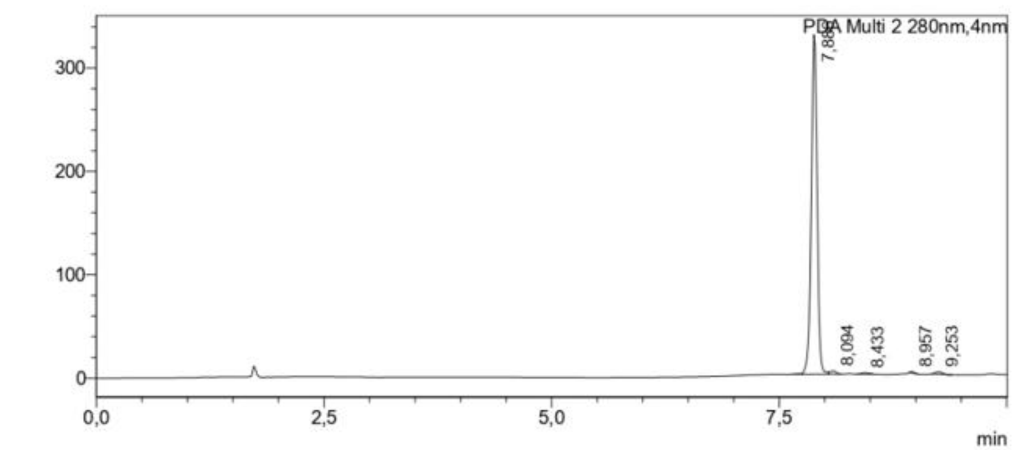

<Peak Table>

PDA Ch2 280nm

Peak#	Ret. Time	Area	Height	Area%	Name
1	7,886	1448566	328481	97,472	
2	8,094	14780	3308	0,995	
3	8,433	7163	1275	0,482	
4	8,957	3988	1416	0,268	
5	9,253	11637	2222	0,783	
Total		1486134	336703	100,000	

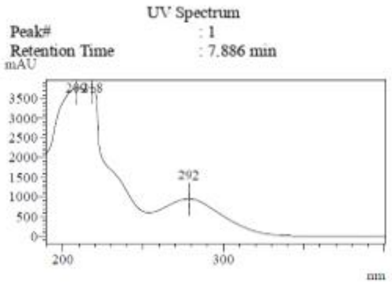

Figure S90: HPLC chromatogram of **11r**.

SHIMADZU LabSolutions Analysis Report

<Sample Information>

Sample Name	: Naphthyl		
Data Filename	: Naphthyl.lcd		
Method Filename	: Daiana MeOH-Acido 20-80%.lcm		
Vial #	: 1-1	Sample Type	: Unknown
Injection Volume	: 10 uL		
Date Acquired	: 30/08/2025 13:29:24	Acquired by	: HPLC
Date Processed	: 30/08/2025 13:52:50	Processed by	: HPLC

<Chromatogram>

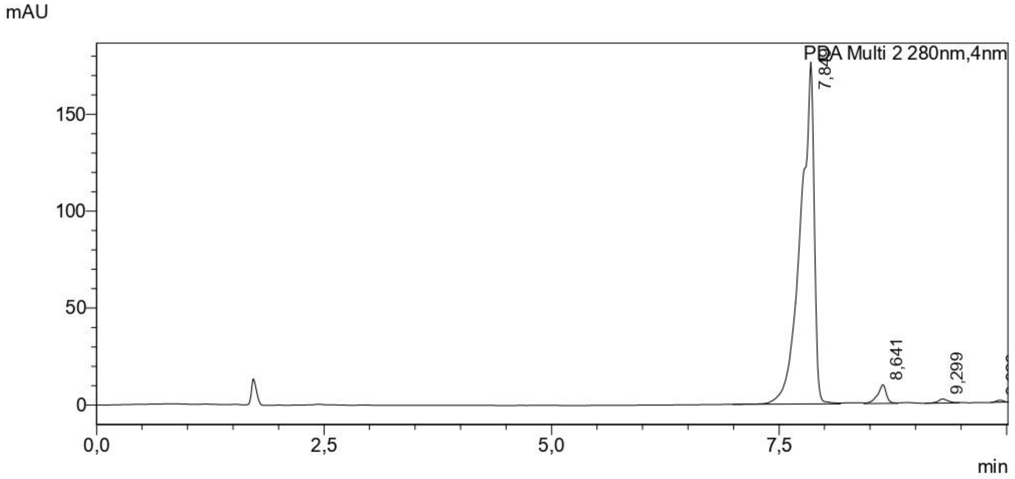

<Peak Table>

PDA Ch2 280nm

Peak#	Ret. Time	Area	Height	Area%	Name
1	7,849	1844113	176184	95,522	
2	8,641	64774	9724	3,355	
3	9,299	15877	2081	0,822	
4	9,926	5802	1075	0,301	
Total		1930566	189064	100,000	

UV Spectrum

Peak# : 1

Retention Time : 7,849 min

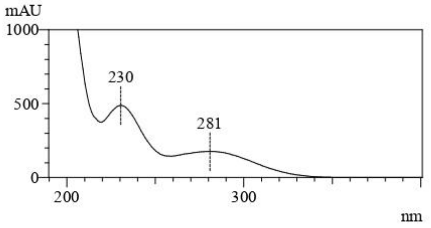

Figure S91: HPLC chromatogram of **11s**.

SHIMADZU LabSolutions

Analysis Report

<Sample Information>

Sample Name	: Benzyl		
Data Filename	: Benzyl.lcd		
Method Filename	: Daiana MeOH-Acido 20-80%.lcm		
Vial #	: 1-1	Sample Type	: Unknown
Injection Volume	: 10 uL		
Date Acquired	: 30/08/2025 14:50:41	Acquired by	: HPLC
Date Processed	: 30/08/2025 15:00:44	Processed by	: HPLC

<Chromatogram>

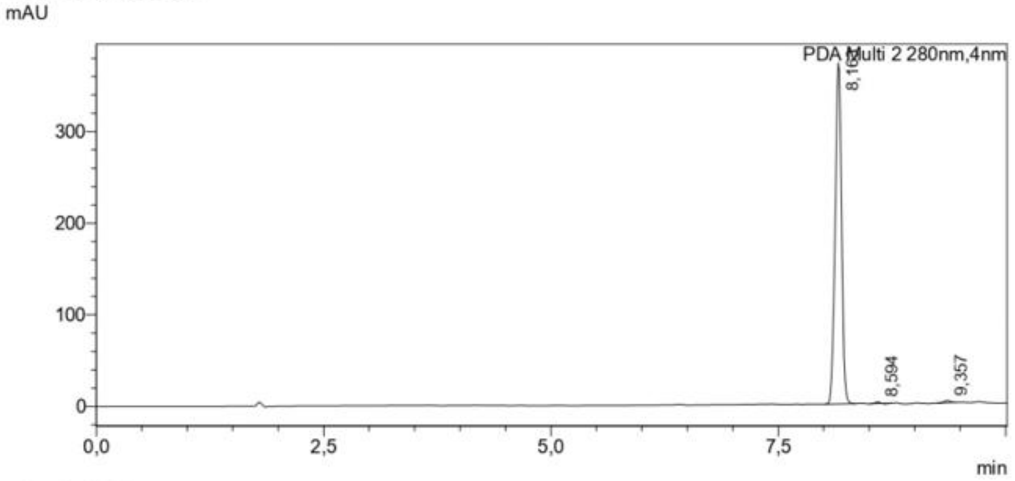

<Peak Table>

PDA Ch2 280nm

Peak#	Ret. Time	Area	Height	Area%	Name
1	8,163	1822976	371824	99,111	
2	8,594	4796	1745	0,261	
3	9,357	11547	2337	0,628	
Total		1839320	375906	100,000	

UV Spectrum

Peak# : 1

Retention Time : 8,163 min

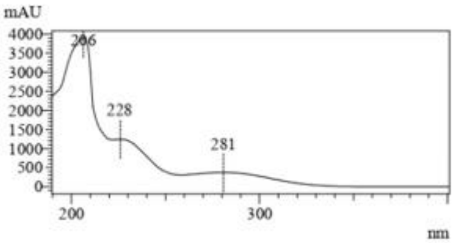

Figure S92: HPLC chromatogram of **11t**.

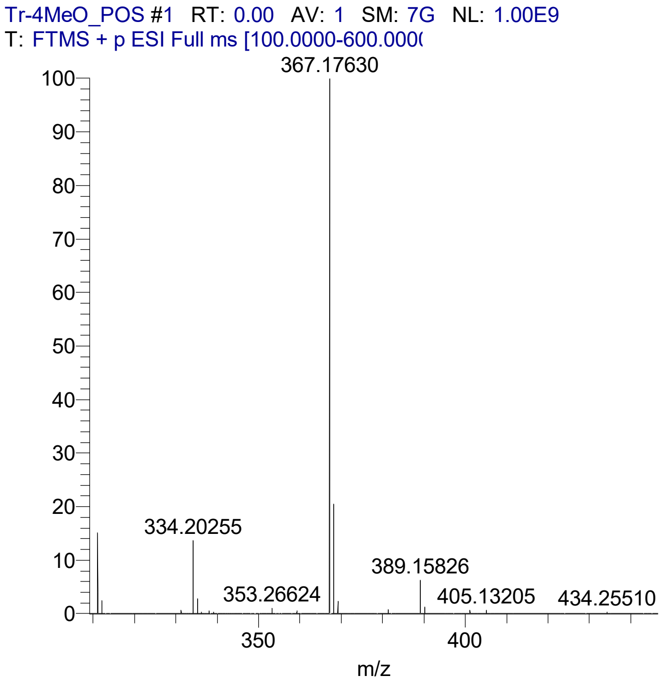

Figure S93: HRMS spectra of **16e**.

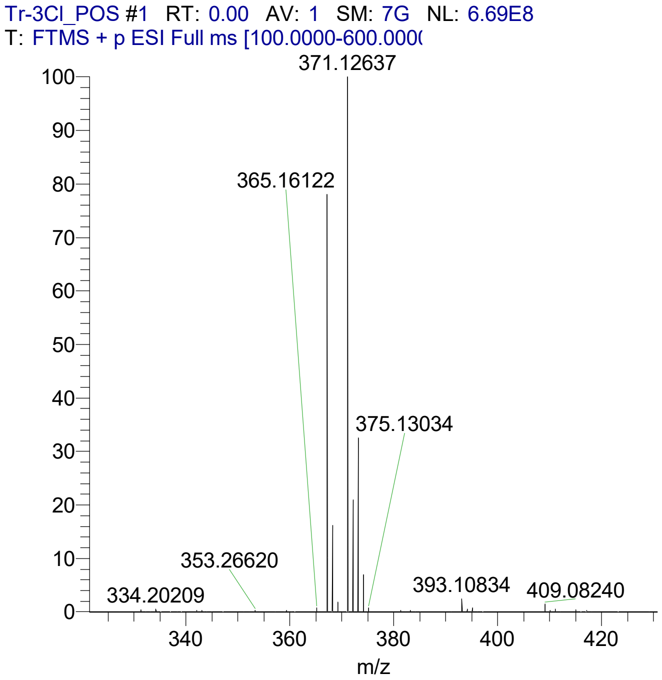

Figure S94: HRMS spectra of **16i**.

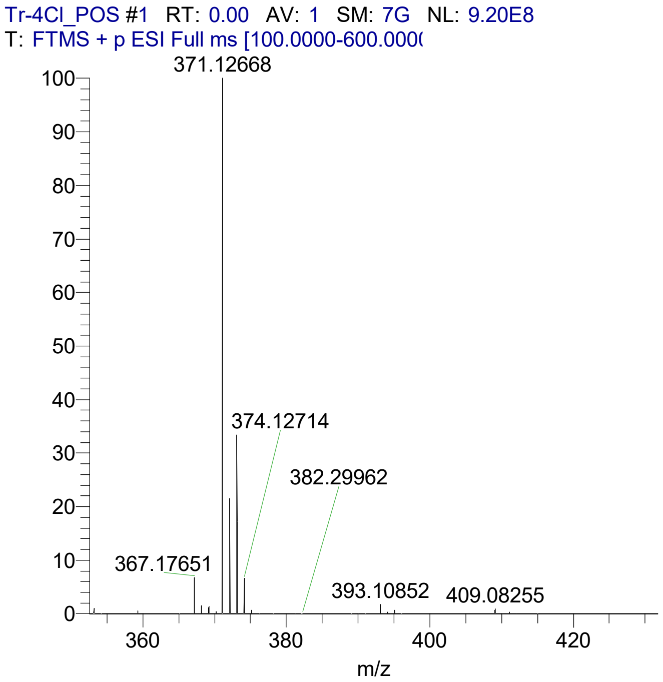

Figure S95: HRMS spectra of **16j**.

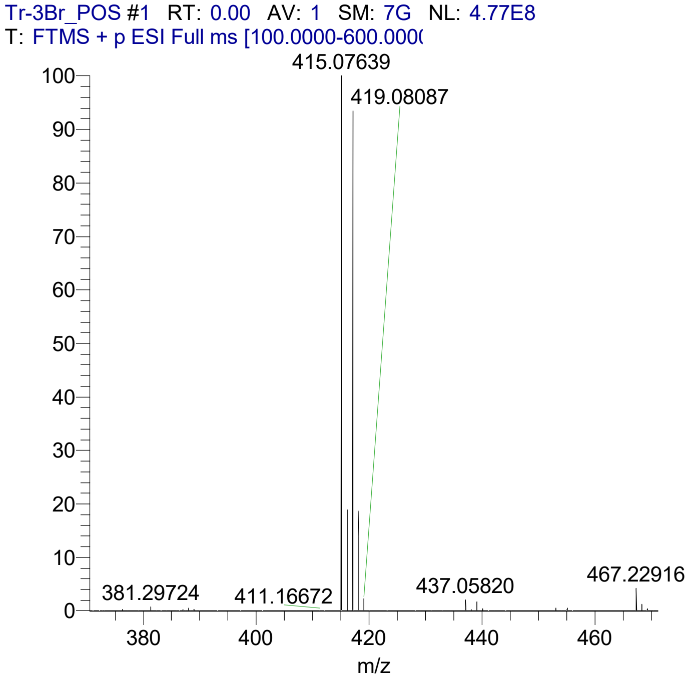

Figure S96: HRMS spectra of **16k**.

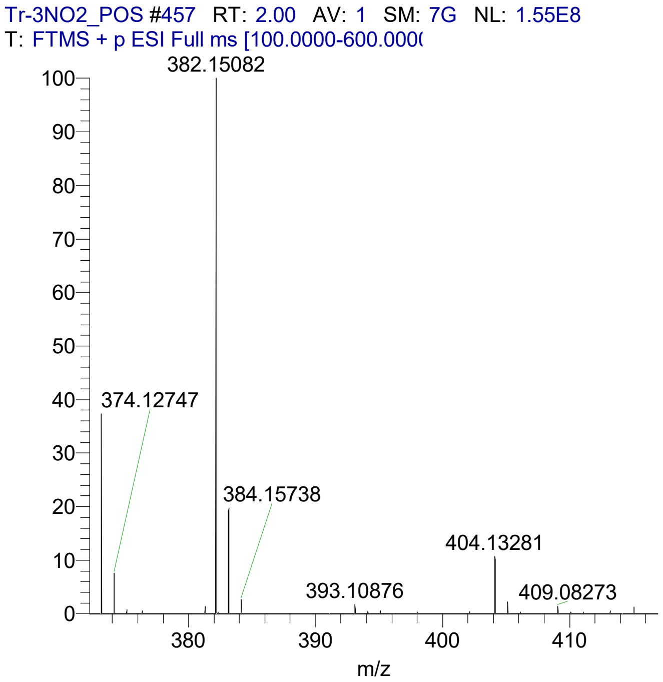

Figure S97: HRMS spectra of **16m**.

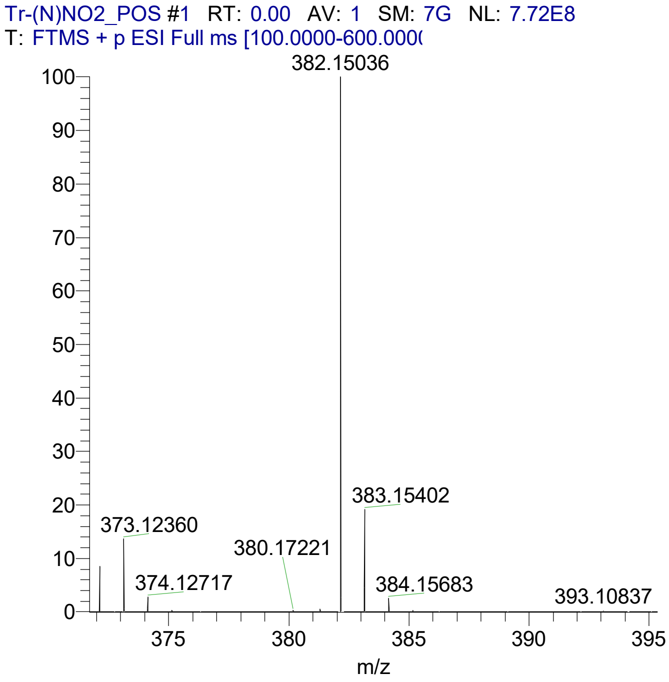

Figure S98: HRMS spectra of **16o**.

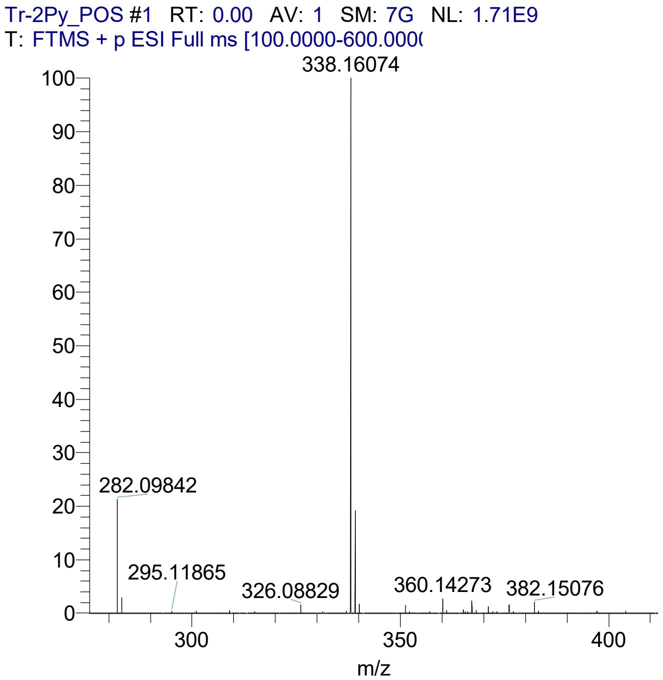

Figure S99: HRMS spectra of **16p**.

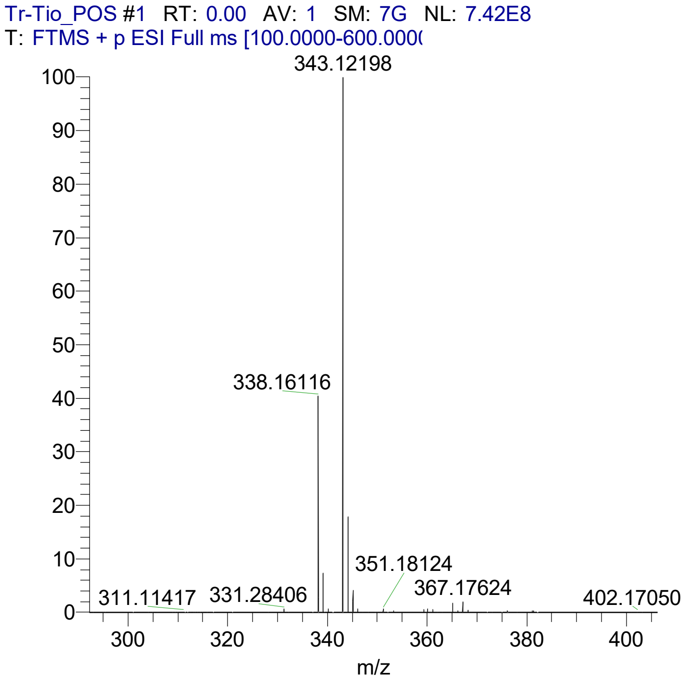

Figure S100: HRMS spectra of **16r**.

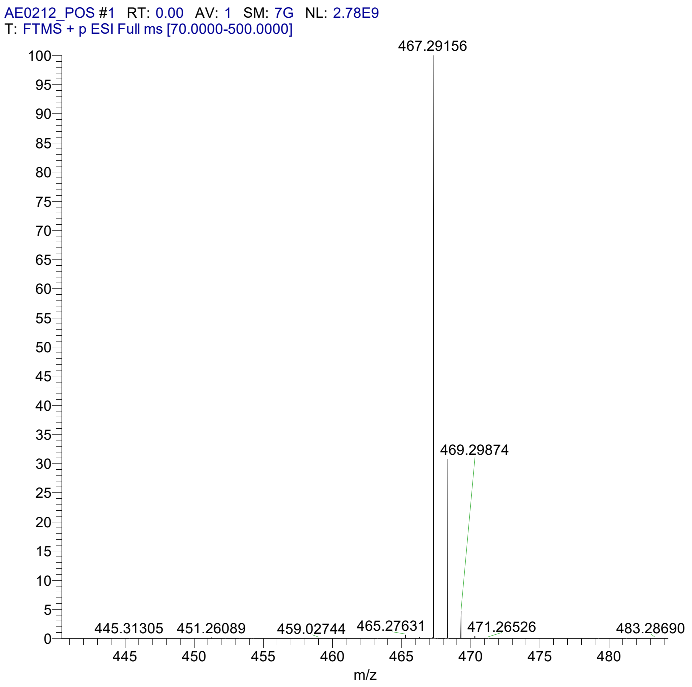

Figure S101: HRMS spectra of **11a**.

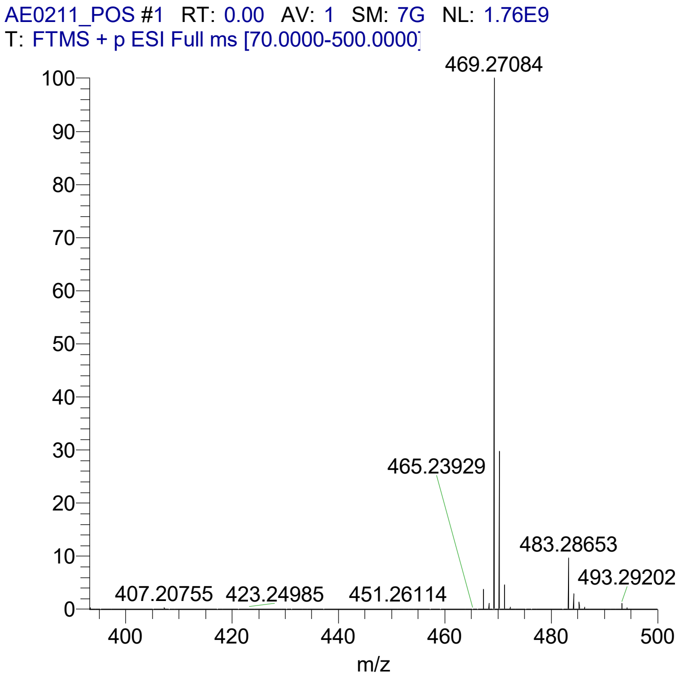

Figure S102: HRMS spectra of **11b**.

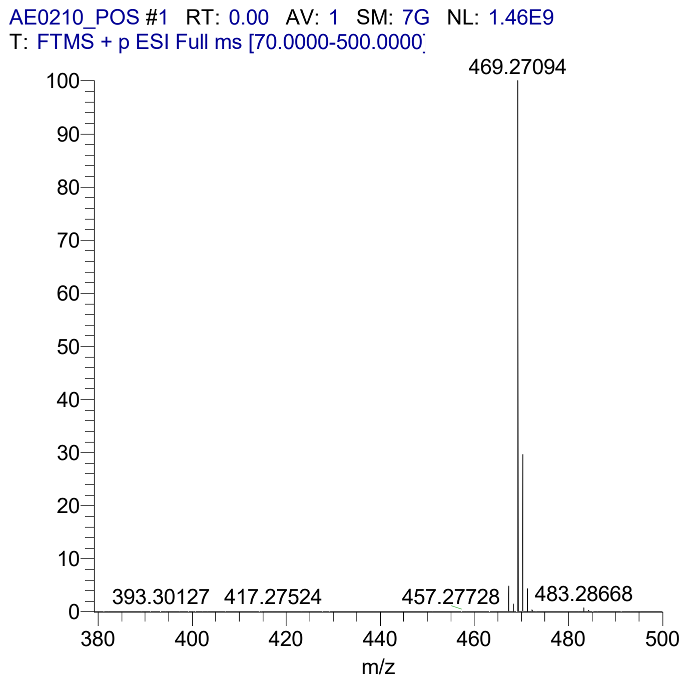

Figure S103: HRMS spectra of **11c**.

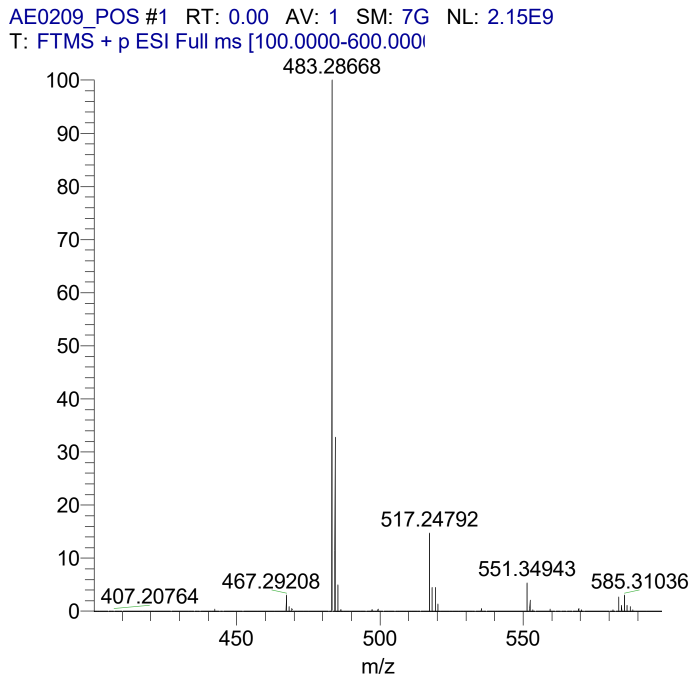

Figure S104: HRMS spectra of **11d**.

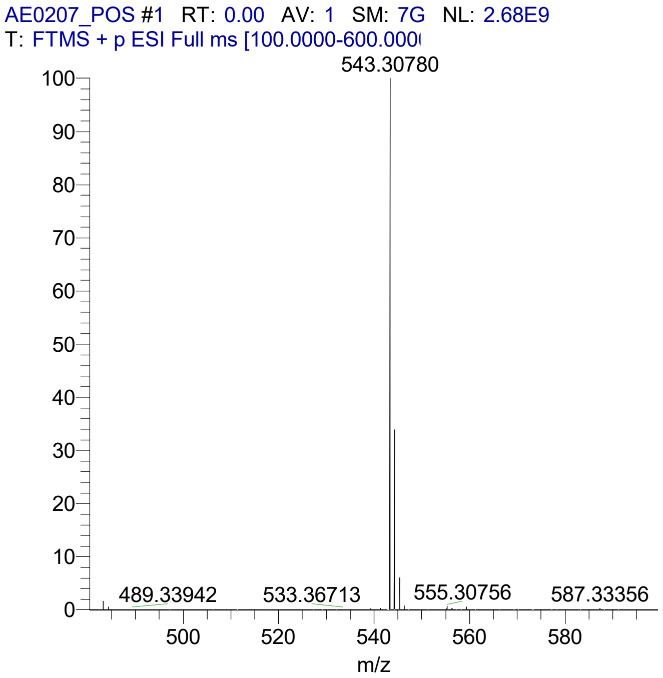

Figure S105: HRMS spectra of **11f**.

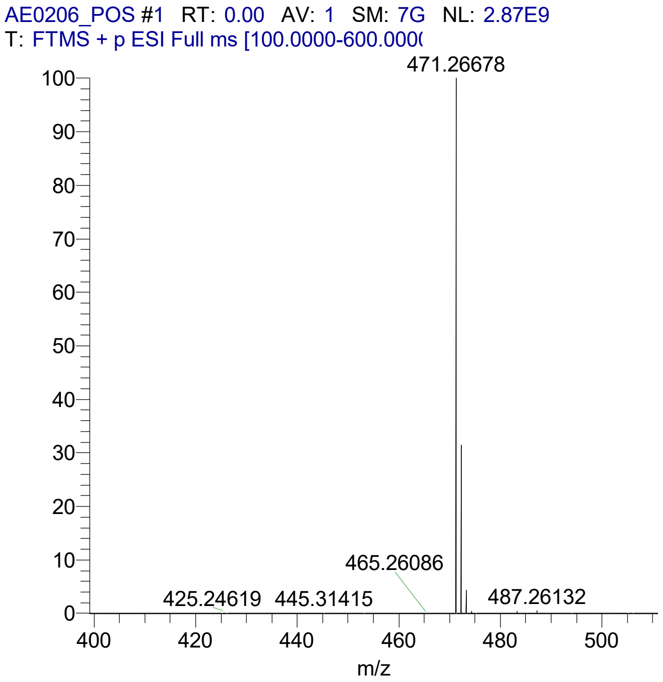

Figure S106: HRMS spectra of **11g**.

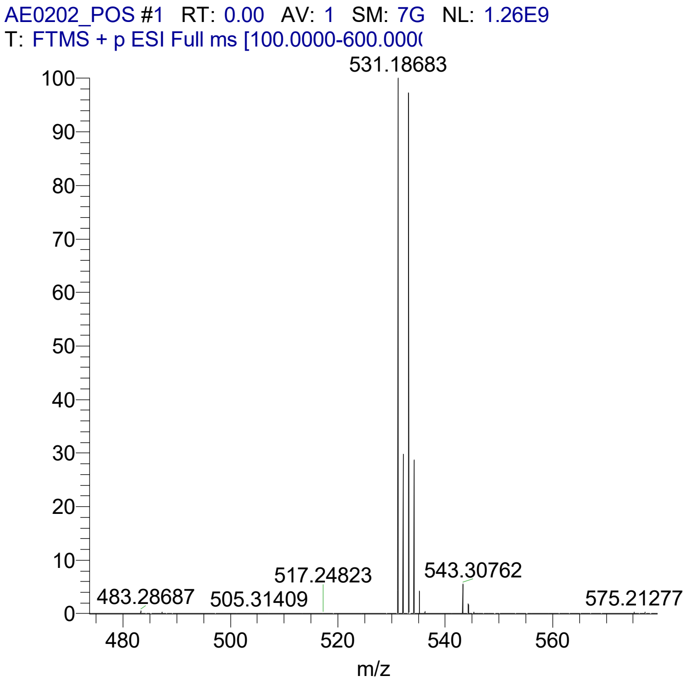

Figure S107: HRMS spectra of **11k**.

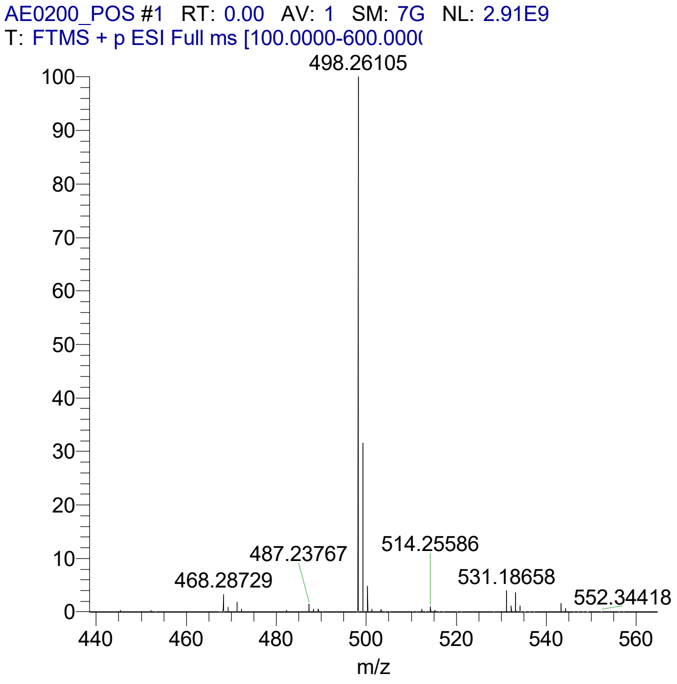

Figure S108: HRMS spectra of **11m**.

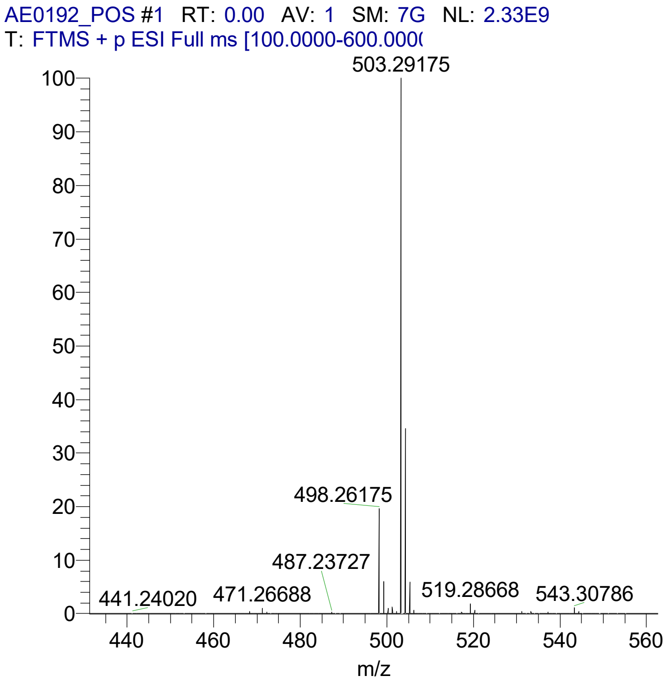

Figure S109: HRMS spectra of **11s**.

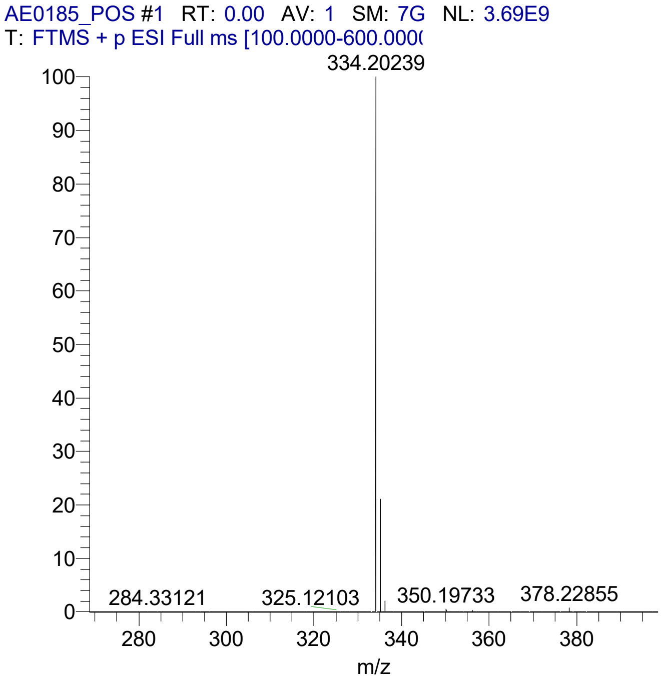

Figure S110: HRMS spectra of **21**.
